# Subunit fusion unlocks rapid *in vitro* maturation for slowly activating heterodimeric [FeFe]-hydrogenases

**DOI:** 10.1039/d5sc07299a

**Published:** 2026-02-03

**Authors:** Jan Jaenecke, Konstantin Bikbaev, Miriam Malagnini, Julia Bronold, Shanika Yadav, Ulf-Peter Apfel, Christophe Léger, James A. Birrell, Ingrid Span, Nicolas Plumeré, Martin Winkler

**Affiliations:** a Professorship for Electrobiotechnology, Technical University of Munich, Campus Straubing for Biotechnology and Sustainability Uferstrasse 53 94315 Straubing Germany martin-h.winkler@tum.de nicolas.plumere@tum.de; b Bioinorganic Chemistry, Friedrich-Alexander-Universität Erlangen-Nürnberg Egerlandstrasse 1 91058 Erlangen Germany; c Laboratoire de Bioénergétique et Ingénierie des Protéines (BIP), CNRS, Aix-Marseille Université 31 chemin Joseph Aiguier 13009 Marseille France; d Activation of Small Molecules/Technical Electrochemistry, Faculty of Chemistry and Biochemistry, Ruhr-University Bochum Universitätsstrasse 150 44801 Bochum Germany; e Department of Electrosynthesis, Fraunhofer UMSICHT 46047 Oberhausen Germany; f School of Life Sciences, University of Essex Wivenhoe Park Colchester CO4 3SQ UK; g Inorganic Spectroscopy, Energy Converting Enzymes, Max-Planck-Institute for Chemical Energy Conversion Stiftstrasse 34-36 45470 Mühlheim an der Ruhr Germany

## Abstract

Hydrogenases offer a sustainable alternative to noble metals for catalyzing H_2_-oxidation and H_2_-production. The heterodimeric [FeFe]-hydrogenase of *Desulfovibrio desulfuricans* ATCC 7757 (*Dd*HydAB) is most promising due to its exceptional catalytic activity and high-yield heterologous expression of its apo-form. Scalable production of the holo-form relies on *in vitro* maturation of the apo-enzyme using a chemically synthesized 2Fe_H_ cofactor mimic. However, the unusually slow *in vitro* maturation of *Dd*HydAB raises mechanistic questions and limits its scalability. Through structural and sequence analysis, we identified the cause of this slow maturation and redesigned the enzyme *via* subunit fusion, inserting short peptide linkers near the active site. This modification facilitates the rearrangement of a critical locking element after cofactor uptake, increasing the maturation rate by up to 41-fold without compromising catalytic performance. Our findings elucidate a key step in the plug-lock-lid mechanism underlying maturation and promote the industrial applicability of *Dd*HydAB.

## Abstract 2

Subunit fusion unlocks fast maturation for slowly maturating [FeFe]-hydrogenase *Dd*HydAB. Linking small and large subunit stabilizes active site closure after 2Fe_H_ uptake, accelerating H-cluster formation and activity development by 40-fold.

## Graphical abstract

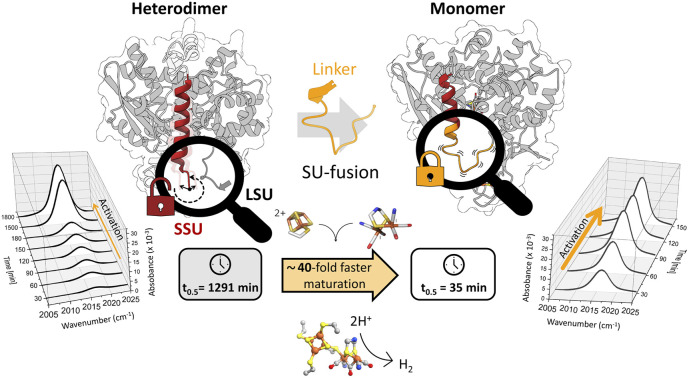

## Introduction

Hydrogenases are increasingly recognized as sustainable catalysts for H_2_-production and utilization in green energy systems and the (bio)chemical industry.^1–7^ Among the three existing main classes, called [Fe]-, [NiFe]-, and [FeFe]-hydrogenases, the latter stand out for their bidirectional H^+^/H_2_ interconversion at turnover frequencies that rival precious-metal catalysts.^8–10^ Previous studies highlight the superior features of individual [FeFe]-hydrogenases rendering them as promising candidates for future applications; this includes *Dd*HydAB and *Cb*A5H, both of which can reversibly adopt an inactive, O_2_-resistant state (H_inact_), permitting aerobic purification and facile sample handling.^11–15^ Recent publications have demonstrated scalable heterologous expression and provide the basis for their implementation in electrochemical systems.^6,7,16–18^

**Fig. 1:**
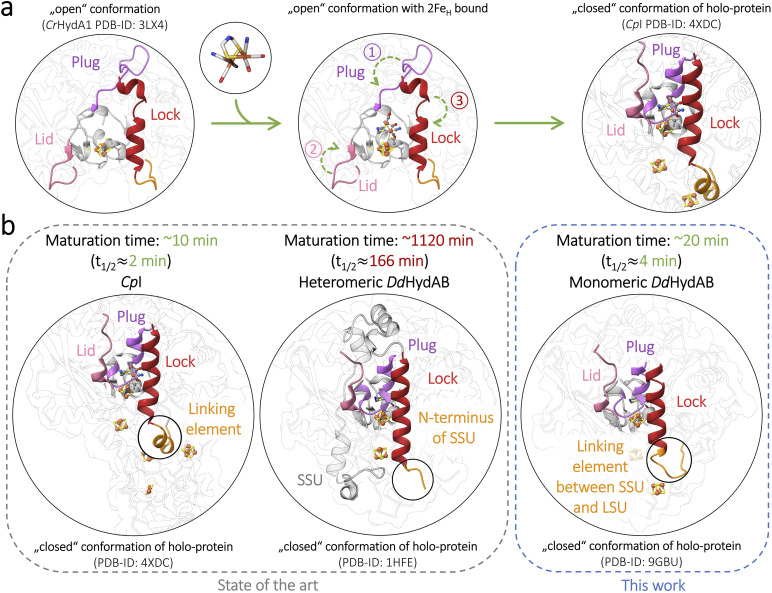
Final steps of H-cluster assembly in [FeFe]-hydrogenases *Cp*I and *Dd*HydAB after uptake of the 2Fe_H_ precursor. *In vivo*, the 2Fe_H_ precursor is biosynthesized by the three maturases HydE, HydG, and HydF (see Fig. S1). Alternatively, a mimic of the 2Fe_H_ precursor can be chemically generated and mixed with apo-hydrogenase to enable maturation of the H-cluster *in vitro*. (a) Upon insertion of the 2Fe_H_ precursor complex, the open H-cluster binding pocket is closed and locked by the “plug-lock-lid” system. (b) Closed-state structures of *Cp*I from *Clostridium pasteurianum* (PDB-ID: 4XDC) and *Dd*HydAB (PDB-ID: 1HFE), illustrating that the gap between the small (SSU) and large (LSU) subunit is located right at the N-terminus of the lock-element. This potentially impacts the rate of binding site closure after cofactor insertion. In the monomeric *Cp*I, the lock-element is stabilized by a native linker-element. Fusion of the small subunit (SSU, grey) and the large subunit (LSU, transparent) in the heterodimeric *Dd*HydAB *via* a linker peptide (orange) accelerates the maturation process by up to 41-fold (see *t*_1/2_ reduction in Table S2).

[FeFe]-hydrogenases offer a particularly convenient route to production at scales relevant for industry: their catalytic cofactor (H-cluster) comprises a [4Fe4S]-subcluster (4Fe_H_) covalently linked *via* one of its Cys ligands to an unusual [2Fe]-subcluster (2Fe_H_). In their apo-state, which lacks the 2Fe_H_-subcluster, [FeFe]-hydrogenases can be effectively overexpressed in *Escherichia coli*. The purified apo-form can be maturated *in vitro* to the active holo-enzyme by simply mixing the apo-hydrogenase with an excess of a synthetic 2Fe_H_ mimic complex (2Fe^MIM^_H_), which spontaneously enters the open cofactor-binding site (Fig. 1).^19–21^

Upon *in vitro* maturation, the heterodimeric M2-type [FeFe]-hydrogenase *Dd*HydAB of *Desulfovibrio desulfuricans* (identical to *Nitratidesulfovibrio vulgaris* and formerly labeled as *D. vulgaris*; see SI Discussion) reaches exceptionally high activities of 7400 s^−1^ (H_2_-evolution) and 56 000 s^−1^ (H_2_-oxidation),^21–23^ combined with the possibility of reversible sulfide-induced O_2_-protection.^14,15^ However, the production of the active *Dd*HydAB holo-enzyme still faces a major challenge.^21^ In contrast to the apo-states of monomeric [FeFe]-hydrogenases that fully activate within 15–30 min upon adding the 2Fe^MIM^_H_ complex,^20^*in vitro* maturation of *Dd*HydAB requires more than two days under similar conditions,^21^ lowering its application potential and motivating a mechanistic re-examination of the maturation process. *In vivo*, H-cluster maturation is assumed to be conserved among [FeFe]-hydrogenases of eubacteria and eukaryotes.^24–27^ When heterologously expressed in its apo-form, *Dd*HydAB is already equipped with the 4Fe_H_-subcluster along with its two accessory [FeS]-clusters characteristic of the M2-type architecture, while exhibiting a vacant but accessible 2Fe_H_ binding site. The native 2Fe_H_ precursor arises *via* the contribution of three maturases (HydG, HydE, and HydF). HydG, one of the two radical-SAM enzymes involved, generates the mononuclear [Fe^II^(CO)_2_ (CN)(Cys)]^−^ synthons. The other (HydE) combines two synthons to the 2Fe_H_-pre-complex [Fe^I^_2_(µ-SH)_2_(CO)_4_(CN)_2_]^2−^ before both of its Fe-sites are cross-bridged by the dithiomethylamine ligand, which is essential for H^+^-exchange between the proton transfer pathway and the active site (Fig. S1a).^27–32^ HydF serves as a scaffold protein for the biosynthesis of the 2Fe_H_ precursor before it is finally transferred from the mature HydF protein (holo-HydF) to the vacant cofactor-binding site of the apo-hydrogenase during a transient protein–protein interaction (Fig. 1 and S1b).^28,33–36^ Functional 2Fe_H_ insertion entails a rotation of the primary ligand sphere at the distal iron (Fe_d_) of the 2Fe_H_ precursor complex, favored by H-bond contacts between the two CN^−^ ligands and individual residues in the secondary ligand sphere,^30,37^ followed by a local reconfiguration of three structural elements first indicated by Mulder and coauthors in 2010.^26^ One element turns into a plug-like structure that loosely closes the binding site over the inserted 2Fe_H_ precursor, thereby supporting the adoption of its rotated conformation. Next, this plug placement is barred in place from two sides by a restructuring of both remaining loop elements, termed lid and lock (Fig. 1a and S1b).^24–26,30,31^ The coupling of the 2Fe^MIM^_H_ complex with the 4Fe_H_-subcluster is enabled by one of its Cys ligands, which adopts a bridging configuration (µ-SCys) between both subclusters (Fig. 1 and S1), yielding the transient H_red_CO state.^25,27,38^ A single oxidation step leads to the likewise inhibited but rather stable H_ox_CO state (Fig. S1c). Upon release of the apical CO ligand from Fe_d_, the oxidized active-ready state H_ox_ is formed.^25,27,38^

We previously hypothesized that *Dd*HydAB's unusually slow maturation results from a higher propensity of the apo-form to adopt the closed conformation.^21^ However, no attempts have been made to verify this idea or examine other potential reasons for the extremely low maturation rate of *Dd*HydAB, let alone to speed up the maturation process. We propose an alternative, structure-based explanation centered on its heterodimeric state: a superposition of the X-ray structure of *Dd*HydAB (1HFE)^39^ with those of monomeric enzymes (*Cr*HydA1, PDB ID: 3LX4; *Cp*I, PDB ID: 4XDC)^26,30^ illustrates that the C-terminus of the small-subunit (SSU) coincides with the end of the lock-element (Fig. S2). In monomeric enzymes, the lock-helix straightens upon 2Fe_H_ uptake, bolting the plug in its place over the occupied binding site; in the case of *Dd*HydAB, we hypothesize that the gap between SSU and LSU likely increases the flexibility of the lock-element at the free SSU N-terminus, hindering an efficient lock rearrangement and delaying the final closure of the pocket. Subunit fusion can be a powerful strategy to enhance the features of heterodimeric proteins, such as stability, yield, and catalytic efficiency.^40,41^ Linker-mediated coupling was also used to create chimeric catalysts with new functions.^40–42^

Here, we investigated whether a linker-based monomerization approach positively impacts the unusually low *in vitro* maturation rate of *Dd*HydAB. Employing FTIR spectroscopy and various kinetic measurements, we show that the rates of H-cluster formation and activity development of the monomeric variants increase by nearly two orders of magnitude relative to the heterodimeric wild-type.^21,24^ Protein-film electrochemistry and X-ray crystallography enable us to ensure the conservation of the advantageous traits of *Dd*HydAB and to rationalize the differences in the variant-specific maturation rates.

## Results and discussion

**Fig. 2:**
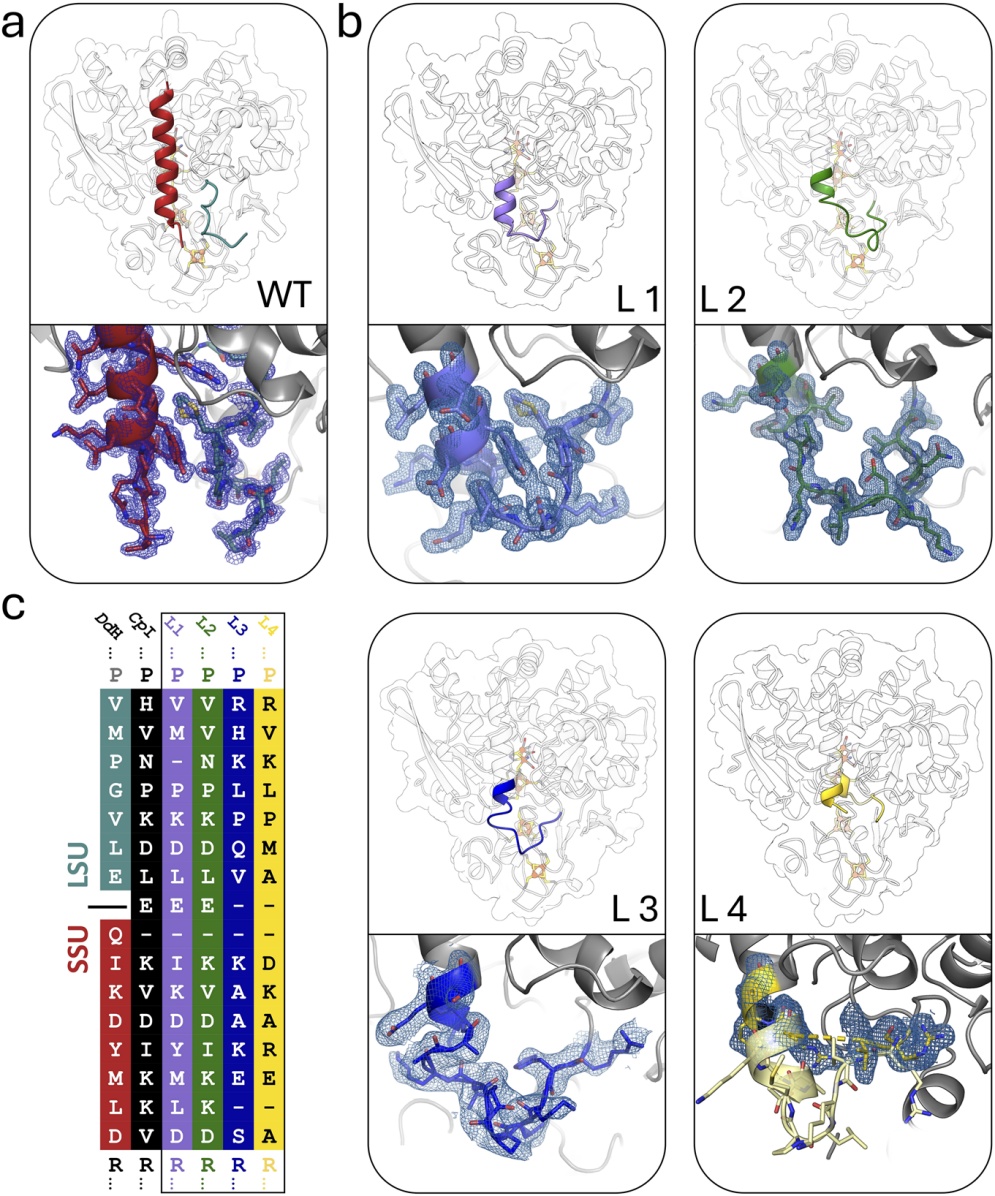
Design of linker elements for subunit fusion in *Dd*HydAB. (a) In the heterodimeric *Dd*HydAB (PDB ID: 9QD6), the N-terminus of the small subunit is marked in red and the C-terminus of the large subunit in cyan. For holo-*Dd*HydAB^WT^ and the monomeric fusion proteins L1–L3 (b) X-ray structures were solved including the linker region (PDB IDs: L1: 8RU6; L2: 9GNK; L3: GBU; L4: 8RYH). Linkers are colored according to the corresponding color code defined in (c) and shown as close-ups with their individual electron density as mesh contoured at 0.5*σ* (L1–L3). For L4, the low local resolution in the linker section does not allow to derive the corresponding electron density mesh. Here, the linker section of a model structure of L4 generated *via* Boltz-2 was superposed with the X-ray structure obtained for L4. (c) L1 and L2 were derived from the sequence and structural alignment between *Dd*HydAB and *Cp*I. L3 and L4 were adopted from sequences of monomeric homologs of *Dd*HydAB (see Fig. S4).

To investigate apo-*Dd*HydAB for possible structural differences from monomeric [FeFe]-hydrogenases, particularly in the terminal regions of the subunits and the structural elements responsible for the closure and locking of the holo-protein, we crystallized *Dd*HydAB in its unmaturated apo-form. The resulting crystal structure exhibited the closed state of the H-cluster binding site but did not show significant differences from the apo-state structure of the fast-maturating *Cp*I enzyme, which was likewise crystallized in its closed state (PDB ID: 4XDD)^30^ (Fig. S3a–e). As we were unable to support the hypothesis of a significantly higher tendency for premature binding-site closure in the case of *Dd*HydAB, we focused instead on the fact that its heterodimeric state is the most prominent structural difference compared to monomeric enzymes such as *Cp*I and *Cr*HydA1. The gap between the two *Dd*HydAB subunits coincides with the N-terminus of the lock-element, potentially affecting the 2Fe_H_ binding-site closure in the H-domain, and thus the rate of *in vitro* activation. To examine this hypothesis experimentally and with the aim of improving the maturation rate of *Dd*HydAB, we engineered four SSU–LSU fusion constructs (variants L1–L4) by inserting linkers of defined length and sequence (Fig. 2).

For the linker design, we followed two strategies: (1) structural mimicry with *Cp*I: based on a structural alignment between *Dd*HydAB (1HFE)^39^ and the monomeric *Cp*I (PDB ID: 4XDC)^30^ two linkers were designed (L1–2) with sequence homology and structural features corresponding to the native linker element in *Cp*I (Fig. 2, L1–L2, and S4a, b). (2) Native linker sequences of monomeric homologs: we optimized the structural integration of the linker sequence into the protein environment to avoid destabilization and steric clashes. From a multiple sequence alignment, we selected two monomeric homologs of *Dd*HydAB (M2-type [FeFe]-hydrogenases of *Solobacterium moorei* and *Veillonella atypica*; see corresponding sequences in Fig. S5) with a linker sequence coupling the parts that correspond to the SSU and LSU of *Dd*HydAB (Fig. 2, L3–L4, and S4a, b). The resulting fusion proteins were heterologously expressed, purified (Fig. S6), and compared to the dimeric wild-type enzyme for their impact on the rate of *in vitro* maturation, comparing two independent approaches: (i) we used IR-spectroscopy to monitor the rate of H_ox_CO formation as a product of 4Fe_H_/2Fe_H_-cluster coupling during maturation in a sealed sample of apo-enzyme upon 2Fe^MIM^_H_ addition. (ii) We followed the activity development of the apo-hydrogenase variants upon addition of a 3-fold excess of 2Fe^MIM^_H_ by periodically testing aliquots for the rate of H_2_-evolution, which requires CO release from H_ox_CO.

### Electrochemical investigation of CO-binding kinetics and spectroscopic tracking of H-cluster formation rates

**Fig. 3:**
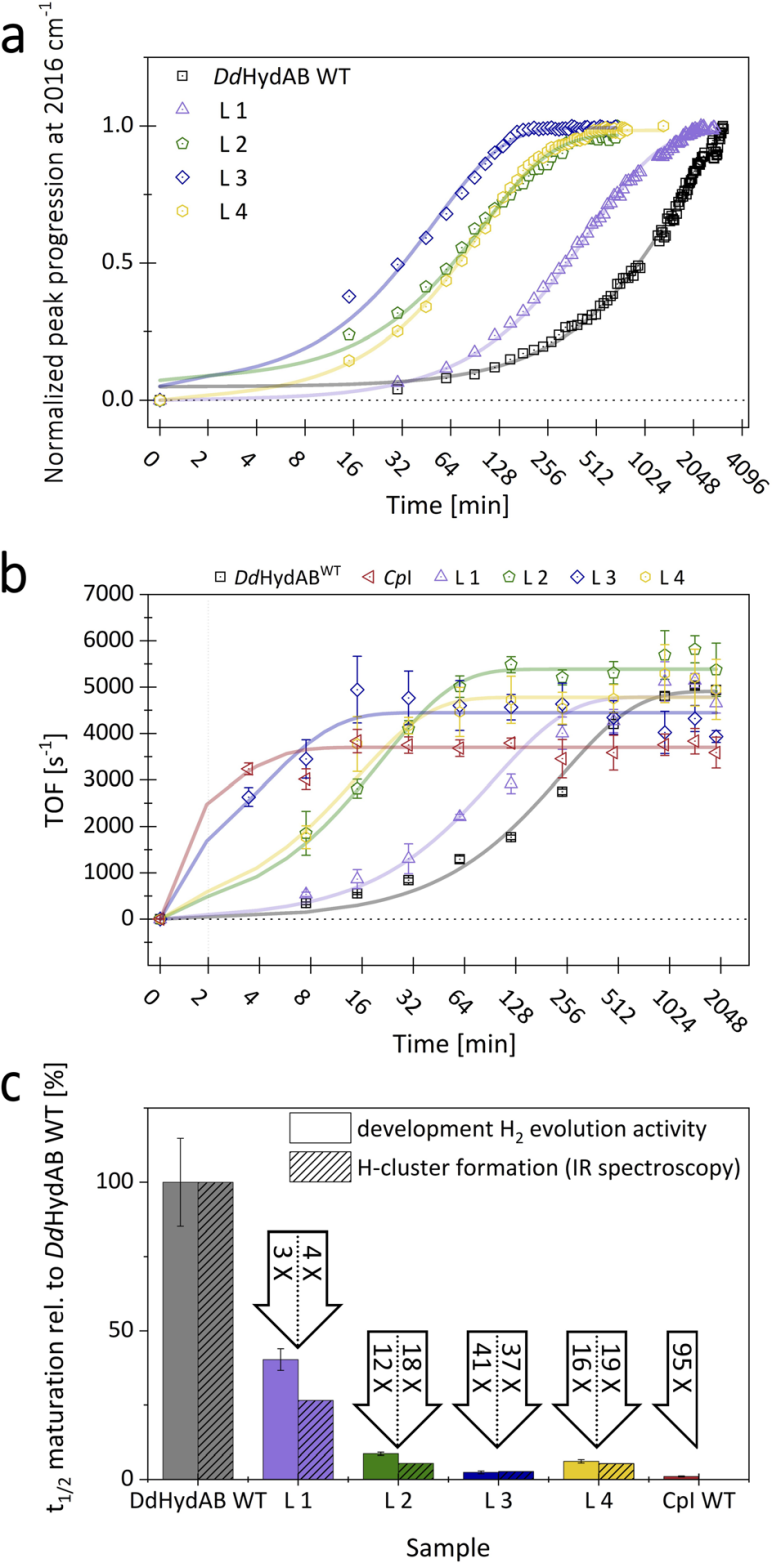
Comparison of *in vitro* maturation rates for the apo-states of *Dd*HydAB^WT^ and monomeric fusion variants L1–L4. (a) H-cluster formation was followed *via* FTIR spectroscopy by the progression of the amplitude of the indicator peak for H-cluster state H_ox_CO at 2016 cm^−1^. (b) *In vitro* maturation was tracked by monitoring the development of H_2_-evolution activity over time in aliquots of *in vitro* maturation stock samples (error bars: standard deviation of biological dublicates (*n* = 2) and technical triplicates (*n* = 3)). (c) Comparison of the half-times for H-cluster formation and activity development during *in vitro* maturation for variants L1–L4 relative to the heterodimeric WT. The factor by which the half-time of the *in vitro* maturation process is decreased is indicated by the corresponding arrow above each bar (error bars: standard error of the fit parameters based on a covariance matrix of the parameters).

The production of the active enzyme from the H_ox_CO state requires the final release of the apical CO ligand that blocks the substrate binding site (Fig. S1c). Among the best-characterized [FeFe]-hydrogenases, *Dd*HydAB shows the lowest inhibition constant (*K*_i_) for external CO.^39^ We exploited this to track the amplitude increase of a characteristic peak at 2016 cm^−1^ in the IR-spectrum of H_ox_CO between 0 and 68 h (Fig. 3a; see Fig. S9 for full spectra) as a reporter for plotting the rate of H-cluster formation during the maturation process.

To ensure that all monomeric variants retain the high CO affinity of the wild-type enzyme, we employed protein film electrochemistry to monitor the kinetics of CO binding and release. In this classical chronoamperometry experiment, a film of holo-enzyme is prepared on the pyrolytic graphite surface of a rotating disk electrode.^43^ The H_2_-oxidation current is measured as a function of time before and after the enzyme is exposed to CO (Fig. S8). For all proteins, we determined and compared the Michaelis–Menten constants for H_2_ (Fig. S7) and the inhibition constants for CO (*K*^CO^_i_). Both the binding affinity for the substrate (H_2_) and the affinity for CO (*K*_i_ = *k*_out_/*k*_in_) are similar among all proteins (Table S1) with maximum differences among all proteins remaining well below a factor of two. The final CO-release step is associated with a *K*_i_ in the nM range, which confirms that the equilibrium of the reaction H_ox_CO ⇔ H_ox_ + CO lies on the left side (H_ox_CO). This especially applies to the conditions of the FTIR-spectroscopy experiment, as throughout the maturation process, the samples are kept in a tightly sealed cell, which maintains a high CO concentration and excludes further competition with the substrate (H_2_), thus ensuring a homogeneous accumulation of the H_ox_CO-state (Fig. S1c).^21^

Although *Cp*I would be an ideal positive control for observing the H-cluster formation rate, its rapid activation and low enzyme-specific CO–ligand binding affinity prevent linear monitoring of the H-cluster formation rate using the state specific IR spectrum of H_ox_CO.

For each protein, we determined the times required to reach 50% cofactor saturation (half-times; *t*_1/2_) (Fig. 3c). All monomeric variants exhibited a significantly higher H-cluster formation rate compared to the heterodimeric wild-type, albeit to varying degrees. Based on the individual *t*_1/2_-values for the progression to full H-cluster occupancy, the rates of H-cluster formation can be ranked as follows: L3 > L2 ≈ L4 > L1 >> *Dd*HydAB. With a 3.8-fold enhancement compared to *Dd*HydAB^WT^, the linker of variant L1 showed the lowest impact on the enzyme's maturation rate. Fusion proteins L2 and L4 displayed an 18- and 19-fold increase in the H-cluster formation rate, while variant L3 exhibited the highest impact with a 37-fold increase in the H-cluster formation rate relative to *Dd*HydAB^WT^.

### Kinetics of enzyme activation monitored by solution assays

To assess *in vitro* maturation beyond H-cluster formation, we further monitored the activation to the catalytically competent holo-enzyme by periodically testing aliquots from an *in vitro* maturation mix for the enzyme's H_2_-evolution rate. This was achieved through a well-established sodium-dithionite-driven and methyl viologen (MV)-mediated activity assay measured *via* gas chromatography (Fig. 3b).^20,24^ This approach enabled the inclusion of *Cp*I as a positive control. The hydrogen evolution assay mirrored the FTIR-based maturation trends, confirming its overall ranking among the compared enzymes. All proteins reached their maximum activity within 34 h, consistent with previous data on the *in vitro* maturation of *Dd*HydAB.^21^ While variant L1 exceeded the maturation rate of WT by reaching its full activity after approximately 7.4 h (see Table S2), it was nevertheless the slowest among the four monomeric *Dd*HydAB-variants. In contrast, variant L3 showed an extraordinarily rapid activation with a half-time of merely 4 min, which nearly reached the activation rate of *Cp*I (*t*_1/2_: 1.7 min), being one of the fastest-maturating hydrogenases reported to date (Fig. 3c).^20^ Fusion variants L2 and L4 behaved similarly, peaking at a turnover frequency of around 5000 s^−1^ after 95 and 68 min, respectively, with variant L2 achieving a higher turnover rate than L4 and the heterodimeric wild-type. To verify that *in vitro* maturation is discontinued once the aliquot is added to the *in vitro* assay reaction mix, we prepared a control sample using the fastest-maturating *Dd*HydAB-variant L3. 2Fe^MIM^_H_ and the apo-form of variant L3 were separately diluted and combined in the assay mix right before sample incubation. Under these conditions even L3 showed negligible H_2_-production (37 s^−1^), confirming that the *in vitro* maturation process is effectively stopped when added to the reaction mix of the assay.

Although the enzyme to 2Fe^MIM^_H_ ratio (1 : 3) was the same for both approaches compared here, other experimental conditions differed due to the constraints of the respective methods (*e.g.*, enzyme concentration and incubation in open *vs.* closed vessels). These differences account for the 5-9-fold deviations in absolute *t*_1/2_-values for the same enzymes (compare absolute *t*_1/2_ values for FTIR and the HER of the same protein in Table S2). We normalized the half-times of *in vitro* maturation for the different fusion proteins relative to the wild-type (set to 100%). For the same enzyme variants, the relative *t*_1/2_ values (rate increase compared to wild-type) obtained on the one hand by monitoring H-cluster formation *via* FTIR and on the other hand by measuring H_2_-evolution activity development (HER) aligned closely for all variants (L1: 4-fold/3-fold; L2: 18-fold/12-fold; L3: 37-fold/41-fold; L4: 19-fold/16-fold). Remarkably, L3 shows, in both approaches, by far the highest relative increases compared to the other variants. Its relative HER rate increase is only 2.3-fold lower than the relative rate observed for the monomeric reference enzyme *Cp*I (95-fold; see Table S2). Overall, all linkers enabled a significant increase in the *in vitro* maturation rate, which was likewise reflected in the rates of H-cluster formation and activity increase. This suggests that the heterodimeric state either slows down subcluster coupling or affects an earlier step in the maturation process. This bottleneck could involve the uptake of the 2Fe_H_ precursor, which may be blocked by an unfavorable configuration of the free N-terminus at the lock-element or arise from steps further downstream. It may concern an early state, with the precursor complex 2Fe^MIM^_H_ being embedded in the binding pocket but not yet covalently coupled to the 4Fe_H_-subcluster. Such a state was postulated and experimentally supported to precede subcluster coupling as an inactive intermediate species by Megarity and coauthors.^25^ Cluster coupling only happens if the applied potential is high enough to oxidize the 4Fe_H_-subcluster, while the uncoupled intermediate state is conserved at potentials below −0.54 V. Megarity *et al.* described the 2Fe^MIM^_H_ complex to be tightly bound, withstanding several buffer exchanges even at potentials below −0.54 V, where no subcluster coupling occurred. This suggests that after 2Fe_H_ uptake, stable binding site closure occurs and is a prerequisite of subcluster coupling. Thus, the unbound 2Fe^MIM^_H_ complex could be easily lost if binding site lockage *via* the plug, lock, and lid reconfiguration is incomplete.^24,30^ It can be assumed that subcluster-coupling *via* the formation of a bridging Cys ligand not only requires a suitable potential range but also a complete and stable H-cluster environment, granted by a sturdy closure of the open 2Fe_H_ binding site. The connection between the heterodimeric state and slow maturation rate supports this hypothesis. In *Dd*HydAB^WT^, the flexible N-terminus of the small subunit slows down the locking of the plug-element over the 2Fe_H_ binding site after 2Fe^MIM^_H_ uptake, as it lacks the stable resistance and anchoring of the terminal helix segment that is needed for a rapid structural rearrangement at the other end of the helix (Fig. 1a, b, and S1b).

### Enzyme yields and O_2_ protection

**Fig. 4:**
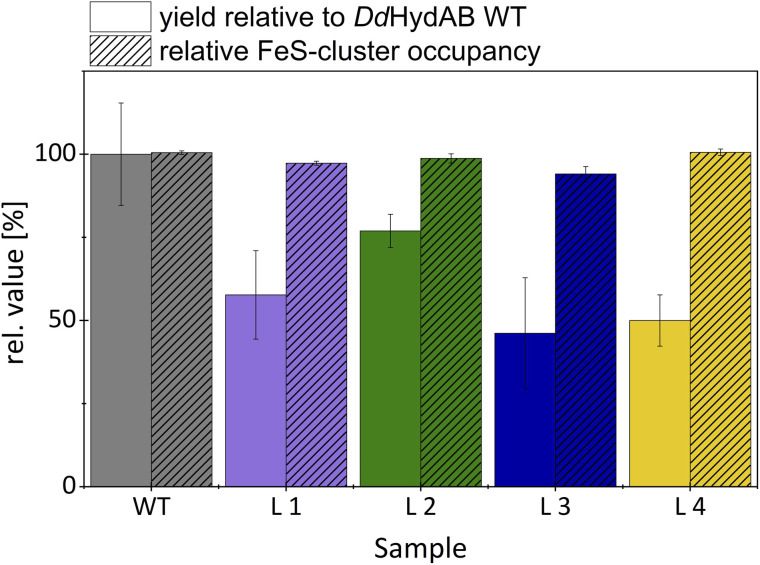
Protein yields from heterologous expression and [FeS]-cluster occupancies of the monomeric *Dd*HydAB-variants relative to wild-type. Error bars represent standard deviations from three independent purifications and technical replicates (*n* = 3).

**Fig. 5:**
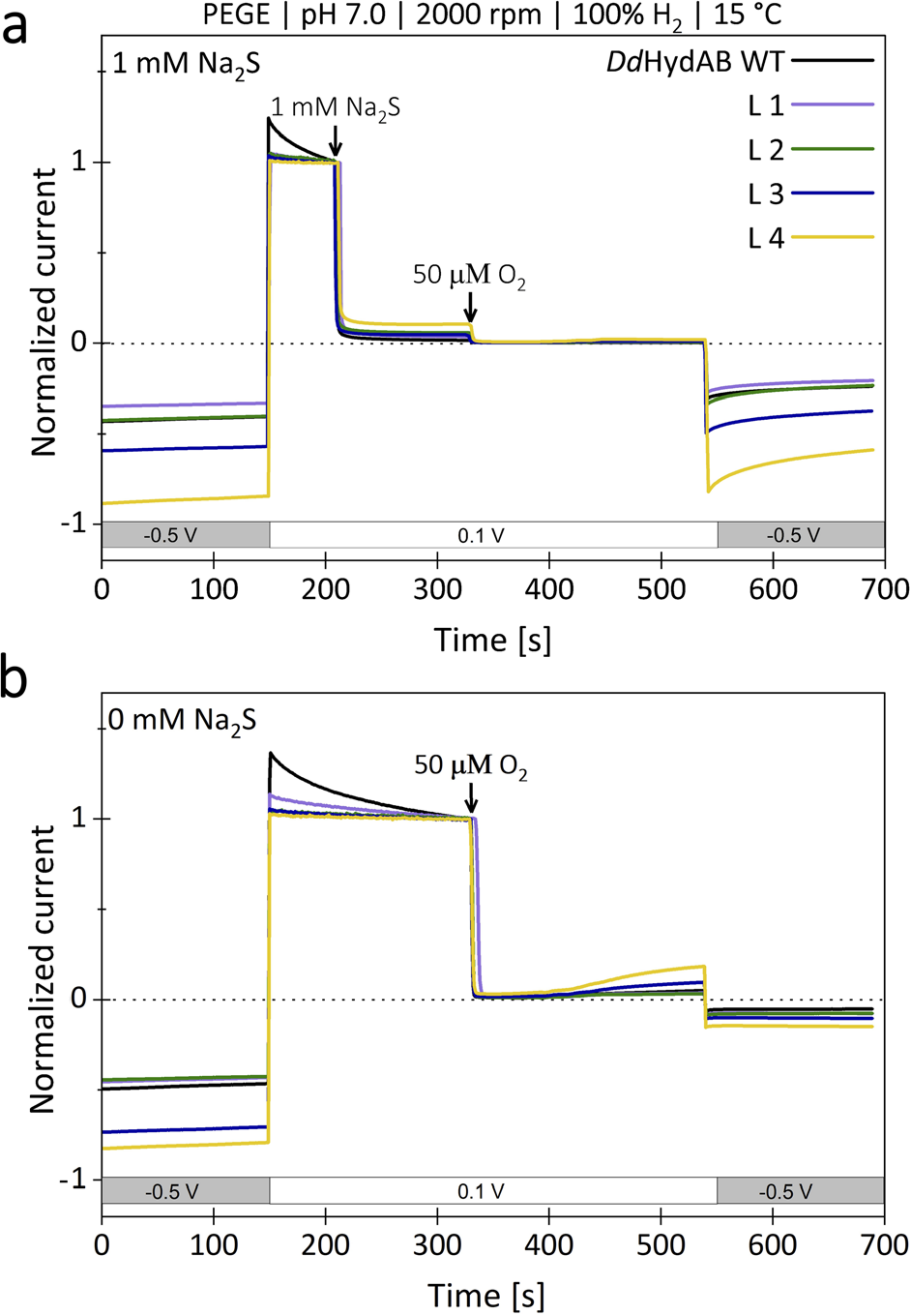
Potential-step voltammetry of *Dd*HydAB^WT^ compared to the monomeric variants L1–L4 (a). When adding Na_2_S under oxidative conditions (0.1 V *vs.* SHE), all monomeric variants show the same, well-characterized inactivation as the heterodimeric *Dd*HydAB^WT^. When adding O_2_-saturated buffer to a final O_2_ concentration of 50 µM under these conditions, only a slight decrease in current can be observed for all proteins. By shifting the potential of the working electrode back to reducing conditions (0.5 V *vs.* SHE), all monomeric proteins show a residual activity comparable to that of *Dd*HydAB. (b) If O_2_-saturated buffer is added without prior addition of Na_2_S, almost no activity is recovered under reducing conditions, which likewise applies to all tested proteins.

Considering that *Dd*HydAB is a prime candidate for future biotechnological applications that include H_2_-driven or H_2_-producing processes, it is crucial to preserve the beneficial characteristics of the original enzyme in its monomeric variants. Therefore, we investigated the monomeric fusion proteins for their heterologous expression yields, [FeS]-cluster occupancy (Fig. 4), and the possibility to provide effective protection against O_2_ by transiently turning the sample into the sulfide-bound H_inact_ state (Fig. 5).^14^

We determined differences in the yields from heterologous expression and protein purification between the monomeric variants and the heterodimeric wild-type (Fig. 4). Based on an average of three independent protein purifications, we calculated for L3 the lowest relative yield of 46% compared to *Dd*HydAB^WT^. For fusion variants L4 and L1, the relative yields were slightly higher (50% and 58%). Variant L2, which already enabled an approximately 15-fold maturation rate increase, not only reached and maintained the highest H_2_-evolution rate (118% compared to wild-type) but also exhibited the highest relative expression yield of 77%. The retention of the protective effect of sulfide treatment against molecular O_2_ was examined *via* potential-step voltammetry with (Fig. 5a) and without the addition of 1 mM Na_2_S prior to an injection of 50 µM O_2_ (Fig. 5b), as previously described by Rodríguez-Maciá and coworkers.^14,15^ After determining the original level of catalytic current resulting from H_2_-evolution at −0.5 V *vs.* SHE, the potential was stepped up to 0.1 V *vs.* SHE supporting H_2_-oxidation. Under such oxidative conditions, as soon as 1 mM Na_2_S is added to the measuring solution, all four monomeric variants show a rapid and complete loss of catalytic current just like *Dd*HydAB^WT^ (Fig. 5a), indicating for all proteins a likewise effective and potential-dependent inactivation *via* sulfide-binding to the active site. To further verify that the attached sulfide ligand protects the active site against O_2_-induced damage, O_2_-saturated buffer was injected to reach a final O_2_ concentration of 50 µM in the measuring buffer. For the sulfide-treated enzyme sample, the addition of O_2_-saturated buffer only caused a minimal loss of residual activity. When triggering the release of sulfide from the active site by switching back to −0.5 V *vs.* SHE, the activities of all investigated proteins recovered accordingly. A comparison between the initial H_2_-evolution current and the reductive current after O_2_-exposure suggests for WT and all monomeric variants a comparable decrease in activity, mostly due to film loss from the electrode surface, while in all cases only a very small fraction of the enzyme is irreversibly inactivated by O_2_. To compare the impact of O_2_-exposure on enzyme activity between sulfide protected and unprotected samples, a second series of experiments was performed in which O_2_ exposure was carried out without prior sulfide addition (Fig. 5b). As expected, all enzyme variants show an instantaneous and complete loss of catalytic activity upon O_2_-addition, which is not recovered upon stepping the potential back to −0.5 V *vs.* SHE. Thus, all monomeric proteins can be effectively protected against irreversible O_2_-induced inactivation *via* pre-exposure to sulfide (Fig. 5a). When kept anaerobically, each variant retains a high catalytic activity comparable to *Dd*HydAB^WT^ (Fig. 3b).

### Structural analysis

When designing the four linker elements, we aimed at covering a range of lengths and amino acid compositions, considering features such as bulkier side chains that influence the local flexibility based on preceding AlphaFold modelling.^44^ Accordingly, different characteristics emerged in maturation efficiency, H_2_-evolution activity, and heterologous expression. To elucidate the structural basis for the strong impact of subunit fusion on enzyme maturation of *Dd*HydAB and to further identify the steric individualities responsible for differences in maturation rate increases between L1 and the other variants, we solved the crystal structures of variants L1–L4. Crystals of variants L1 and L4 were obtained under similar conditions to those previously established for the wild-type,^45^ whereas variants L2 and L3 required additional optimizations of the crystallization conditions. Diffraction data were collected at beamline P11 at PETRA III (DESY, Hamburg, Germany)^46,47^ and structures of all monomeric variants were determined at resolutions of 1.05–1.78 Å (Table S3).

The linker regions in variants L1, L2, and L3 showed clear electron densities, enabling the modelling of all amino acid residues as well as the tracking of interactions with or within the corresponding loop region (Fig. 2b, S5f, k and p). However, the structures of variant L4 lacked sufficient electron density in the linker segment (Fig. S10), which indicates high mobility of the linker residues. Overall, the protein backbones of the variants are very similar to the wild-type protein, suggesting that the linkers do not affect the overall fold of the protein. We employed ChimeraX^48^ to superpose a Boltz-2 model^49^ of L4 with the well-resolved remaining part of the corresponding crystal structure, offering a plausible suggestion for the unresolved linker region in the L4 structure (see Fig. S10).

The 2Fe_H_-subcluster is present in all structures, but with different occupancy levels. In variants L1 and L2, the occupancy of 2Fe_H_ has been refined to 0.9 to better match the experimental electron density, while in variants L3 and L4 the 2Fe_H_-subcluster is fully occupied.

L1 and L2 show a well-defined diatomic ligand bound to the open coordination site of Fe_d_ with occupancies of 0.5 and 0.9, respectively. We propose that this diatomic ligand corresponds to a CO molecule derived from the synthetic complex used for the maturation of the enzymes. Electron density maps of L3 and L4 show very little positive density at the open coordination site in the *F*_o_–*F*_c_ maps, suggesting that most of the molecules in these crystals possess an open coordination site.

**Fig. 6:**
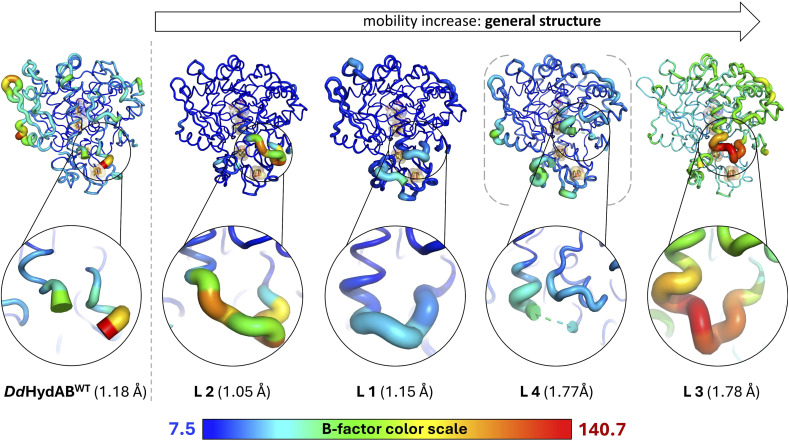
Illustration of the impact of local *B*-factor differences on protein stability (general structure) and maturation-rate increase (linker). Structures of *Dd*HydAB^WT^ (PDB ID: 9QD6) and SU-fusion variants L1-L4 (PDB IDs: 8RU6; 9GNK; 9GBU; 8RYH) are shown in the *B*-factor putty mode of PyMOL with close-ups of the linker elements. The highest *B*-factor among all structures is colored red and the lowest in dark blue, as indicated by the *B*-factor scale bar. The maximum and minimum values are presented at the corresponding ends of the color scale. The thickness of the protein backbone is also proportional to the *B*-factors. The H-cluster and accessory [FeS]-clusters are displayed as sticks and semi-transparent spheres. This image was generated with PyMOL 2.1.0.

While the structures of all the variants are overall very similar, we observed significant differences in the local mobility between the variants, as highlighted by the visualization of the crystallographic displacement factors (*B*-factors) in Fig. 6. The displacement of atoms from their mean position in a crystal structure may be the result of temperature-dependent atomic vibrations or static disorder in a crystal lattice. Since all structures were determined from crystals with the same lattice, the different *B*-factors observed for wild-type and the monomeric variants are more likely to be the result of local mobility. As expected, the heterodimeric *Dd*HydAB^WT^ structure shows increased mobility values at the termini of the two subunits, especially at the carboxy terminus of the LSU. The overall mobility of the structures for L1 and L2 is very low; however, the linkers of all variants show individual local flexibilities. The linker of the rather slowly maturating variant L1 is the least mobile one, while the longer linker of L2 is significantly more flexible. Interestingly, the linker of the fastest-maturating variant L3 shows the highest mobility. The L3 structure also shows the highest overall mobility range. However, as the structure of variant L3 was solved at a comparatively lower resolution, the increased *B*-factors may not be directly comparable with the *B*-factors determined for the high-resolution structures of L1 and L2. Crystal structures of L3, unlike those of L1 and L2, showed very low occupancies for the surplus CO ligand at the substrate binding site. This may suggest a lower CO-binding affinity or a faster release of the extra ligand, which could result from increased structural mobility compared to the wild-type and the other variants. Even though L3 indeed exhibits the highest *k*_react_ value among the five proteins, the difference to the other proteins is not very pronounced (see Table S1). Linker 1 showed by far the least impact on the enzyme maturation rate, being 4.5- to 10-fold lower compared to the other fusion proteins (Fig. 3c). According to a comparison of the side chain packing densities in the linker regions of X-ray structures obtained for L1, L2, and L3, the linker of L1 seems to be more tightly packed, while the side chains of linkers in L2 and L3 are comparatively more loosely embedded (Fig. S11). Thus, they may provide sufficient room for flexible movements (Fig. 2b, S5a, f, k and p). This is likewise reflected in the rather low *B*-factors assigned to linker 1 (Fig. 6), resulting in high local structural quality, while the higher *B*-factors for the linkers in structures of L2 and L3 rendered the local quality in the linker regions rather low compared to the remaining structural parts (Fig. 6). These results suggest that stabilization of the N-terminal part of the lock-helix by a linker supports the transition to the stably locked configuration after 2Fe_H_ precursor uptake. However, a certain degree of residual flexibility may still be necessary to permit and buffer movements during the structural reconfiguration. In the final section, we examine whether the unusually slow maturation rate of the heterodimeric wild-type enzyme is an intrinsic property of *Dd*HydAB or merely an artifact of the non-native conditions used for *in vitro* maturation.

### Impact of the maturase HydF on maturation

**Fig. 7:**
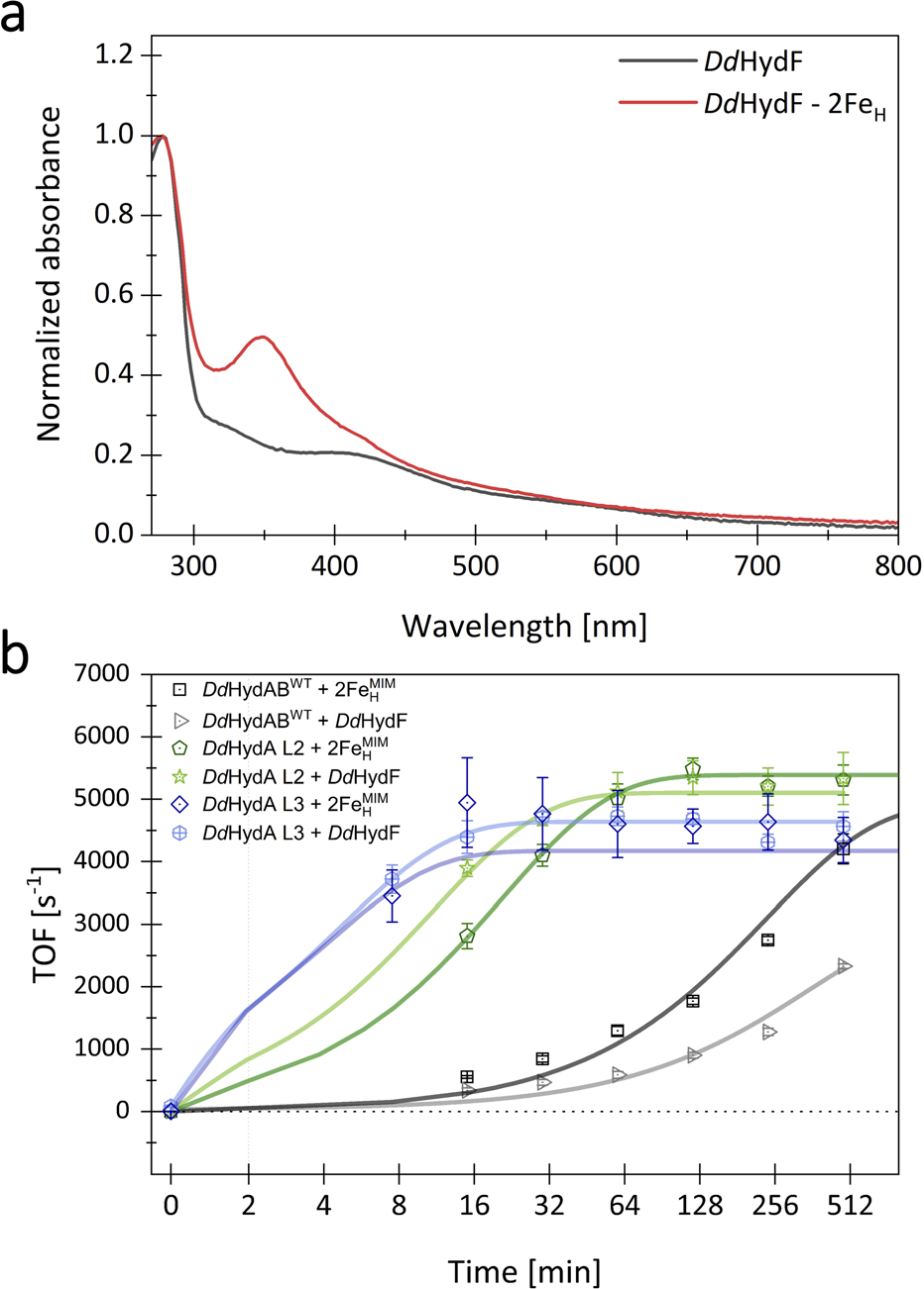
Loading and use of *Dd*HydF as a mediator for *in vitro* maturation of *Dd*HydAB^WT^ and subunit-fusion variant L2. (a) UV-Vis-spectra of as-purified *Dd*HydF and *Dd*HydF loaded with 2Fe^MIM^_H_. The spectra were normalized by setting the peak at 280 nm to 1.0. Protein samples were diluted with 0.1 M Tris–HCl buffer, pH 8. Both spectra are in line with those earlier reported for the apo- and cofactor-loaded holo-state of HydF from *Thermotoga maritima*.^28^ (b) Monitoring the time-dependent increase of H_2_-evolution activity for *Dd*HydAB^WT^ and subunit-fusion variants L2 and L3 during *in vitro* maturation of 0.16 mM hydrogenase, using either a 3-fold concentration of maturase-free 2Fe^MIM^_H_ or holo-HydF loaded with 2Fe^MIM^_H_.

So far, we have only investigated the change in maturation rate upon monomerization with different linkers of *Dd*HydAB by activating the apo-enzyme *in vitro* with free 2Fe^MIM^_H_. However, *in vivo*, the 2Fe_H_ precursor is assembled by the maturases HydE and G on the scaffold maturase HydF. Once loaded, HydF is proposed to transfer the 2Fe_H_ precursor to the apo-hydrogenase by forming a transient HydF/2Fe^MIM^_H_/apo-*Dd*HydAB complex. Intermolecular interactions in this ternary complex may stabilize the N-terminus of the small subunit of *Dd*HydAB, thereby enabling fast 2Fe_H_ insertion, comparable to the situation in the monomeric *Dd*HydAB versions. To investigate the influence of HydF-supported maturation, we expressed, purified, and loaded *Dd*HydF of *D. desulfuricans* ATCC 7757 (for sequence of *Dd*HydF, see Fig. S5), as previously done to study the interactions of HydF with different [FeFe]-hydrogenases (Fig. S1).^33–35^ Loading of *Dd*HydF was verified *via* electronic absorption spectroscopy (UV-Vis range) (Fig. 7a). HydF-supported activation was tested for the heterodimeric *Dd*HydAB^WT^, the monomeric variant L3, showing the highest activation rate with free 2Fe^MIM^_H_, and L2, as this variant showed an improved H_2_-evolution activity even in comparison to *Dd*HydAB^WT^. The activation was tracked using the same assay as before for *in vitro* maturation, with free 2Fe^MIM^_H_ replaced by equimolar amounts of 2Fe^MIM^_H_-loaded *Dd*HydF (Fig. 7b). The maturation rates achieved by HydF-mediated activation of the apo-enzyme largely reflect the trends observed when activating the apo-enzyme with free 2Fe^MIM^_H_. *Dd*HydAB fusion variant L2 shows an even lower *t*_1/2_-value of 8.7 ± 0.5 min in the HydF-mediated approach, compared to 14.3 ± 0.9 min when being activated by free 2Fe^MIM^_H_. L3 reaches a *t*_1/2_-value of approximately 4.7 ± 0.9 min in the HydF-supported assay, being only slightly higher than measured for the unmediated assay (2.3 ± 1.1 min). While the monomeric fusion variants were found to show largely comparable half-times for activation in the unmediated and HydF-supported assay, *Dd*HydAB^WT^ even showed a two-fold increase in *t*_1/2_ (341 ± 154 min compared to 166 ± 25 min). These results suggest that the slow *in vitro* maturation of *Dd*HydAB^WT^ cannot be overcome by the formation of a transient protein–protein complex with *Dd*HydF during the maturation process. Nevertheless, we cannot exclude alternative explanations based on the specific *in vivo* maturation conditions of periplasmic [FeFe]-hydrogenases such as *Dd*HydAB^WT^ (see SI file S2). Our results, however, underscore the importance of subunit fusion, as exemplified by L2 and L3, for achieving high maturation rates, irrespective of the mediator function of HydF.

## Conclusion

We demonstrated that using a linker with specific properties to connect the two subunits of the heterodimeric [FeFe]-hydrogenase *Dd*HydAB significantly enhances the rate of *in vitro* maturation, while retaining the important catalytic features of this enzyme, such as high activity and affinity for the protecting inhibitor sulfide. Our data provide important insights into the mechanism and rate-limiting factors of cofactor maturation. Our results support the hypothesis of a maturation mechanism where 2Fe^MIM^_H_ must first be tightly bound and stabilized in the binding pocket by reconfigurations in the plug-lock-lid system before subcluster coupling can occur. Consequently, efficient *in vitro* maturation depends on the right degree of flexibility of the structural elements involved. This flexibility must allow for a fully open conformation to enable efficient and fast 2Fe_H_ uptake *via* the maturation channel to the 4Fe_H_-moiety of the H-cluster. At the same time, the structural elements still need to be rigid enough to sufficiently support the conformational changes required to close and lock up the maturation channel before forcing the 2Fe_H_-subcluster into its rotated, catalytically preferred conformation and enabling sub-cluster coupling. On the path toward establishing *Dd*HydAB as a competitive and fully regenerative biocatalyst in future applications, subunit-fusion allowed us to overcome the hurdle of the extremely slow catalytic activation of the heterodimeric wild-type enzyme.

## Author contributions

M. W., J. J., N. P., I. S. and C. L. conceived and planned the experiments. J. J., M. W., J. B., M. M. and K. B. carried out the experiments. U. P. A. and S. Y. provided the cofactor for the *in vitro* maturation experiments. M. W., J. J., N. P., I. S., K. B. and J. A. B. contributed to the interpretation of the results. M. W., J. J. and N. P. took the lead in writing the manuscript. All authors provided critical feedback and helped shape the research, analysis and manuscript.

## Conflicts of interest

There are no conflicts to declare.

## Supplementary Material

SC-OLF-D5SC07299A-s001

SC-OLF-D5SC07299A-s002

SC-OLF-D5SC07299A-s003

## Data Availability

The data supporting this article have been included as part of the supplementary information (SI). Supplementary information: File S1: materials and methods, text, Fig. S1–S12, and Tables S1–S4. A separate file (deposition reports) comprises all deposition reports associated with the newly published X-ray structure data summarized in Table S3. File S2: genomic data, polypeptide sequences, and further information on the origin and phylogenetic distribution of heterodimeric M2-type [FeFe]-hydrogenases. It also provides a hypothesis for the evolutionary pressures that may have led to the unusual heterodimeric configuration of periplasmic M2-type [FeFe]-hydrogenases and may help explain the low *in vitro* maturation rate of this M2 subtype. See DOI: https://doi.org/10.1039/d5sc07299a.
