# Supplementary material for: Subunit fusion unlocks rapid in vitro maturation for slowly activating heterodimeric [FeFe]-hydrogenases
Source: Chem Sci. 2026 Feb 3;17(15):7678–89. doi: 10.1039/d5sc07299a (PMC12933642; doi:10.1039/d5sc07299a)
Supplement: SC-017-D5SC07299A-s001 [file SC-017-D5SC07299A-s001.pdf]

Supplementary Information (SI) for Chemical Science.
This journal is © The Royal Society of Chemistry 2026

Supplementary Information 1

Subunit fusion unlocks rapid *in vitro* maturation for slowly activating heterodimeric [FeFe]-hydrogenases

Jan Jaenecke, Konstantin Bikbaev, Miriam Malagnini, Julia Bronold, Shanika Yadav, Ulf-Peter Apfel, Christophe Léger, James A. Birrell, Ingrid Span, Nicolas Plumeré*, Martin Winkler*

J. Jaenecke, J. Bronold, Prof. Dr. N. Plumeré, Dr. M. Winkler
Professorship for Electrobiotechnology, Technical University of Munich, Campus Straubing for Biotechnology and Sustainability, Uferstrasse 53, 94315 Straubing, Germany.
E-mail: martin-h.winkler@tum.de, nicolas.plumere@tum.de

Dr. M. Winkler
Inorganic Spectroscopy, Energy Converting Enzymes, Max-Planck-Institute for Chemical Energy Conversion, Stiftstrasse 34-36, 45470 Mühlheim an der Ruhr, Germany

Dr. K. Bikbaev, Prof. Dr. I. Span
Bioinorganic Chemistry, Friedrich-Alexander-Universität Erlangen-Nürnberg, Egerlandstrasse 1, 91058 Erlangen, Germany

Dr. S. Yadav, Prof. Dr. U.-P. Apfel
Activation of Small Molecules/Technical Electrochemistry, Faculty of Chemistry and Biochemistry, Ruhr-University Bochum, Universitätsstrasse 150, 44801 Bochum, Germany

Prof. Dr. U.-P. Apfel
Department of Electrosynthesis, Fraunhofer UMSICHT, 46047 Oberhausen, Germany

Dr. J. A. Birrell
School of Life Sciences, University of Essex, Wivenhoe Park, Colchester, CO4 3SQ, UK

Dr C. Léger, Ms M. Malagnini
Laboratoire de Bioénergétique et Ingénierie des Protéines (BIP), CNRS, Aix-Marseille Université, 31 chemin Joseph Aiguier, 13009 Marseille, France

This PDF file includes:

- Material and Methods
- Supporting Text
- Supporting Figures S1 to S12
- Supporting Tables S1 to S4

Materials and Methods

Plasmid preparation

The genes for the large (LSU) and small subunit (SSU) were amplified using the pACYC-Duet-1-*Dd*HydAB expression plasmid previously published by Birrell and coauthors [1] following a Fusion-PCR approach with primers 1-16. The corresponding linker sequences are inserted between both genes using primers A and B (see Tab. S3) in a Fusion-PCR step, retaining the C-terminal Strep-tag II on the large subunit. The genes for the monomeric *Dd*HydAB fusion proteins were integrated into the MCS of expression vector pET21b. The sequences of the resulting expression constructs were verified via Sanger sequencing.

Enzyme preparation

*Dd*HydAB, the *Dd*HydAB fusion variants L1-L4, *Dd*HydF, *Cr*HydA1 and *Cp*I were recombinantly expressed in *Escherichia coli* BL21 (DE3) *ΔiscR* [2] and isolated as apo-hydrogenases, lacking the catalytically essential $2Fe_H$-subcluster of the H-cluster, as described before by Kuchenreuther and coauthors. [3] After reaching the desired OD_{600}, all steps were conducted in an anaerobic chamber (Coy Laboratories), under a mixed atmosphere of 97.5% N_2 and 2.5% H_2. All solutions for the cultivation were degassed. Target proteins were purified via column chromatography exploiting the selective affinity of the C-terminal Strep-tag II to the Strep-Tactin® Superflow resin (IBA Lifesciences) following manufacturer's instructions. The purity of isolated proteins was evaluated by applying SDS-PAGE. Samples were stored at -80 °C under anaerobic conditions in 0.1 M Tris-HCl buffer, pH 8.0.

Determination of concentration and iron-sulfur cluster-occupancy of the protein isolates

The concentrations of isolates were determined using electronic absorption spectroscopy. According to earlier work [1], the absorbance values at 280 nm and 400 nm were used to determine the protein concentration and [FeS]-content of all samples. Protein concentrations were determined via a standard Bradford assay, using BSA for calibration.

In vitro *maturation*

$2Fe_H^{MIM}$ ($Fe_2[\mu\text{-}(SCH_2)_2NH](CN)_2(CO)_4[Et_4N]_2$) was synthesized as described earlier. [4] The crystalline $2Fe_H^{MIM}$-complex was dissolved in 0.1 M potassium phosphate buffer (pH 6.8). All steps were performed in an anaerobic chamber (Coy Laboratories) [5], using an atmosphere of approximately 97.5% N_2 and 2.5% H_2. For the activation of the apo-hydrogenase, the enzyme was diluted to 160 µM in 0.1 M Tris-HCl (pH 8.0) and incubated in the dark with a 3-fold molar excess of $2Fe_H^{MIM}$ for 50 h at 35 °C. The $2Fe_H^{MIM}$ and holo-proteins were kept under dim light to prevent a light-induced decomposition of the cofactor mimic. After activation, the excess $2Fe_H^{MIM}$ was separated from the activated protein by spin filtration (30 kDa MWCO) and further incubated in the dark for 35 h at 10 °C under a 2.5% H_2 atmosphere. For the protein crystallization samples, the buffer was exchanged to 0.01 M Tris-HCl, pH 7.6. *Dd*HydF was loaded with $2Fe_H^{MIM}$ by mixing a 160 µM enzyme solution with a 3-fold excess of $2Fe_H^{MIM}$ in 0.1 M Tris-HCl buffer (pH 8.0) and incubation on ice for 2 h. After the incubation, excess $2Fe_H^{MIM}$ was removed by using a 30 kDa MWCO spin filter. Holo-protein samples were aliquoted and stored under anaerobic conditions at -80 °C.

In vitro *maturation kinetics*

For tracking the *in vitro* maturation, a modified version of the hydrogen evolution assay was used as described earlier. [3, 5, 6] All steps were performed in an anaerobic chamber (Coy Laboratories), in an atmosphere of approximately 97.5% N_2 and 2.5% H_2. The apo-protein with a final concentration

of 160 µM was mixed with a 3-fold molar excess of $2Fe_H$-mimic complex in 0.1 M Tris-HCl buffer (pH 8.0) and incubated at 35 °C and 400 rpm. At the given time points, samples were prepared with a final volume of 2 mL containing 300 ng of the respective enzyme sample. The reaction mixture further included 0.1 M Tris-HCl, pH 8.0, 200 mM sodium dithionite and 10 mM methyl viologen dichloride. Samples were sealed in 8 mL headspace-vessels and purged with 100% argon for 5 min prior to 20 min incubation at 37 °C in a shaking water bath (100 rpm). To quantify the H_2 amount produced during the 20 min incubation period, 400 µL of the headspace were analyzed via gas chromatography (Shimadzu, 2010Pro). To monitor the kinetics of *Dd*HydF-dependent *in vitro* maturation, a 3-fold excess of $2Fe_H^{MIM}$-loaded *Dd*HydF was used instead of free $2Fe_H^{MIM}$ complex.

FTIR spectroscopy

All FTIR-spectra were collected using a Bruker Vertex 80. For the measurement, 20 µL of 0.4 mM enzyme dilution in 0.1 M Tris-HCl buffer (pH 8.0) with a 3-fold excess of $2Fe_H^{MIM}$ complex were placed between two CaF_2 windows and sealed in a transmission FTIR-cell under anaerobic conditions. Spectra were recorded at room temperature every 15 min against pure 0.1 M Tris-HCl as blank using OPUS 7. As soon as the progression of the peak increase at 2016 cm^{-1} was saturated, the measurement was stopped.

Protein film electrochemistry (PFE)

a) Kinetics of CO-inhibition (see Tab. S1)

The WT enzyme and all variants were characterized using the same procedures to measure the Michaelis constants for H_2 and the rate constants of binding and release of CO. In all cases protein films were produced by drop-casting a small (0.2 µL) amount of protein solution (about 100 nM) onto a freshly polished pyrolytic graphite edge electrode (diameter: 3 mm), letting it dry for about 20 seconds. The electrode was inserted in the electrochemical cell, filled with pH 7 buffer (chloride-free mixed buffer consisting of 5 mM of each of MES, CHES, HEPES, TAPS, Na-acetate, Na_2SO_4 100 mM, titrated NaOH 1 M), and rotated at a high rate (3000 rpm) to prevent H_2 depletion near the electrode surface. The temperature of the electrochemical cell was set to 20 °C.
To measure the Michaelis constants, the H_2 oxidation current was measured at -160 mV vs SHE while the cell solution, initially saturated with H_2, was flushed with argon (Fig. S7). The Michaelis-Menten constant was obtained by fitting eq. 2 published in our previous study [7] to the current-time trace. The value of K_M was converted from atm of H_2 to mM using a solubility of 0.80 mM/atm of H_2 at 20 °C.[8, 9]
To measure the rate constants for the binding and release of CO, the H_2-oxidation current was monitored at -260 mV and -160 mV vs SHE (this made no difference). A small amount (typically 100 nM) of CO was added to the electrochemical cell solution, which resulted in a decrease in the H_2-oxidation current. The cell was constantly bubbled with H_2, which makes the concentration of CO decrease (exponentially) as a function of time. A previously published simple two-step kinetic model [10, 11] was fitted to the data using the program Qsoas (qsoas.org) [12], as illustrated in Fig. S8, to measure the bimolecular binding rate constant and the 1st order CO release rate constant. The former was corrected to take into account the protection by H_2 using the classical equation $K_i = k_i^{app} \times (1+[H_2]/K_M)$.[10, 13]

b) Impact of sulfide inhibition on irreversible O_2 induced enzyme inactivation (Fig. 5)

To compare the protective effect of sulfide addition on irreversible O_2 induced enzyme inactivation

PFE measurements were performed in an anaerobic chamber (Coy Laboratories) under anaerobic conditions using an Autolab PGSTAT204 potentiostat, controlled by NOVA 2.1.5. An electrochemical cell with a water jacket was used to control the temperature by a circulating water bath during the measurement. The homemade pyrolytic graphite edge electrode (PEG, A=9 mm^2)

was rotated at 2000 rpm using an Autolab rotator unit. The potential was controlled via an Ag/AgCl reference electrode (Ag/AgCl; 3 M KCl) and a platinum wire was used as a counter electrode. When processing the data, the reference electrode's potential was converted to the standard hydrogen potential (SHE) following the equation: $E_{SHE} = E_{ref} + 0.216$ V at 15 °C.

After filling the electrochemical cell with 7 mL of 0.1 M potassium phosphate buffer with 0.1 M NaCl as supporting electrolyte (pH 7), the gas mixture in the electrochemical cell was controlled by using mass flow controllers (MKS Instruments Inc.; Model GE50A). To saturate the buffer in the electrochemical cell, the buffer was purged with H_2 (5.0; Westfalen) for 30 min prior to every measurement. The PEG electrode was polished inside the anaerobic chamber with a polishing grid and thoroughly cleaned with distilled water to remove graphite dust particles. To ensure that the electrode was clean, a blank measurement under 100% H_2 atmosphere was performed (CV; 20 $mV \cdot s^{-1}$). For the measurement itself, the electrode was cleaned again with distilled water to remove buffer salts from the surface. Then the dry electrode was drop-cast with 3 µL of a 10 µM enzyme solution and incubated for 3 min at room temperature. To remove unattached enzyme, the electrode was again thoroughly rinsed with distilled water.

Chronoamperometric data were recorded starting at -0.5 V vs. SHE. After 150 s, the potential was rapidly shifted to 0.1 V vs. SHE. The sulfide inhibition step was initiated by adding Na_2S to the buffer in the electrochemical cell to a final concentration of 1 mM 50 s after the potential shift. For the negative control, no Na_2S was added. 120 s after the Na_2S addition, O_2-saturated buffer was injected into the buffer to a final concentration of 50 µM O_2. To remove trace amounts of O_2 from the buffer, the buffer was exchanged 3-fold 120 s after adding the O_2-saturated buffer. After a total of 540 s, the potential of the working electrode was shifted back to -0.5 V vs. SHE.

Protein crystallography

All crystallization experiments were carried out inside an anaerobic vinyl chamber maintained at ambient temperature (approximately 20 °C) under an atmosphere of 2% H_2 and 98% N_2. Crystallization of all protein variants was performed using the vapor-diffusion sitting-drop method in MRC 96-Well 2-Drop crystallization plates. The proteins were prepared in 0.01 M Tris-HCl buffer (pH 7.6). Drops of 2 µL, consisting of protein solution and precipitant in a 1:1 ratio, were placed over a 50 µL reservoir. Crystals of variant L1 were obtained by mixing protein solution at 17.5 mg/mL with reservoir solution containing 0.1 M sodium acetate, 0.2 M LiCl, and 25% w/v PEG 4000. Crystals of variant L2 were obtained by mixing protein solution at 20 mg/mL with reservoir solution containing 100 mM HEPES, pH 7.5, and 20% w/v PEG 1500. Crystals of variant L3 were obtained by mixing protein solution at 32 mg/mL with reservoir solution containing 0.1 M HEPES, pH 7.6, and 50% v/v MPD. Crystals of variant L4 were obtained by mixing protein solution at 15 mg/mL with reservoir solution containing 0.1 M sodium acetate, 1 M LiCl, and 25% w/v PEG 4000. Crystals of apo-*Dd*HydAB were obtained by mixing protein solution at 20 mg/mL with reservoir solution containing 0.1 M sodium acetate, 1 M LiCl, and 27.5% w/v PEG 4000. Crystals of holo-*Dd*HydAB were obtained by mixing protein solution at 20 mg/mL with reservoir solution containing 0.1 M sodium acetate, 1 M LiCl, and 30% w/v PEG 4000. Brown crystals were observed within one week, harvested within 1-2 weeks, soaked in cryo-protectant, and stored in liquid nitrogen.

All datasets were collected at the Deutsches Elektronen-Synchrotron (DESY, Hamburg, Germany[14, 15]) on beamline P11 using an Eiger 2-16M CdTe detector. Data were recorded at 100 K at energies of 12 keV (L3, L4), 15 keV (L2), 16 keV (L1), or 18 keV (apo- and holo-*Dd*HydAB). Data processing was carried out using XDS [16], and data reduction was performed with AIMLESS [17]. Phases were obtained by molecular replacement using MOLREP[18] for L3 or PHASER [19] for L1, L2, L4, apo-, and holo-*Dd*HydAB, with the coordinates of *Dd*HydAB H_{inact} (PDB ID: 6SG2) as a starting model. Automated model building was performed with BUCCANEER[20] for L1, L3, and L4. Iterative cycles of refinement and manual model building were carried out using REFMAC [21]and Coot [22]. Validation and preparation for deposition were performed using CCP4i [23]. The final coordinates and structure factors have been deposited in the Research Collaboratory for Structural

Bioinformatics Protein Data Bank (RCSB PDB) under accession codes 8RU6 (L1), 9GNK (L2), 9GBU (L3), 8RYH (L4), 8RTG (apo-*Dd*HydAB), and 9QD6 (holo-*Dd*HydAB).

Supporting Text

Supplementary discussion

As documented by the American Type Culture Collection (ATCC), the strain ATCC 7757 was formerly classified as *Desulfovibrio desulfuricans subsp. desulfuricans*.* [24, 25] The reassignment of ATCC 7757 to *Desulfovibrio vulgaris* aligns with advancements in bacterial taxonomy and phylogenetic analysis. Additional references, such as LGC Standards, confirm this by declaring ATCC 7757 as *Desulfovibrio vulgaris* while acknowledging its original designation as *Desulfovibrio desulfuricans*.** *Desulfovibrio vulgaris* is also the basionym of the species *Nitratidesulfovibrio vulgaris.*[26] The latter declaration emphasizes its metabolic versatility, particularly in reducing nitrate alongside sulfate under anaerobic conditions, a characteristic further explored in recent studies.

* ATCC (American Type Culture Collection): Desulfovibrio vulgaris ATCC 7757; https://www.atcc.org/products/7757

** LGC Standards*:* Desulfovibrio vulgaris (ATCC 7757); https://www.lgcstandards.com/DE/de/Desulfovibrio-vulgaris/p/ATCC-7757

Supporting figures

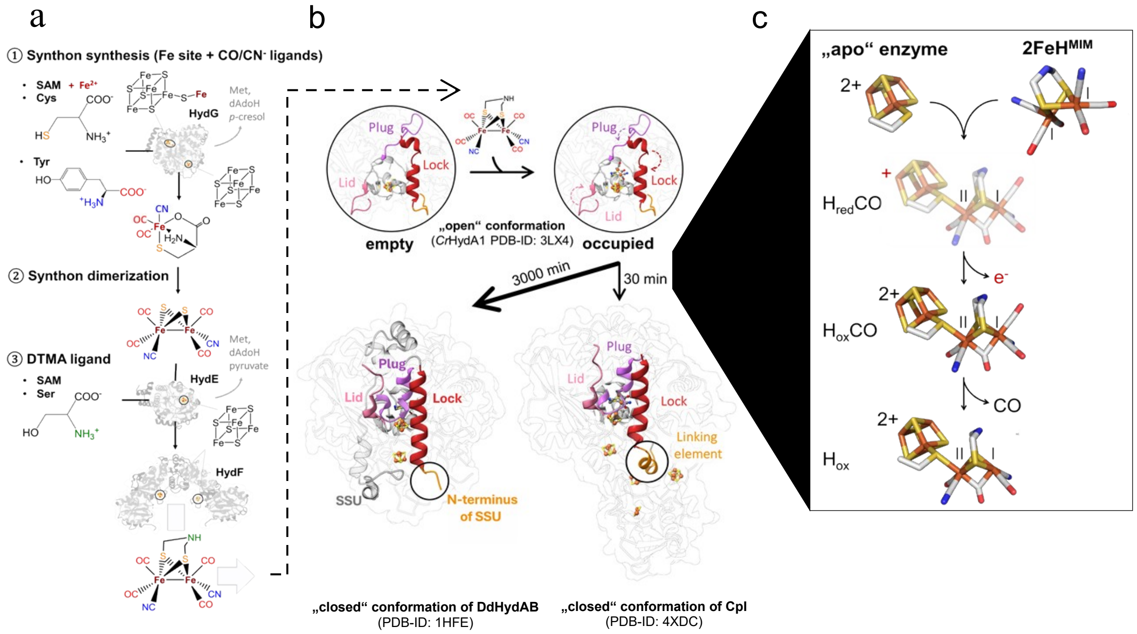

Figure S1| Graphical summary of *in vivo* and *in vitro* maturation. **(a)** *In vivo* the $2Fe_H$ precursor is synthesized by the three maturases HydE, HydG and HydF. The turnover of two tyrosine molecules at a dangler iron that is attached to the [4Fe4S]-cluster of radical-SAM protein HydG yields a single CN^- and two CO ligands. Together with a cysteine molecule, these constituents are combined to synthon complex B $[Fe^{II}(CO)_2(CN)(Cys)]^-$. Two of these mononuclear low-spin iron complexes are modified and finally combined by HydE, a second radical SAM protein, to produce the dinuclear precomplex $[Fe^I_2(\mu\text{-SH})_2(CO)_4(CN)_2]^{2-}$. In the final step, the two iron sites of the chemically binuclear complex are cross-bridged by a dithiomethylamine (DTMA) ligand contributed by the serine-hydroxymethyltransferase (SHMT) and the aminomethyltransferase (T-protein) of the glycine cleavage system. Alternatively, the $2Fe_H$ precursor can be chemically synthesized as a mimic and used for *in vitro* maturation. Upon insertion of the $2Fe_H$ precursor complex into the H-cluster binding pocket, the open conformation of the protein is closed and locked by the “plug-lock-lid” mechanism. **(b)** A comparison of the closed-state structures of *Cp*I (4XDC) and *Dd*HydAB (1HFE) illustrates that the gap between SSU and LSU is right at the N-terminus of the lock-element and could thus have an impact on the rate of binding site closure after cofactor insertion. In the monomeric *Cp*I, the lock helix is stabilized by a linking element. **(c)** Process of H-cluster formation and activation comprising $2Fe_H$ and $4Fe_H$-subcluster coupling via a Cys ligand, adopting a bridging configuration (μCys) which generates the transient $H_{red}CO$ state. An oxidation step leads to the more stable $H_{ox}CO$ state before the loss of the apical CO-ligand yields the oxidized active-ready state H_{ox}.

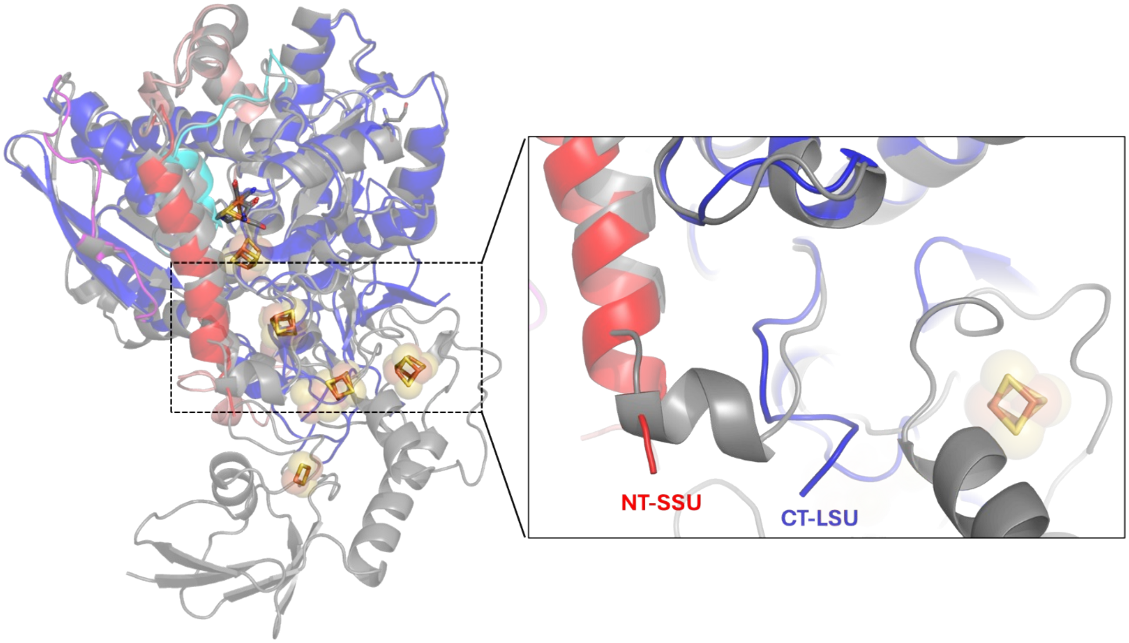

Figure S2| Superposition of X-ray structures of the heterodimeric *Dd*HydAB^{WT} (PDB ID: 9QD6; M2-type; LSU in blue; SSU in light red; Plug in cyan; Lid in magenta and Lock in red) and the monomeric *Cp*I (PDB code: 4XDC; M3-type; gray). A zoomed view shows the C-terminus (CT) of the LSU and the N-terminus (NT) of the SSU in *Dd*HydAB^{WT}. A gray helix element cross-bridges both sections in the monomeric *Cp*I enzyme.

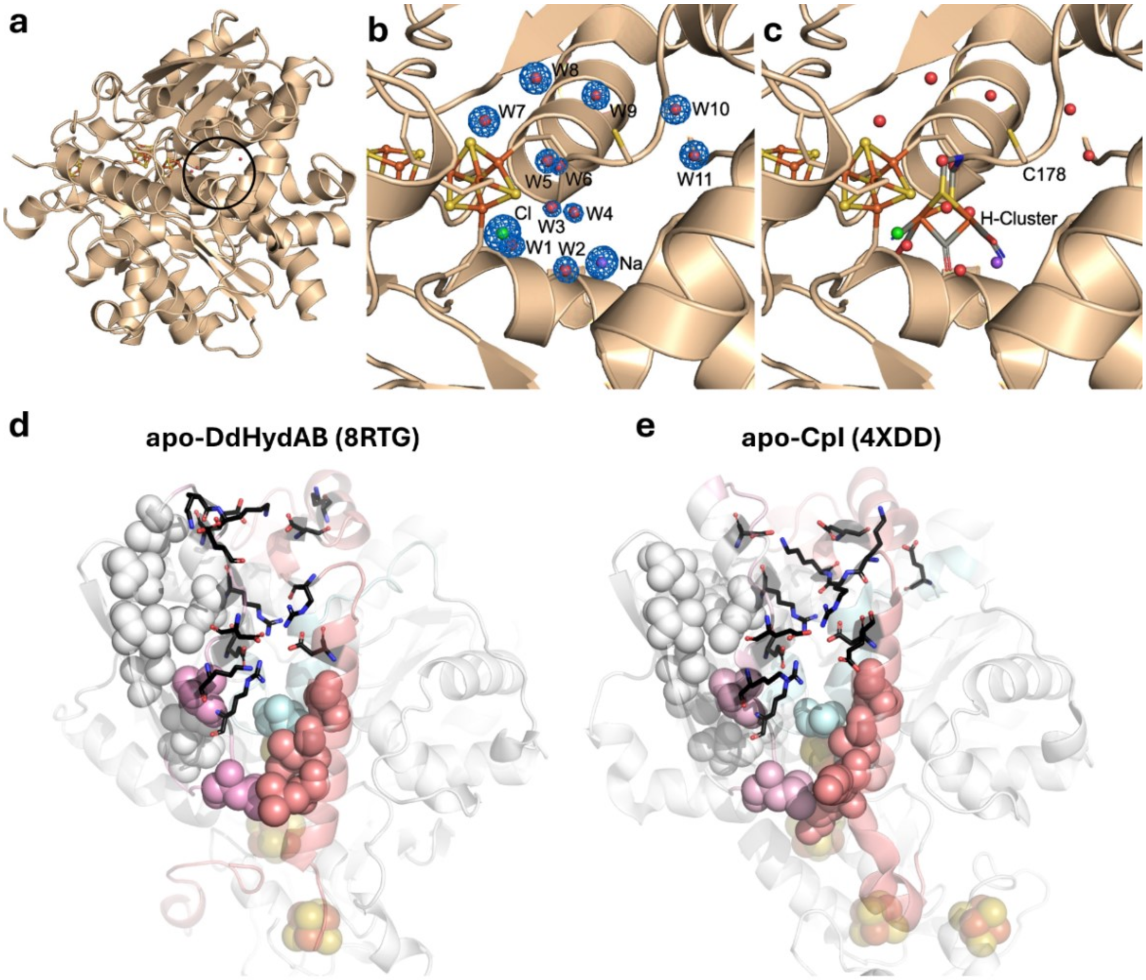

***Dd*HydAB-LSU(in holoprotein)**

MSRTVMERIEYEMHTPDPKADPDKLHFVQIDEAKCIGCDTCSQYCPTAAIFGEMGEPHSIPHIEACINCGQCLTHCPEN
AIYEAQSWVPEVEKKLKDGKVKCIAMPAPAVRYALGDAFGMPVGSVTTGKMLAALQKLGFAHCWDTEFTADVTIWEEGS
EFVERLTKKSDMPLPQFTSCCPGWQKYAETYYPELLPHFSTCKSPIGMNGALAKTYGAERMKYDPKQVYTVSIMPCIAK
KYEGLRPELKSSGMRDIDATLTTRELAYMIKKAGIDFAKLPDGKRDSLMGESTGGATIFGVTGGVMEAALRFAYEAVTG
KKPDSWDFKAVRGLDGIKEATVNVGGTDVKVAVVHGAKRFKQVCDDVKAGKSPYHFIEYMACPGGCVCGGGQPVMPGVL
EA

***Dd*HydAB-SSU (in holoprotein)**

AVKQIKDYMLDRINGVYGADAKFPVRASQDNTQVKALYKSYLEKPLGHKSHDLLHTHWFDKSKGVKELTTAGKLPNPRA
SEFEGPYPYE

***Cp*I**

MKTIIINGVQFNTDEDTTILKFARDNNIDISALCFLNNCNNDINKCEICTVEVEGTGLVTACDTLIEDGMIINTNSDAV
NEKIKSRISQLLDIHEFKCGPCNRRENCEFLKLVIKYKARASKPFLPKDKTEYVDERSKSLTVDRTKCLLCGRCVNACG
KNTETYAMKFLNKNGKTIIGAEDEKCFDDTNCLLCGQCIIACPVAALSEKSHMDRVKNALNAPEKHVIVAMAPSVRASI
GELFNMGFGVDVTGKIYTALRQLGFDKIFDINFGADMTIMEEATELVQRIENNGPFPMFTSCCPGWVRQAENYYPELLN
NLSSAKSPQQIFGTASKTYYPSISGLDPKNVFTVTVMPCTSKKFEADRPQMEKDGLRDIDAVITTRELAKMIKDAKIPF
AKLEDSEADPAMGEYSGAGAIFGATGGVMEAALRSAKDFAENAELEDIEYKQVRGLNGIKEAEVEINNNKYNVAVINGA
SNLFKFMKSGMINEKQYHFIEVMACHGGCVNGGGQPHVNPKDLEKVDIKKVRASVLYNQDEHLSKRKSHENTALVKMYQ
NYFGKPGEGRAHEILHFKYKKSA

Fig. S3| Annotated X-ray structures depicting the apo-forms of *Dd*HydAB (PDB ID: 8RTG) and *Cp*I (PDB ID: 4XDD) both crystallized in the 'premature' closed-state configuration. (a) Overall structure of apo-*Dd*HydAB. **(b)** Zoomed view on the closed but cofactor-free $2Fe_H$ binding

site. **(c)** Superposition with the $2Fe_H$ cofactor of the holo-enzyme, revealing that water molecules at locations W1-W6 will be replaced with structure elements of the $2Fe_H$-subcluster such as the CO-ligands and the adt-bridge while Na^+ and Cl^- ions occupy the positions of the CN^- ligands. **(d, e)** Residues involved in salt bridge contacts (black sticks) and hydrophobic interactions (colored spheres) that stabilize the reconfigured plug (cyan), lock (red) and lid (magenta) elements. Contact sites not originating from any of the three elements are shown as white spheres. Corresponding positions are likewise marked below in the polypeptide sequences of *Cp*I, as well as LSU and SSU from *Dd*HydAB, showing amino acids contributing to salt bridge contacts with a black background and those involved in hydrophobic interactions within and between the reconfigured structure elements are indicated by a correspondingly colored background.

a

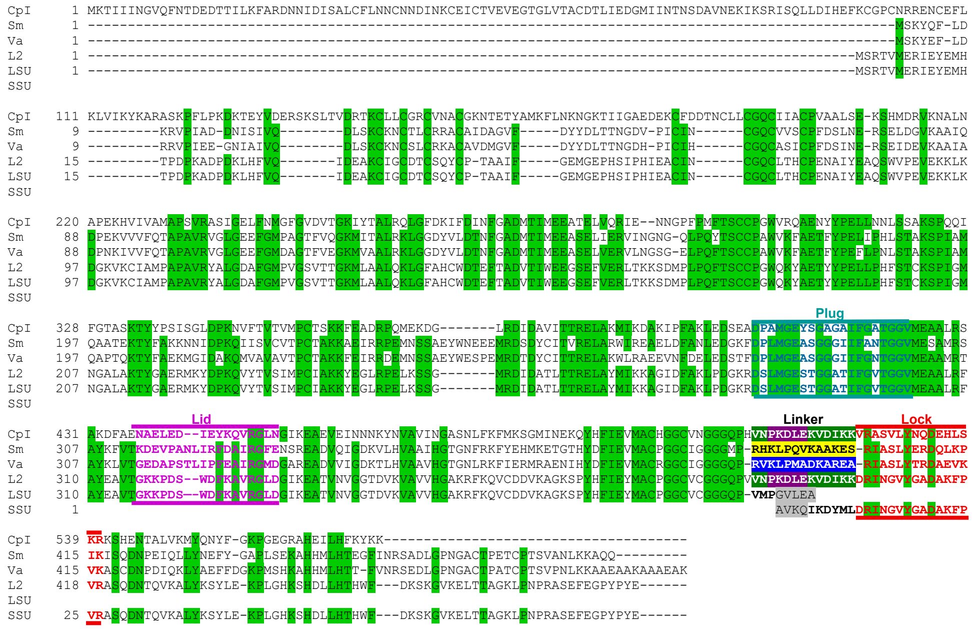

b

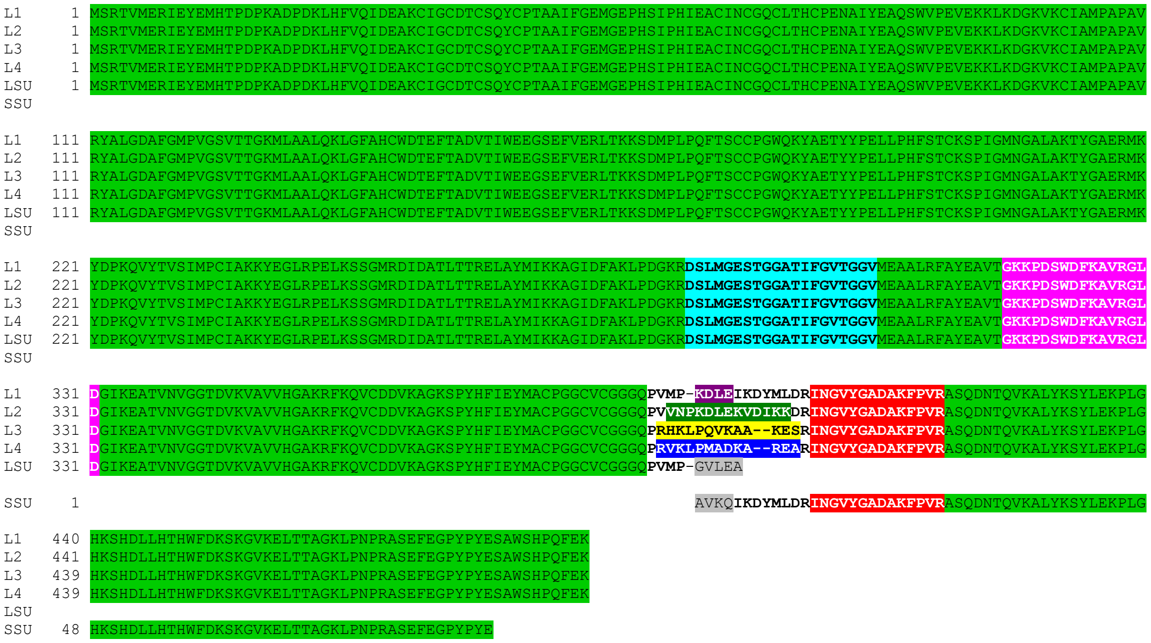

Fig. S4| Multiple sequence alignments between the subunits of *Dd*HydAB and reference sequences for linkers 1-4. (a) MSA with LSU and SSU of *Dd*HydAB^{WT} and the [FeFe]-hydrogenases of *Solobacterium moorei* and *Veillonella atypica* that served as references for the linker elements of monomeric *Dd*HydAB variants L3 and L4 (Clone manager 9; method: FastScan-Max Qual (Cons N)). **(b)** MSA of LSU and SSU with monomeric variants L1-L4. In addition to the individual sections of the corresponding linker regions, sequence parts contributing to the plug-lock-lid mechanism are highlighted in cyan, red and pink.

*Dd*HydAB^{WT}

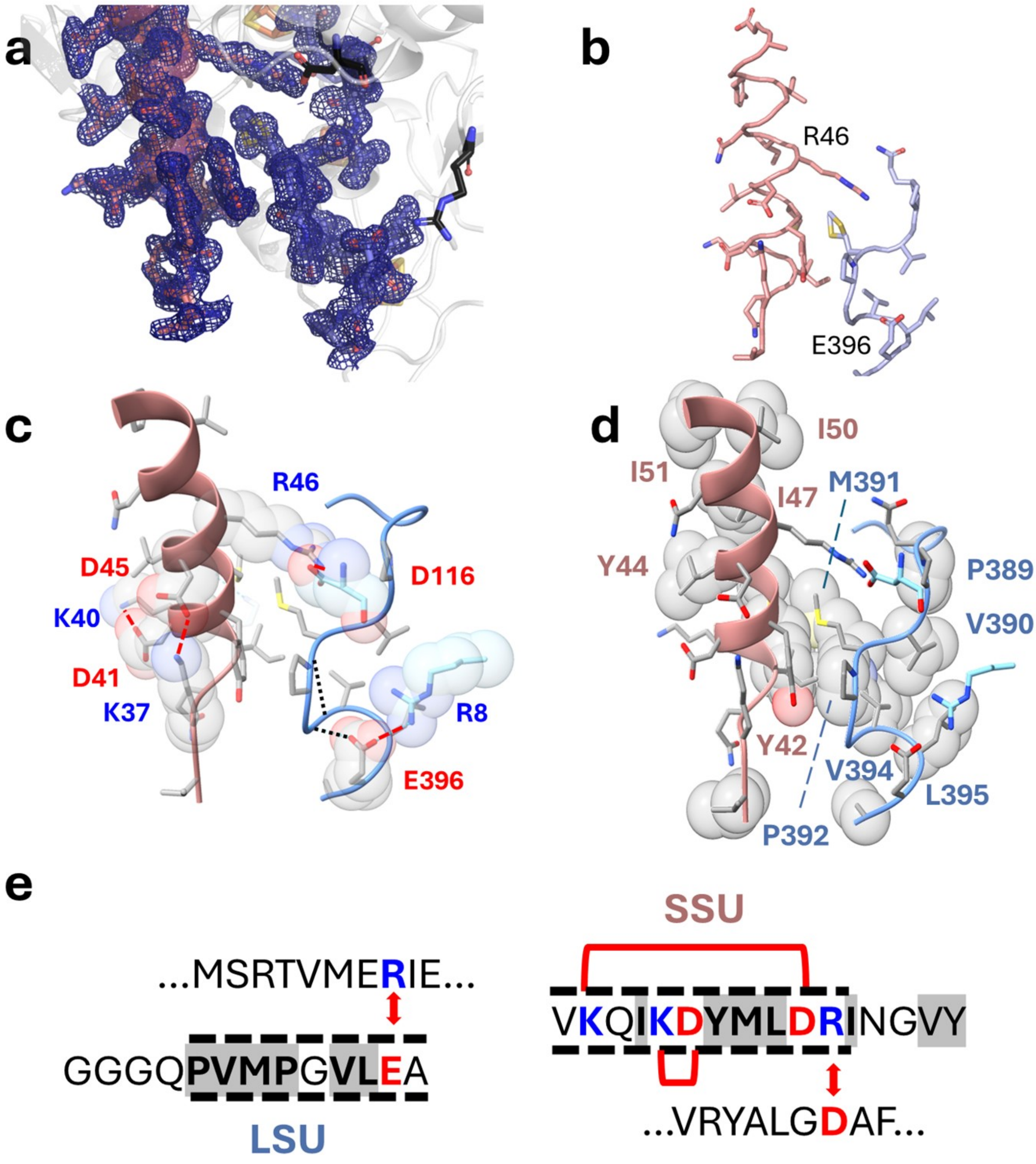

a
b
R46
E396
c
R46
D45
D116
K40
D41
K37
R8
E396
d
I50
I51
I47
M391
Y44
P389
V390
Y42
V394
L395
P392
e
SSU
...MSRTVMERIE...
GGGQPVMPGVLEA
LSU
VKQIKDYMLDRINGVY
...VRYALGDAF...

Variant L1

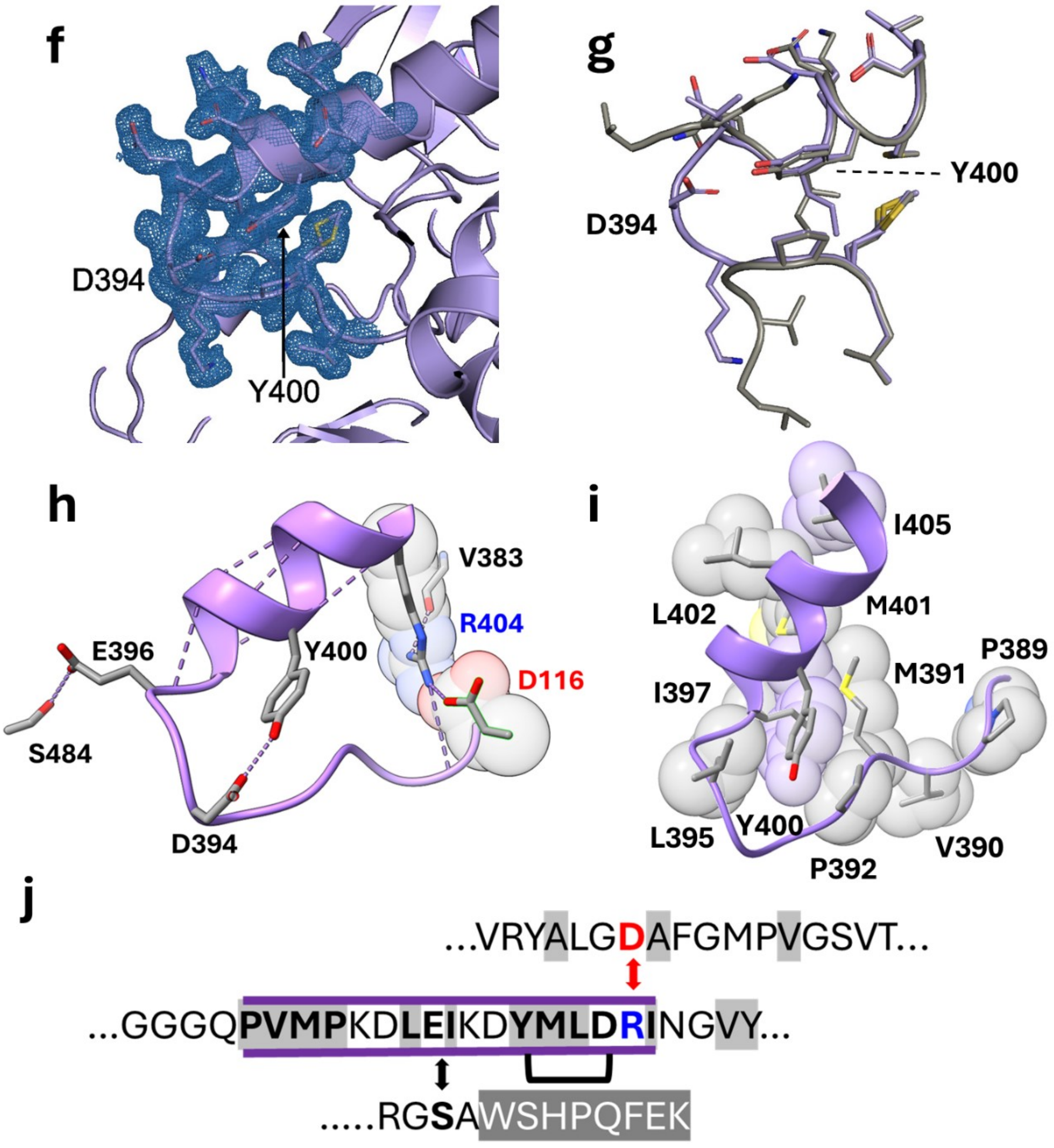

f
D394
Y400
g
Y400
D394
h
V383
R404
D116
E396
Y400
S484
D394
i
I405
M401
L402
M391
P389
I397
L395
Y400
V390
P392
j
...VRYALGDAFGMPVGSVT...
...GGGQPVMPKDLEIKDYMLDRINGVY...
.....RGSAWSHPQFEK

Variant L2

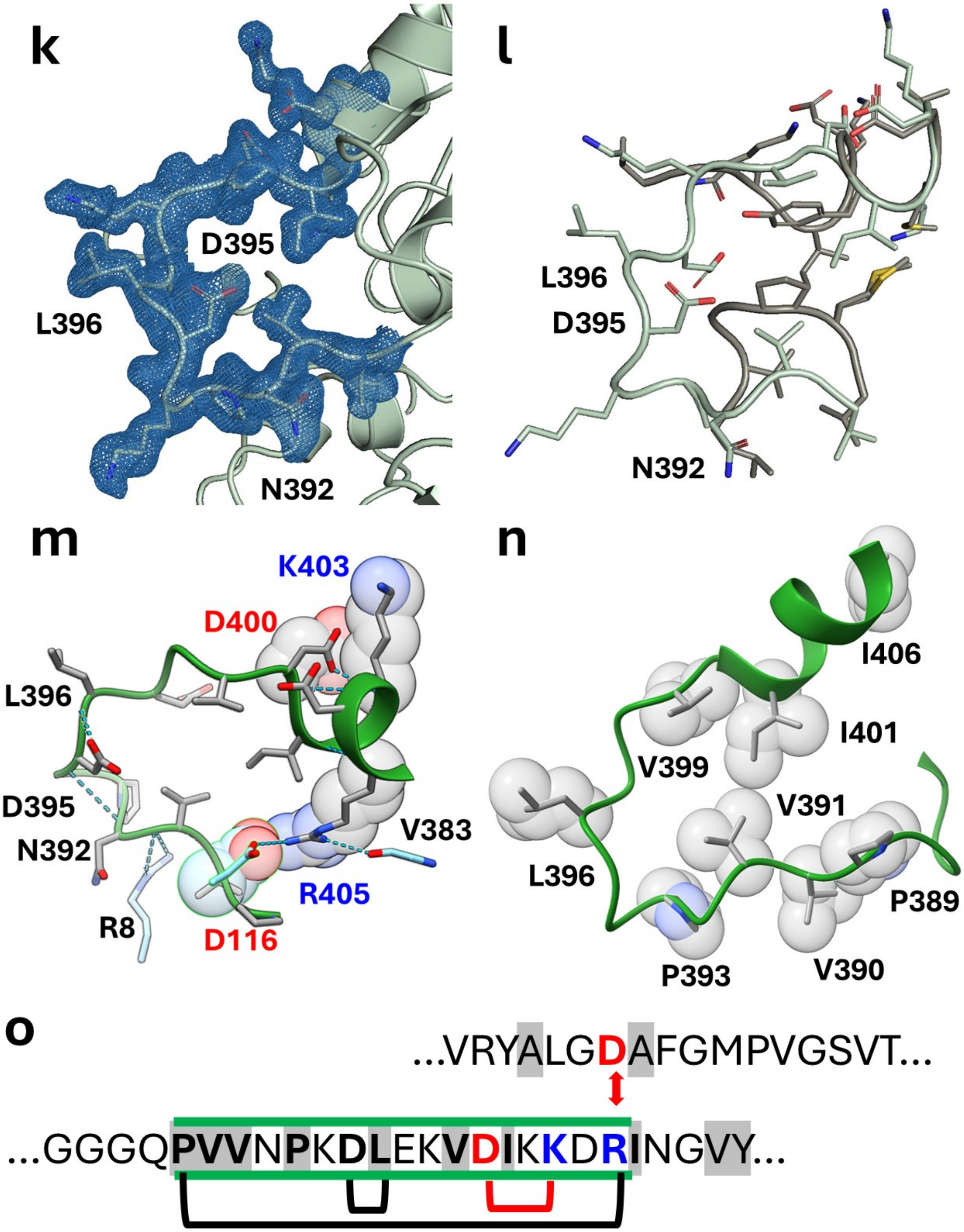

k
D395
L396
N392
l
L396
D395
N392
m
K403
D400
L396
D395
N392
R8
D116
R405
V383
n
I406
I401
V399
V391
L396
P389
P393
V390
o
...VRYALGDAFGMPVGSVT...
...GGGQPVVNPKDLEKVDIKKDRINGVY...

Variant L3

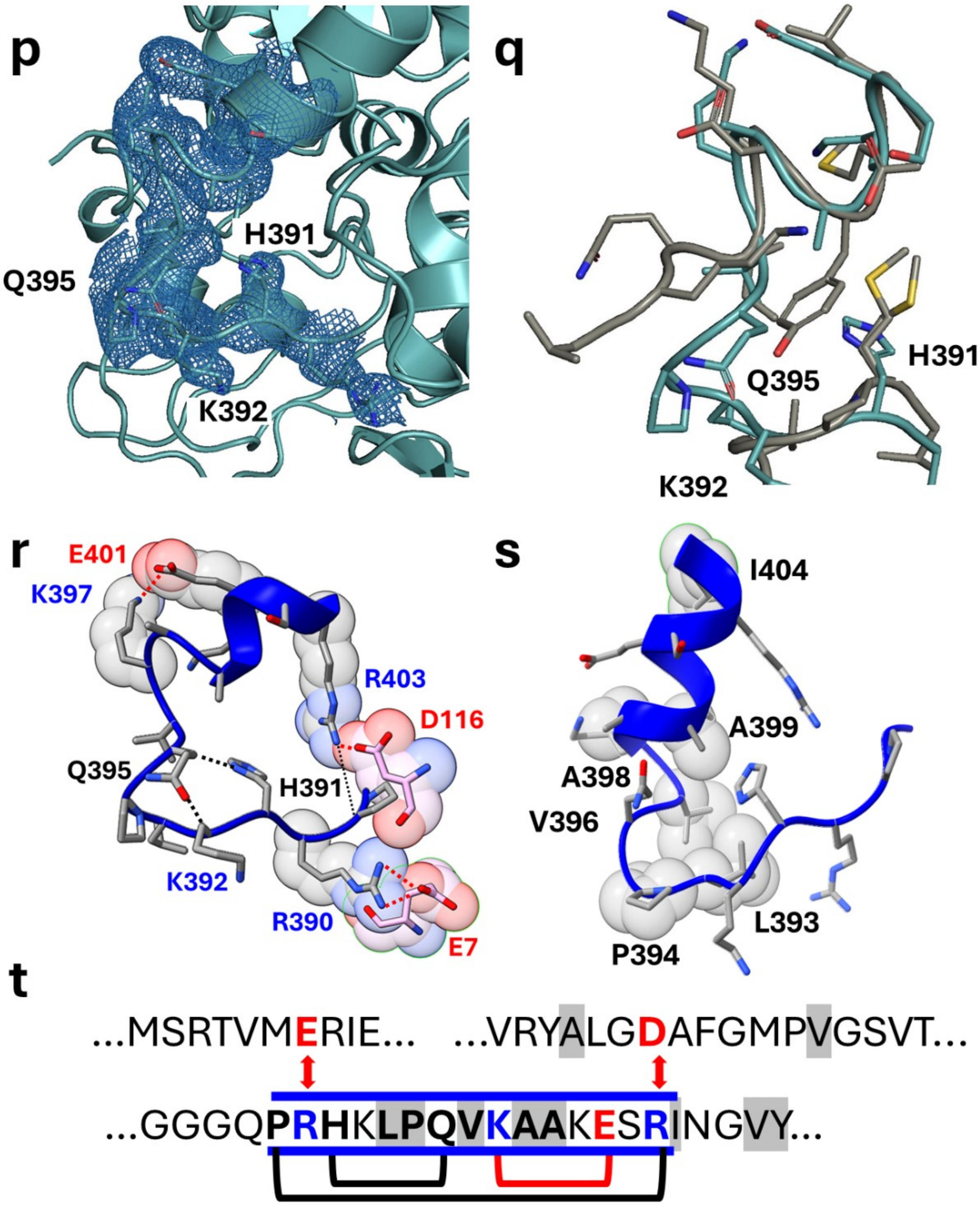
p
H391
Q395
K392
q
H391
Q395
K392
r
E401
K397
R403
D116
Q395
H391
K392
R390
E7
s
I404
A399
A398
V396
L393
P394
t
...MSRTVMERIE...
...VRYALGDAFGMPVGSVT...
...GGGQPRHKLPQVKAAKESRINGVY...

Variant L4

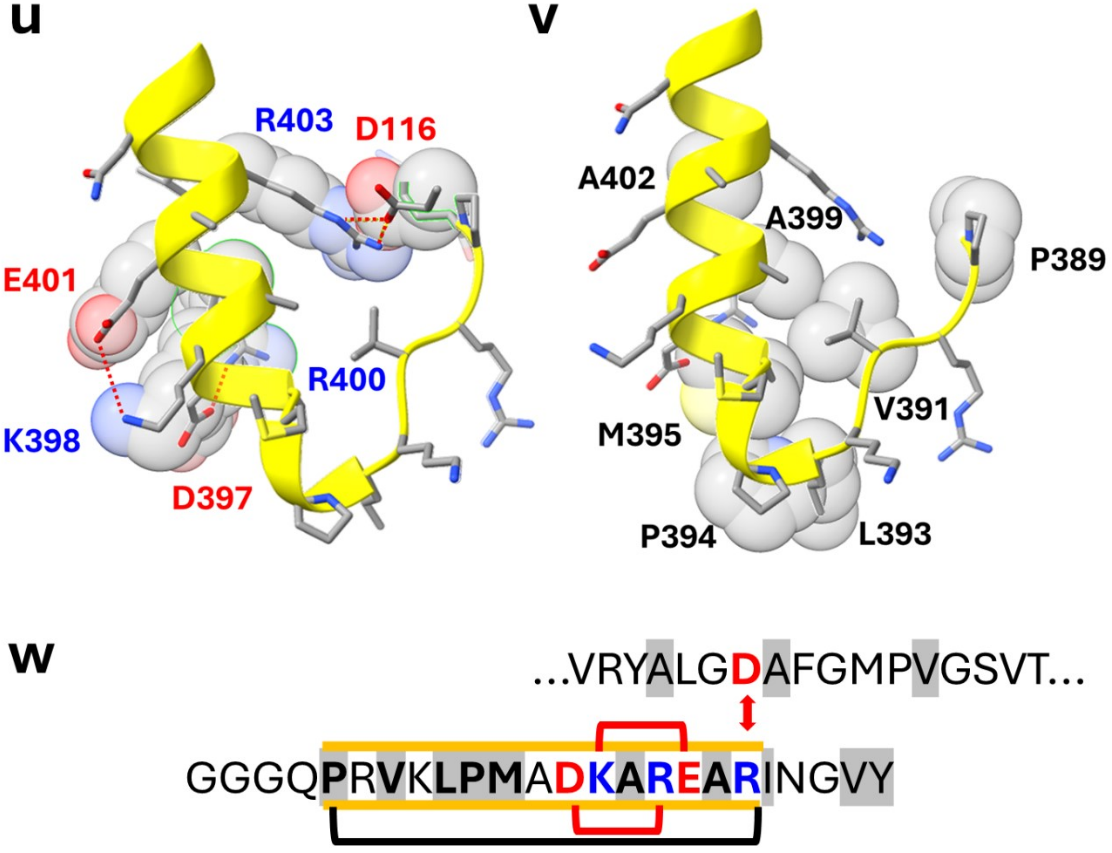

***Cp*I➔Origin of linker in L1, L2**

MKTIIINGVQFNTDEDTTILKFARDNNIDISALCFLNNCNNDINKCEICTVEVEGTGLVTACDTLIED
GMIINTNSDAVNEKIKSRISQLLDIHEFKCGPCNRRENCEFLKLVIKYKARASKPFLPKDKTEYVDE
RSKSLTVDRTKCLLCGRCVNACGKNTETYAMKFLNKNGKTIIGAEDEKCFDDTNCLLCGQCIIACP
VAALSEKSHMDRVKNALNAPEKHVIVAMAPSVRASIGELFNMGFGVDVTGKIYTALRQLGFDKIF
DINFGADMTIMEEATELVQRIENNGPFPMFTSCCPGWVRQAENYYPELLNNLSSAKSPQQIFGTAS
KTYYPSISGLDPKNVFTVTVMPCTSKKFEADRPQMEKDGLRDIDAVITTRELAKMIKDAKIPFAKL
EDSEAD**PAMGEYSGAGAIFGATGGV**MEAALRSAKDFAE**NAELEDIEYKQVRGLNG**IKEAEVEI
NNNKYNVAVINGASNLFKFMKSGMINEKQYHFIEVMACHGGCVNGGGQPHVN**PKDLE**KVDIKK
VR**ASVLYNQDEHLSKR**KSHENTALVKMYQNYFGKPGEGRAHEILHFKYKK

***Dd*HydAB subunit fusion variant L1**

MSRTVMERIEYEMHTPDPKADPDKLHFVQIDEAKCIGCDTCSQYCPTAAIFGEMGEPHSIPHIEACI
NCGQCLTHCPENAIYEAQSWVPEVEKKLKDGKVKCIAMPAPAVRYALGDAFGMPVGSVTTGKM
LAALQKLGFAHCWDTEFTADVTIWEEGSEFVERLTKKSDMPLPQFTSCCPGWQKYAETYYPELLP
HFSTCKSPIGMNGALAKTYGAERMKYDPKQVYTVSIMPCIAKKYEGLRPELKSSGMRDIDATLTT
RELAYMIKKAGIDFAKLPDGKRDSLMGESTGGATIFGVTGGVMEAALRFAYEAVTGKKPDSWDF
KAVRGLDGIKEATVNVGGTDVKVAVVHGAKRFKQVCDDVKAGKSPYHFIEYMACPGGCVCGGG

QPVMPKDLEIKDYMLDRINGVYGADAKFPVRASQDNTQVKALYKSYLEKPLGHKSHDLLHTHW
FDKSKGVKELTTAGKLPNPRASEFEGPYPYESAWSHPQFEK

DdHydAB subunit fusion variant L2

MSRTVMERIEYEMHTPDPKADPDKLHFVQIDEAKCIGCDTCSQYCPTAAIFGEMGEPHSIPHIEACI
NCGQCLTHCPENAIYEAQSWVPEVEKKLKDGKVKCIAMPAPAVRYALGDAFGMPVGSVTTGKM
LAALQKLGFAHCWDTEFTADVTIWEEGSEFVERLTKKSDMPLPQFTSCCPGWQKYAETYYPELLP
HFSTCKSPIGMNGALAKTYGAERMKYDPKQVYTVSIMPCIAKKYEGLRPELKSSGMRDIDATLTT
RELAYMIKKAGIDFAKLPDGKRDSLMGESTGGATIFGVTGGVMEAALRFAYEAVTGKKPDSWDF
KAVRGLDGIKEATVNVGGTDVKVAVVHGAKRFKQVCDDVKAGKSPYHFIEYMACPGGCVCGGG
QPVVNPKDLEKVDIKKDRINGVYGADAKFPVRASQDNTQVKALYKSYLEKPLGHKSHDLLHTH
WFDKSKGVKELTTAGKLPNPRASEFEGPYPYESAWSHPQFEK

***Solobacterium moorei* (GenBank: RGT55168.1)➔Origin of linker in L3**

MSKYQFLDKRVPIADDNISIVQDLSKCKNCTLCRRACAIDAGVFDYYDLTTNGDVPICINCGQCVV
SCPFDSLNERSELDGVKAAIQDPEKVVVFQTAPAVRVGLGEEFGMPAGTFVQGKMITALRKLGGD
YVLDTNFGADMTIMEEASELIERVINGNGQLPQYTSCCPAWVKFAETFYPELIPHLSTAKSPIAMQ
AATEKTYFAKKNNIDPKQIISVCVTPCTAKKAEIRRPEMNSSAEYWNEEEMRDSDYCITVRELARW
IREAELDFANLEDGKFDPLMGEASGGGIIFANTGGVMESAMRSAYKFVTKDEVPANLIRFDAIRGF
ENSREADVQIGDKVLHVAAIHGTGNFRKFYEHMKETGTHYDFIEVMACPGGCIGGGGMPRHKLP
QVKAAKESRIASLYERDQLKPIKISQDNPEIQLLYNEFYGAPLSEKAHHMLHTEGFINRSADLGPN
GACTPETCPTSVANLKKAQQ

DdHydAB subunit fusion variant L3

MSRTVMERIEYEMHTPDPKADPDKLHFVQIDEAKCIGCDTCSQYCPTAAIFGEMGEPHSIPHIEACI
NCGQCLTHCPENAIYEAQSWVPEVEKKLKDGKVKCIAMPAPAVRYALGDAFGMPVGSVTTGKM
LAALQKLGFAHCWDTEFTADVTIWEEGSEFVERLTKKSDMPLPQFTSCCPGWQKYAETYYPELLP
HFSTCKSPIGMNGALAKTYGAERMKYDPKQVYTVSIMPCIAKKYEGLRPELKSSGMRDIDATLTT
RELAYMIKKAGIDFAKLPDGKRDSLMGESTGGATIFGVTGGVMEAALRFAYEAVTGKKPDSWDF
KAVRGLDGIKEATVNVGGTDVKVAVVHGAKRFKQVCDDVKAGKSPYHFIEYMACPGGCVCGGG
QPRHKLPQVKAAKESRINGVYGADAKFPVRASQDNTQVKALYKSYLEKPLGHKSHDLLHTHWF
DKSKGVKELTTAGKLPNPRASEFEGPYPYESAWSHPQFEK

***Veillonella atypica* 2 ➔L4 (GenBank: ARF99709.1) Origin of linker in L4**

MSKYEFLDRRVPIEEGNIAIVQDLSKCKNCSLCRKACAVDMGVFDYYDLTTNGDHPICIHCGQCAS
ICPFDSINERSEIDEVKAAIADPNKIVVFQTAPAVRVGLGEEFGMDAGTFVEGKMVAALRKLGGDY
VLDTNFGADMTIMEEASELVERVLNGSGELPQFTSCCPAWVKFAETFYPEFLPNLSTAKSPIAMQA
PTQKTYFAEKMGIDAKQMVAVAVTPCTAKKFEIRRDEMNSSAEYWESPEMRDTDYCITTRELAK
WLRAEEVNFDELEDSTFDPLMGEASGGGIIFGNTGGVMEAAMRTAYKLVTGEDAPSTLIPFEAIRG
MDGAREADVVIGDKTLHVAAVHGTGNLRKFIERMRAENIHYDFIEVMACRGGCIGGGGQPRVKL
PMADKAREARIASLYTRDAEVKVKASCDNPDIQKLYAEFFDGKPMSHKAHHMLHTTFVNRSEDL
GPNGACTPATCPTSVPNLKKAAEAAKAAAEAK

DdHydAB subunit fusion variant L4

MSRTVMERIEYEMHTPDPKADPDKLHFVQIDEAKCIGCDTCSQYCPTAAIFGEMGEPHSIPHIEACI
NCGQCLTHCPENAIYEAQSWVPEVEKKLKDGKVKCIAMPAPAVRYALGDAFGMPVGSVTTGKM
LAALQKLGFAHCWDTEFTADVTIWEEGSEFVERLTKKSDMPLPQFTSCCPGWQKYAETYYPELLP
HFSTCKSPIGMNGALAKTYGAERMKYDPKQVYTVSIMPCIAKKYEGLRPELKSSGMRDIDATLTT
RELAYMIKKAGIDFAKLPDGKRDSLMGESTGGATIFGVTGGVMEAALRFAYEAVTGKKPDSWDF
KAVRGLDGIKEATVNVGGTDVKVAVVHGAKRFKQVCDDVKAGKSPYHFIEYMACPGGCVCGGG
Q**PRVKLPMADKAREAR**INGVYGADAKFPVRASQDNTQVKALYKSYLEKPLGHKSHDLLHTHWF
DKSKGVKELTTAGKLPNPRASEFEGPYPYESAWSHPQFEK

***Dd*HydF**

MQDTPKGLRLHIGIYGRRNVGKSSLLNALTRQTVSIVSATPGTTTDPVEKTMEMAPIGPVVFIDTA
GIDDTGDLGLMRSDRTRQMFDRTDLALVASEGDVWGDYERDLVAAFRERKIPVIAVLTKGDETP
VAQTVRQSLLAEGIESAAVCSTDGSGIDALRALMARMAPEHAISQPALLADLVPAGGLVIFVVPID
LGAPKGRLILPQVQAIRDILDGDAMCMVVKERELRPALDRLTQPPDLVVCDSQVVLKAAADTPPG
IPLTTFSILMARFKGELETLARGAAVIETLAPGDRVLVAEACTHHPLADDIGRVKIPRWLRQYAGG
DIHIDTFAGKDMPADLSGYRLIIHCGGCVITRGHMLGRMHAAAAAGVPMTNYGLAISLTQGVLHR
ALGPFPAAQEAFDSIPRGSAWSHPQFEK

Fig. S5| Detailed analysis of amino acid side chain interactions within or between the inserted linkers and adjacent parts of the remaining enzyme structure. (a) Local electron density map (ED) covering the termini/linker elements in the X-ray structures of the heterodimeric wild type. **(f, k, p)** ED-maps for the monomeric subunit fusion variants L1-L3 . **(b)** Termini of the main chains in the X-ray structure for *Dd*HydAB^{WT} (PDB code: 8RTG). **(g, l, q)** Superpositions of these termini with the linkers of monomeric subunit fusion variants L1-L3. **(c)** H-bonds and salt bridge contacts between the termini of *Dd*HydAB^{WT}. **(h, m, r, u)** Corresponding interactions for the linker regions in variants L1-L4. Due to its incomplete X-ray structure, for L4 the model structure generated with Boltz-2 was used (see Fig. S10). Amino acid side chains that may contribute to the structural and steric features of the linker are shown as sticks with their Van-der-Waals radii being presented as transparent spheres. **(d)** Hydrophobic interactions between the termini of the heterodimeric *Dd*HydAB^{WT}. **(i, n, s, v)** Hydrophobic residues and interactions with or within the linker regions of variants L1-L4. **(e)** Polypeptide positions participating in interactions within and with the termini of *Dd*HydAB^{WT}. The same was done for the linker elements of subunit-fusion variants L1-L4 **(j,o,t,w)**. A pair of bars colored in line with the color code for the individual variants covers the sequence range of the respective linker elements. Positions marked in red (acidic) and blue (basic) correspond to salt bridge partners; individual pairs are indicated by red connector lines. Locations labeled with a gray background mark amino acids that contribute to hydrophobic interactions. H-bonds are indicated by black connector lines between the interacting locations. The amino acid sequences of the heterologously expressed *Dd*HydF and the monomeric variants L1-L4 as well as the sequences that served as origins of the respective linker elements are presented below, highlighting the sequence parts contributing to the plug-lock-lid mechanism in cyan, red and pink. Individual linkers are marked by background colors according to the color-code introduced in Fig. 2. The C-terminal Strep-Tag II sequences are indicated in gray.

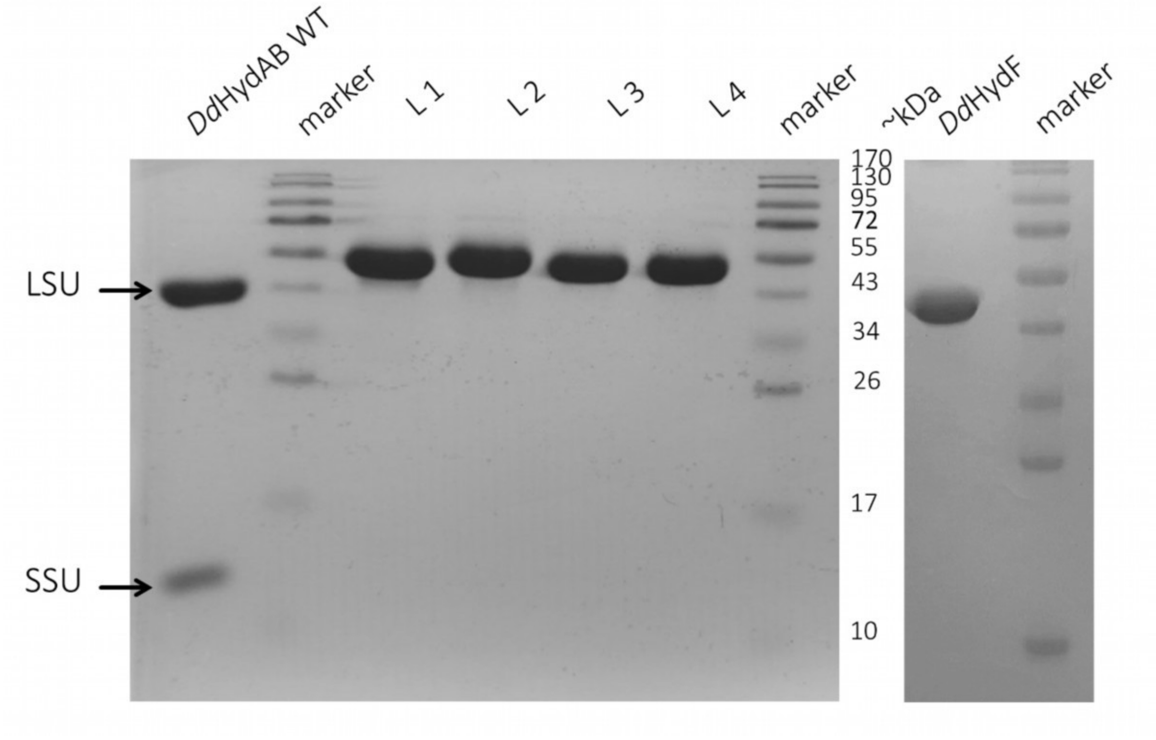

Figure S6| SDS-PAGE of the purified protein samples (expected band sizes: LSU: 45.95 kDa; SSU: 13.62 kDa; L1: 54.20 kDa; L2: 54.23 kDa; L3: 53.98 kDa; L4: 53.98 kDa; HydF: 44.87 kDa). For all samples, 15 μg of protein was loaded onto the gel.

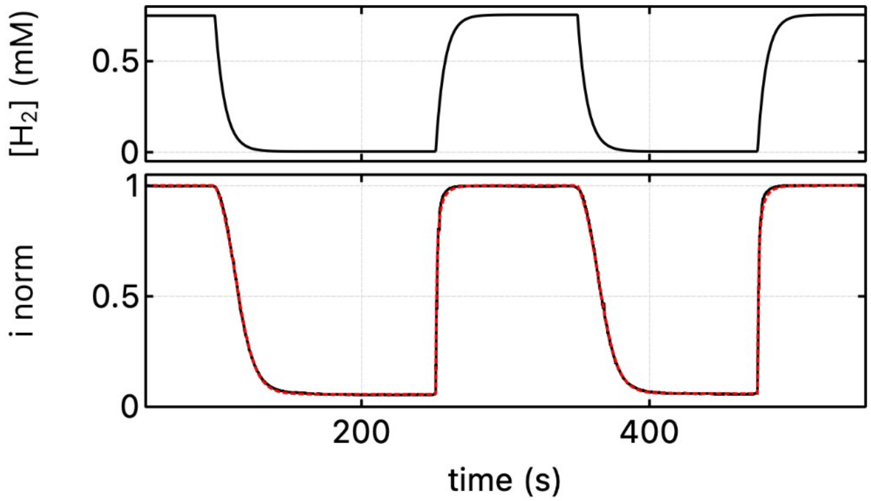

Figure S7| Illustration of the electrochemical measurement of the Michaelis constant for H_2. The hydrogen concentration was varied as shown on the top panel by flushing the electrochemical cell with either H_2 or Ar, resulting in exponential variations of the concentration of H_2. A Michaelis Menten model was fitted (red in the bottom panel) to the resulting current (black in the bottom panel). The data shown here have been recorded with the WT enzyme, but all variants were found to have very similar Michaelis constants for H_2.

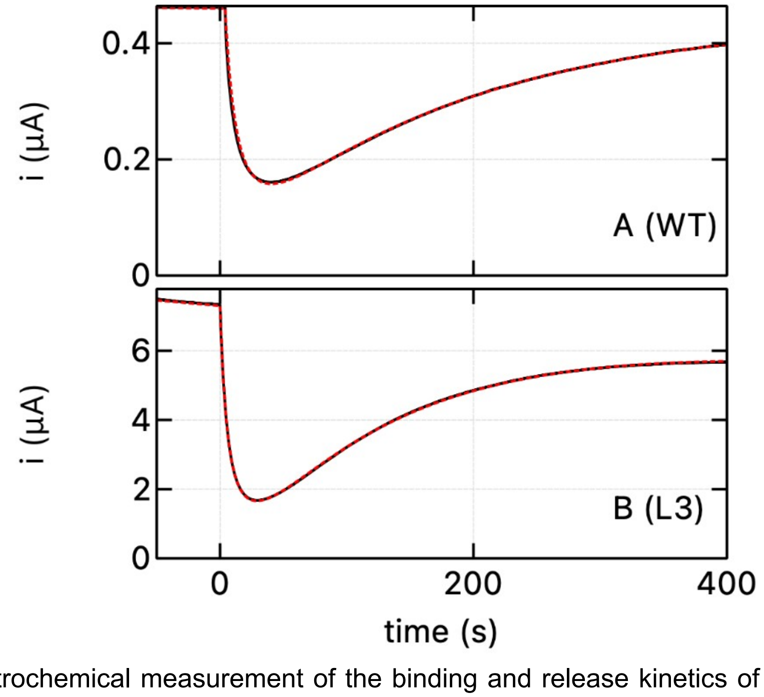

Figure S8| Electrochemical measurement of the binding and release kinetics of CO to the holo-form of the enzyme. The H_2-oxidation current was measured as a function of time following a burst of CO concentration. Here the model (dashed red line) includes a slow current decay that accounts for film loss. The data shown in panels A and B were recorded with the WT and variant L3, respectively.

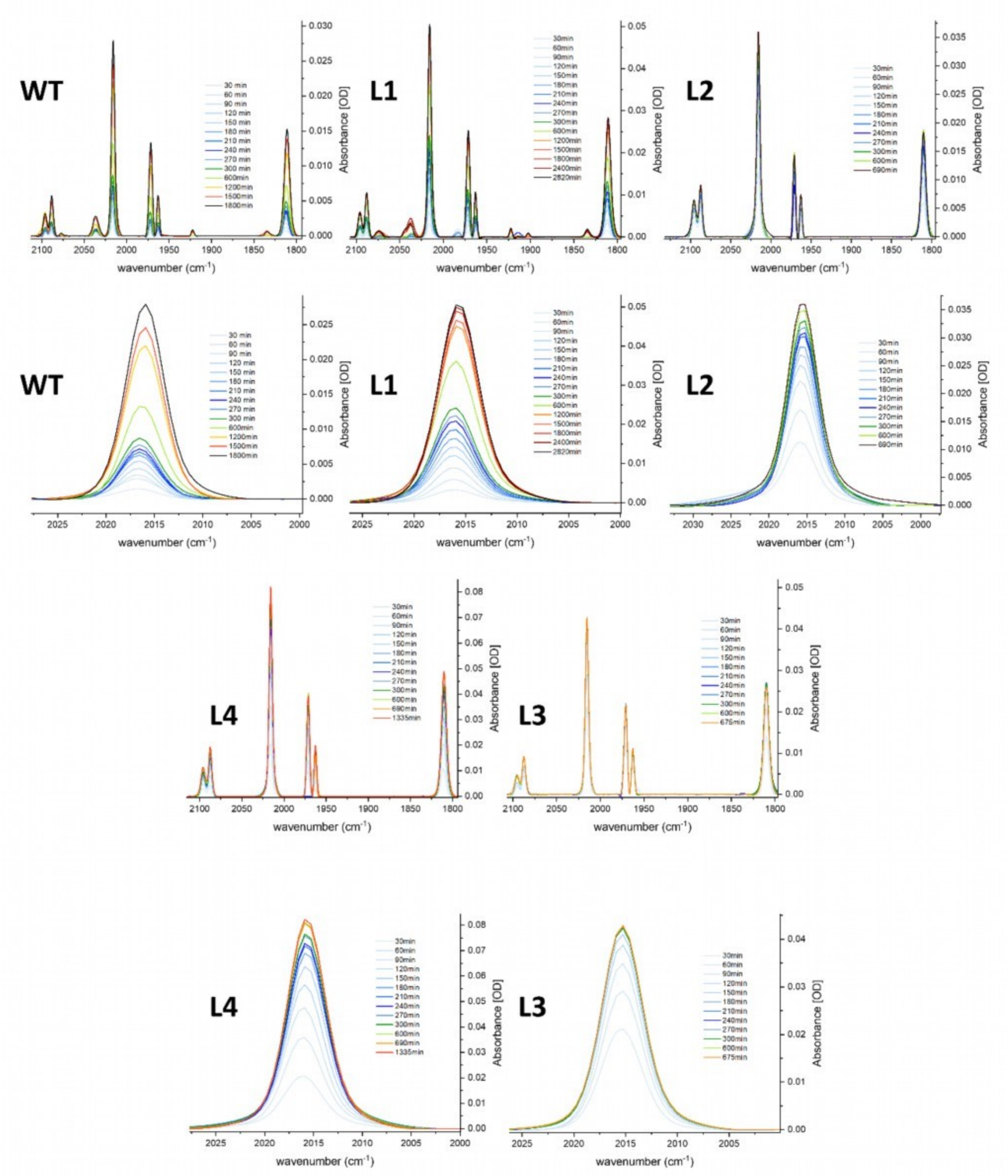

Figure S9| Accumulation of the state-specific transmission FTIR-spectra for $H_{ox}CO$ of *Dd*HydAB and subunit-fusion variants L1-L4 upon combining apo-hydrogenase and the synthetic $2Fe_H^{MIM}$ complex at a 1:3 ratio and close-ups of the respective indicator peak at 2016 cm^{-1}. IR-spectra of 20 µL protein solution (0.4 mM) are shown for selected time points of *in vitro* maturation in a time range between 30 and 2820 min. The corresponding color code clearly shows the significant rate increase according to the succession of WT< L1< L4/L2<L3. *Dd*HydAB^{WT} and variant L1 were both repeatedly measured for significantly longer time periods compared to the faster maturating variants L2-L4 until reaching their saturation level. After 1500-1800 min, minor spectral features of active states (e.g. $H_{red}H^+$) can be observed to slowly arise along with the rapidly increasing $H_{ox}CO$ state, suggesting that a growing fraction of the CO-inhibited holo-enzyme releases its surplus apical CO ligand possibly due to tiny leakages or inhomogeneous distributions of CO within the sealed FTIR-cell.

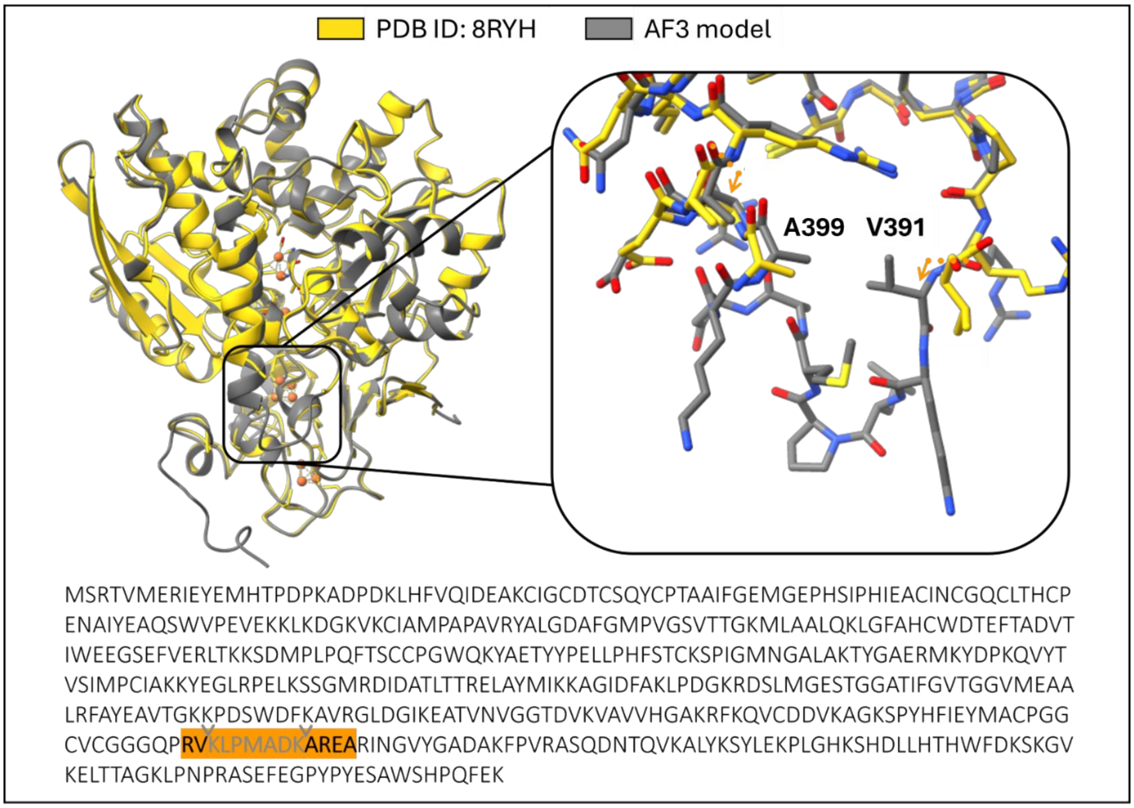

Figure S10| Superposition of the Boltz-2 [27] model (gray) and X-ray structure (yellow, resolution: 1.77 Å) of L4 with a close-up of the linker region. The linker region is highlighted in the protein sequence. The not resolved part of the linker element in the crystal structure of L4 is indicated in the protein sequence in gray (pLDDT (per-residue confidence score) in the model structure covering positions 392-398: 0.74 - 0.84, corresponding to the general evaluation ‘confident’).

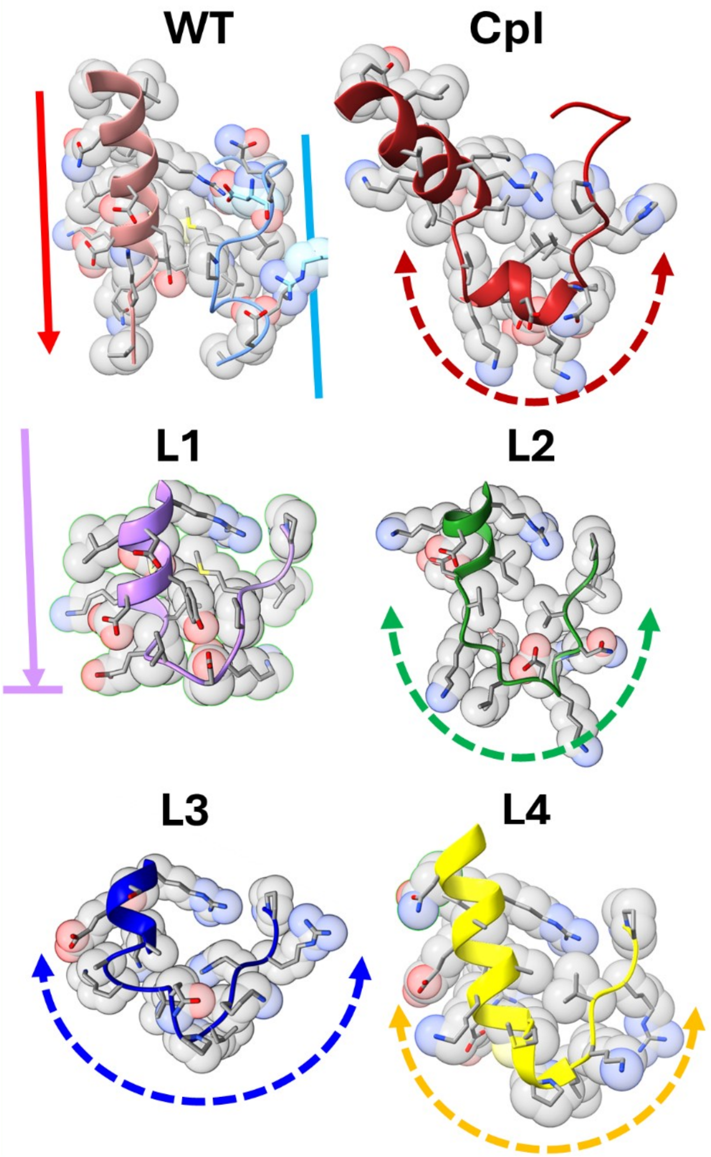

Fig. S11| Local packing density of amino acid residues at the termini of LSU and SSU subunits of *Dd*HydAB^{WT} compared to the native linker element in *Cp*I and to the designed linkers in the monomeric subunit fusion variants L1-L4. The linker structure of variant L4 was modelled via Boltz-2. The inverse directed arrows along the unconnected termini of the wildtype structure suggest an instability of this conformation via shear forces. In the case of L1 the packing density is especially high, suggesting that this linker lacks the required level of flexibility to effectively support the structural rearrangement of the lock-element during binding site closure. The lower packing densities of residues in the linker elements of *Cp*I, L2 and especially L3 suggest that these linkers may provide the necessary level of flexibility (indicated by the broken circular double arrows) to support a fast restructuring of the lock-element and thus a rapid stable closure and activation of these enzymes. In the case of L4 the Boltz-2 model suggests only a low number of hydrophobic contacts, which may easily be disrupted preventing the stalled packing shown for the linker in L1.

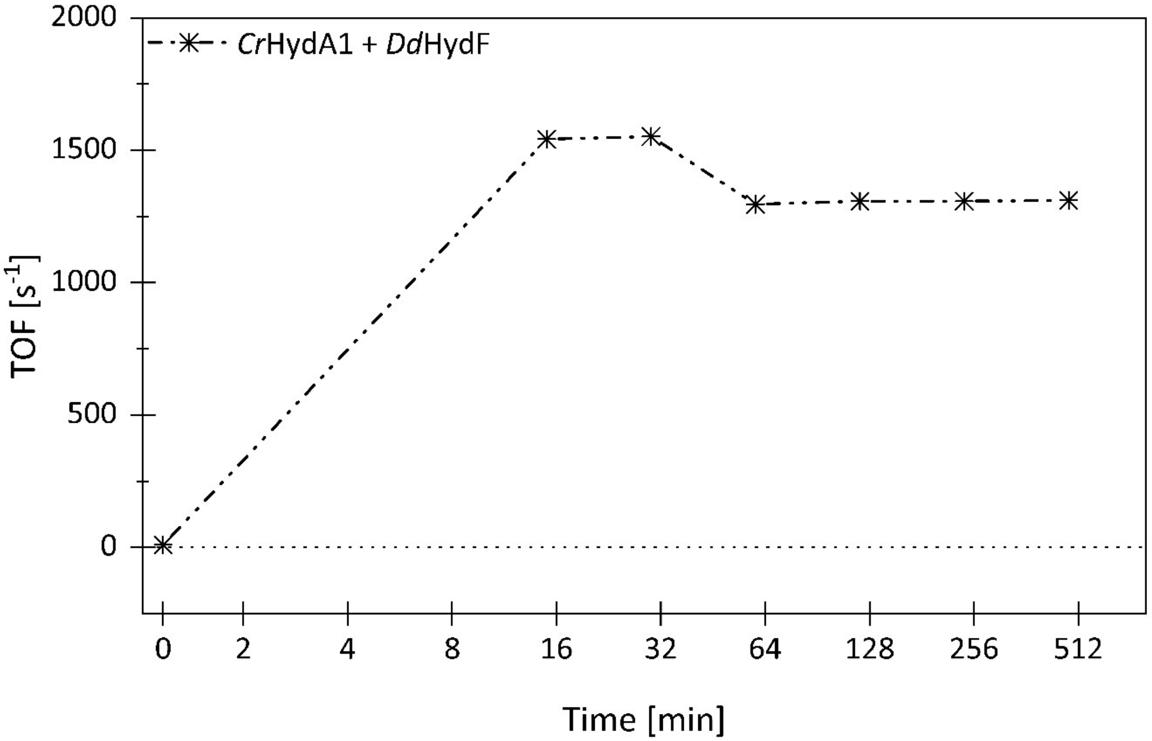

Figure S12| Progression of *in vitro* maturation of *Cr*HydA1 by periodically testing aliquots from an *in vitro* maturation mix for the catalytic competency of H_2-evolution activity using a 3-fold excess of $2FeH^{MIM}$-loaded HydF, applying a standard methyl viologen-mediated activity assay. This procedure was first tested with the apo-state of [FeFe]-hydrogenase HydA1 of *Chlamydomonas reinhardtii*, which was used as a control to establish the HydF-mediated version of the *in vitro* maturation procedure first described by Berggren and coauthors.[29] Even exceeding their results obtained with HydF of *Thermotoga maritima*, *Cr*HydA activity reached its top level after only 16 min of apo-HydA1/$2Fe_H$-HydF preincubation at a 3.3-fold lower surplus of holo-HydF than used by Berggren and co-workers. This confirms the effectiveness of the present approach and suggests a sufficiently high level of compatibility between the green-algal apo-*Cr*HydA1 hydrogenase and HydF of *D. desulfuricans* ATCC 7757, which would be a precondition for directed complex formation and fast cofactor transfer.

Supporting Tables

Table S1| Kinetics of CO binding to and release from the different variants and Michaelis constant for H_2, measured by protein film electrochemistry, at pH 7, T=20 °C. The apparent binding rate constants measured under one atm. of H_2 were corrected to account for the protective effect of H_2.

enzyme	k_{in}^{app} ($s^{-1}mM^{-1}$)	k_{out} (10^{-3} s^{-1})	K_M (H_2) (mM)	K_i (CO) (nM)
**WT**	890±100	4.7±0.2	0.09	0.53
**L1**	1240±150	5.1±0.3	0.08	0.37
**L2**	1890±200	6.2±0.5	0.09	0.33
**L3**	1440±150	6.7±0.3	0.08	0.42
**L4**	1680±150	6.3±0.3	0.10	0.42

Table S2| Comparison of half times ($t_{1/2}$) and times needed to reach 99% of maximal H-cluster occupancy or enzyme activation in the maturation samples (t_{99}) monitored via Fourier-transform-infrared (FTIR) spectroscopy or H_2 evolution activity assay (HER) for *Dd*HydAB and variants L1-L4. To provide a better basis for comparing the results of both approaches, the decreases in $t_{1/2}$ for the fusion variants L1-L4 have been presented relative to *Dd*HydAB (decr. $t_{1/2}$). Errors represent the standard error of fit parameters based on the covariance matrix of parameters.

sample	$t_{1/2}$ HER [min]	$t_{1/2}$ FTIR [min]	t_{99} HER [min]	t_{99} FTIR [min]	fold-decr. $t_{1/2}$ HER	fold-decr. $t_{1/2}$ FTIR
***Dd*HydAB**	166 ± 25	1291 ± 52	1120 ± 143	8499 ± 340	1 ± 15	1 ± 0.1
**L1**	67 ± 6	343 ± 5	443 ± 40	2288 ± 14	2.5 ± 0.4	3.8 ± 0.2
**L2**	14 ± 1	71 ± 2	95 ± 6	462 ± 15	12 ± 2	18 ± 1
**L3**	4 ± 1.	35 ± 1	19 ± 4	230 ± 7	41 ± 10	37 ± 2
**L4**	10 ± 1	69.6 ± 1	68 ± 6	462 ± 7	16 ± 3	19 ± 1
**CpI**	1.7 ± 0.3	//	8 ± 1	//	95 ± 20	//

$t_{1/2}$- and t_{99}-values have been derived from exponential fits of the data presented in Fig. 3a+b as symbols according to equation: $y = A * \exp\left(-\frac{x}{t}\right) + y0$ with R^2-values higher than 0.99 for all fits, except of the H_2 evolution assay for *Dd*HydAB (R^2=0.984). Fitting was done by using the ExpDec1-function of Origin 2024.

Table S3| Data collection and refinement statistics.

	L1 (PDB ID: 8RU6)	L2 (PDB ID: 9GNK)	L3 (PDB ID: 9GBU)	L4 (PDB ID: 8RYH)	apo-*Dd*HydAB (PDB ID: 8RTG)	holo-*Dd*HydAB^{WT} (PDB ID: 9QD6)
**Data collection**						
Space group	$P2_12_12_1$	$P2_12_12_1$	$P2_12_12_1$	C2	$P2_12_12_1$	$P2_12_12_1$
Cell dimensions						
a, *b*, *c* (Å)	49.40 88.86 106.59	49.14, 89.21, 106.45	93.02, 98.81, 112.21	106.12, 50.60, 86.64	49.15, 84.79, 88.04	49.44, 87.27,89.17
α, β, γ (°)	90.00, 90.00, 90.00	90.00, 90.00, 90.00	90.00, 90.00, 90.00	90.00, 105.33, 90.00	90.00, 90.00, 90.00	90.00, 90.00, 90.00
Resolution (Å)*	45.71-1.15 (1.17-1.15)	45.71-1.05 (1.07-1.05)	48.79-1.78 (1.82-1.78)	45.36-1.77 (1.81-1.77)	49.15-1.46 (1.49-1.46)	49.44-1.18 (1.20-1.18)
R_{merge}*	0.103 (1.834)	0.058 (1.737)	0.101 (2.141)	0.159 (1.058)	0.450 (3.556)	0.176 (1.703)
I / σI	15.4 (1.5)	19.9 (1.6)	13.3 (1.4)	8.9 (2.3)	6.3 (1.3)	10.5 (2.2)
Completeness (%)*	100.0 (99.8)	99.9 (99.6)	99.6 (98.3)	96.4 (55.8)	100 (99.7)	99.9 (99.8)
Multiplicity*	13.6 (11.9)	13.3 (13.2)	13.4 (13.6)	6.9 (7.0)	13.9 (14.4)	13.6 (13.9)
CC(1/2)*	1.000 (0.621)	1.000 (0.581)	0.999 (0.569)	0.996 (0.726)	0.995 (0.505)	0.999 (0.667)
Anomalous completeness*	100.0 (99.8)	99.8 (99.6)	99.6 (98.3)	95.8 (55.3)	100.0 (99.7)	99.8 (99.8)
Anomalous multiplicity*	7.0 (6.1)	6.8 (6.6)	6.9 (6.9)	3.5 (3.6)	7.2 (7.4)	7.0 (7.1)
**Refinement**						
Resolution (Å)	45.75-1.15	45.75-1.05	48.84-1.78	45.40-1.77	44.06-1.46	44.62-1.18
No. reflections	166548	217156	98317	41647	64506	127008
R_{work} / R_{free}	0.137 / 0.158	0.168 / 0.185	0.201 / 0.242	0.205 / 0.246	0.165 / 0.189	0.151 / 0.168
No. atoms						
Protein	3787	3796	3707/3737**	3621	3796	3793
Ligand/ion	98	110	73/57**	41	24/2***	65
Water	480	565	221	117	329	375
B-factors						
Protein	14.51	12.97	40.06/43.41**	20.5	15.68	12.15
Ligand/ion	21.54	19.28	50.77/40.31**	15.13	11.55/16.32***	17.04
Water	26.89	23.26	39.6	20.53	21.74	20.66
R.m.s. deviations						
Bond lengths (Å)	0.0122	0.0043	0.0097	0.0082	0.0093	0.0139
Bond angles (°)	2.112	1.604	1.897	1.861	1.890	2.02

*Values in parentheses are for highest-resolution shell
**First value is for molecule A and second value for molecule B
***Values for ligands and ions separately

Table S4| Oligonucleotides used in the mega-primer PCR for fusing the large and small subunit of *Dd*HydAB, including individual linker segments for constructs L1-L4.

#	Construct	Target	fwd/rev	Sequence (5'➔3')
1	L1	small subunit	fwd	GCCGAAAGACCTGGAAATTAAAGATTATATGCTGGACCGTATCAAC
2	L1	small subunit	rev	CAGTCGAGCTCTTATTTTTCAAATTGCGGATGGGACCAGGCTGATTCATACGGATACGGACCTTCAAATTC
3	L1	large subunit	fwd	GAGATCATATGTCCCGCACCGTGATGGAACGCATTGAA
4	L1	large subunit	rev	TTTCCAGGTCTTTCGGCATAACCGGTTGGCCACCGCCACAC
5	L2	small subunit	fwd	CCTGGAAAAAGTGGACATCAAAAAAGACCGTATCAACGGCGTCTA
6	L2	small subunit	rev	CAGTCGAGCTCTTATTTTTCAAATTGCGGATGGGACCAGGCTGATTCATACGGATACGGACCTTCAAATTC
7	L2	large subunit	fwd	GAGATCATATGTCCCGCACCGTGATGGAACGCATTGAA
8	L2	large subunit	rev	ATGTCCACTTTTTCCAGGTCTTTTGGGTTTACAACCGGTTGGCCACCGCCACACAC
9	L3	small subunit	fwd	CTTTTGCTGCTTTAACCTGAGGCAGTTTATGACGCGGTTGGCCACCGCCACACAC
10	L3	small subunit	rev	CAGTCGAGCTCTTATTTTTCAAATTGCGGATGGGACCAGGCTGATTCATACGGATACGGACCTTCAAATTC
11	L3	large subunit	fwd	GAGATCATATGTCCCGCACCGTGATGGAACGCATTGAA
12	L3	large subunit	rev	CTTTTGCTGCTTTAACCTGAGGCAGTTTATGACGCGGTTGGCCACCGCCACACAC
13	L4	small subunit	fwd	GATGGCAGATAAAGCACGTGAAGCACGTATCAACGGCGTCTACGG
14	L4	small subunit	rev	CAGTCGAGCTCTTATTTTTCAAATTGCGGATGGGACCAGGCTGATTCATACGGATACGGACCTTCAAATTC
15	L4	large subunit	fwd	GAGATCATATGTCCCGCACCGTGATGGAACGCATTGAA
16	L4	large subunit	rev	CGTGCTTTATCTGCCATCGGCAGTTTAACACGCGGTTGGCCACCGCCACACAC
A	Fusion PCR small subunit/large subunit		fwd	GAGATCATATGTCCCGCACCGTGATGGAACGCATTGAA
B			rev	CAGTCGAGCTCTTATTTTTCAAATTGCGGATGGGACCAGGCTGATTCATACGGATACGGACCTTCAAATTC

REFERENCES

1. J. A. Birrell, K. Wrede, K. Pawlak, P. Rodriguez-Maciá, O. Rüdiger, E. J. Reijerse and W. Lubitz, Artificial Maturation of the Highly Active Heterodimeric [FeFe] Hydrogenase from Desulfovibrio desulfuricans ATCC 7757, *Israel Journal of Chemistry*, 2016, **56**, 852–863.
2. M. K. Akhtar and P. R. Jones, Deletion of iscR stimulates recombinant clostridial Fe-Fe hydrogenase activity and H2-accumulation in Escherichia coli BL21(DE3), *Applied microbiology and biotechnology*, 2008, **78**, 853–862.
3. J. M. Kuchenreuther, C. S. Grady-Smith, A. S. Bingham, S. J. George, S. P. Cramer and J. R. Swartz, High-yield expression of heterologous [FeFe] hydrogenases in Escherichia coli, *PLoS One*, 2010, **5**, e15491.
4. S. Yadav, R. Haas, E. B. Boydas, M. Roemelt, T. Happe, U.-P. Apfel and S. T. Stripp, Oxygen sensitivity of [FeFe]-hydrogenase: a comparative study of active site mimics inside vs. outside the enzyme, *Physical Chemistry Chemical Physics*, 2024, **26**, 19105–19116.
5. J. Esselborn, C. Lambertz, A. Adamska-Venkatesh, T. Simmons, G. Berggren, J. Noth, J. Siebel, A. Hemschemeier, V. Artero, E. Reijerse, M. Fontecave, W. Lubitz and T. Happe, Spontaneous activation of [FeFe]-hydrogenases by an inorganic [2Fe] active site mimic, *Nat Chem Biol*, 2013, **9**, 607–609.
6. O. Lampret, J. Esselborn, R. Haas, A. Rutz, R. L. Booth, L. Kertess, F. Wittkamp, C. F. Megarity, F. A. Armstrong, M. Winkler and T. Happe, The final steps of [FeFe]-hydrogenase maturation, *Proceedings of the National Academy of Sciences of the United States of America*, 2019, **116**, 15802–15810.
7. C. Léger, S. Dementin, P. Bertrand, M. Rousset and B. Guigliarelli, Inhibition and Aerobic Inactivation Kinetics of Desulfovibrio fructosovorans NiFe Hydrogenase Studied by Protein Film Voltammetry, *Journal of the American Chemical Society*, 2004, **126**, 12162–12172.
8. T. J. Morrison and F. Billett, 730. The salting-out of non-electrolytes. Part II. The effect of variation in non-electrolyte, *Journal of the Chemical Society (Resumed)*, 1952, DOI: 10.1039/JR9520003819, 3819–3822.
9. T. J. Morrison, 729. The salting-out of non-electrolytes. Part I. The effect of ionic size, ionic charge, and temperature, *Journal of the Chemical Society (Resumed)*, 1952, DOI: 10.1039/JR9520003814, 3814–3818.
10. F. Leroux, S. Dementin, B. Burlat, L. Cournac, A. Volbeda, S. Champ, L. Martin, B. Guigliarelli, P. Bertrand, J. Fontecilla-Camps, M. Rousset and C. Léger, Experimental approaches to kinetics of gas diffusion in hydrogenase, *Proceedings of the National Academy of Sciences*, 2008, **105**, 11188–11193.
11. C. Baffert, L. Bertini, T. Lautier, C. Greco, K. Sybirna, P. Ezanno, E. Etienne, P. Soucaille, P. Bertrand, H. Bottin, I. Meynial-Salles, L. De Gioia and C. Léger, CO Disrupts the Reduced H-Cluster of FeFe Hydrogenase. A Combined DFT and Protein Film Voltammetry Study, *Journal of the American Chemical Society*, 2011, **133**, 2096–2099.
12. V. Fourmond, QSoas: A Versatile Software for Data Analysis, *Analytical Chemistry*, 2016, **88**, 5050–5052.
13. M. G. Almeida, C. M. Silveira, B. Guigliarelli, P. Bertrand, J. J. G. Moura, I. Moura and C. Léger, A needle in a haystack: The active site of the membrane-bound complex cytochrome c nitrite reductase, *FEBS Letters*, 2007, **581**, 284–288.
14. A. Meents, B. Reime, N. Stuebe, P. Fischer, M. Warmer, D. Göries, J. Roever, J. Meyer, J. Fischer, A. Burkhardt, I. Vartiainen, P. Karvinen and C. David, Development of an in-vacuum X-ray microscope with cryogenic sample cooling for beamline P11 at PETRA III, *Proc SPIE*, 2013, **8851**.
15. A. Burkhardt, T. Pakendorf, B. Reime, J. Meyer, P. Fischer, N. Stübe, S. Panneerselvam, O. Lorbeer, K. Stachnik, M. Warmer, P. Rödig, D. Göries and A. Meents, Status of the crystallography beamlines at PETRA III, *The European Physical Journal Plus*, 2016, **131**, 56.
16. W. Kabsch, Integration, scaling, space-group assignment and post-refinement, *Acta crystallographica. Section D, Biological crystallography*, 2010, **66**, 133–144.
17. P. R. Evans and G. N. Murshudov, How good are my data and what is the resolution?, *Acta Crystallographica Section D*, 2013, **69**, 1204–1214.
18. A. Vagin and A. Teplyakov, MOLREP: an Automated Program for Molecular Replacement, *Journal of Applied Crystallography*, 1997, **30**, 1022–1025.
19. A. J. McCoy, R. W. Grosse-Kunstleve, P. D. Adams, M. D. Winn, L. C. Storoni and R. J. Read, Phaser crystallographic software, *J Appl Crystallogr*, 2007, **40**, 658–674.
20. C. K., The Buccaneer software for automated model building. 1. Tracing protein chains, *Acta Crystallographica Section* 2006, **62**, 1002–1011.
21. A. A. V. a. E. J. D. G.N. Murshudov, Refinement of Macromolecular Structures by the Maximum-Likelihood method, *Acta Cryst.*, 1997, **D53**, 240–255.
22. P. Emsley, B. Lohkamp, W. G. Scott and K. Cowtan, Features and development of Coot, *Acta crystallographica. Section D, Biological crystallography*, 2010, **66**, 486–501.

23. M. D. Winn, C. C. Ballard, K. D. Cowtan, E. J. Dodson, P. Emsley, P. R. Evans, R. M. Keegan, E. B. Krissinel, A. G. W. Leslie, A. McCoy, S. J. McNicholas, G. N. Murshudov, N. S. Pannu, E. A. Potterton, H. R. Powell, R. J. Read, A. Vagin and K. S. Wilson, Overview of the CCP4 suite and current developments, *Acta Crystallographica Section D*, 2011, **67**, 235–242.
24. H. Guo, R. Zhong, B. Liu, J. Yang, Z. Liu, C. Du and X. Li, Characteristic and Mechanistic Investigation of Stress-Assisted Microbiologically Influenced Corrosion of X80 Steel in Near-Neutral Solutions.*Journal*, 2023, **16**.
25. Z. Li, J. Yang, H. Guo, S. Kumseranee, S. Punpruk, M. E. Mohamed, M. A. Saleh and T. Gu, Mechanical property degradation of X80 pipeline steel due to microbiologically influenced corrosion caused by Desulfovibrio vulgaris, *Frontiers in Bioengineering and Biotechnology*, 2022, **Volume 10 - 2022**.
26. D. W. Waite, M. Chuvochina, C. Pelikan, D. H. Parks, P. Yilmaz, M. Wagner, A. Loy, T. Naganuma, R. Nakai, W. B. Whitman, M. W. Hahn, J. Kuever and P. Hugenholtz, Proposal to reclassify the proteobacterial classes Deltaproteobacteria and Oligoflexia, and the phylum Thermodesulfobacteria into four phyla reflecting major functional capabilities, *International Journal of Systematic and Evolutionary Microbiology*, 2020, **70**, 5972–6016.
27. S. Passaro, G. Corso, J. Wohlwend, M. Reveiz, S. Thaler, V. R. Somnath, N. Getz, T. Portnoi, J. Roy, H. Stark, D. Kwabi-Addo, D. Beaini, T. Jaakkola and R. Barzilay, Boltz-2: Towards Accurate and Efficient Binding Affinity Prediction, *bioRxiv*, 2025, DOI: 10.1101/2025.06.14.659707.
