# Supplementary material for: Subunit fusion unlocks rapid in vitro maturation for slowly activating heterodimeric [FeFe]-hydrogenases
Source: Chem Sci. 2026 Feb 3;17(15):7678–89. doi: 10.1039/d5sc07299a (PMC12933642; doi:10.1039/d5sc07299a)
Supplement: SC-017-D5SC07299A-s002 [file SC-017-D5SC07299A-s002.pdf]

Supplementary Information (SI) for Chemical Science.
This journal is © The Royal Society of Chemistry 2026

Full wwPDB X-ray Structure Validation Report (i)

Feb 13, 2024 – 10:40 am GMT

PDB ID	:	8RU6
Title	:	Desulfovibrio desulfuricans [FeFe]-hydrogenase variant with both subunits linked by a 4 amino acid linker peptide derived from CpI of Clostridium pasteurianum
Deposited on	:	2024-01-30
Resolution	:	1.15 Å(reported)

This wwPDB validation report is for manuscript review

This is a Full wwPDB X-ray Structure Validation Report.

This report is produced by the wwPDB biocuration pipeline after annotation of the structure.

We welcome your comments at *validation@mail.wwpdb.org*
A user guide is available at
https://www.wwpdb.org/validation/2017/XrayValidationReportHelp
with specific help available everywhere you see the (i) symbol.

The types of validation reports are described at
http://www.wwpdb.org/validation/2017/FAQs#types.

For Manuscript Review

The following versions of software and data (see references (i)) were used in the production of this report:

MolProbity	:	4.02b-467
Mogul	:	1.8.4, CSD as541be (2020)
Xtriage (Phenix)	:	1.13
EDS	:	2.36
buster-report	:	1.1.7 (2018)
Percentile statistics	:	20191225.v01 (using entries in the PDB archive December 25th 2019)
Refmac	:	5.8.0158
CCP4	:	7.0.044 (Gargrove)

1 Overall quality at a glance (i)

The following experimental techniques were used to determine the structure:
X-RAY DIFFRACTION

The reported resolution of this entry is 1.15 Å.

Percentile scores (ranging between 0-100) for global validation metrics of the entry are shown in the following graphic. The table shows the number of entries on which the scores are based.

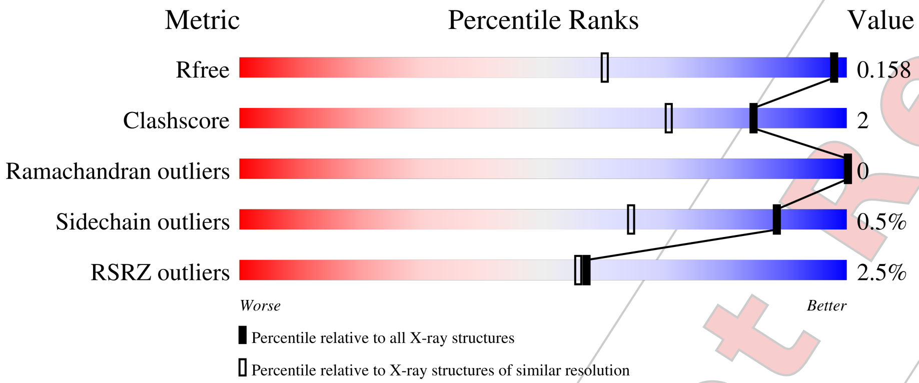

Metric	Whole archive (#Entries)	Similar resolution (#Entries, resolution range(Å))
R_{free}	130704	1492 (1.18-1.10)
Clashscore	141614	1537 (1.18-1.10)
Ramachandran outliers	138981	1483 (1.18-1.10)
Sidechain outliers	138945	1480 (1.18-1.10)
RSRZ outliers	127900	1464 (1.18-1.10)

The table below summarises the geometric issues observed across the polymeric chains and their fit to the electron density. The red, orange, yellow and green segments of the lower bar indicate the fraction of residues that contain outliers for >=3, 2, 1 and 0 types of geometric quality criteria respectively. A grey segment represents the fraction of residues that are not modelled. The numeric value for each fraction is indicated below the corresponding segment, with a dot representing fractions <=5% The upper red bar (where present) indicates the fraction of residues that have poor fit to the electron density. The numeric value is given above the bar.

Mol	Chain	Length	Quality of chain
1	B	491	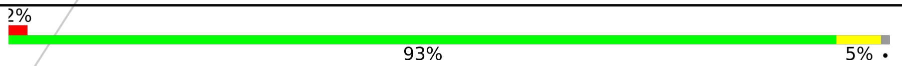

Ideal geometry (proteins) : Engh & Huber (2001)
Ideal geometry (DNA, RNA) : Parkinson et al. (1996)
Validation Pipeline (wwPDB-VP) : 2.36

For Manuscript Review

2 Entry composition (i)

There are 5 unique types of molecules in this entry. The entry contains 8204 atoms, of which 3839 are hydrogens and 0 are deuteriums.

In the tables below, the ZeroOcc column contains the number of atoms modelled with zero occupancy, the AltConf column contains the number of residues with at least one atom in alternate conformation and the Trace column contains the number of residues modelled with at most 2 atoms.

- Molecule 1 is a protein called Periplasmic [Fe] hydrogenase large subunit,CpI,Periplasmic [Fe] hydrogenase small subunit.

Mol	Chain	Residues	Atoms						ZeroOcc	AltConf	Trace
			Total	C	H	N	O	S			
1	B	484	7543	2412	3756	631	706	38	122	9	0

There are 10 discrepancies between the modelled and reference sequences:

Chain	Residue	Modelled	Actual	Comment	Reference
B	482	SER	-	expression tag	UNP P07603
B	483	ALA	-	expression tag	UNP P07603
B	484	TRP	-	expression tag	UNP P07603
B	485	SER	-	expression tag	UNP P07603
B	486	HIS	-	expression tag	UNP P07603
B	487	PRO	-	expression tag	UNP P07603
B	488	GLN	-	expression tag	UNP P07603
B	489	PHE	-	expression tag	UNP P07603
B	490	GLU	-	expression tag	UNP P07603
B	491	LYS	-	expression tag	UNP P07603

- Molecule 2 is TETRAETHYLENE GLYCOL (three-letter code: PG4) (formula: $C_8H_{18}O_5$).

For Manuscript Review

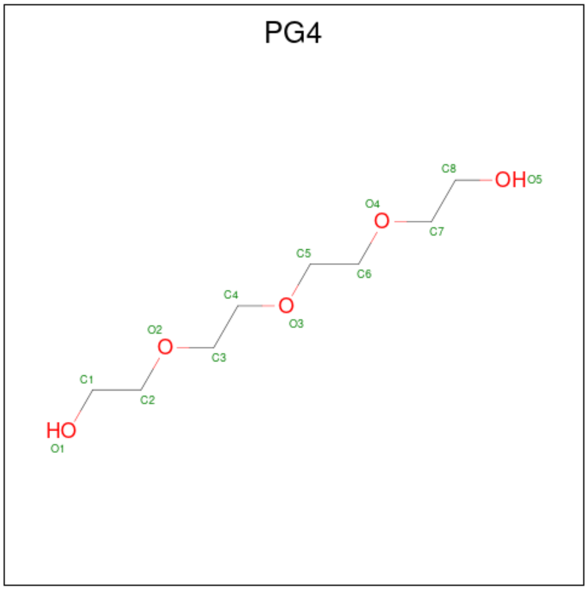

Mol	Chain	Residues	Atoms	ZeroOcc	AltConf
2	B	1	Total C H O<br>26 7 15 4	1	0
2	B	1	Total C H O<br>31 8 18 5	1	0
2	B	1	Total C H O<br>12 3 7 2	1	0
2	B	1	Total C H O<br>62 16 36 10	2	1

- Molecule 3 is IRON/SULFUR CLUSTER (three-letter code: SF4) (formula: Fe_4S_4) (labeled as "Ligand of Interest" by depositor).

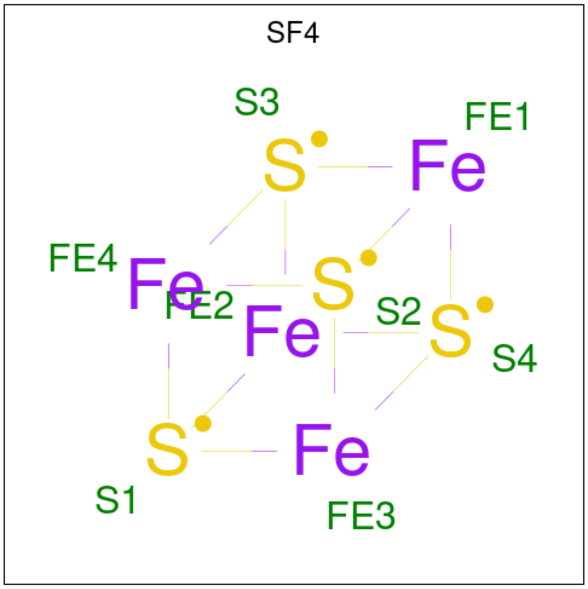

For Manuscript Review

Mol	Chain	Residues	Atoms			ZeroOcc	AltConf
			Total	Fe	S		
3	B	1	8	4	4	0	0
3	B	1	8	4	4	0	0
3	B	1	8	4	4	0	0

- Molecule 4 is Binuclear [FeFe], di(thiomethyl)amine, carbon monoxide, cyanide cluster (-CO form) (three-letter code: MHX) (formula: $C_8H_5Fe_2N_3O_4S_2$) (labeled as "Ligand of Interest" by depositor).

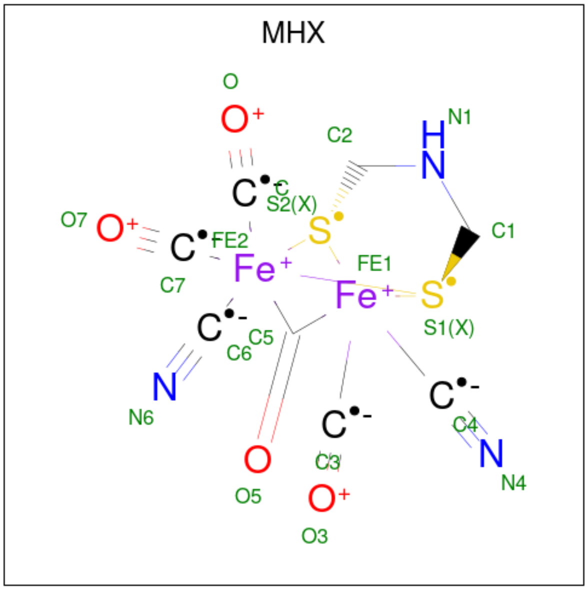

Mol	Chain	Residues	Atoms							ZeroOcc	AltConf
			Total	C	Fe	H	N	O	S		
4	B	1	26	8	2	7	3	4	2	0	0

- Molecule 5 is water.

Mol	Chain	Residues	Atoms		ZeroOcc	AltConf
			Total	O		
5	B	480	480	480	0	0

For Manuscript Review

3 Residue-property plots (i)

These plots are drawn for all protein, RNA, DNA and oligosaccharide chains in the entry. The first graphic for a chain summarises the proportions of the various outlier classes displayed in the second graphic. The second graphic shows the sequence view annotated by issues in geometry and electron density. Residues are color-coded according to the number of geometric quality criteria for which they contain at least one outlier: green = 0, yellow = 1, orange = 2 and red = 3 or more. A red dot above a residue indicates a poor fit to the electron density (RSRZ > 2). Stretches of 2 or more consecutive residues without any outlier are shown as a green connector. Residues present in the sample, but not in the model, are shown in grey.

• Molecule 1: Periplasmic [Fe] hydrogenase large subunit,CpI,Periplasmic [Fe] hydrogenase small subunit

Chain B:

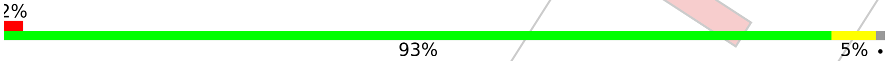

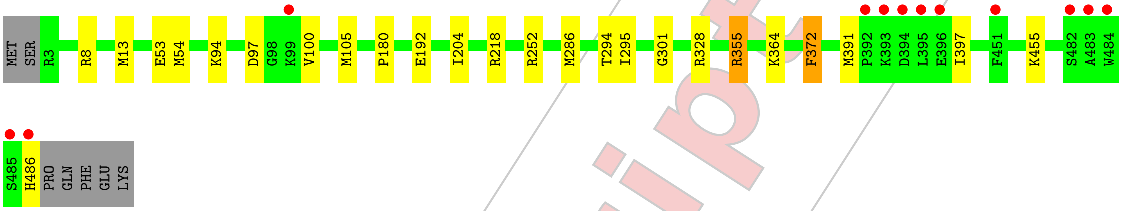

For Manuscript Review

4 Data and refinement statistics (i)

Property	Value	Source
Space group	P 21 21 21	Depositor
Cell constants<br>a, b, c, α, β, γ	49.40Å 88.86Å 106.59Å<br>90.00° 90.00° 90.00°	Depositor
Resolution (Å)	45.75 – 1.15<br>45.71 – 1.15	Depositor<br>EDS
% Data completeness<br>(in resolution range)	99.9 (45.75-1.15)<br>99.9 (45.71-1.15)	Depositor<br>EDS
R_{merge}	0.10	Depositor
R_{sym}	(Not available)	Depositor
$< I/\sigma(I) >$ [1]	1.48 (at 1.15Å)	Xtriage
Refinement program	REFMAC 5.8.0425	Depositor
R, R_{free}	0.137 , 0.158<br>0.138 , 0.158	Depositor<br>DCC
R_{free} test set	8363 reflections (5.02%)	wwPDB-VP
Wilson B-factor (Å^2)	11.0	Xtriage
Anisotropy	0.439	Xtriage
Bulk solvent k_{sol}(e/Å^3), B_{sol}(Å^2)	0.39 , 44.7	EDS
L-test for twinning[2]	$< \lvert L \rvert > = 0.48$, $< L^2 > = 0.30$	Xtriage
Estimated twinning fraction	No twinning to report.	Xtriage
F_o,F_c correlation	0.98	EDS
Total number of atoms	8204	wwPDB-VP
Average B, all atoms (Å^2)	15.0	wwPDB-VP

Xtriage's analysis on translational NCS is as follows: *The largest off-origin peak in the Patterson function is 4.02% of the height of the origin peak. No significant pseudotranslation is detected.*

[1] Intensities estimated from amplitudes.

[2] Theoretical values of $< \lvert L \rvert >$, $< L^2 >$ for acentric reflections are 0.5, 0.333 respectively for untwinned datasets, and 0.375, 0.2 for perfectly twinned datasets.

For Manuscript Review

5 Model quality (i)

5.1 Standard geometry (i)

Bond lengths and bond angles in the following residue types are not validated in this section: SF4, PG4, MHX

The Z score for a bond length (or angle) is the number of standard deviations the observed value is removed from the expected value. A bond length (or angle) with $|Z| > 5$ is considered an outlier worth inspection. RMSZ is the root-mean-square of all Z scores of the bond lengths (or angles).

Mol	Chain	Bond lengths		Bond angles	
		RMSZ	#|Z| >5	RMSZ	#|Z| >5
1	B	0.55	2/3909 (0.1%)	0.90	7/5287 (0.1%)

Chiral center outliers are detected by calculating the chiral volume of a chiral center and verifying if the center is modelled as a planar moiety or with the opposite hand.A planarity outlier is detected by checking planarity of atoms in a peptide group, atoms in a mainchain group or atoms of a sidechain that are expected to be planar.

Mol	Chain	#Chirality outliers	#Planarity outliers
1	B	0	2

All (2) bond length outliers are listed below:

Mol	Chain	Res	Type	Atoms	Z	Observed(Å)	Ideal(Å)
1	B	355	ARG	NE-CZ	-5.26	1.26	1.33
1	B	355	ARG	CG-CD	5.09	1.64	1.51

All (7) bond angle outliers are listed below:

Mol	Chain	Res	Type	Atoms	Z	Observed(^{o})	Ideal(^{o})
1	B	13	MET	CG-SD-CE	-10.41	83.54	100.20
1	B	355	ARG	NE-CZ-NH2	-8.31	116.15	120.30
1	B	286	MET	CG-SD-CE	-7.78	87.76	100.20
1	B	328	ARG	NE-CZ-NH2	-7.24	116.68	120.30
1	B	252	ARG	NE-CZ-NH1	-6.26	117.17	120.30
1	B	372	PHE	CB-CG-CD1	5.45	124.62	120.80
1	B	53	GLU	CG-CD-OE1	5.21	128.71	118.30

There are no chirality outliers.

All (2) planarity outliers are listed below:

For Manuscript Review

Mol	Chain	Res	Type	Group
1	B	355	ARG	Sidechain
1	B	8	ARG	Sidechain

5.2 Too-close contacts (i)

In the following table, the Non-H and H(model) columns list the number of non-hydrogen atoms and hydrogen atoms in the chain respectively. The H(added) column lists the number of hydrogen atoms added and optimized by MolProbity. The Clashes column lists the number of clashes within the asymmetric unit, whereas Symm-Clashes lists symmetry-related clashes.

Mol	Chain	Non-H	H(model)	H(added)	Clashes	Symm-Clashes
1	B	3787	3756	3738	16	2
2	B	55	76	72	9	0
3	B	24	0	0	1	0
4	B	19	7	0	1	0
5	B	480	0	0	4	1
All	All	4365	3839	3810	18	2

The all-atom clashscore is defined as the number of clashes found per 1000 atoms (including hydrogen atoms). The all-atom clashscore for this structure is 2.

All (18) close contacts within the same asymmetric unit are listed below, sorted by their clash magnitude.

Atom-1	Atom-2	Interatomic distance (Å)	Clash overlap (Å)
1:B:94:LYS:HZ2	2:B:502:PG4:H61	1.47	0.78
2:B:502:PG4:O1	2:B:503:PG4:C6	2.35	0.74
1:B:54:MET:HG3	5:B:602:HOH:O	1.87	0.72
1:B:94:LYS:NZ	2:B:502:PG4:H32	2.18	0.58
1:B:100:VAL:HG22	2:B:502:PG4:H72	1.88	0.56
1:B:105[B]:MET:HE3	1:B:204:ILE:HD11	1.87	0.56
1:B:391[B]:MET:HB3	5:B:615:HOH:O	2.08	0.53
1:B:397:ILE:HG13	5:B:615:HOH:O	2.08	0.52
1:B:192:GLU:OE2	1:B:364:LYS:HD2	2.10	0.51
1:B:94:LYS:HD3	2:B:503:PG4:H72	1.93	0.50
1:B:295:ILE:O	1:B:301:GLY:HA3	2.13	0.49
1:B:97:ASP:OD2	2:B:503:PG4:H81	2.13	0.48
4:B:508:MHX:C	4:B:508:MHX:N1	2.78	0.47
1:B:94:LYS:HE2	2:B:502:PG4:H11	1.97	0.46
1:B:94:LYS:NZ	2:B:502:PG4:H61	2.26	0.44
1:B:391[B]:MET:CB	5:B:615:HOH:O	2.65	0.43
1:B:180:PRO:HD2	3:B:506:SF4:S1	2.59	0.42

Continued on next page...

For Manuscript Review

Continued from previous page...

Atom-1	Atom-2	Interatomic distance (Å)	Clash overlap (Å)
1:B:97:ASP:OD2	2:B:503:PG4:C8	2.68	0.41

All (2) symmetry-related close contacts are listed below. The label for Atom-2 includes the symmetry operator and encoded unit-cell translations to be applied.

Atom-1	Atom-2	Interatomic distance (Å)	Clash overlap (Å)
1:B:218:ARG:HE	1:B:486:HIS:HD2[2_454]	1.31	0.29
1:B:455:LYS:NZ	5:B:935:HOH:O[3_454]	2.12	0.08

5.3 Torsion angles (i)

5.3.1 Protein backbone (i)

In the following table, the Percentiles column shows the percent Ramachandran outliers of the chain as a percentile score with respect to all X-ray entries followed by that with respect to entries of similar resolution.

The Analysed column shows the number of residues for which the backbone conformation was analysed, and the total number of residues.

Mol	Chain	Analysed	Favoured	Allowed	Outliers	Percentiles	
1	B	491/491 (100%)	484 (99%)	7 (1%)	0	100	100

There are no Ramachandran outliers to report.

5.3.2 Protein sidechains (i)

In the following table, the Percentiles column shows the percent sidechain outliers of the chain as a percentile score with respect to all X-ray entries followed by that with respect to entries of similar resolution.

The Analysed column shows the number of residues for which the sidechain conformation was analysed, and the total number of residues.

Mol	Chain	Analysed	Rotameric	Outliers	Percentiles	
1	B	407/405 (100%)	405 (100%)	2 (0%)	88	64

All (2) residues with a non-rotameric sidechain are listed below:

For Manuscript Review

Mol	Chain	Res	Type
1	B	294	THR
1	B	372	PHE

Sometimes sidechains can be flipped to improve hydrogen bonding and reduce clashes. All (2) such sidechains are listed below:

Mol	Chain	Res	Type
1	B	26	HIS
1	B	225	GLN

5.3.3 RNA ⓘ

There are no RNA molecules in this entry.

5.4 Non-standard residues in protein, DNA, RNA chains ⓘ

There are no non-standard protein/DNA/RNA residues in this entry.

5.5 Carbohydrates ⓘ

There are no monosaccharides in this entry.

5.6 Ligand geometry ⓘ

9 ligands are modelled in this entry.

In the following table, the Counts columns list the number of bonds (or angles) for which Mogul statistics could be retrieved, the number of bonds (or angles) that are observed in the model and the number of bonds (or angles) that are defined in the Chemical Component Dictionary. The Link column lists molecule types, if any, to which the group is linked. The Z score for a bond length (or angle) is the number of standard deviations the observed value is removed from the expected value. A bond length (or angle) with $|Z| > 2$ is considered an outlier worth inspection. RMSZ is the root-mean-square of all Z scores of the bond lengths (or angles).

Mol	Type	Chain	Res	Link	Bond lengths			Bond angles		
					Counts	RMSZ	$\#\|Z\| > 2$	Counts	RMSZ	$\#\|Z\| > 2$
2	PG4	B	501	-	10,10,12	0.20	0	9,9,11	0.54	0
3	SF4	B	506	1	0,12,12	-	-	-		
4	MHX	B	508	1	12,21,21	2.89	7 (58%)	2,42,42	0.95	0
2	PG4	B	504[A]	-	12,12,12	0.14	0	11,11,11	0.55	0
2	PG4	B	504[B]	-	12,12,12	0.15	0	11,11,11	0.49	0

For Manuscript Review

Mol	Type	Chain	Res	Link	Bond lengths			Bond angles		
					Counts	RMSZ	#|Z| > 2	Counts	RMSZ	#|Z| > 2
2	PG4	B	503	-	4,4,12	0.23	0	3,3,11	0.56	0
3	SF4	B	505	1	0,12,12	-	-	-		
2	PG4	B	502	-	12,12,12	0.24	0	11,11,11	1.24	2 (18%)
3	SF4	B	507	1	0,12,12	-	-	-		

In the following table, the Chirals column lists the number of chiral outliers, the number of chiral centers analysed, the number of these observed in the model and the number defined in the Chemical Component Dictionary. Similar counts are reported in the Torsion and Rings columns. '-' means no outliers of that kind were identified.

Mol	Type	Chain	Res	Link	Chirals	Torsions	Rings
2	PG4	B	501	-	-	1/8/8/10	-
3	SF4	B	506	1	-	-	0/6/5/5
4	MHX	B	508	1	-	-	0/5/3/3
2	PG4	B	504[A]	-	-	4/10/10/10	-
2	PG4	B	504[B]	-	-	3/10/10/10	-
2	PG4	B	503	-	-	0/2/2/10	-
3	SF4	B	505	1	-	-	0/6/5/5
2	PG4	B	502	-	-	5/10/10/10	-
3	SF4	B	507	1	-	-	0/6/5/5

All (7) bond length outliers are listed below:

Mol	Chain	Res	Type	Atoms	Z	Observed(Å)	Ideal(Å)
4	B	508	MHX	C2-S2	-5.41	1.75	1.85
4	B	508	MHX	O5-C5	4.76	1.25	1.17
4	B	508	MHX	C1-S1	-3.86	1.78	1.85
4	B	508	MHX	S2-FE1	2.85	2.30	2.26
4	B	508	MHX	O-C	2.51	1.20	1.14
4	B	508	MHX	S1-FE1	2.39	2.29	2.26
4	B	508	MHX	C6-FE2	-2.26	1.88	1.95

All (2) bond angle outliers are listed below:

Mol	Chain	Res	Type	Atoms	Z	Observed(°)	Ideal(°)
2	B	502	PG4	O3-C4-C3	-2.28	100.10	110.39
2	B	502	PG4	C5-O3-C4	2.16	122.63	113.29

There are no chirality outliers.

All (13) torsion outliers are listed below:

For Manuscript Review

Mol	Chain	Res	Type	Atoms
2	B	502	PG4	O2-C3-C4-O3
2	B	504[A]	PG4	O2-C3-C4-O3
2	B	504[A]	PG4	O3-C5-C6-O4
2	B	502	PG4	O1-C1-C2-O2
2	B	504[B]	PG4	O1-C1-C2-O2
2	B	504[A]	PG4	C5-C6-O4-C7
2	B	504[A]	PG4	C3-C4-O3-C5
2	B	502	PG4	C5-C6-O4-C7
2	B	504[B]	PG4	O4-C7-C8-O5
2	B	504[B]	PG4	C1-C2-O2-C3
2	B	502	PG4	O3-C5-C6-O4
2	B	501	PG4	C4-C3-O2-C2
2	B	502	PG4	C6-C5-O3-C4

There are no ring outliers.

4 monomers are involved in 11 short contacts:

Mol	Chain	Res	Type	Clashes	Symm-Clashes
3	B	506	SF4	1	0
4	B	508	MHX	1	0
2	B	503	PG4	4	0
2	B	502	PG4	6	0

The following is a two-dimensional graphical depiction of Mogul quality analysis of bond lengths, bond angles, torsion angles, and ring geometry for all instances of the Ligand of Interest. In addition, ligands with molecular weight > 250 and outliers as shown on the validation Tables will also be included. For torsion angles, if less then 5% of the Mogul distribution of torsion angles is within 10 degrees of the torsion angle in question, then that torsion angle is considered an outlier. Any bond that is central to one or more torsion angles identified as an outlier by Mogul will be highlighted in the graph. For rings, the root-mean-square deviation (RMSD) between the ring in question and similar rings identified by Mogul is calculated over all ring torsion angles. If the average RMSD is greater than 60 degrees and the minimal RMSD between the ring in question and any Mogul-identified rings is also greater than 60 degrees, then that ring is considered an outlier. The outliers are highlighted in purple. The color gray indicates Mogul did not find sufficient equivalents in the CSD to analyse the geometry.

For Manuscript Review

Ligand SF4 B 506

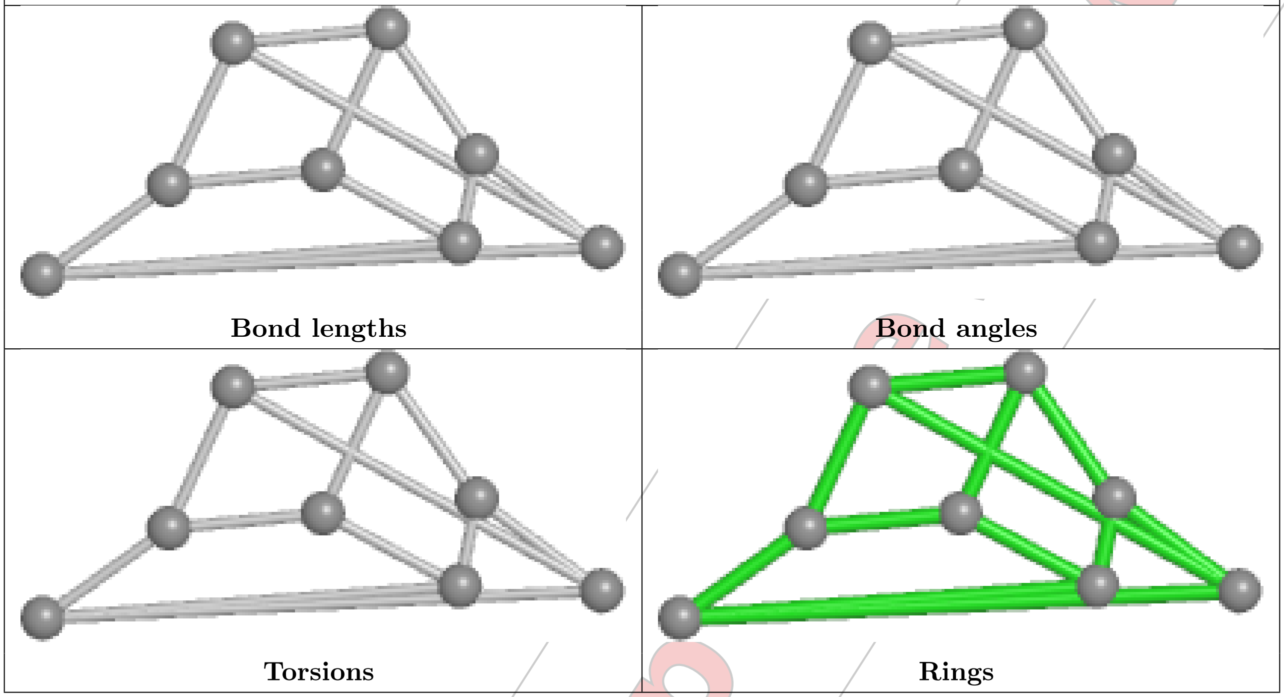

Ligand MHX B 508

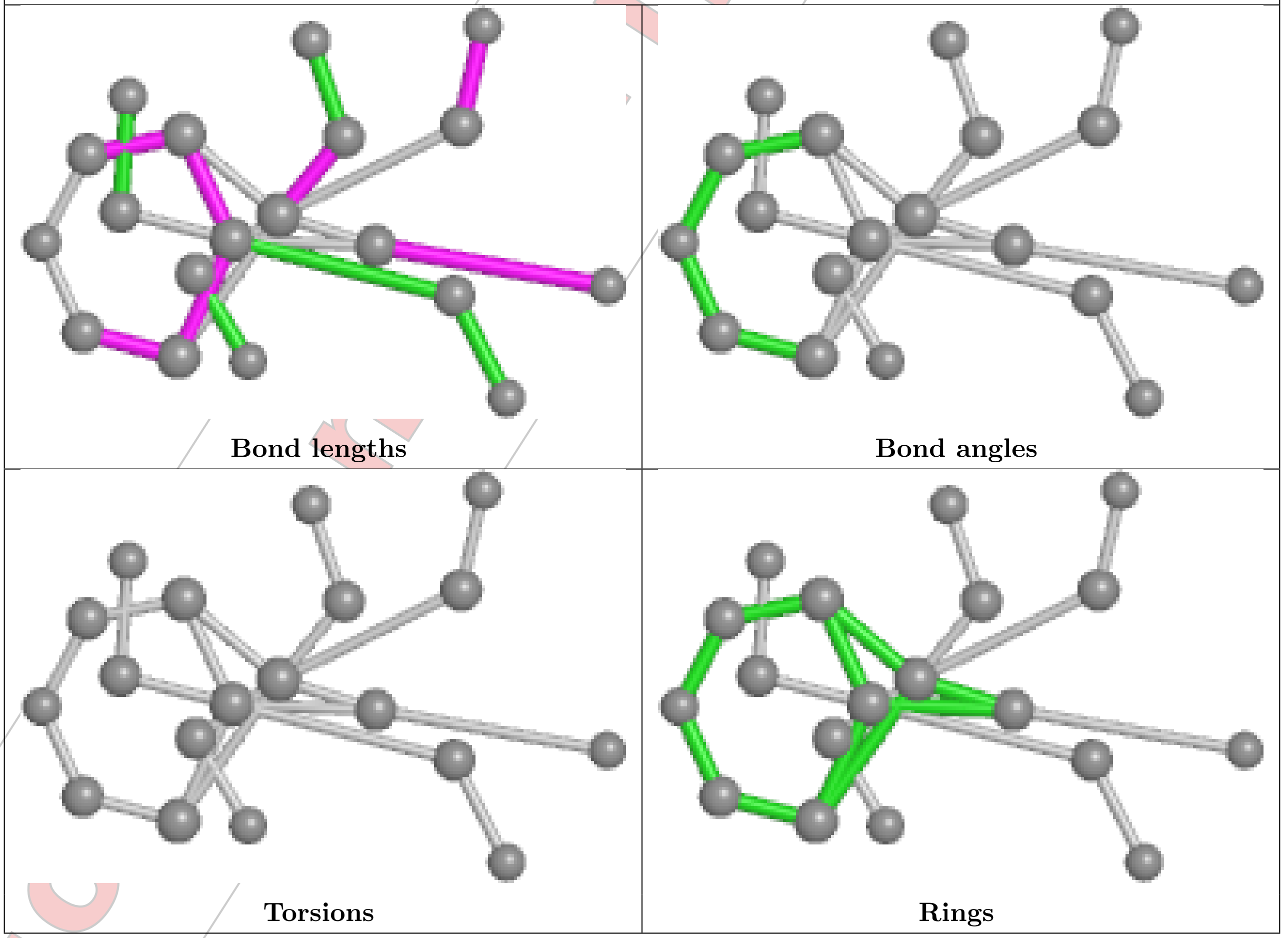

Ligand SF4 B 505

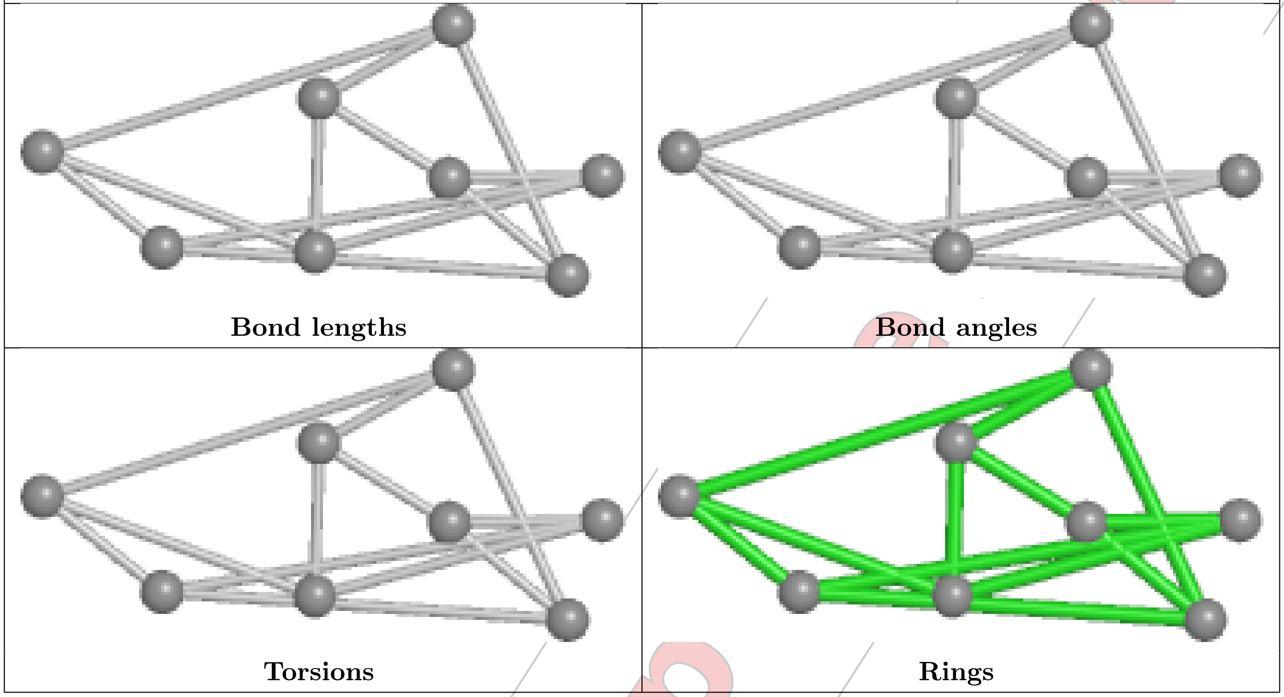

Ligand SF4 B 507

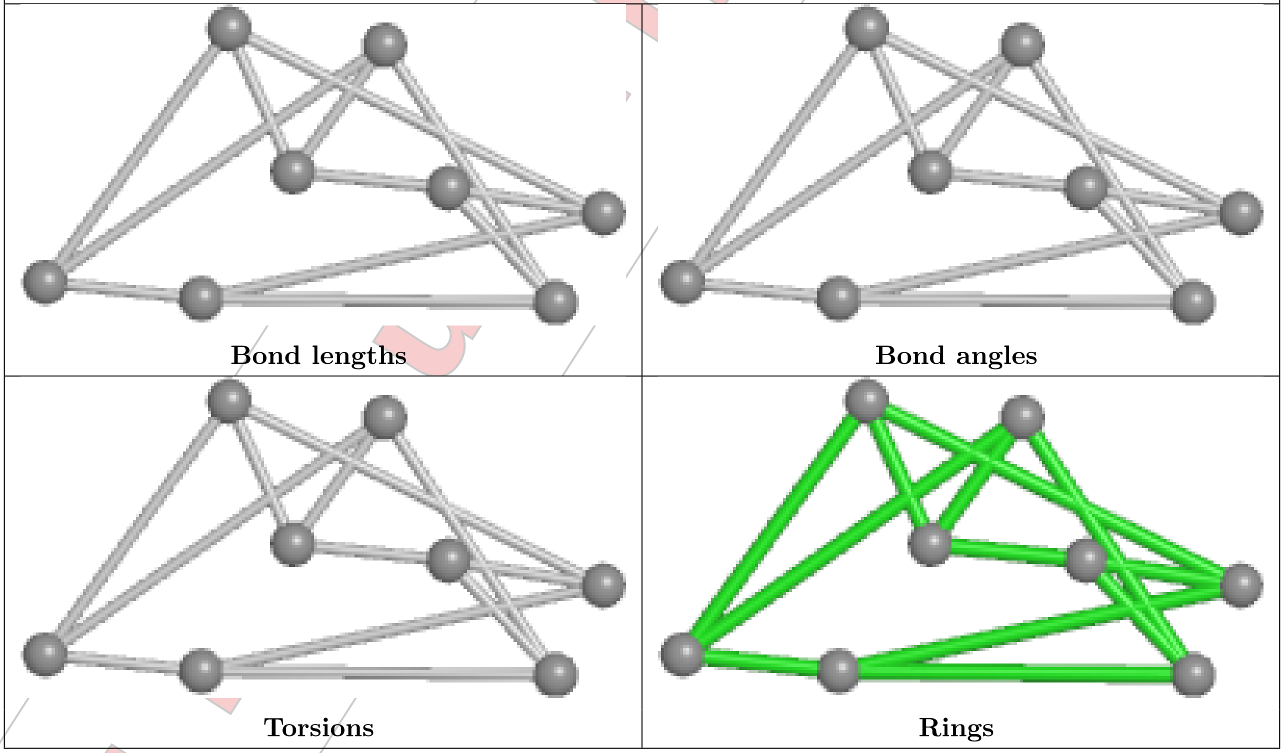

5.7 Other polymers (i)

There are no such residues in this entry.

5.8 Polymer linkage issues (i)

There are no chain breaks in this entry.

For Manuscript Review

6 Fit of model and data (i)

6.1 Protein, DNA and RNA chains (i)

In the following table, the column labelled '#RSRZ> 2' contains the number (and percentage) of RSRZ outliers, followed by percent RSRZ outliers for the chain as percentile scores relative to all X-ray entries and entries of similar resolution. The OWAB column contains the minimum, median, 95th percentile and maximum values of the occupancy-weighted average B-factor per residue. The column labelled 'Q< 0.9' lists the number of (and percentage) of residues with an average occupancy less than 0.9.

Mol	Chain	Analysed	<RSRZ>	#RSRZ>2			OWAB(Å^2)	Q<0.9
1	B	484/491 (98%)	-0.28	12 (2%)	57	56	8, 13, 23, 38	0

All (12) RSRZ outliers are listed below:

Mol	Chain	Res	Type	RSRZ
1	B	484	TRP	7.9
1	B	483	ALA	7.4
1	B	485	SER	5.2
1	B	486	HIS	4.2
1	B	395	LEU	3.6
1	B	482	SER	3.3
1	B	396	GLU	3.0
1	B	392	PRO	2.8
1	B	394	ASP	2.3
1	B	99	LYS	2.3
1	B	451	PHE	2.2
1	B	393	LYS	2.1

6.2 Non-standard residues in protein, DNA, RNA chains (i)

There are no non-standard protein/DNA/RNA residues in this entry.

6.3 Carbohydrates (i)

There are no monosaccharides in this entry.

6.4 Ligands (i)

In the following table, the Atoms column lists the number of modelled atoms in the group and the number defined in the chemical component dictionary. The B-factors column lists the minimum,

For Manuscript Review

median, 95th percentile and maximum values of B factors of atoms in the group. The column labelled 'Q< 0.9' lists the number of atoms with occupancy less than 0.9.

Mol	Type	Chain	Res	Atoms	RSCC	RSR	B-factors($Å^2$)	Q<0.9
2	PG4	B	503	5/13	0.77	0.21	32,36,39,41	1
2	PG4	B	502	13/13	0.90	0.13	26,34,38,40	1
2	PG4	B	504[A]	13/13	0.91	0.28	23,32,35,38	31
2	PG4	B	504[B]	13/13	0.91	0.28	18,26,30,32	31
2	PG4	B	501	11/13	0.94	0.14	19,24,28,30	1
3	SF4	B	505	8/8	1.00	0.05	9,9,10,10	0
3	SF4	B	506	8/8	1.00	0.05	8,8,8,8	0
3	SF4	B	507	8/8	1.00	0.03	13,14,15,16	0
4	MHX	B	508	19/19	1.00	0.07	8,9,11,11	2

The following is a graphical depiction of the model fit to experimental electron density of all instances of the Ligand of Interest. In addition, ligands with molecular weight > 250 and outliers as shown on the geometry validation Tables will also be included. Each fit is shown from different orientation to approximate a three-dimensional view.

For Manuscript Review

Electron density around SF4 B 505:

$2mF_o$-DF_c (at 0.7 rmsd) in gray
mF_o-DF_c (at 3 rmsd) in purple (negative)
and green (positive)

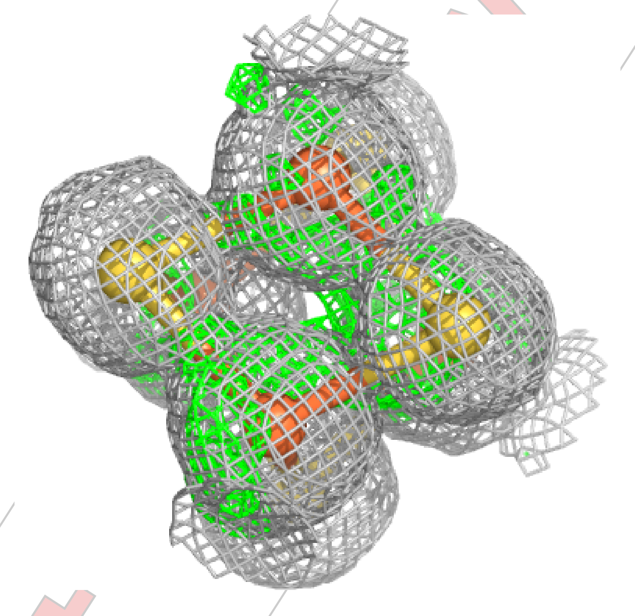

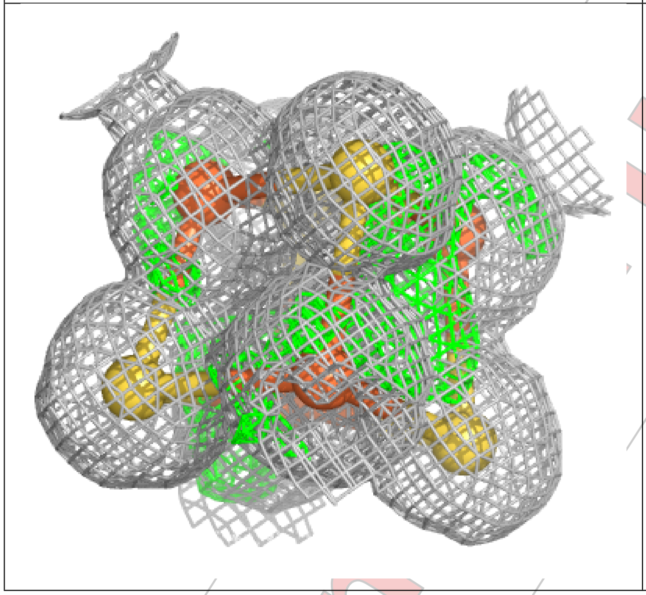

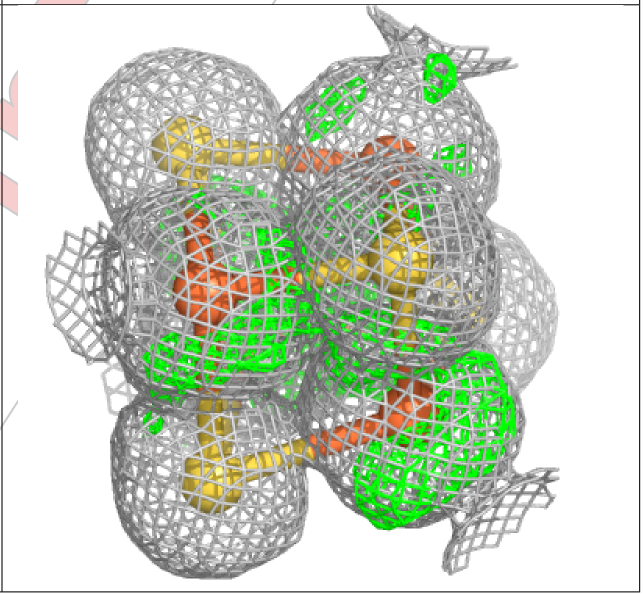

Electron density around SF4 B 506:

$2mF_o$-DF_c (at 0.7 rmsd) in gray
mF_o-DF_c (at 3 rmsd) in purple (negative)
and green (positive)

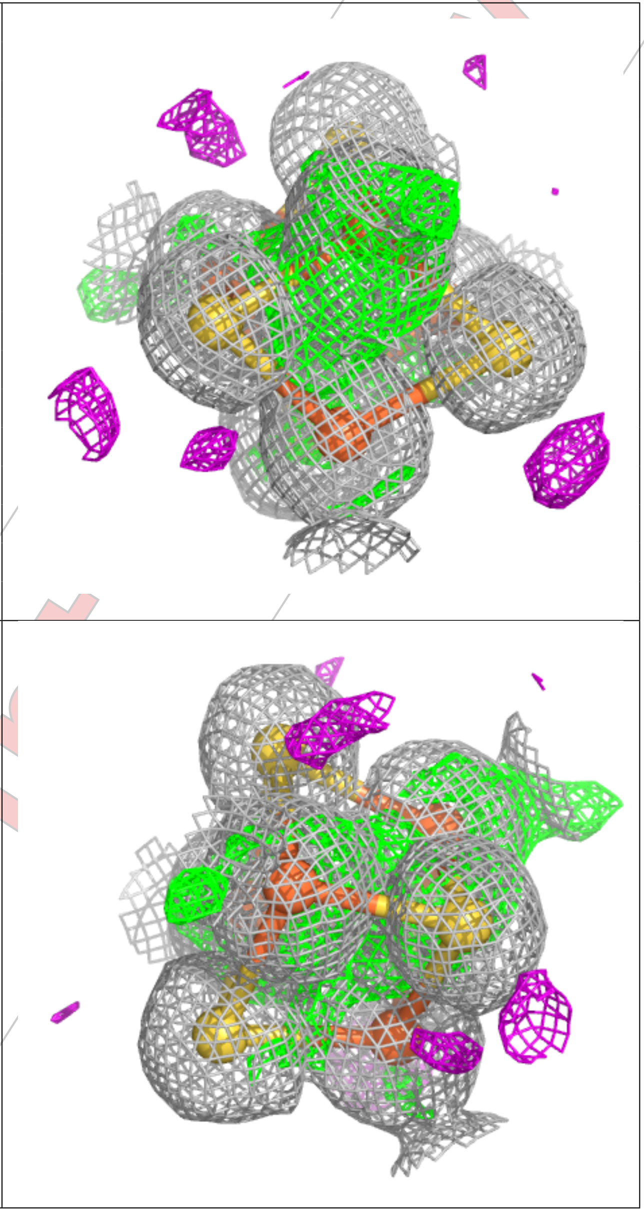

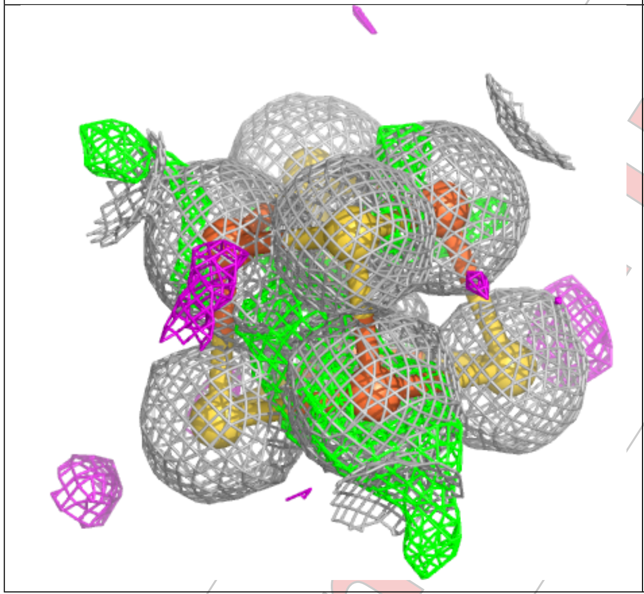

For Manuscript Review

Electron density around SF4 B 507:

2mF$_o$-DF$_c$ (at 0.7 rmsd) in gray
mF$_o$-DF$_c$ (at 3 rmsd) in purple (negative)
and green (positive)

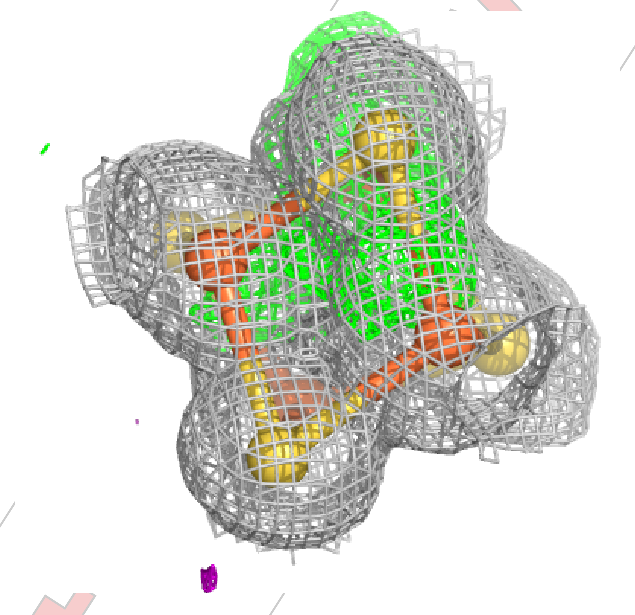

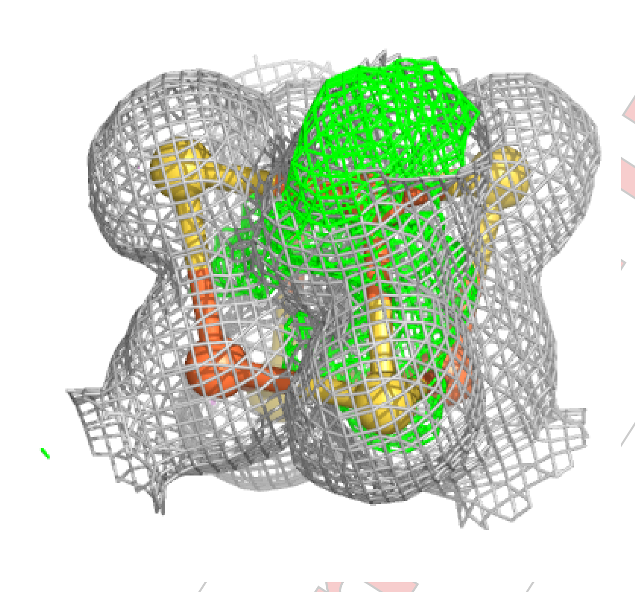

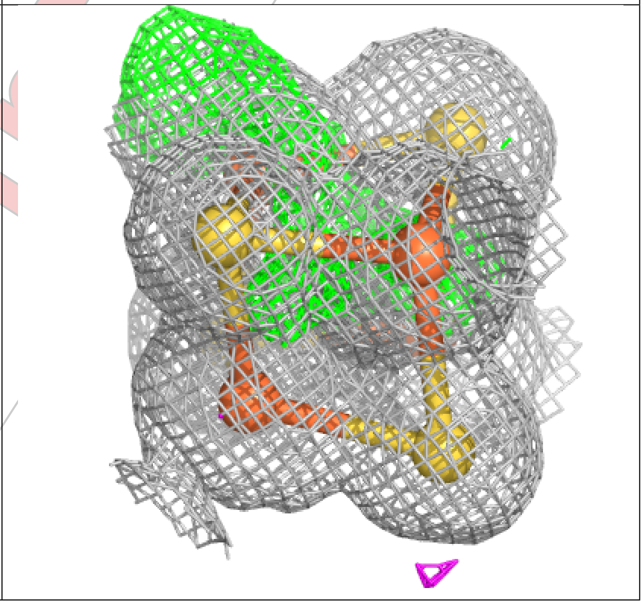

Electron density around MHX B 508:

$2mF_o$-DF_c (at 0.7 rmsd) in gray
mF_o-DF_c (at 3 rmsd) in purple (negative)
and green (positive)

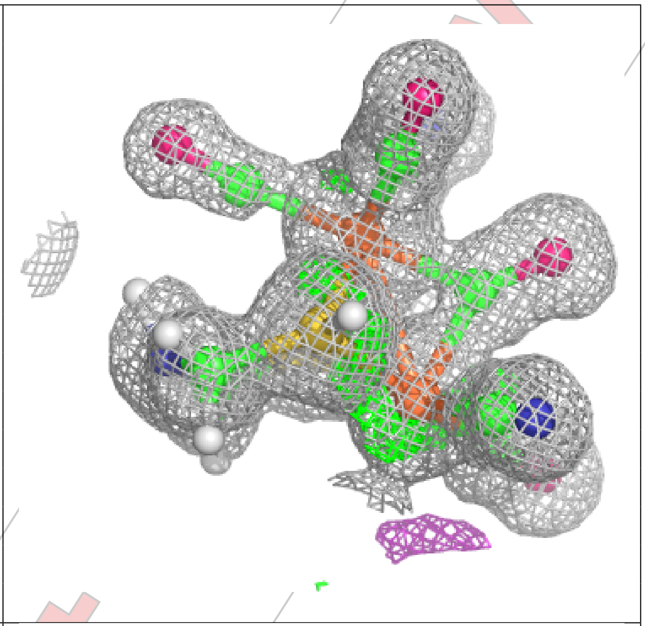

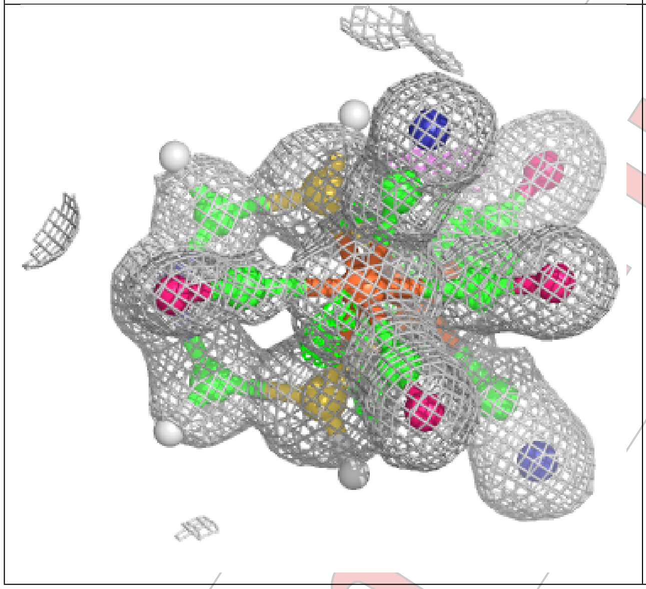

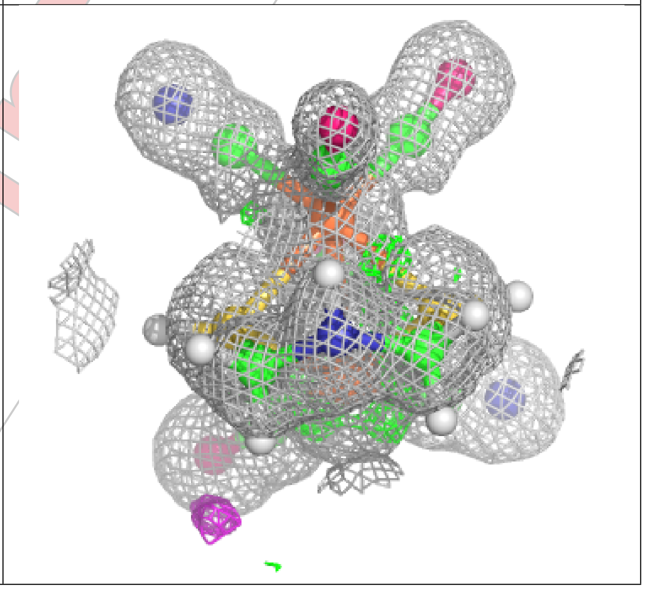

6.5 Other polymers (i)

There are no such residues in this entry.

For Manuscript Review

WORLDWIDE PDB PROTEIN DATA BANK

Full wwPDB X-ray Structure Validation Report (i)

Sep 3, 2024 – 03:54 pm BST

PDB ID	:	9GNK
Title	:	Desulfovibrio desulfuricans [FeFe]-hydrogenase variant with both subunits linked by a 13 amino acid linker peptide derived from CpI of Clostridium pasteurianum
Deposited on	:	2024-09-03
Resolution	:	1.05 Å(reported)

This wwPDB validation report is for manuscript review

For Manuscript Review

This is a Full wwPDB X-ray Structure Validation Report.

This report is produced by the wwPDB biocuration pipeline after annotation of the structure.

We welcome your comments at *validation@mail.wwpdb.org*
A user guide is available at
https://www.wwpdb.org/validation/2017/XrayValidationReportHelp
with specific help available everywhere you see the (i) symbol.

The types of validation reports are described at
http://www.wwpdb.org/validation/2017/FAQs#types.

The following versions of software and data (see references (i)) were used in the production of this report:

MolProbity	:	4.02b-467
Mogul	:	1.8.4, CSD as541be (2020)
Xtriage (Phenix)	:	1.13
EDS	:	3.0
buster-report	:	1.1.7 (2018)
Percentile statistics	:	20231227.v01 (using entries in the PDB archive December 27th 2023)
CCP4	:	9.0.002 (Gargrove)
Density-Fitness	:	1.0.11

1 Overall quality at a glance (i)

The following experimental techniques were used to determine the structure:
X-RAY DIFFRACTION

The reported resolution of this entry is 1.05 Å.

Percentile scores (ranging between 0-100) for global validation metrics of the entry are shown in the following graphic. The table shows the number of entries on which the scores are based.

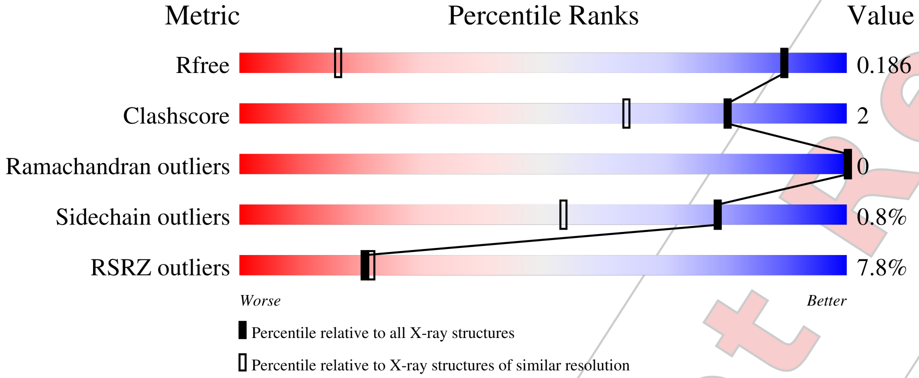

Metric	Whole archive (#Entries)	Similar resolution (#Entries, resolution range(Å))
R_{free}	164625	1027 (1.08-1.04)
Clashscore	180529	1152 (1.08-1.04)
Ramachandran outliers	177936	1127 (1.08-1.04)
Sidechain outliers	177891	1128 (1.08-1.04)
RSRZ outliers	164620	1026 (1.08-1.04)

The table below summarises the geometric issues observed across the polymeric chains and their fit to the electron density. The red, orange, yellow and green segments of the lower bar indicate the fraction of residues that contain outliers for >=3, 2, 1 and 0 types of geometric quality criteria respectively. A grey segment represents the fraction of residues that are not modelled. The numeric value for each fraction is indicated below the corresponding segment, with a dot representing fractions <=5% The upper red bar (where present) indicates the fraction of residues that have poor fit to the electron density. The numeric value is given above the bar.

Mol	Chain	Length	Quality of chain
1	A	492	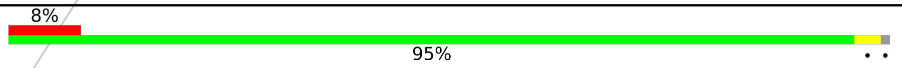

Ideal geometry (proteins) : Engh & Huber (2001)
Ideal geometry (DNA, RNA) : Parkinson et al. (1996)
Validation Pipeline (wwPDB-VP) : 2.38.2

For Manuscript Review

2 Entry composition (i)

There are 8 unique types of molecules in this entry. The entry contains 8357 atoms, of which 3886 are hydrogens and 0 are deuteriums.

In the tables below, the ZeroOcc column contains the number of atoms modelled with zero occupancy, the AltConf column contains the number of residues with at least one atom in alternate conformation and the Trace column contains the number of residues modelled with at most 2 atoms.

- Molecule 1 is a protein called Periplasmic [Fe] hydrogenase large subunit,Periplasmic [Fe] hydrogenase small subunit.

Mol	Chain	Residues	Atoms						ZeroOcc	AltConf	Trace
			Total	C	H	N	O	S			
1	A	485	7584	2424	3788	627	709	36	128	14	0

There are 23 discrepancies between the modelled and reference sequences:

Chain	Residue	Modelled	Actual	Comment	Reference
A	391	VAL	-	linker	UNP P07598
A	392	ASN	-	linker	UNP P07598
A	393	PRO	-	linker	UNP P07598
A	394	LYS	-	linker	UNP P07598
A	395	ASP	-	linker	UNP P07598
A	396	LEU	-	linker	UNP P07598
A	397	GLU	-	linker	UNP P07598
A	398	LYS	-	linker	UNP P07598
A	399	VAL	-	linker	UNP P07598
A	400	ASP	-	linker	UNP P07598
A	401	ILE	-	linker	UNP P07598
A	402	LYS	-	linker	UNP P07598
A	403	LYS	-	linker	UNP P07598
A	483	SER	-	expression tag	UNP P07603
A	484	ALA	-	expression tag	UNP P07603
A	485	TRP	-	expression tag	UNP P07603
A	486	SER	-	expression tag	UNP P07603
A	487	HIS	-	expression tag	UNP P07603
A	488	PRO	-	expression tag	UNP P07603
A	489	GLN	-	expression tag	UNP P07603
A	490	PHE	-	expression tag	UNP P07603
A	491	GLU	-	expression tag	UNP P07603
A	492	LYS	-	expression tag	UNP P07603

- Molecule 2 is CARBON MONOXIDE (three-letter code: CMO) (formula: CO) (labeled as "Ligand of Interest" by depositor).

For Manuscript Review

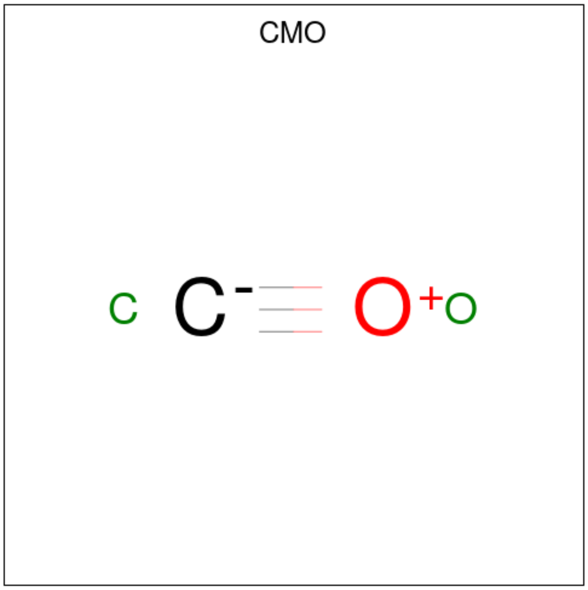

Mol	Chain	Residues	Atoms			ZeroOcc	AltConf
			Total	C	O		
2	A	1	2	1	1	0	0

- Molecule 3 is dicarbonyl[bis(cyanide-kappaC)]-mu-(iminodimethanethiolatato-1kappaS:2kappaS)-mu-(oxomethylidene)diiron(2+) (three-letter code: 402) (formula: $C_7H_5Fe_2N_3O_3S_2$) (labeled as "Ligand of Interest" by depositor).

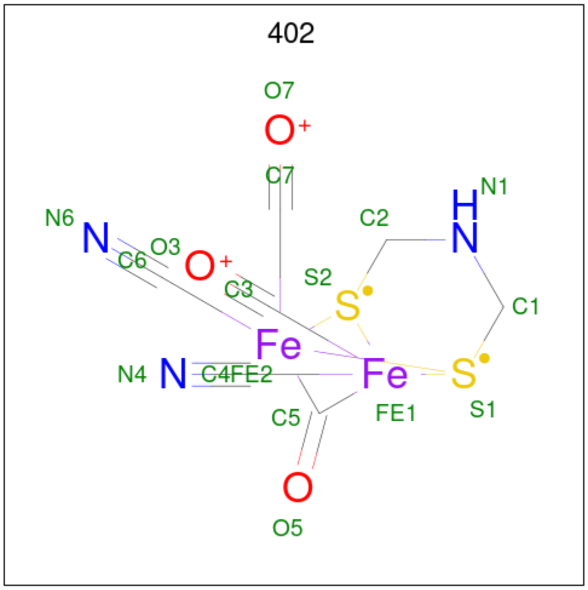

Mol	Chain	Residues	Atoms							ZeroOcc	AltConf
			Total	C	Fe	H	N	O	S		
3	A	1	24	7	2	7	3	3	2	1	0

- Molecule 4 is IRON/SULFUR CLUSTER (three-letter code: SF4) (formula: Fe_4S_4) (labeled

For Manuscript Review

as "Ligand of Interest" by depositor).

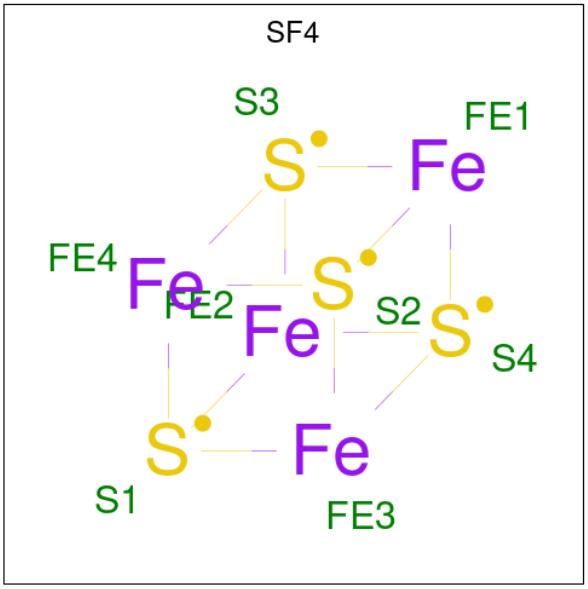

Mol	Chain	Residues	Atoms	ZeroOcc	AltConf
4	A	1	Total Fe S<br>8 4 4	0	0
4	A	1	Total Fe S<br>8 4 4	0	0
4	A	1	Total Fe S<br>8 4 4	0	0

- Molecule 5 is 4-(2-HYDROXYETHYL)-1-PIPERAZINE ETHANESULFONIC ACID (three-letter code: EPE) (formula: $C_8H_{18}N_2O_4S$).

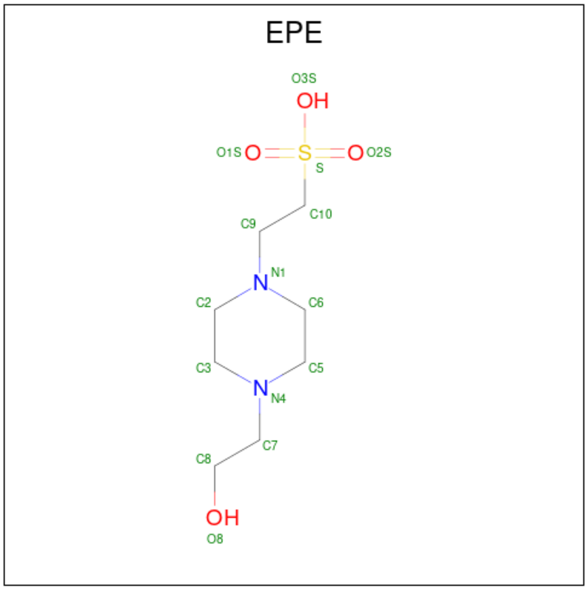

For Manuscript Review

Mol	Chain	Residues	Atoms						ZeroOcc	AltConf
			Total	C	H	N	O	S		
5	A	1	33	8	18	2	4	1	2	0

- Molecule 6 is TETRAETHYLENE GLYCOL (three-letter code: PG4) (formula: $C_8H_{18}O_5$).

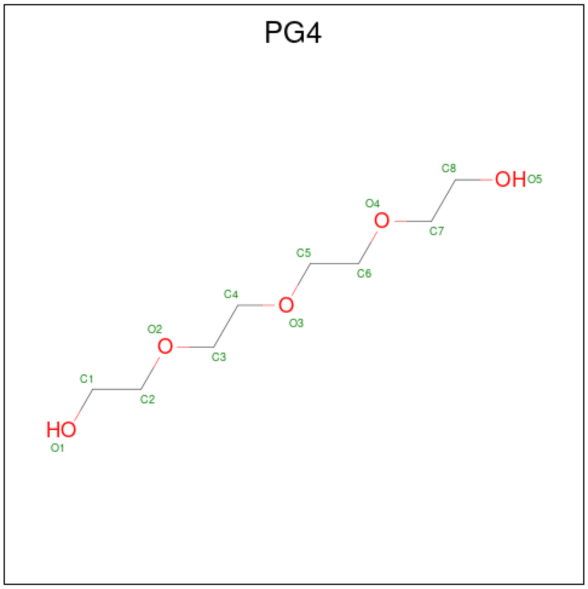

Mol	Chain	Residues	Atoms				ZeroOcc	AltConf
			Total	C	H	O		
6	A	1	31	8	18	5	2	0
6	A	1	31	8	18	5	2	0
6	A	1	17	5	10	2	4	0

- Molecule 7 is DI(HYDROXYETHYL)ETHER (three-letter code: PEG) (formula: $C_4H_{10}O_3$).

For Manuscript Review

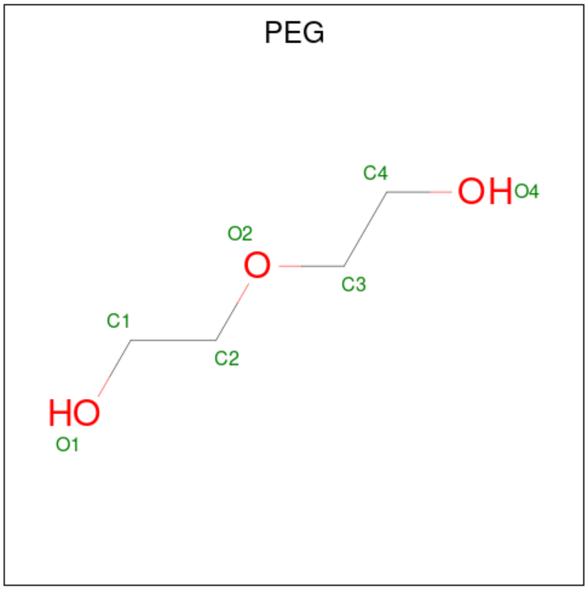

Mol	Chain	Residues	Atoms	ZeroOcc	AltConf
7	A	1	Total C H O<br>17 4 10 3	2	0
7	A	1	Total C H O<br>12 3 7 2	3	0
7	A	1	Total C H O<br>17 4 10 3	2	0

- Molecule 8 is water.

Mol	Chain	Residues	Atoms	ZeroOcc	AltConf
8	A	543	Total O<br>565 565	0	22

For Manuscript Review

3 Residue-property plots (i)

These plots are drawn for all protein, RNA, DNA and oligosaccharide chains in the entry. The first graphic for a chain summarises the proportions of the various outlier classes displayed in the second graphic. The second graphic shows the sequence view annotated by issues in geometry and electron density. Residues are color-coded according to the number of geometric quality criteria for which they contain at least one outlier: green = 0, yellow = 1, orange = 2 and red = 3 or more. A red dot above a residue indicates a poor fit to the electron density (RSRZ > 2). Stretches of 2 or more consecutive residues without any outlier are shown as a green connector. Residues present in the sample, but not in the model, are shown in grey.

• Molecule 1: Periplasmic [Fe] hydrogenase large subunit,Periplasmic [Fe] hydrogenase small subunit

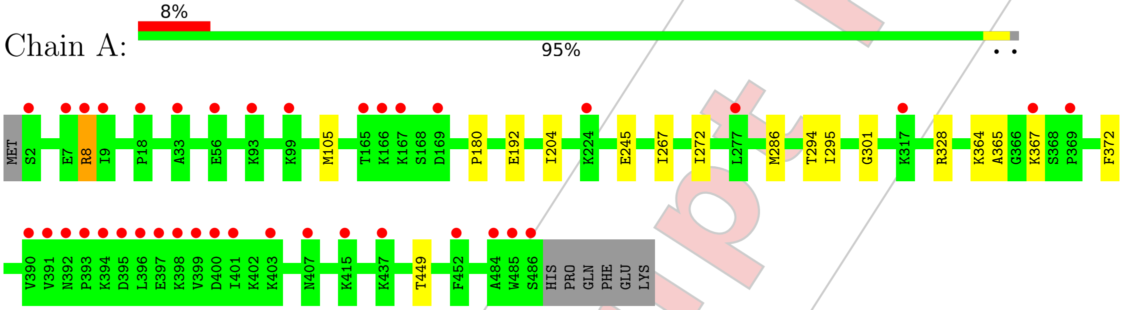

For Manuscript Review

4 Data and refinement statistics (i)

Property	Value	Source
Space group	P 21 21 21	Depositor
Cell constants<br>a, b, c, α, β, γ	49.14Å 89.21Å 106.45Å<br>90.00° 90.00° 90.00°	Depositor
Resolution (Å)	45.75 – 1.05<br>45.75 – 1.05	Depositor<br>EDS
% Data completeness<br>(in resolution range)	99.8 (45.75-1.05)<br>99.8 (45.75-1.05)	Depositor<br>EDS
R_{merge}	0.06	Depositor
R_{sym}	(Not available)	Depositor
$< I/\sigma(I) >$ [1]	1.41 (at 1.05Å)	Xtriage
Refinement program	REFMAC 5.8.0425	Depositor
R, R_{free}	0.168 , 0.185<br>0.169 , 0.186	Depositor<br>DCC
R_{free} test set	10877 reflections (5.01%)	wwPDB-VP
Wilson B-factor (Å^2)	11.3	Xtriage
Anisotropy	0.122	Xtriage
Bulk solvent k_{sol}(e/Å^3), B_{sol}(Å^2)	0.39 , 37.5	EDS
L-test for twinning[2]	$< \|L\| > = 0.49$, $< L^2 > = 0.32$	Xtriage
Estimated twinning fraction	No twinning to report.	Xtriage
F_o,F_c correlation	0.97	EDS
Total number of atoms	8357	wwPDB-VP
Average B, all atoms (Å^2)	13.0	wwPDB-VP

Xtriage's analysis on translational NCS is as follows: *The largest off-origin peak in the Patterson function is 4.53% of the height of the origin peak. No significant pseudotranslation is detected.*

[1] Intensities estimated from amplitudes.

[2] Theoretical values of $< |L| >$, $< L^2 >$ for acentric reflections are 0.5, 0.333 respectively for untwinned datasets, and 0.375, 0.2 for perfectly twinned datasets.

For Manuscript Review

5 Model quality (i)

5.1 Standard geometry (i)

Bond lengths and bond angles in the following residue types are not validated in this section: CMO, PG4, EPE, PEG, 402, SF4

The Z score for a bond length (or angle) is the number of standard deviations the observed value is removed from the expected value. A bond length (or angle) with $|Z| > 5$ is considered an outlier worth inspection. RMSZ is the root-mean-square of all Z scores of the bond lengths (or angles).

Mol	Chain	Bond lengths		Bond angles	
		RMSZ	#$\|Z\|$ >5	RMSZ	#$\|Z\|$ >5
1	A	0.30	0/3934	0.62	2/5319 (0.0%)

Chiral center outliers are detected by calculating the chiral volume of a chiral center and verifying if the center is modelled as a planar moiety or with the opposite hand.A planarity outlier is detected by checking planarity of atoms in a peptide group, atoms in a mainchain group or atoms of a sidechain that are expected to be planar.

Mol	Chain	#Chirality outliers	#Planarity outliers
1	A	0	1

There are no bond length outliers.

All (2) bond angle outliers are listed below:

Mol	Chain	Res	Type	Atoms	Z	Observed(^{o})	Ideal(^{o})
1	A	286[A]	MET	CG-SD-CE	-5.78	90.95	100.20
1	A	286[B]	MET	CG-SD-CE	-5.78	90.95	100.20

There are no chirality outliers.

All (1) planarity outliers are listed below:

Mol	Chain	Res	Type	Group
1	A	8	ARG	Sidechain

5.2 Too-close contacts (i)

In the following table, the Non-H and H(model) columns list the number of non-hydrogen atoms and hydrogen atoms in the chain respectively. The H(added) column lists the number of hydrogen atoms added and optimized by MolProbity. The Clashes column lists the number of clashes within the asymmetric unit, whereas Symm-Clashes lists symmetry-related clashes.

For Manuscript Review

Mol	Chain	Non-H	H(model)	H(added)	Clashes	Symm-Clashes
1	A	3796	3788	3748	18	0
2	A	2	0	0	1	0
3	A	17	7	5	1	0
4	A	24	0	0	1	0
5	A	15	18	18	0	0
6	A	33	46	42	11	0
7	A	19	27	25	1	0
8	A	565	0	0	1	0
All	All	4471	3886	3838	19	0

The all-atom clashscore is defined as the number of clashes found per 1000 atoms (including hydrogen atoms). The all-atom clashscore for this structure is 2.

All (19) close contacts within the same asymmetric unit are listed below, sorted by their clash magnitude.

Atom-1	Atom-2	Interatomic distance (Å)	Clash overlap (Å)
1:A:328:ARG:HB2	7:A:510:PEG:H31	1.80	0.63
1:A:245:GLU:HB2	6:A:512:PG4:C4	2.32	0.60
1:A:449:THR:HA	6:A:512:PG4:H31	1.83	0.60
1:A:245:GLU:HG3	6:A:512:PG4:H42	1.83	0.60
1:A:367:LYS:HD3	8:A:602:HOH:O	2.03	0.58
1:A:245:GLU:CG	6:A:512:PG4:H42	2.36	0.55
1:A:245:GLU:HB2	6:A:512:PG4:H42	1.90	0.53
1:A:364[A]:LYS:HG2	6:A:507:PG4:H71	1.91	0.52
1:A:364[A]:LYS:CG	6:A:507:PG4:H71	2.41	0.51
1:A:245:GLU:HB2	6:A:512:PG4:H41	1.93	0.51
1:A:192[A]:GLU:OE2	1:A:364[A]:LYS:HD2	2.12	0.50
1:A:105[B]:MET:HE3	1:A:204:ILE:HD11	1.93	0.50
2:A:501:CMO:C	3:A:502:402:N1	2.76	0.49
1:A:295:ILE:O	1:A:301:GLY:HA3	2.14	0.48
1:A:180:PRO:HD2	4:A:503:SF4:S3	2.58	0.44
1:A:267:ILE:HG23	1:A:272:ILE:HB	1.99	0.44
1:A:365:ALA:N	6:A:507:PG4:H72	2.33	0.43
1:A:364[A]:LYS:CD	6:A:507:PG4:H71	2.49	0.43
1:A:364[A]:LYS:HG2	6:A:507:PG4:C7	2.49	0.41

There are no symmetry-related clashes.

For Manuscript Review

5.3 Torsion angles (i)

5.3.1 Protein backbone (i)

In the following table, the Percentiles column shows the percent Ramachandran outliers of the chain as a percentile score with respect to all X-ray entries followed by that with respect to entries of similar resolution.

The Analysed column shows the number of residues for which the backbone conformation was analysed, and the total number of residues.

Mol	Chain	Analysed	Favoured	Allowed	Outliers	Percentiles	
1	A	497/492 (101%)	483 (97%)	14 (3%)	0	100	100

There are no Ramachandran outliers to report.

5.3.2 Protein sidechains (i)

In the following table, the Percentiles column shows the percent sidechain outliers of the chain as a percentile score with respect to all X-ray entries followed by that with respect to entries of similar resolution.

The Analysed column shows the number of residues for which the sidechain conformation was analysed, and the total number of residues.

Mol	Chain	Analysed	Rotameric	Outliers	Percentiles	
1	A	406/406 (100%)	403 (99%)	3 (1%)	81	60

All (3) residues with a non-rotameric sidechain are listed below:

Mol	Chain	Res	Type
1	A	8	ARG
1	A	294	THR
1	A	372	PHE

Sometimes sidechains can be flipped to improve hydrogen bonding and reduce clashes. All (1) such sidechains are listed below:

Mol	Chain	Res	Type
1	A	71	GLN

5.3.3 RNA (i)

There are no RNA molecules in this entry.

For Manuscript Review

For Manuscript Review

5.4 Non-standard residues in protein, DNA, RNA chains (i)

There are no non-standard protein/DNA/RNA residues in this entry.

5.5 Carbohydrates (i)

There are no oligosaccharides in this entry.

5.6 Ligand geometry (i)

12 ligands are modelled in this entry.

In the following table, the Counts columns list the number of bonds (or angles) for which Mogul statistics could be retrieved, the number of bonds (or angles) that are observed in the model and the number of bonds (or angles) that are defined in the Chemical Component Dictionary. The Link column lists molecule types, if any, to which the group is linked. The Z score for a bond length (or angle) is the number of standard deviations the observed value is removed from the expected value. A bond length (or angle) with $|Z| > 2$ is considered an outlier worth inspection. RMSZ is the root-mean-square of all Z scores of the bond lengths (or angles).

Mol	Type	Chain	Res	Link	Bond lengths			Bond angles		
					Counts	RMSZ	$\#\|Z\| > 2$	Counts	RMSZ	$\#\|Z\| > 2$
4	SF4	A	504	1	0,12,12	-	-	-		
6	PG4	A	511	-	12,12,12	0.15	0	11,11,11	0.43	0
6	PG4	A	512	-	6,6,12	0.20	0	5,5,11	0.49	0
7	PEG	A	508	-	6,6,6	0.12	0	5,5,5	0.10	0
4	SF4	A	503	1	0,12,12	-	-	-		
5	EPE	A	506	-	15,15,15	0.64	1 (6%)	18,20,20	0.76	1 (5%)
4	SF4	A	505	1	0,12,12	-	-	-		
3	402	A	502	1	13,19,19	4.37	7 (53%)	2,36,36	0.56	0
7	PEG	A	510	-	6,6,6	0.14	0	5,5,5	0.10	0
2	CMO	A	501	-	0,1,1	-	-	-		
7	PEG	A	509	-	4,4,6	0.23	0	3,3,5	0.20	0
6	PG4	A	507	-	12,12,12	0.15	0	11,11,11	0.42	0

In the following table, the Chirals column lists the number of chiral outliers, the number of chiral centers analysed, the number of these observed in the model and the number defined in the Chemical Component Dictionary. Similar counts are reported in the Torsion and Rings columns. '-' means no outliers of that kind were identified.

Mol	Type	Chain	Res	Link	Chirals	Torsions	Rings
6	PG4	A	512	-	-	3/4/4/10	-
6	PG4	A	511	-	-	6/10/10/10	-

Continued on next page...

Continued from previous page...

Mol	Type	Chain	Res	Link	Chirals	Torsions	Rings
7	PEG	A	508	-	-	0/4/4/4	-
4	SF4	A	504	1	-	-	0/6/5/5
5	EPE	A	506	-	-	3/9/19/19	0/1/1/1
4	SF4	A	503	1	-	-	0/6/5/5
4	SF4	A	505	1	-	-	0/6/5/5
7	PEG	A	510	-	-	3/4/4/4	-
3	402	A	502	1	-	-	0/5/3/3
7	PEG	A	509	-	-	1/2/2/4	-
6	PG4	A	507	-	-	5/10/10/10	-

All (8) bond length outliers are listed below:

Mol	Chain	Res	Type	Atoms	Z	Observed(Å)	Ideal(Å)
3	A	502	402	C2-S2	-10.08	1.67	1.85
3	A	502	402	C1-S1	-9.39	1.68	1.85
3	A	502	402	S1-FE2	-4.38	2.20	2.26
3	A	502	402	O5-C5	3.67	1.24	1.17
3	A	502	402	S1-FE1	-3.16	2.21	2.26
3	A	502	402	S2-FE1	-2.55	2.22	2.26
5	A	506	EPE	O3S-S	2.21	1.55	1.47
3	A	502	402	S2-FE2	-2.06	2.23	2.26

All (1) bond angle outliers are listed below:

Mol	Chain	Res	Type	Atoms	Z	Observed(°)	Ideal(°)
5	A	506	EPE	O3S-S-C10	-2.06	102.44	105.77

There are no chirality outliers.

All (21) torsion outliers are listed below:

Mol	Chain	Res	Type	Atoms
6	A	511	PG4	O2-C3-C4-O3
6	A	507	PG4	O2-C3-C4-O3
7	A	510	PEG	O2-C3-C4-O4
6	A	507	PG4	O4-C7-C8-O5
5	A	506	EPE	C8-C7-N4-C5
6	A	511	PG4	O1-C1-C2-O2
7	A	509	PEG	O2-C3-C4-O4
7	A	510	PEG	O1-C1-C2-O2
5	A	506	EPE	N4-C7-C8-O8

Continued on next page...

For Manuscript Review

Continued from previous page...

Mol	Chain	Res	Type	Atoms
5	A	506	EPE	S-C10-C9-N1
6	A	512	PG4	C3-C4-O3-C5
6	A	507	PG4	C8-C7-O4-C6
6	A	511	PG4	C5-C6-O4-C7
6	A	511	PG4	C1-C2-O2-C3
6	A	507	PG4	C3-C4-O3-C5
6	A	512	PG4	C1-C2-O2-C3
6	A	512	PG4	O2-C3-C4-O3
7	A	510	PEG	C4-C3-O2-C2
6	A	507	PG4	O3-C5-C6-O4
6	A	511	PG4	O3-C5-C6-O4
6	A	511	PG4	C8-C7-O4-C6

There are no ring outliers.

6 monomers are involved in 14 short contacts:

Mol	Chain	Res	Type	Clashes	Symm-Clashes
6	A	512	PG4	6	0
4	A	503	SF4	1	0
3	A	502	402	1	0
7	A	510	PEG	1	0
2	A	501	CMO	1	0
6	A	507	PG4	5	0

The following is a two-dimensional graphical depiction of Mogul quality analysis of bond lengths, bond angles, torsion angles, and ring geometry for all instances of the Ligand of Interest. In addition, ligands with molecular weight > 250 and outliers as shown on the validation Tables will also be included. For torsion angles, if less then 5% of the Mogul distribution of torsion angles is within 10 degrees of the torsion angle in question, then that torsion angle is considered an outlier. Any bond that is central to one or more torsion angles identified as an outlier by Mogul will be highlighted in the graph. For rings, the root-mean-square deviation (RMSD) between the ring in question and similar rings identified by Mogul is calculated over all ring torsion angles. If the average RMSD is greater than 60 degrees and the minimal RMSD between the ring in question and any Mogul-identified rings is also greater than 60 degrees, then that ring is considered an outlier. The outliers are highlighted in purple. The color gray indicates Mogul did not find sufficient equivalents in the CSD to analyse the geometry.

For Manuscript Review

Ligand SF4 A 504

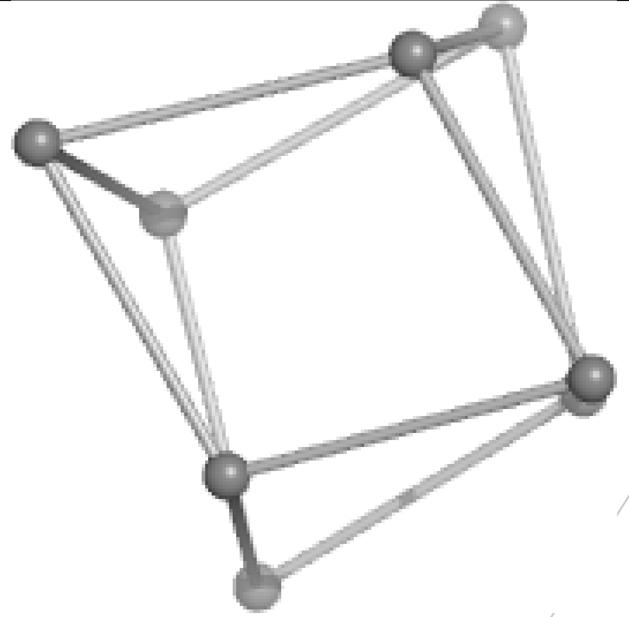

Bond lengths

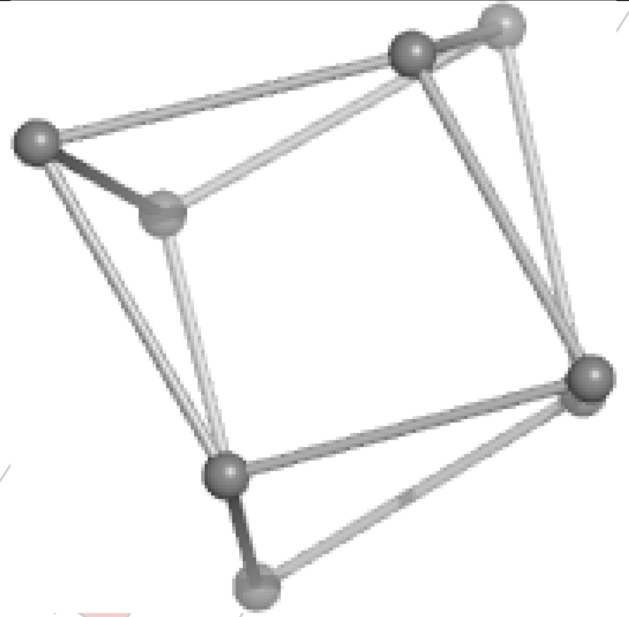

Bond angles

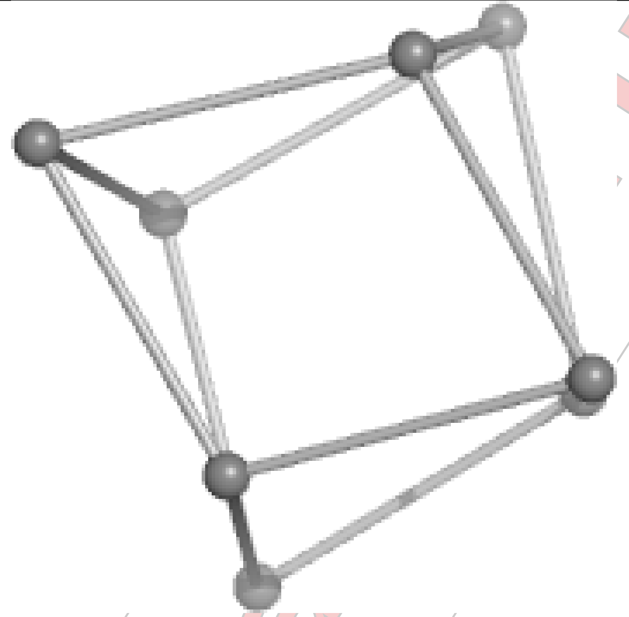

Torsions

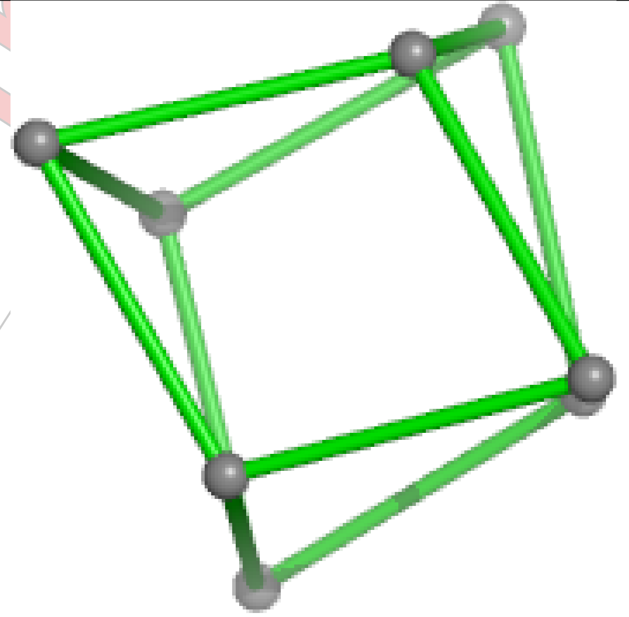

Rings

For Manuscript Review

Ligand SF4 A 503

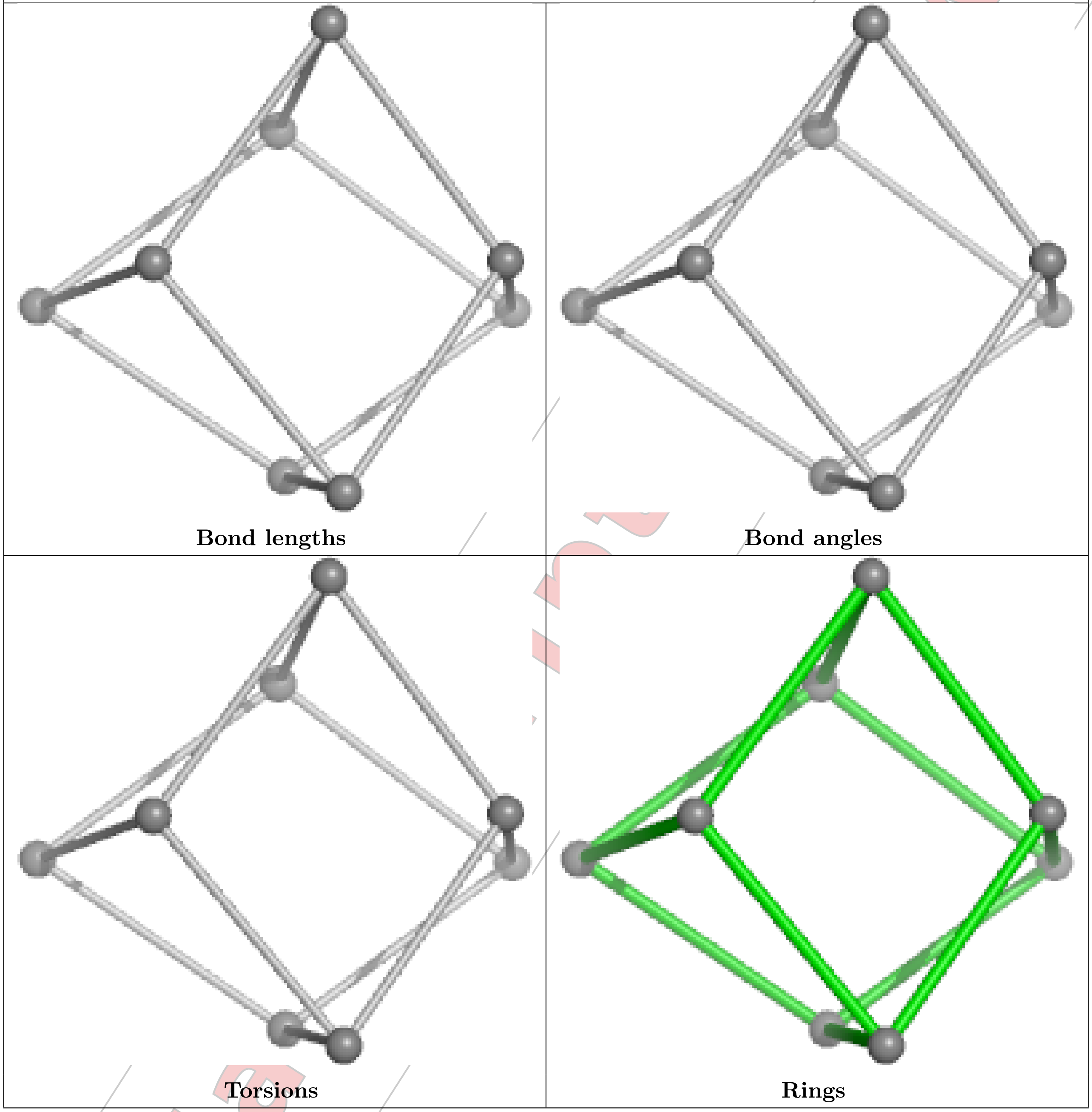

For Manuscript Review

Ligand SF4 A 505

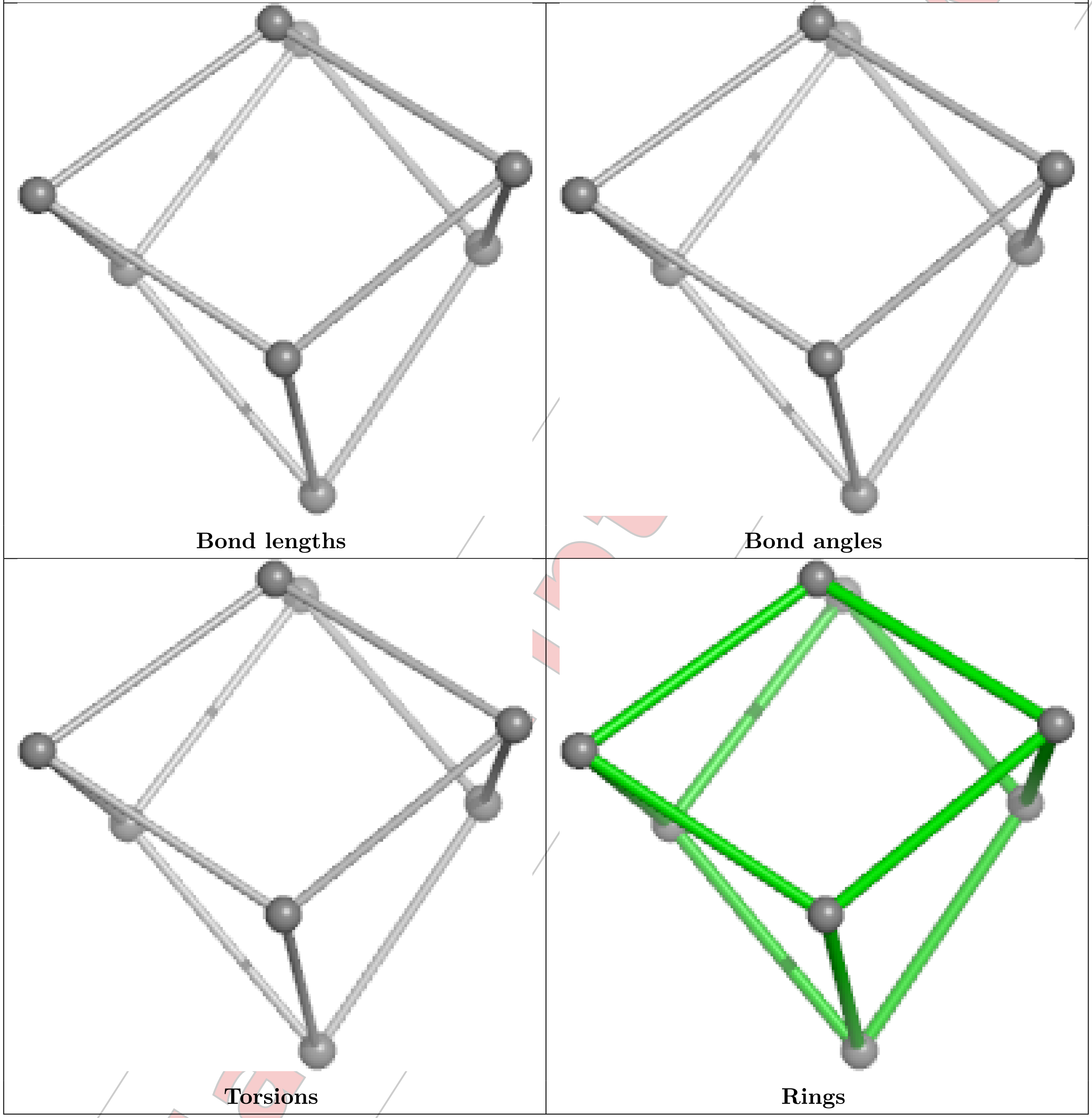

For Manuscript Review

Ligand 402 A 502

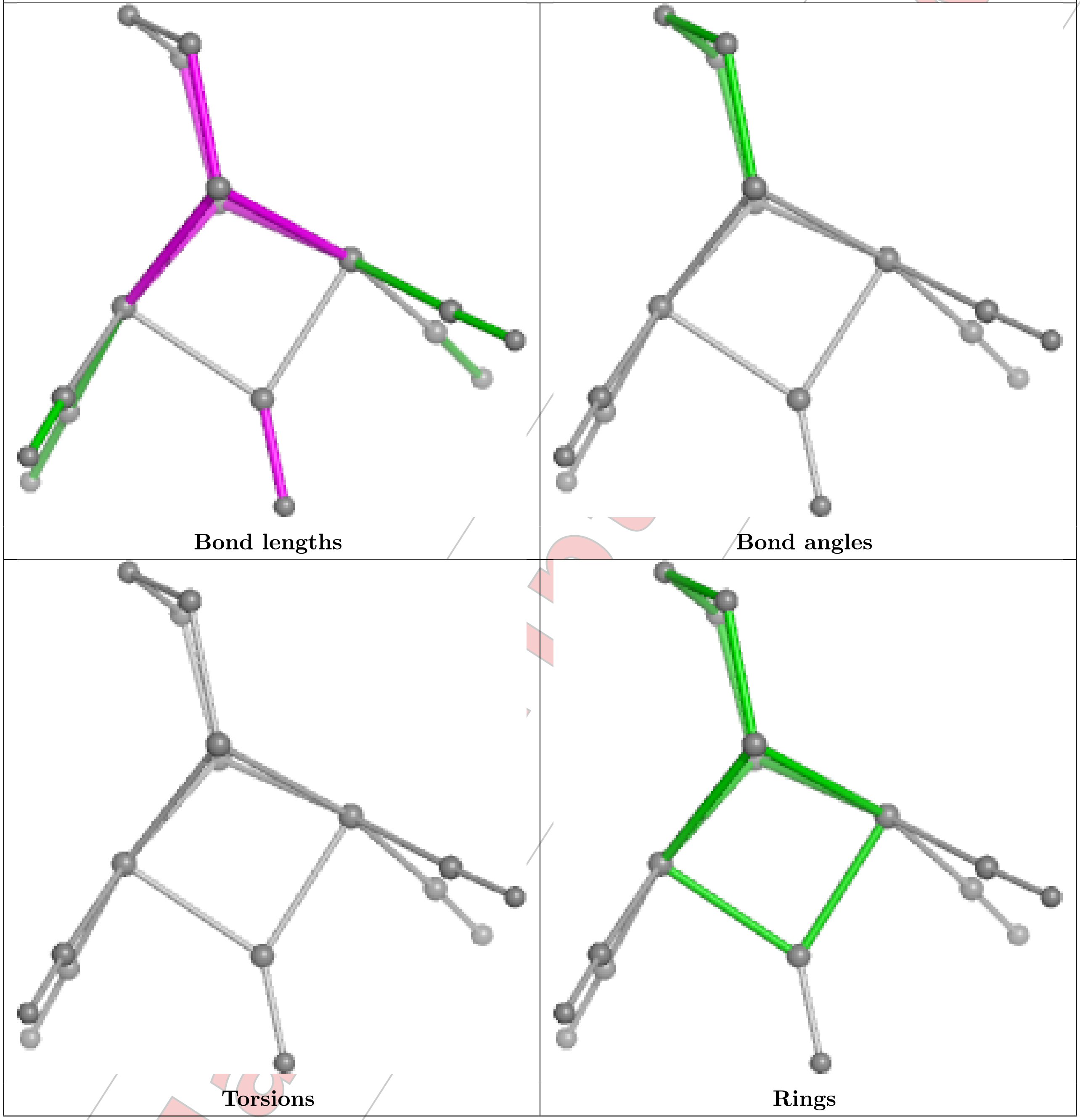

Ligand CMO A 501

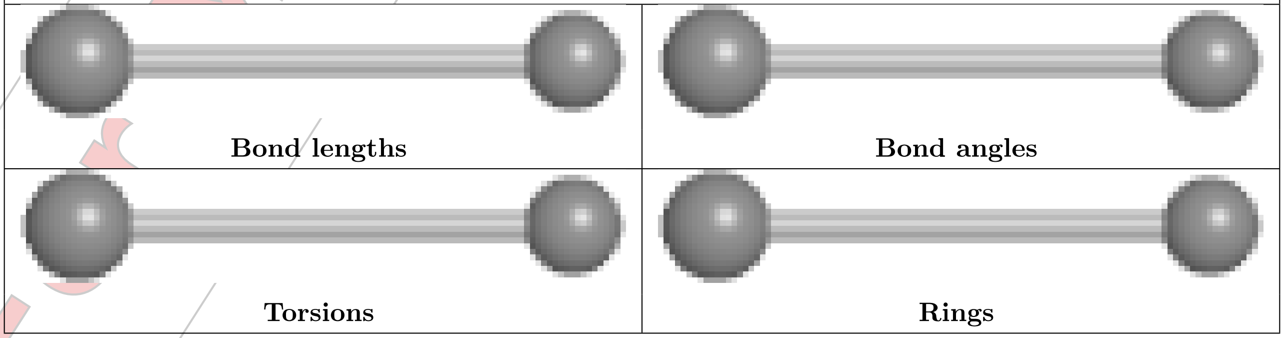

5.7 Other polymers (i)

There are no such residues in this entry.

5.8 Polymer linkage issues (i)

There are no chain breaks in this entry.

For Manuscript Review

6 Fit of model and data (i)

6.1 Protein, DNA and RNA chains (i)

In the following table, the column labelled '#RSRZ> 2' contains the number (and percentage) of RSRZ outliers, followed by percent RSRZ outliers for the chain as percentile scores relative to all X-ray entries and entries of similar resolution. The OWAB column contains the minimum, median, 95th percentile and maximum values of the occupancy-weighted average B-factor per residue. The column labelled 'Q< 0.9' lists the number of (and percentage) of residues with an average occupancy less than 0.9.

Mol	Chain	Analysed	<RSRZ>	#RSRZ>2			OWAB(Å^2)	Q<0.9
1	A	485/492 (98%)	0.61	38 (7%)	20	22	5, 12, 18, 43	14 (2%)

All (38) RSRZ outliers are listed below:

Mol	Chain	Res	Type	RSRZ
1	A	399	VAL	8.3
1	A	2	SER	7.6
1	A	392	ASN	7.3
1	A	391	VAL	7.1
1	A	393	PRO	5.7
1	A	486	SER	5.6
1	A	394	LYS	5.1
1	A	8	ARG	5.1
1	A	396	LEU	4.6
1	A	398	LYS	4.5
1	A	390	VAL	3.9
1	A	484	ALA	3.8
1	A	403	LYS	3.7
1	A	485	TRP	3.7
1	A	395	ASP	3.3
1	A	397	GLU	3.2
1	A	317	LYS	3.1
1	A	369	PRO	3.1
1	A	407	ASN	2.8
1	A	400	ASP	2.7
1	A	401	ILE	2.7
1	A	166	LYS	2.6
1	A	224	LYS	2.6
1	A	367	LYS	2.5
1	A	7	GLU	2.4
1	A	452	PHE	2.4
1	A	9	ILE	2.4

Continued on next page...

For Manuscript Review

Continued from previous page...

Mol	Chain	Res	Type	RSRZ
1	A	93	LYS	2.3
1	A	167	LYS	2.3
1	A	169	ASP	2.3
1	A	18	PRO	2.2
1	A	277	LEU	2.2
1	A	437	LYS	2.2
1	A	99	LYS	2.1
1	A	165	THR	2.1
1	A	415	LYS	2.0
1	A	56	GLU	2.0
1	A	33	ALA	2.0

6.2 Non-standard residues in protein, DNA, RNA chains (i)

There are no non-standard protein/DNA/RNA residues in this entry.

6.3 Carbohydrates (i)

There are no monosaccharides in this entry.

6.4 Ligands (i)

In the following table, the Atoms column lists the number of modelled atoms in the group and the number defined in the chemical component dictionary. The B-factors column lists the minimum, median, 95th percentile and maximum values of B factors of atoms in the group. The column labelled 'Q< 0.9' lists the number of atoms with occupancy less than 0.9.

Mol	Type	Chain	Res	Atoms	RSCC	RSR	B-factors(Å^2)	Q<0.9
6	PG4	A	511	13/13	0.58	0.17	24,28,32,44	2
7	PEG	A	510	7/7	0.63	0.18	28,28,29,29	2
6	PG4	A	507	13/13	0.72	0.15	24,27,29,30	2
6	PG4	A	512	7/13	0.75	0.18	19,19,20,20	17
7	PEG	A	509	5/7	0.80	0.15	15,17,18,18	12
5	EPE	A	506	15/15	0.80	0.18	27,29,29,29	33
7	PEG	A	508	7/7	0.84	0.12	24,25,26,26	2
2	CMO	A	501	2/2	0.97	0.12	9,9,9,10	0
4	SF4	A	505	8/8	0.99	0.04	12,12,13,13	0
3	402	A	502	17/17	0.99	0.07	8,8,8,9	1
4	SF4	A	503	8/8	1.00	0.01	7,7,8,8	0
4	SF4	A	504	8/8	1.00	0.02	9,9,9,9	0

For Manuscript Review

The following is a graphical depiction of the model fit to experimental electron density of all instances of the Ligand of Interest. In addition, ligands with molecular weight > 250 and outliers as shown on the geometry validation Tables will also be included. Each fit is shown from different orientation to approximate a three-dimensional view.

Electron density around CMO A 501:

$2mF_o$-DF_c (at 0.7 rmsd) in gray
mF_o-DF_c (at 3 rmsd) in purple (negative)
and green (positive)

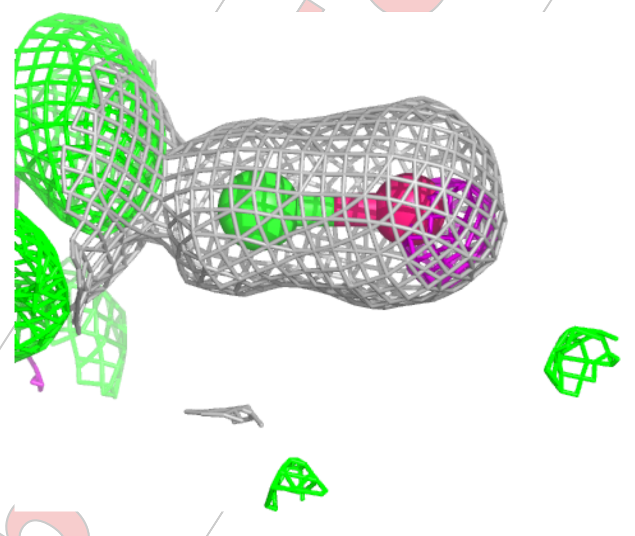

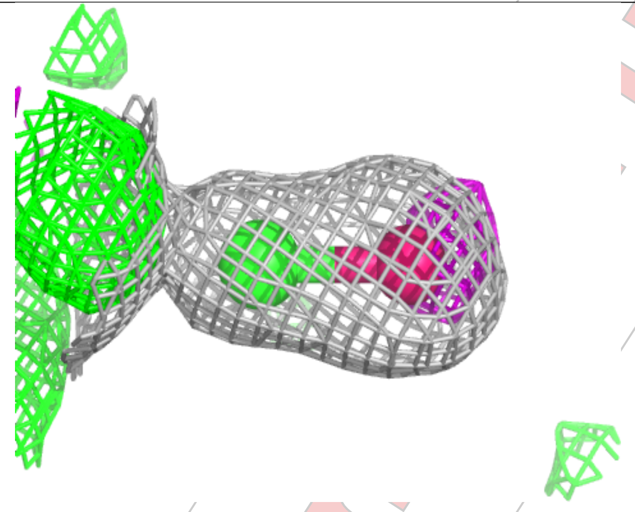

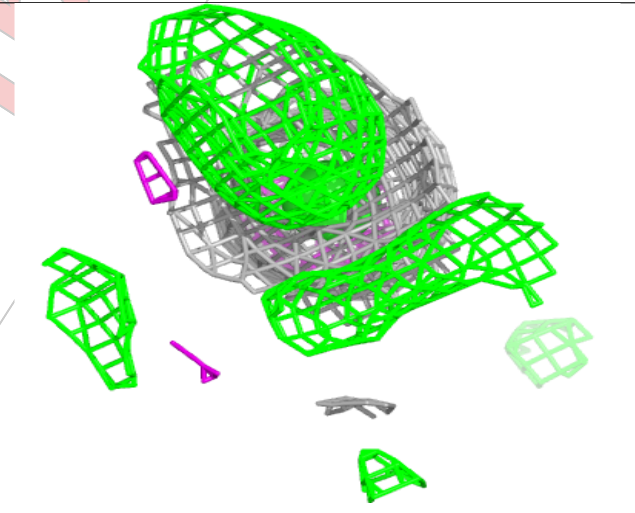

For Manuscript Review

Electron density around SF4 A 505:

$2mF_o$-DF_c (at 0.7 rmsd) in gray
mF_o-DF_c (at 3 rmsd) in purple (negative)
and green (positive)

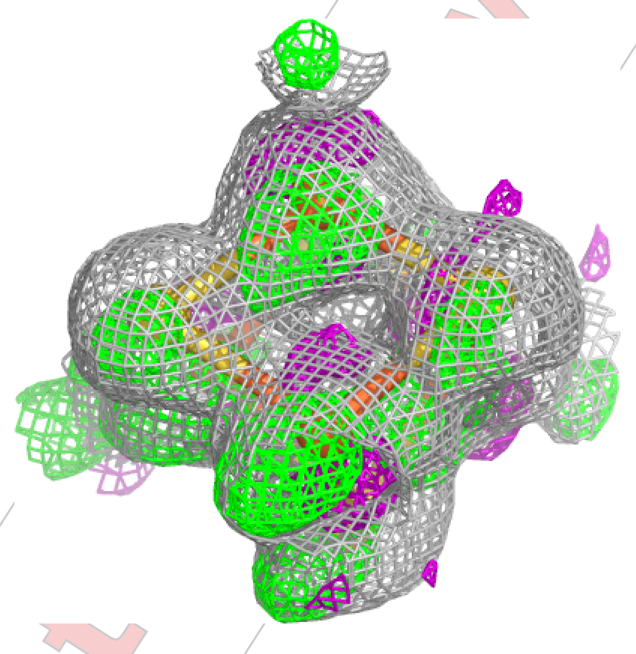

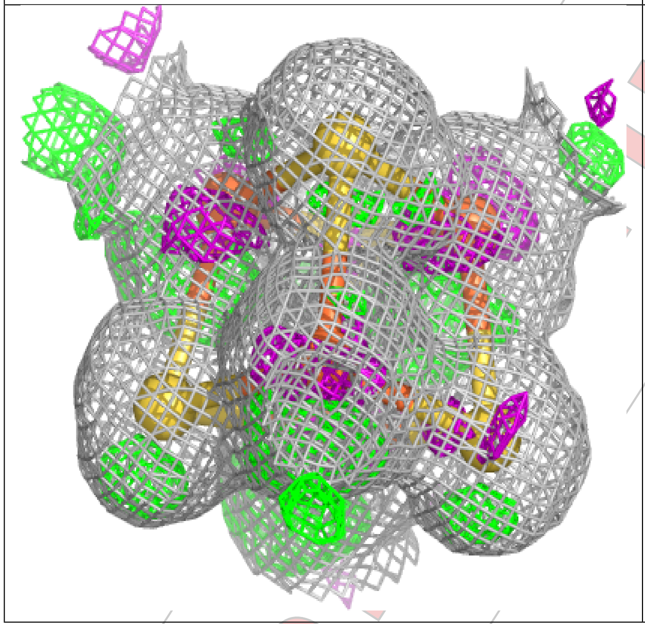

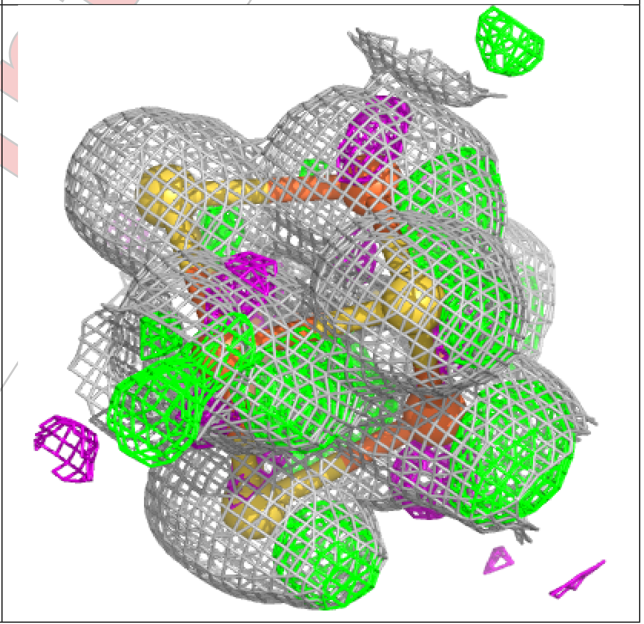

For Manuscript Review

Electron density around 402 A 502:

$2mF_o$-DF_c (at 0.7 rmsd) in gray
mF_o-DF_c (at 3 rmsd) in purple (negative)
and green (positive)

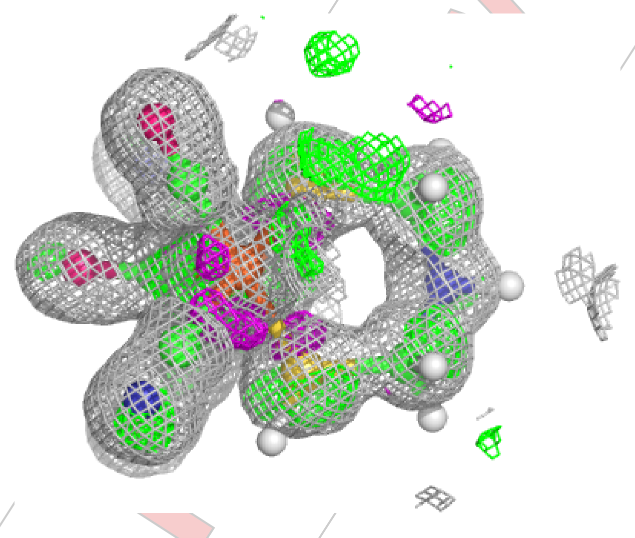

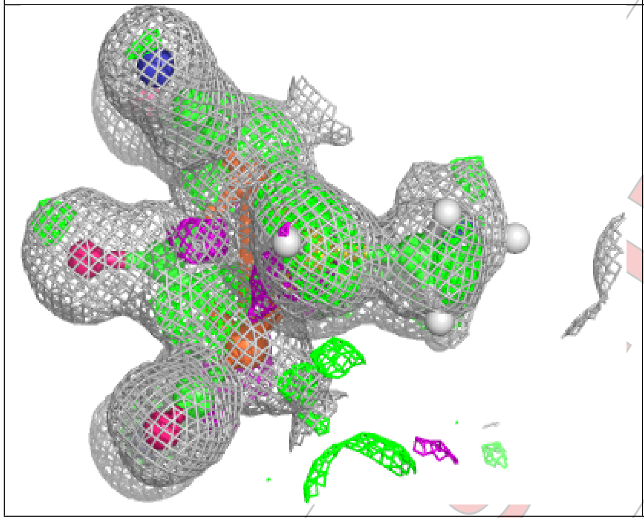

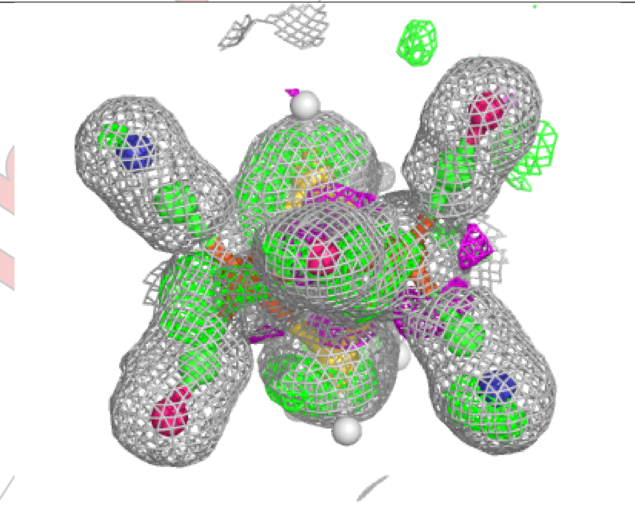

For Manuscript Review

Electron density around SF4 A 503:

2mF$_o$-DF$_c$ (at 0.7 rmsd) in gray
mF$_o$-DF$_c$ (at 3 rmsd) in purple (negative)
and green (positive)

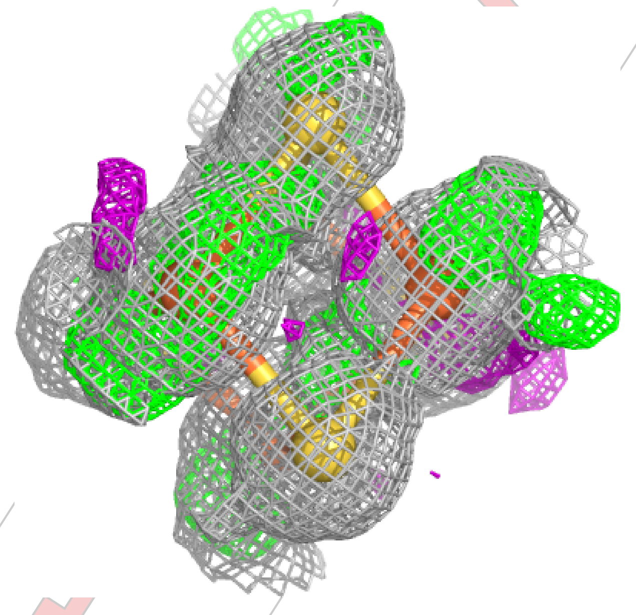

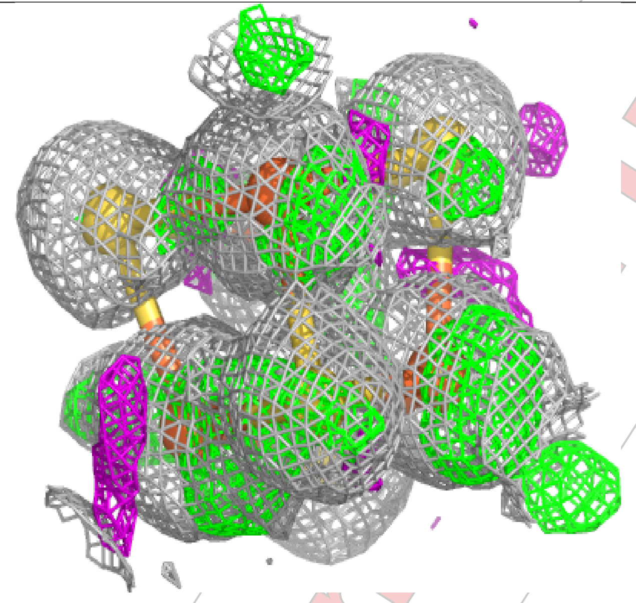

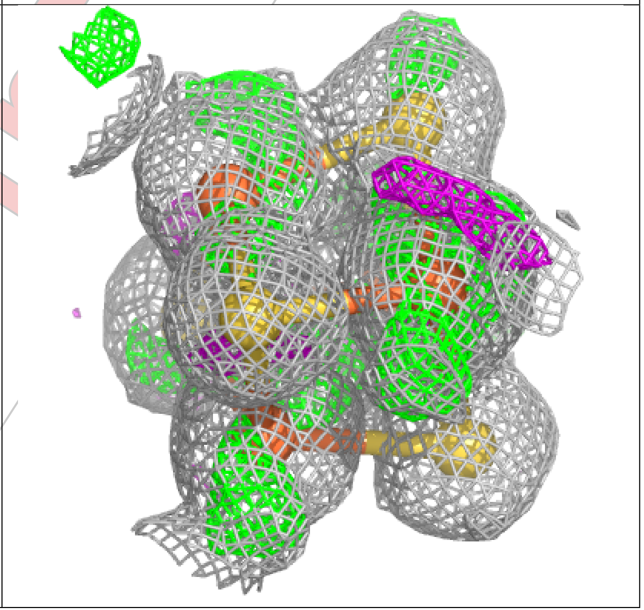

For Manuscript Review

Electron density around SF4 A 504:

$2mF_o$-DF_c (at 0.7 rmsd) in gray
mF_o-DF_c (at 3 rmsd) in purple (negative)
and green (positive)

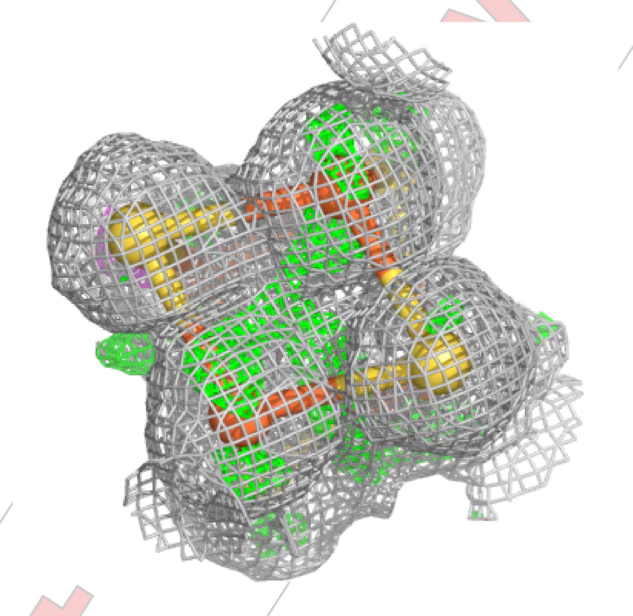

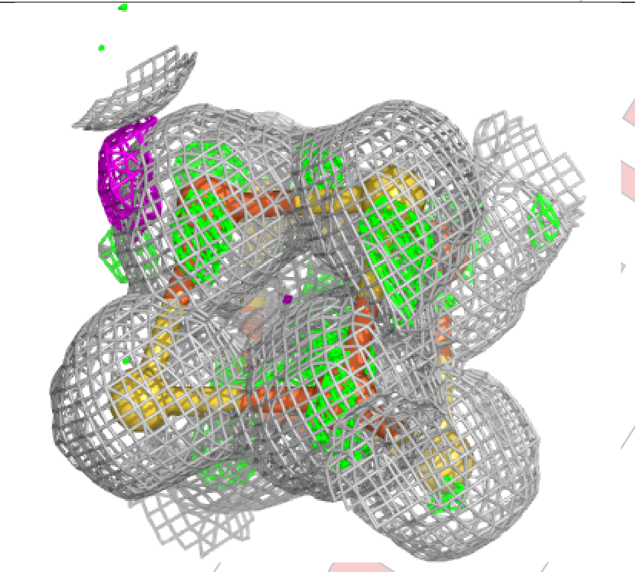

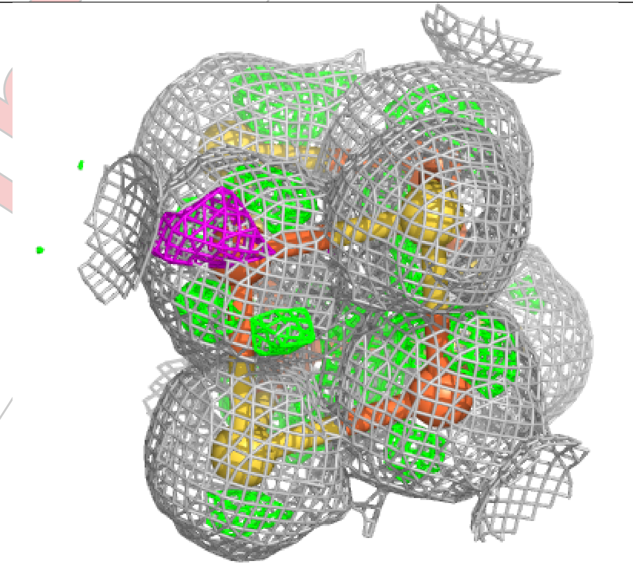

6.5 Other polymers ⓘ

There are no such residues in this entry.

For Manuscript Review

WORLDWIDE PDB PROTEIN DATA BANK

Full wwPDB X-ray Structure Validation Report (i)

Aug 6, 2024 – 03:17 pm BST

PDB ID	:	9GBU
Title	:	Desulfovibrio desulfuricans [FeFe]-hydrogenase variant with both subunits linked by a 13 amino acid linker peptide derived from a group A1 type [FeFe]-hydrogenase of Solobacterium moorei
Deposited on	:	2024-07-31
Resolution	:	1.78 Å(reported)

This wwPDB validation report is for manuscript review

This is a Full wwPDB X-ray Structure Validation Report.

This report is produced by the wwPDB biocuration pipeline after annotation of the structure.

We welcome your comments at *validation@mail.wwpdb.org*
A user guide is available at
https://www.wwpdb.org/validation/2017/XrayValidationReportHelp
with specific help available everywhere you see the (i) symbol.

The types of validation reports are described at
http://www.wwpdb.org/validation/2017/FAQs#types.

For Manuscript Review

The following versions of software and data (see references (i)) were used in the production of this report:

MolProbity	:	4.02b-467
Mogul	:	1.8.4, CSD as541be (2020)
Xtriage (Phenix)	:	1.13
EDS	:	2.37.1
buster-report	:	1.1.7 (2018)
Percentile statistics	:	20191225.v01 (using entries in the PDB archive December 25th 2019)
Refmac	:	5.8.0158
CCP4	:	7.0.044 (Gargrove)

1 Overall quality at a glance (i)

The following experimental techniques were used to determine the structure:
X-RAY DIFFRACTION

The reported resolution of this entry is 1.78 Å.

Percentile scores (ranging between 0-100) for global validation metrics of the entry are shown in the following graphic. The table shows the number of entries on which the scores are based.

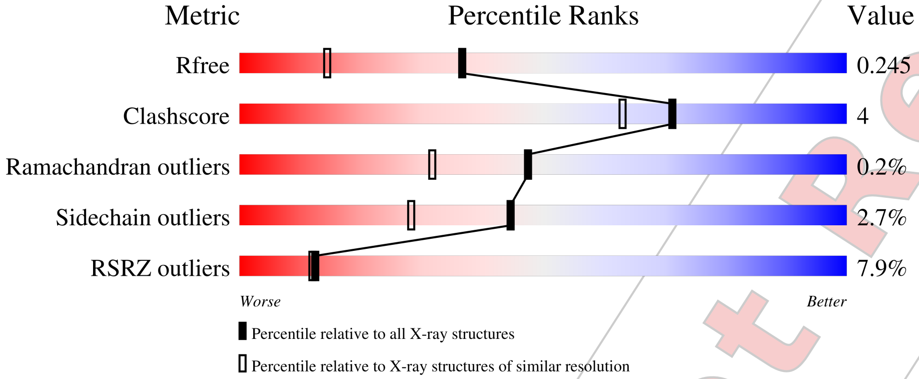

Metric	Whole archive (#Entries)	Similar resolution (#Entries, resolution range(Å))
R_{free}	130704	9185 (1.80-1.76)
Clashscore	141614	10184 (1.80-1.76)
Ramachandran outliers	138981	10051 (1.80-1.76)
Sidechain outliers	138945	10050 (1.80-1.76)
RSRZ outliers	127900	9032 (1.80-1.76)

The table below summarises the geometric issues observed across the polymeric chains and their fit to the electron density. The red, orange, yellow and green segments of the lower bar indicate the fraction of residues that contain outliers for >=3, 2, 1 and 0 types of geometric quality criteria respectively. A grey segment represents the fraction of residues that are not modelled. The numeric value for each fraction is indicated below the corresponding segment, with a dot representing fractions <=5% The upper red bar (where present) indicates the fraction of residues that have poor fit to the electron density. The numeric value is given above the bar.

Mol	Chain	Length	Quality of chain
1	A	490	6% / 89% 8% • •
1	B	490	9% / 87% 9% • • •

The following table lists non-polymeric compounds, carbohydrate monomers and non-standard

Ideal geometry (proteins)	:	Engh & Huber (2001)
Ideal geometry (DNA, RNA)	:	Parkinson et al. (1996)
Validation Pipeline (wwPDB-VP)	:	2.37.1

For Manuscript Review

residues in protein, DNA, RNA chains that are outliers for geometric or electron-density-fit criteria:

Mol	Type	Chain	Res	Chirality	Geometry	Clashes	Electron density
4	SF4	B	503	-	-	X	-

For Manuscript Review

2 Entry composition (i)

There are 5 unique types of molecules in this entry. The entry contains 15309 atoms, of which 7514 are hydrogens and 0 are deuteriums.

In the tables below, the ZeroOcc column contains the number of atoms modelled with zero occupancy, the AltConf column contains the number of residues with at least one atom in alternate conformation and the Trace column contains the number of residues modelled with at most 2 atoms.

- Molecule 1 is a protein called Periplasmic [Fe] hydrogenase large subunit,Periplasmic [Fe] hydrogenase small subunit.

Mol	Chain	Residues	Atoms						ZeroOcc	AltConf	Trace
			Total	C	H	N	O	S			
1	A	478	7402	2355	3695	629	690	33	118	5	0
1	B	480	7458	2370	3721	637	695	35	119	8	0

There are 46 discrepancies between the modelled and reference sequences:

Chain	Residue	Modelled	Actual	Comment	Reference
A	390	ARG	-	linker	UNP P07598
A	391	HIS	-	linker	UNP P07598
A	392	LYS	-	linker	UNP P07598
A	393	LEU	-	linker	UNP P07598
A	394	PRO	-	linker	UNP P07598
A	395	GLN	-	linker	UNP P07598
A	396	VAL	-	linker	UNP P07598
A	397	LYS	-	linker	UNP P07598
A	398	ALA	-	linker	UNP P07598
A	399	ALA	-	linker	UNP P07598
A	400	LYS	-	linker	UNP P07598
A	401	GLU	-	linker	UNP P07598
A	402	SER	-	linker	UNP P07598
A	481	SER	-	expression tag	UNP P07603
A	482	ALA	-	expression tag	UNP P07603
A	483	TRP	-	expression tag	UNP P07603
A	484	SER	-	expression tag	UNP P07603
A	485	HIS	-	expression tag	UNP P07603
A	486	PRO	-	expression tag	UNP P07603
A	487	GLN	-	expression tag	UNP P07603
A	488	PHE	-	expression tag	UNP P07603
A	489	GLU	-	expression tag	UNP P07603
A	490	LYS	-	expression tag	UNP P07603
B	390	ARG	-	linker	UNP P07598

Continued on next page...

For Manuscript Review

Continued from previous page...

Chain	Residue	Modelled	Actual	Comment	Reference
B	391	HIS	-	linker	UNP P07598
B	392	LYS	-	linker	UNP P07598
B	393	LEU	-	linker	UNP P07598
B	394	PRO	-	linker	UNP P07598
B	395	GLN	-	linker	UNP P07598
B	396	VAL	-	linker	UNP P07598
B	397	LYS	-	linker	UNP P07598
B	398	ALA	-	linker	UNP P07598
B	399	ALA	-	linker	UNP P07598
B	400	LYS	-	linker	UNP P07598
B	401	GLU	-	linker	UNP P07598
B	402	SER	-	linker	UNP P07598
B	481	SER	-	expression tag	UNP P07603
B	482	ALA	-	expression tag	UNP P07603
B	483	TRP	-	expression tag	UNP P07603
B	484	SER	-	expression tag	UNP P07603
B	485	HIS	-	expression tag	UNP P07603
B	486	PRO	-	expression tag	UNP P07603
B	487	GLN	-	expression tag	UNP P07603
B	488	PHE	-	expression tag	UNP P07603
B	489	GLU	-	expression tag	UNP P07603
B	490	LYS	-	expression tag	UNP P07603

- Molecule 2 is (4S)-2-METHYL-2,4-PENTANEDIOL (three-letter code: MPD) (formula: $C_6H_{14}O_2$).

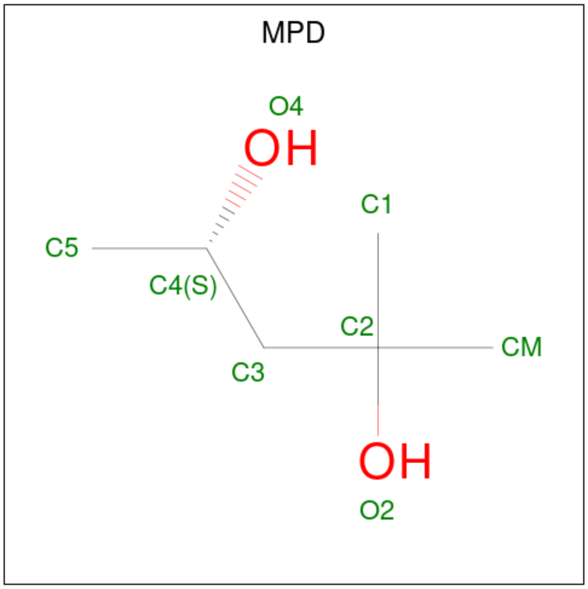

For Manuscript Review

Mol	Chain	Residues	Atoms				ZeroOcc	AltConf
			Total	C	H	O		
2	A	1	22	6	14	2	2	0
2	A	1	22	6	14	2	2	0
2	A	1	22	6	14	2	2	0
2	A	1	22	6	14	2	2	0
2	B	1	22	6	14	2	2	0
2	B	1	22	6	14	2	2	0

- Molecule 3 is dicarbonyl[bis(cyanide-kappaC)]-mu-(iminodimethanethiolatato-1kappaS:2kappaS)-mu-(oxomethylidene)diiron(2+) (three-letter code: 402) (formula: $C_7H_5Fe_2N_3O_3S_2$) (labeled as "Ligand of Interest" by depositor).

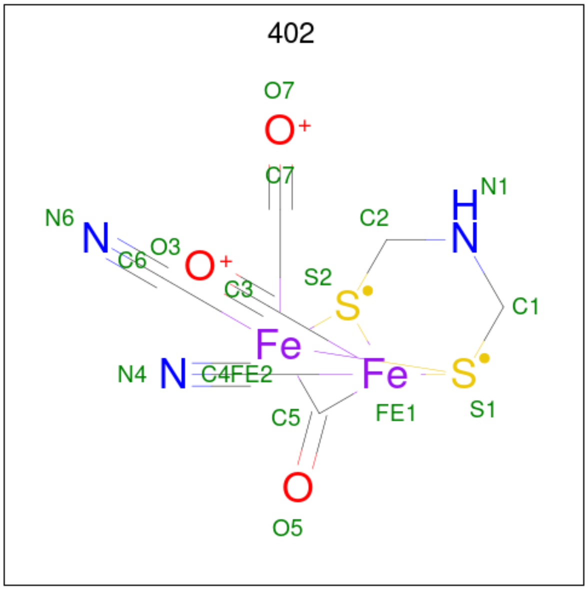

Mol	Chain	Residues	Atoms							ZeroOcc	AltConf
			Total	C	Fe	H	N	O	S		
3	A	1	24	7	2	7	3	3	2	1	0
3	B	1	24	7	2	7	3	3	2	1	0

- Molecule 4 is IRON/SULFUR CLUSTER (three-letter code: SF4) (formula: Fe_4S_4) (labeled as "Ligand of Interest" by depositor).

For Manuscript Review

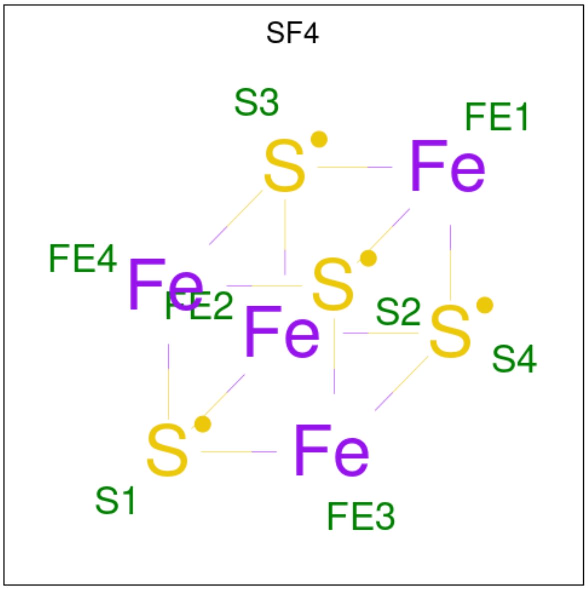

Mol	Chain	Residues	Atoms	ZeroOcc	AltConf
4	A	1	Total Fe S<br>8 4 4	0	0
4	A	1	Total Fe S<br>8 4 4	0	0
4	A	1	Total Fe S<br>8 4 4	0	0
4	B	1	Total Fe S<br>8 4 4	0	0
4	B	1	Total Fe S<br>8 4 4	0	0
4	B	1	Total Fe S<br>8 4 4	0	0

- Molecule 5 is water.

Mol	Chain	Residues	Atoms	ZeroOcc	AltConf
5	A	130	Total O<br>130 130	0	0
5	B	91	Total O<br>91 91	0	0

For Manuscript Review

3 Residue-property plots ⓘ

These plots are drawn for all protein, RNA, DNA and oligosaccharide chains in the entry. The first graphic for a chain summarises the proportions of the various outlier classes displayed in the second graphic. The second graphic shows the sequence view annotated by issues in geometry and electron density. Residues are color-coded according to the number of geometric quality criteria for which they contain at least one outlier: green = 0, yellow = 1, orange = 2 and red = 3 or more. A red dot above a residue indicates a poor fit to the electron density (RSRZ > 2). Stretches of 2 or more consecutive residues without any outlier are shown as a green connector. Residues present in the sample, but not in the model, are shown in grey.

• Molecule 1: Periplasmic [Fe] hydrogenase large subunit,Periplasmic [Fe] hydrogenase small subunit

Chain A:

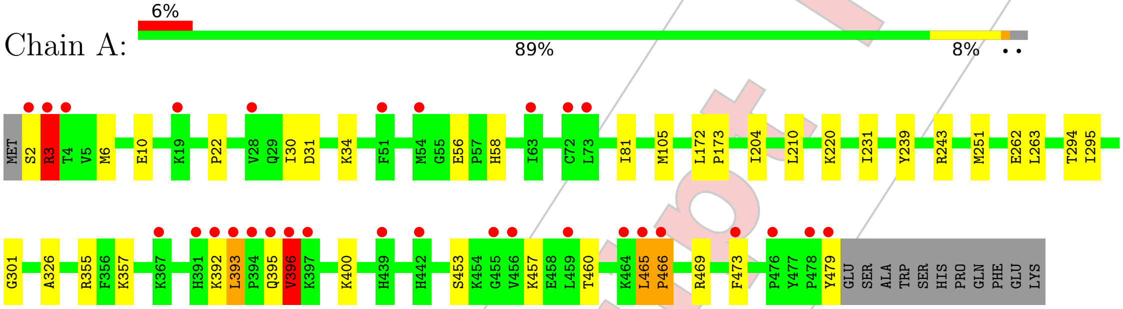

• Molecule 1: Periplasmic [Fe] hydrogenase large subunit,Periplasmic [Fe] hydrogenase small subunit

Chain B:

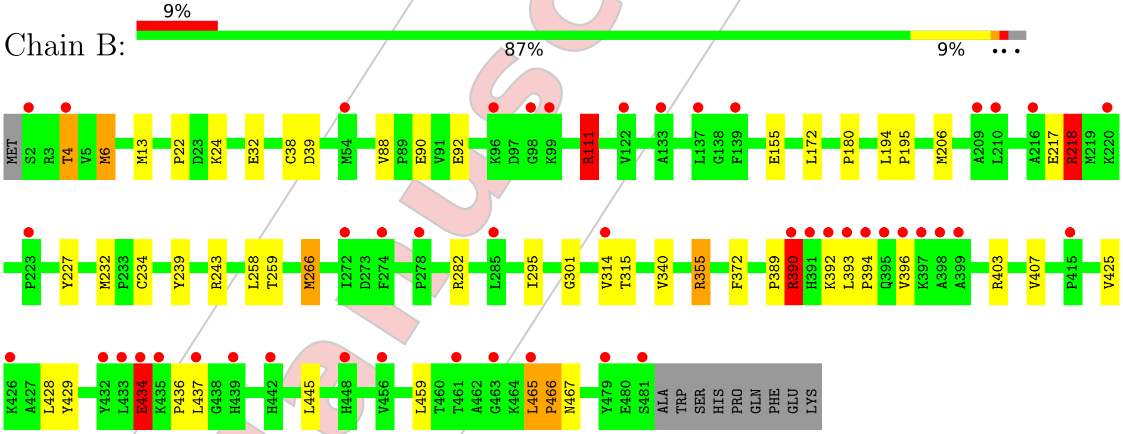

For Manuscript Review

4 Data and refinement statistics (i)

Property	Value	Source
Space group	P 21 21 21	Depositor
Cell constants<br>a, b, c, α, β, γ	93.02Å 98.81Å 112.21Å<br>90.00° 90.00° 90.00°	Depositor
Resolution (Å)	48.84 – 1.78<br>48.79 – 1.78	Depositor<br>EDS
% Data completeness<br>(in resolution range)	99.5 (48.84-1.78)<br>99.5 (48.79-1.78)	Depositor<br>EDS
R_{merge}	0.10	Depositor
R_{sym}	(Not available)	Depositor
$< I/\sigma(I) >$ [1]	1.42 (at 1.78Å)	Xtriage
Refinement program	REFMAC 5.8.0425	Depositor
R, R_{free}	0.201 , 0.242<br>0.209 , 0.245	Depositor<br>DCC
R_{free} test set	4726 reflections (4.81%)	wwPDB-VP
Wilson B-factor (Å^2)	29.6	Xtriage
Anisotropy	0.373	Xtriage
Bulk solvent k_{sol}(e/Å^3), B_{sol}(Å^2)	0.39 , 45.2	EDS
L-test for twinning[2]	$< \|L\| > = 0.47$, $< L^2 > = 0.29$	Xtriage
Estimated twinning fraction	No twinning to report.	Xtriage
F_o,F_c correlation	0.96	EDS
Total number of atoms	15309	wwPDB-VP
Average B, all atoms (Å^2)	42.0	wwPDB-VP

Xtriage's analysis on translational NCS is as follows: *The largest off-origin peak in the Patterson function is 3.85% of the height of the origin peak. No significant pseudotranslation is detected.*

[1]Intensities estimated from amplitudes.

[2]Theoretical values of $< |L| >$, $< L^2 >$ for acentric reflections are 0.5, 0.333 respectively for untwinned datasets, and 0.375, 0.2 for perfectly twinned datasets.

For Manuscript Review

5 Model quality (i)

5.1 Standard geometry (i)

Bond lengths and bond angles in the following residue types are not validated in this section: SF4, MPD, 402

The Z score for a bond length (or angle) is the number of standard deviations the observed value is removed from the expected value. A bond length (or angle) with $|Z| > 5$ is considered an outlier worth inspection. RMSZ is the root-mean-square of all Z scores of the bond lengths (or angles).

Mol	Chain	Bond lengths		Bond angles	
		RMSZ	#|Z| >5	RMSZ	#|Z| >5
1	A	0.46	1/3823 (0.0%)	0.85	6/5166 (0.1%)
1	B	0.43	0/3859	0.91	9/5214 (0.2%)
All	All	0.45	1/7682 (0.0%)	0.88	15/10380 (0.1%)

Chiral center outliers are detected by calculating the chiral volume of a chiral center and verifying if the center is modelled as a planar moiety or with the opposite hand.A planarity outlier is detected by checking planarity of atoms in a peptide group, atoms in a mainchain group or atoms of a sidechain that are expected to be planar.

Mol	Chain	#Chirality outliers	#Planarity outliers
1	A	0	2
1	B	0	10
All	All	0	12

All (1) bond length outliers are listed below:

Mol	Chain	Res	Type	Atoms	Z	Observed(Å)	Ideal(Å)
1	A	466	PRO	N-CD	5.05	1.54	1.47

All (15) bond angle outliers are listed below:

Mol	Chain	Res	Type	Atoms	Z	Observed(^{o})	Ideal(^{o})
1	B	4	THR	OG1-CB-CG2	-15.97	73.28	110.00
1	B	4	THR	CA-CB-OG1	-15.10	77.29	109.00
1	B	434	GLU	CB-CA-C	-11.87	86.66	110.40
1	A	355	ARG	NE-CZ-NH2	-8.36	116.12	120.30
1	A	479	TYR	N-CA-CB	8.28	125.51	110.60
1	B	466	PRO	N-CA-C	-7.67	92.16	112.10
1	B	111	ARG	CB-CA-C	-7.13	96.15	110.40
1	A	3	ARG	CB-CA-C	-6.69	97.02	110.40

Continued on next page...

For Manuscript Review

Continued from previous page...

Mol	Chain	Res	Type	Atoms	Z	Observed(°)	Ideal(°)
1	B	6	MET	CG-SD-CE	6.67	110.88	100.20
1	A	3	ARG	N-CA-C	6.64	128.93	111.00
1	B	4	THR	CA-CB-CG2	6.51	121.52	112.40
1	B	266	MET	CG-SD-CE	6.44	110.51	100.20
1	A	355	ARG	NE-CZ-NH1	5.25	122.92	120.30
1	A	466	PRO	N-CA-C	-5.09	98.87	112.10
1	B	466	PRO	CB-CA-C	5.01	124.54	112.00

There are no chirality outliers.

All (12) planarity outliers are listed below:

Mol	Chain	Res	Type	Group
1	A	243	ARG	Sidechain
1	A	3	ARG	Sidechain
1	B	111	ARG	Sidechain
1	B	172	LEU	Peptide,Mainchain
1	B	218[A]	ARG	Sidechain
1	B	243	ARG	Sidechain
1	B	355	ARG	Sidechain
1	B	389	PRO	Mainchain
1	B	390	ARG	Sidechain
1	B	434	GLU	Peptide,Mainchain

5.2 Too-close contacts (i)

In the following table, the Non-H and H(model) columns list the number of non-hydrogen atoms and hydrogen atoms in the chain respectively. The H(added) column lists the number of hydrogen atoms added and optimized by MolProbity. The Clashes column lists the number of clashes within the asymmetric unit, whereas Symm-Clashes lists symmetry-related clashes.

Mol	Chain	Non-H	H(model)	H(added)	Clashes	Symm-Clashes
1	A	3707	3695	3658	17	1
1	B	3737	3721	3668	34	1
2	A	32	56	56	3	0
2	B	16	28	28	0	0
3	A	17	7	5	0	0
3	B	17	7	5	0	0
4	A	24	0	0	0	0
4	B	24	0	0	2	0
5	A	130	0	0	1	0
5	B	91	0	0	1	0

Continued on next page...

For Manuscript Review

Continued from previous page...

Mol	Chain	Non-H	H(model)	H(added)	Clashes	Symm-Clashes
All	All	7795	7514	7420	53	1

The all-atom clashscore is defined as the number of clashes found per 1000 atoms (including hydrogen atoms). The all-atom clashscore for this structure is 4.

All (53) close contacts within the same asymmetric unit are listed below, sorted by their clash magnitude.

Atom-1	Atom-2	Interatomic distance (Å)	Clash overlap (Å)
1:B:434:GLU:O	1:B:434:GLU:HG2	1.72	0.86
1:B:434:GLU:O	1:B:434:GLU:CG	2.25	0.82
1:B:465:LEU:O	1:B:466:PRO:C	2.23	0.76
2:A:502:MPD:O2	5:A:601:HOH:O	2.04	0.75
1:A:465:LEU:HD23	1:A:465:LEU:O	2.00	0.61
1:B:217:GLU:OE2	1:B:218[B]:ARG:NH1	2.39	0.55
1:B:459:LEU:HB3	1:B:465:LEU:HB2	1.88	0.55
1:B:6:MET:HE1	1:B:88:VAL:HG11	1.89	0.54
1:B:390:ARG:N	1:B:390:ARG:CD	2.72	0.53
1:B:436:PRO:O	1:B:437:LEU:HB2	2.09	0.52
1:B:38:CYS:O	1:B:39:ASP:HB2	2.10	0.52
1:B:88:VAL:O	1:B:92:GLU:HG3	2.09	0.52
1:B:465:LEU:O	1:B:467:ASN:N	2.43	0.52
1:A:460:THR:HA	1:A:466:PRO:HD3	1.92	0.52
1:B:155:GLU:OE2	1:B:429:TYR:OH	2.19	0.50
1:A:469:ARG:O	1:A:473:PHE:CE2	2.64	0.50
1:B:295:ILE:O	1:B:301:GLY:HA3	2.11	0.50
1:B:4:THR:HB	1:B:13:MET:HE3	1.93	0.50
1:A:295:ILE:O	1:A:301:GLY:HA3	2.11	0.49
1:A:393:LEU:HD22	1:A:396:VAL:HG13	1.94	0.48
1:B:90:GLU:OE2	1:B:227:TYR:OH	2.18	0.47
1:B:194:LEU:N	1:B:195:PRO:CD	2.77	0.47
1:B:390:ARG:H	1:B:390:ARG:HD2	1.80	0.46
1:B:314:VAL:HG23	1:B:315:THR:HG23	1.97	0.46
1:B:234:CYS:HB2	4:B:503:SF4:S3	2.56	0.45
1:A:30:ILE:HD12	1:A:81:ILE:HG12	1.97	0.45
1:B:22:PRO:HB2	1:B:239:TYR:CD2	2.52	0.45
1:A:465:LEU:O	1:A:465:LEU:CD2	2.64	0.45
1:A:453:SER:O	1:A:457:LYS:HG3	2.17	0.45
1:B:390:ARG:CD	1:B:390:ARG:H	2.29	0.44
1:A:2:SER:HA	1:A:10:GLU:HB3	1.98	0.44
1:B:459:LEU:CB	1:B:465:LEU:HB2	2.47	0.44
1:B:314:VAL:HG21	1:B:340:VAL:HG11	2.00	0.43

Continued on next page...

For Manuscript Review

Continued from previous page...

Atom-1	Atom-2	Interatomic distance (Å)	Clash overlap (Å)
1:A:31:ASP:OD2	1:A:34:LYS:HE2	2.18	0.43
1:A:105:MET:HE3	1:A:204:ILE:HD11	2.01	0.43
1:B:88:VAL:HG13	1:B:266:MET:HE3	2.00	0.43
1:A:172:LEU:HA	1:A:173:PRO:C	2.39	0.43
1:A:396:VAL:O	1:A:400:LYS:HG3	2.19	0.43
1:B:88:VAL:HG22	1:B:266:MET:HE3	2.00	0.43
1:B:180:PRO:HD2	4:B:503:SF4:S2	2.59	0.42
1:A:326:ALA:HB2	2:A:504:MPD:H53	2.01	0.42
1:B:206[A]:MET:HB3	1:B:445:LEU:HD22	2.02	0.42
1:B:425:VAL:O	1:B:428:LEU:HB3	2.20	0.42
1:A:22:PRO:HB2	1:A:239:TYR:CD2	2.55	0.42
2:A:502:MPD:O4	2:A:502:MPD:HM1	2.20	0.42
1:B:393:LEU:N	1:B:394:PRO:CD	2.83	0.41
1:B:390:ARG:N	1:B:390:ARG:HD2	2.35	0.41
1:B:465:LEU:C	1:B:466:PRO:O	2.55	0.41
1:A:231:ILE:HG21	1:A:263:LEU:HD22	2.03	0.41
1:B:403:ARG:O	1:B:407:VAL:HG23	2.20	0.41
1:B:282[A]:ARG:NH1	5:B:609:HOH:O	2.54	0.41
1:B:232:MET:O	1:B:259:THR:HA	2.21	0.40
1:A:6:MET:HE3	1:A:262:GLU:HG2	2.03	0.40

All (1) symmetry-related close contacts are listed below. The label for Atom-2 includes the symmetry operator and encoded unit-cell translations to be applied.

Atom-1	Atom-2	Interatomic distance (Å)	Clash overlap (Å)
1:A:220[B]:LYS:HZ1	1:B:24:LYS:O[3_544]	1.58	0.02

5.3 Torsion angles (i)

5.3.1 Protein backbone (i)

In the following table, the Percentiles column shows the percent Ramachandran outliers of the chain as a percentile score with respect to all X-ray entries followed by that with respect to entries of similar resolution.

The Analysed column shows the number of residues for which the backbone conformation was analysed, and the total number of residues.

Mol	Chain	Analysed	Favoured	Allowed	Outliers	Percentiles	
1	A	481/490 (98%)	462 (96%)	17 (4%)	2 (0%)	34	19

Continued on next page...

For Manuscript Review

Continued from previous page...

Mol	Chain	Analysed	Favoured	Allowed	Outliers	Percentiles	
1	B	486/490 (99%)	472 (97%)	14 (3%)	0	100	100
All	All	967/980 (99%)	934 (97%)	31 (3%)	2 (0%)	47	32

All (2) Ramachandran outliers are listed below:

Mol	Chain	Res	Type
1	A	396	VAL
1	A	393	LEU

5.3.2 Protein sidechains (i)

In the following table, the Percentiles column shows the percent sidechain outliers of the chain as a percentile score with respect to all X-ray entries followed by that with respect to entries of similar resolution.

The Analysed column shows the number of residues for which the sidechain conformation was analysed, and the total number of residues.

Mol	Chain	Analysed	Rotameric	Outliers	Percentiles	
1	A	396/402 (98%)	385 (97%)	11 (3%)	43	27
1	B	401/402 (100%)	390 (97%)	11 (3%)	44	28
All	All	797/804 (99%)	775 (97%)	22 (3%)	44	27

All (22) residues with a non-rotameric sidechain are listed below:

Mol	Chain	Res	Type
1	A	3	ARG
1	A	56	GLU
1	A	58	HIS
1	A	210	LEU
1	A	251	MET
1	A	294	THR
1	A	357	LYS
1	A	392	LYS
1	A	395	GLN
1	A	396	VAL
1	A	465	LEU
1	B	32	GLU
1	B	111	ARG
1	B	218[A]	ARG

Continued on next page...

For Manuscript Review

Continued from previous page...

Mol	Chain	Res	Type
1	B	218[B]	ARG
1	B	258	LEU
1	B	355	ARG
1	B	372	PHE
1	B	390	ARG
1	B	392	LYS
1	B	396	VAL
1	B	465	LEU

Sometimes sidechains can be flipped to improve hydrogen bonding and reduce clashes. All (1) such sidechains are listed below:

Mol	Chain	Res	Type
1	B	391	HIS

5.3.3 RNA (i)

There are no RNA molecules in this entry.

5.4 Non-standard residues in protein, DNA, RNA chains (i)

There are no non-standard protein/DNA/RNA residues in this entry.

5.5 Carbohydrates (i)

There are no monosaccharides in this entry.

5.6 Ligand geometry (i)

14 ligands are modelled in this entry.

In the following table, the Counts columns list the number of bonds (or angles) for which Mogul statistics could be retrieved, the number of bonds (or angles) that are observed in the model and the number of bonds (or angles) that are defined in the Chemical Component Dictionary. The Link column lists molecule types, if any, to which the group is linked. The Z score for a bond length (or angle) is the number of standard deviations the observed value is removed from the expected value. A bond length (or angle) with $|Z| > 2$ is considered an outlier worth inspection. RMSZ is the root-mean-square of all Z scores of the bond lengths (or angles).

For Manuscript Review

Mol	Type	Chain	Res	Link	Bond lengths			Bond angles		
					Counts	RMSZ	#|Z| > 2	Counts	RMSZ	#|Z| > 2
3	402	B	504	1	13,19,19	5.41	8 (61%)	2,36,36	1.44	0
4	SF4	A	507	1	0,12,12	-	-	-		
4	SF4	A	506	1	0,12,12	-	-	-		
4	SF4	B	503	1	0,12,12	-	-	-		
4	SF4	B	505	1	0,12,12	-	-	-		
2	MPD	B	501	-	7,7,7	0.14	0	9,10,10	0.24	0
2	MPD	A	503	-	7,7,7	0.22	0	9,10,10	0.59	0
4	SF4	B	506	1	0,12,12	-	-	-		
2	MPD	A	504	-	7,7,7	0.12	0	9,10,10	0.42	0
2	MPD	A	501	-	7,7,7	0.17	0	9,10,10	0.55	0
3	402	A	505	1	13,19,19	5.29	7 (53%)	2,36,36	1.53	0
4	SF4	A	508	1	0,12,12	-	-	-		
2	MPD	A	502	-	7,7,7	0.21	0	9,10,10	0.36	0
2	MPD	B	502	-	7,7,7	0.08	0	9,10,10	0.31	0

In the following table, the Chirals column lists the number of chiral outliers, the number of chiral centers analysed, the number of these observed in the model and the number defined in the Chemical Component Dictionary. Similar counts are reported in the Torsion and Rings columns. '-' means no outliers of that kind were identified.

Mol	Type	Chain	Res	Link	Chirals	Torsions	Rings
3	402	B	504	1	-	-	0/5/3/3
4	SF4	A	507	1	-	-	0/6/5/5
4	SF4	A	506	1	-	-	0/6/5/5
4	SF4	B	503	1	-	-	0/6/5/5
4	SF4	B	505	1	-	-	0/6/5/5
2	MPD	B	501	-	-	0/5/5/5	-
2	MPD	A	503	-	-	2/5/5/5	-
4	SF4	B	506	1	-	-	0/6/5/5
2	MPD	A	504	-	-	0/5/5/5	-
2	MPD	A	501	-	-	1/5/5/5	-
3	402	A	505	1	-	-	0/5/3/3
4	SF4	A	508	1	-	-	0/6/5/5
2	MPD	A	502	-	-	1/5/5/5	-
2	MPD	B	502	-	-	1/5/5/5	-

All (15) bond length outliers are listed below:

Mol	Chain	Res	Type	Atoms	Z	Observed(Å)	Ideal(Å)
3	A	505	402	C2-S2	-10.41	1.66	1.85
3	B	504	402	C1-S1	-10.08	1.67	1.85
3	B	504	402	C2-S2	-9.58	1.68	1.85

Continued on next page...

For Manuscript Review

Continued from previous page...

Mol	Chain	Res	Type	Atoms	Z	Observed(Å)	Ideal(Å)
3	A	505	402	S1-FE2	-8.62	2.14	2.26
3	A	505	402	C1-S1	-8.58	1.69	1.85
3	B	504	402	S2-FE1	-7.21	2.16	2.26
3	A	505	402	O5-C5	7.20	1.29	1.17
3	B	504	402	S1-FE2	-6.71	2.16	2.26
3	B	504	402	C4-N4	6.17	1.26	1.15
3	B	504	402	O5-C5	6.05	1.28	1.17
3	A	505	402	S1-FE1	-5.57	2.18	2.26
3	A	505	402	S2-FE2	-3.98	2.20	2.26
3	B	504	402	S2-FE2	-2.86	2.22	2.26
3	B	504	402	C6-N6	2.12	1.18	1.15
3	A	505	402	O3-C3	2.01	1.20	1.15

There are no bond angle outliers.

There are no chirality outliers.

All (5) torsion outliers are listed below:

Mol	Chain	Res	Type	Atoms
2	A	501	MPD	C2-C3-C4-O4
2	A	502	MPD	C2-C3-C4-C5
2	A	503	MPD	C2-C3-C4-O4
2	B	502	MPD	O2-C2-C3-C4
2	A	503	MPD	C2-C3-C4-C5

There are no ring outliers.

3 monomers are involved in 5 short contacts:

Mol	Chain	Res	Type	Clashes	Symm-Clashes
4	B	503	SF4	2	0
2	A	504	MPD	1	0
2	A	502	MPD	2	0

The following is a two-dimensional graphical depiction of Mogul quality analysis of bond lengths, bond angles, torsion angles, and ring geometry for all instances of the Ligand of Interest. In addition, ligands with molecular weight > 250 and outliers as shown on the validation Tables will also be included. For torsion angles, if less then 5% of the Mogul distribution of torsion angles is within 10 degrees of the torsion angle in question, then that torsion angle is considered an outlier. Any bond that is central to one or more torsion angles identified as an outlier by Mogul will be highlighted in the graph. For rings, the root-mean-square deviation (RMSD) between the ring in question and similar rings identified by Mogul is calculated over all ring torsion angles. If the average RMSD is greater than 60 degrees and the minimal RMSD between the ring in question and

For Manuscript Review

any Mogul-identified rings is also greater than 60 degrees, then that ring is considered an outlier. The outliers are highlighted in purple. The color gray indicates Mogul did not find sufficient equivalents in the CSD to analyse the geometry.

Ligand 402 B 504

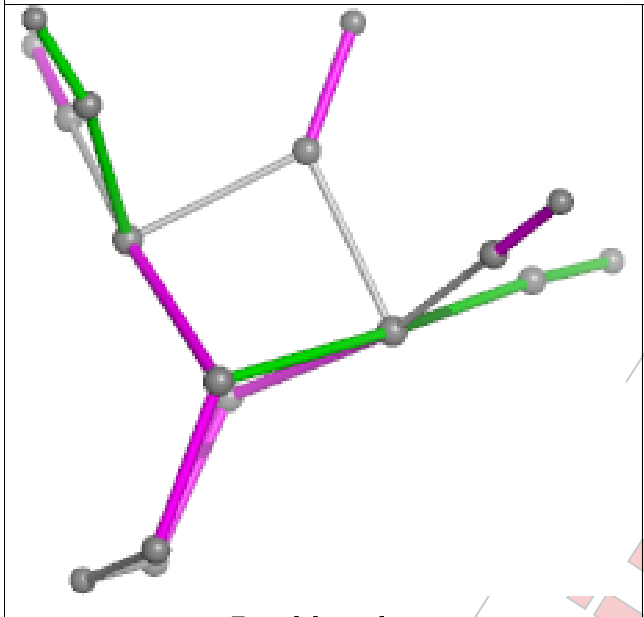

Bond lengths

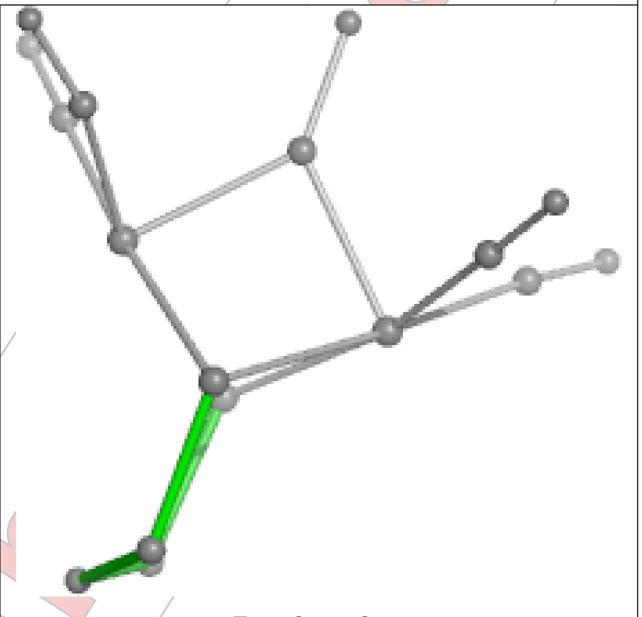

Bond angles

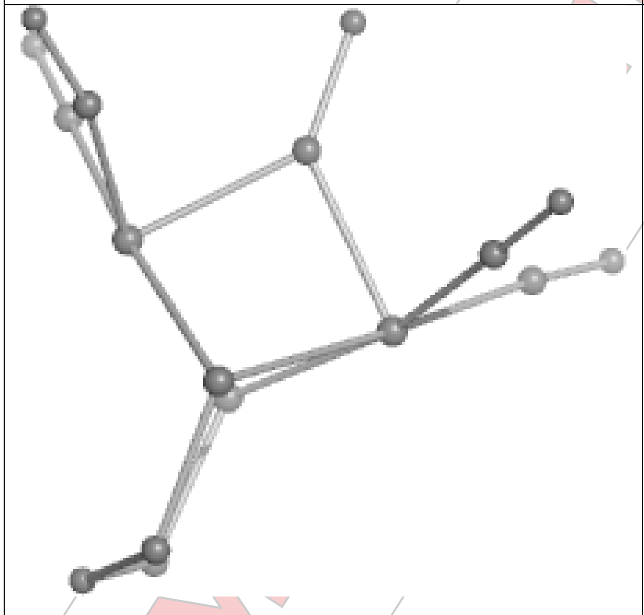

Torsions

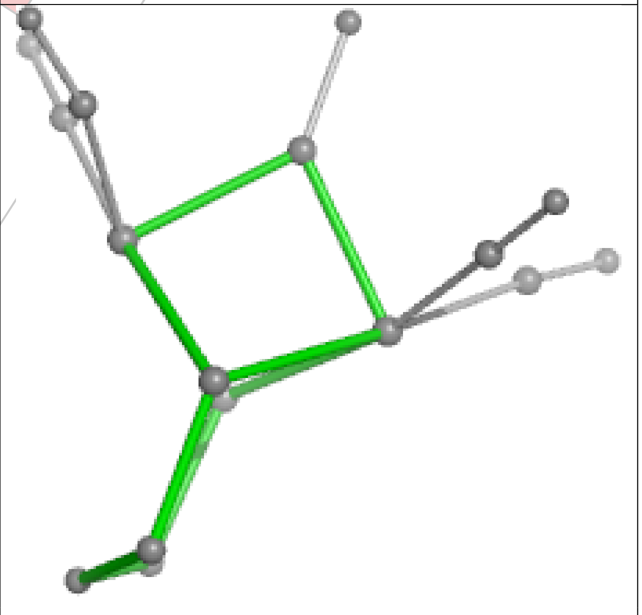

Rings

For Manuscript Review

Ligand SF4 A 507

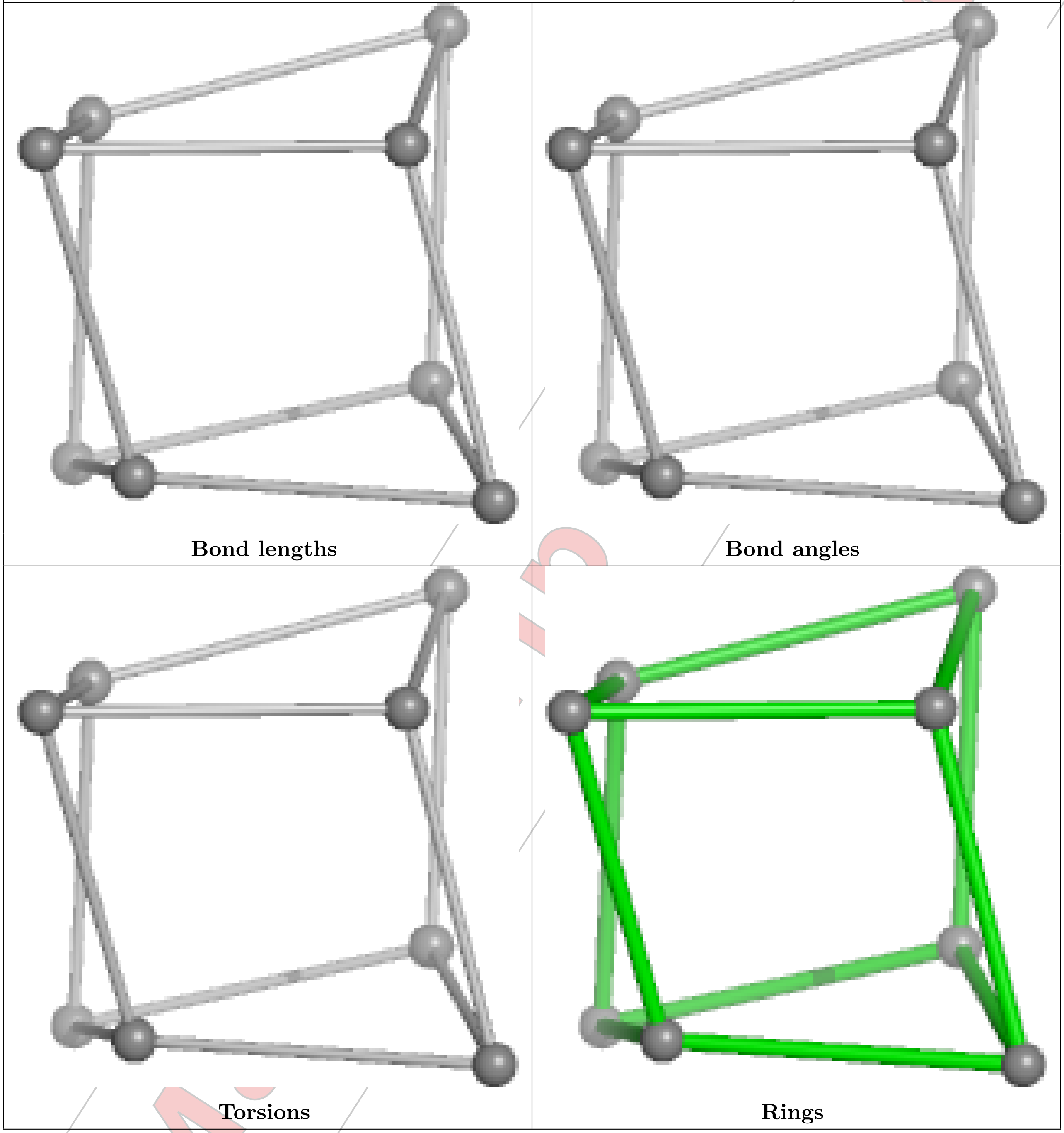

Ligand SF4 A 506

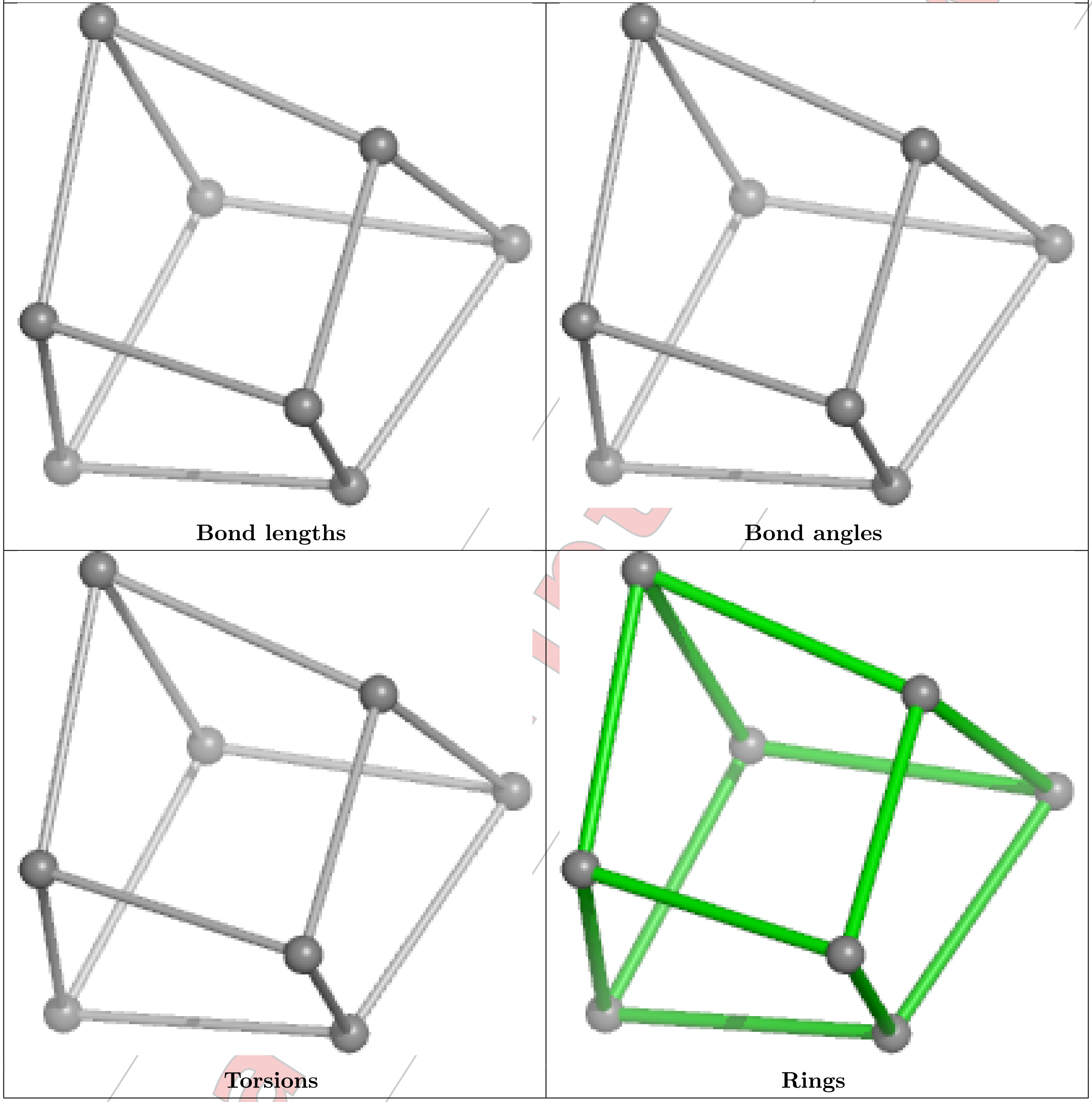

For Manuscript Review

Ligand SF4 B 503

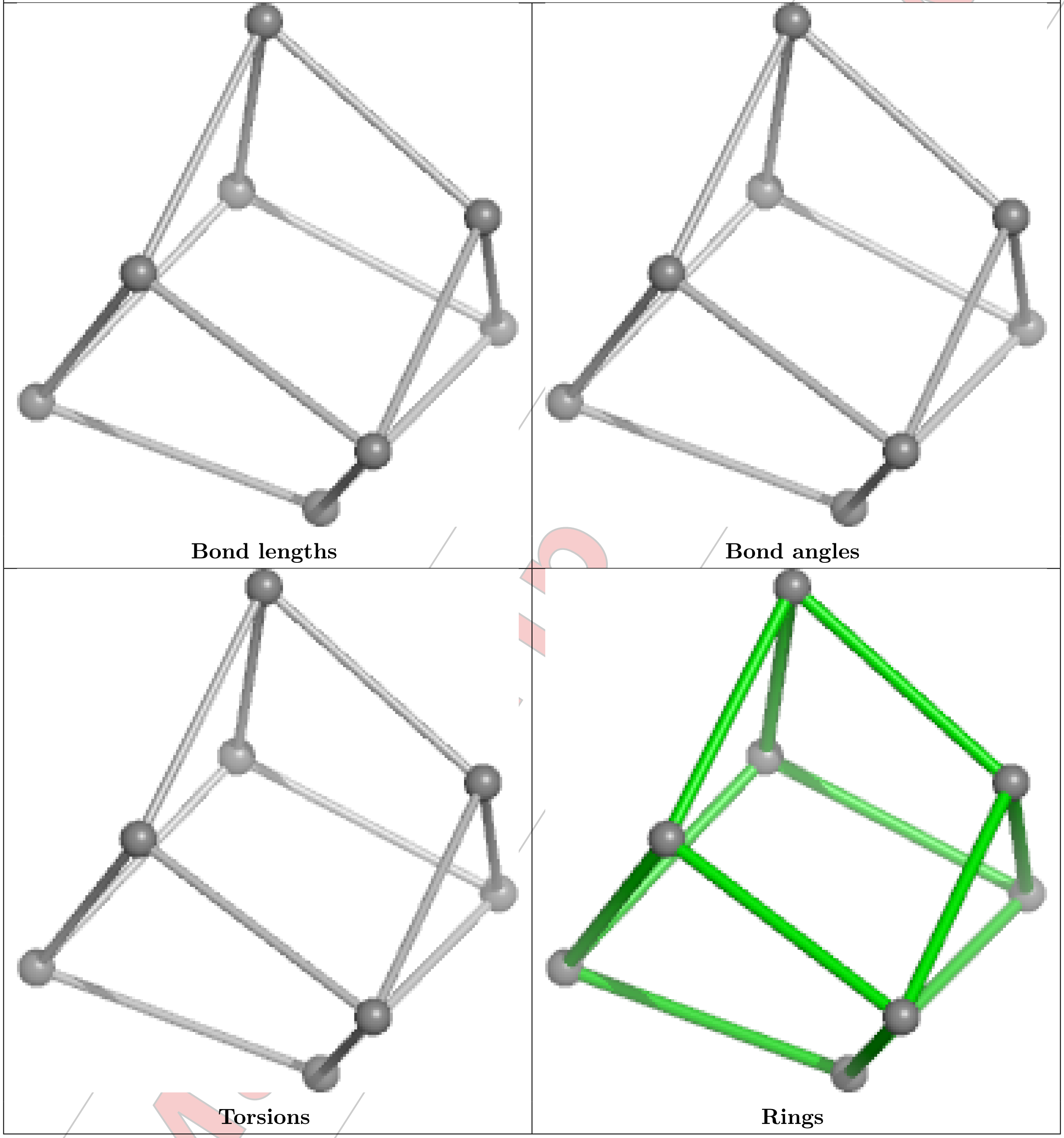

For Manuscript Review

Ligand SF4 B 505

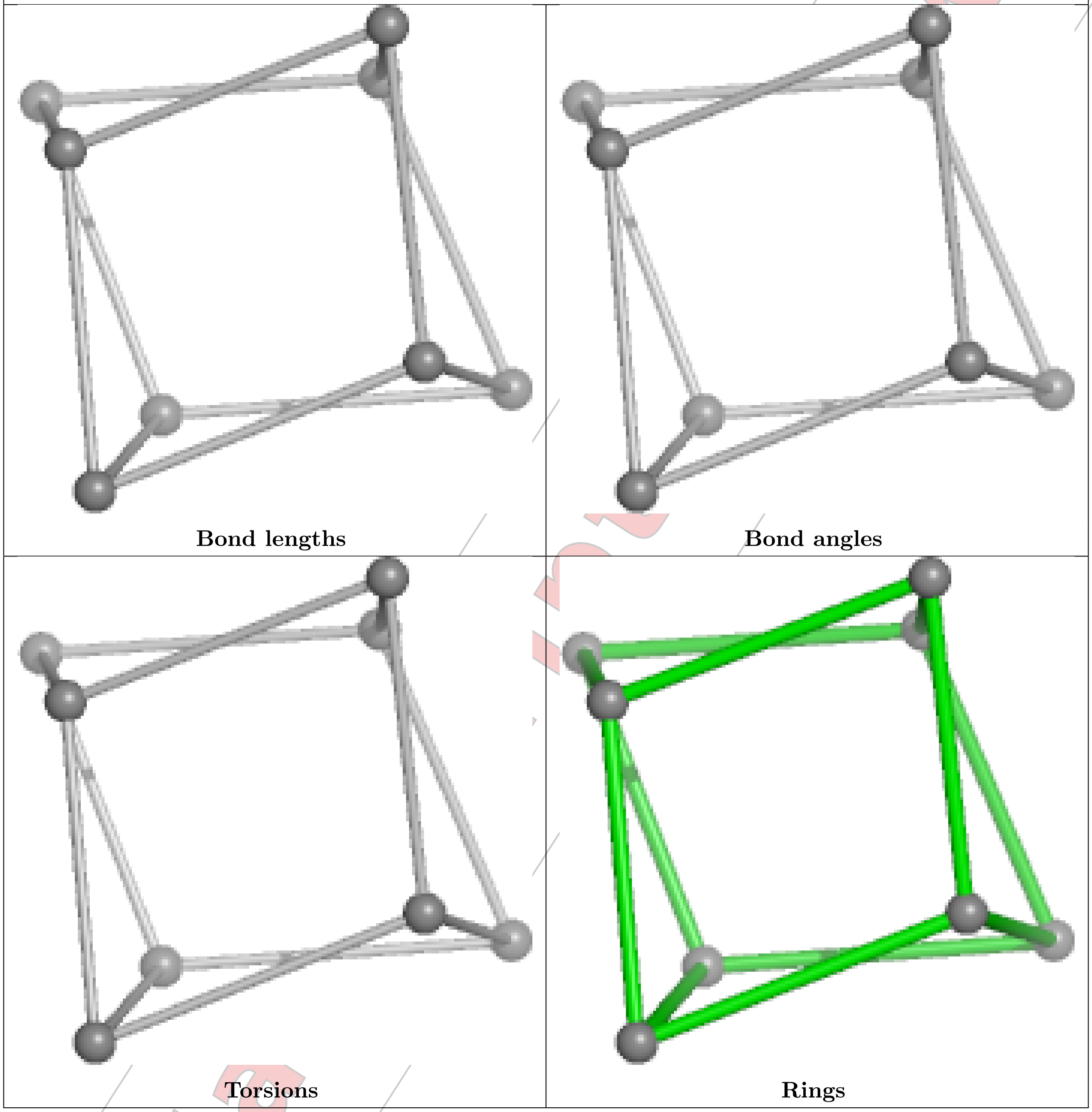

For Manuscript Review

Ligand SF4 B 506

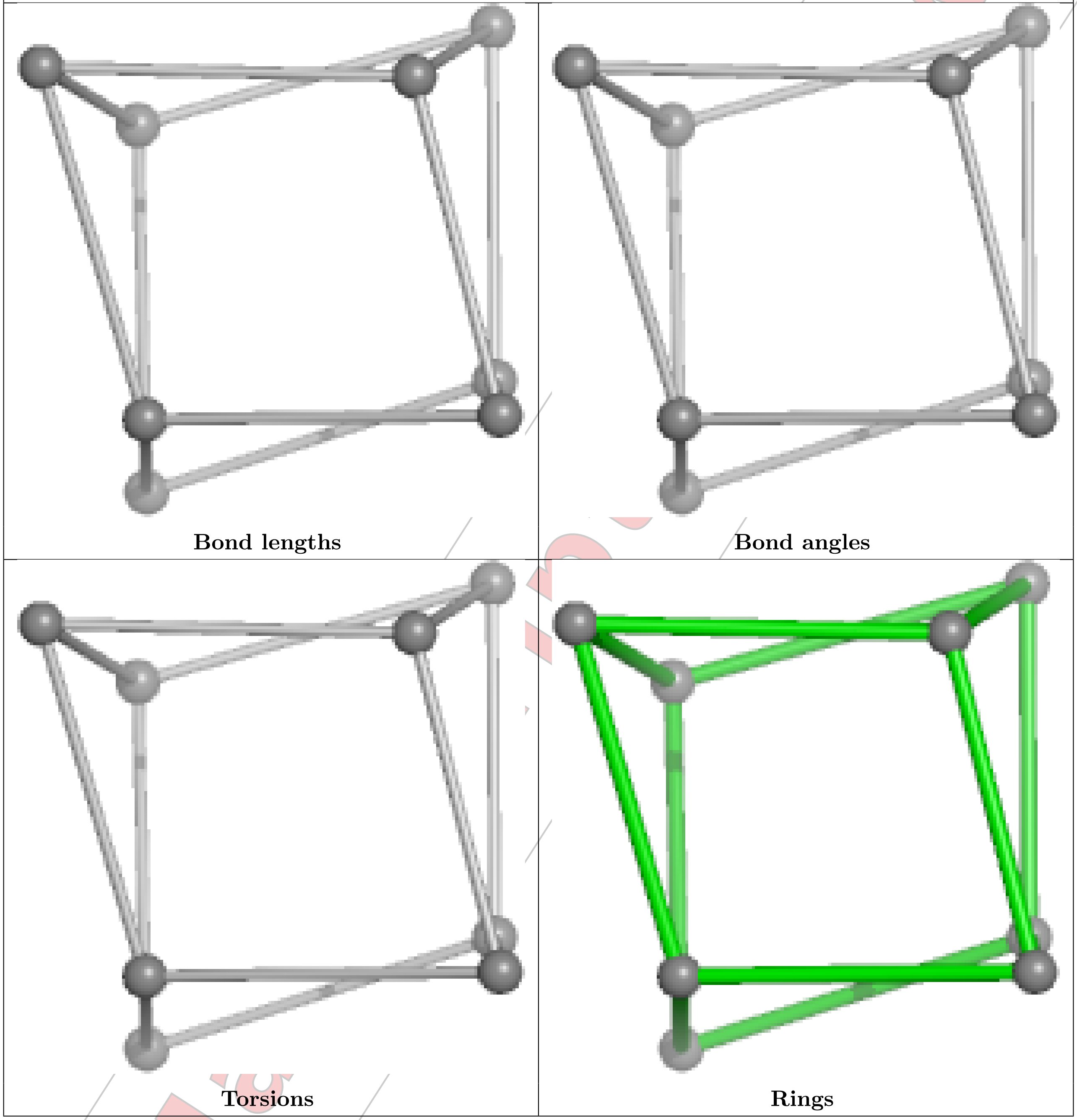

For Manuscript Review

Ligand 402 A 505

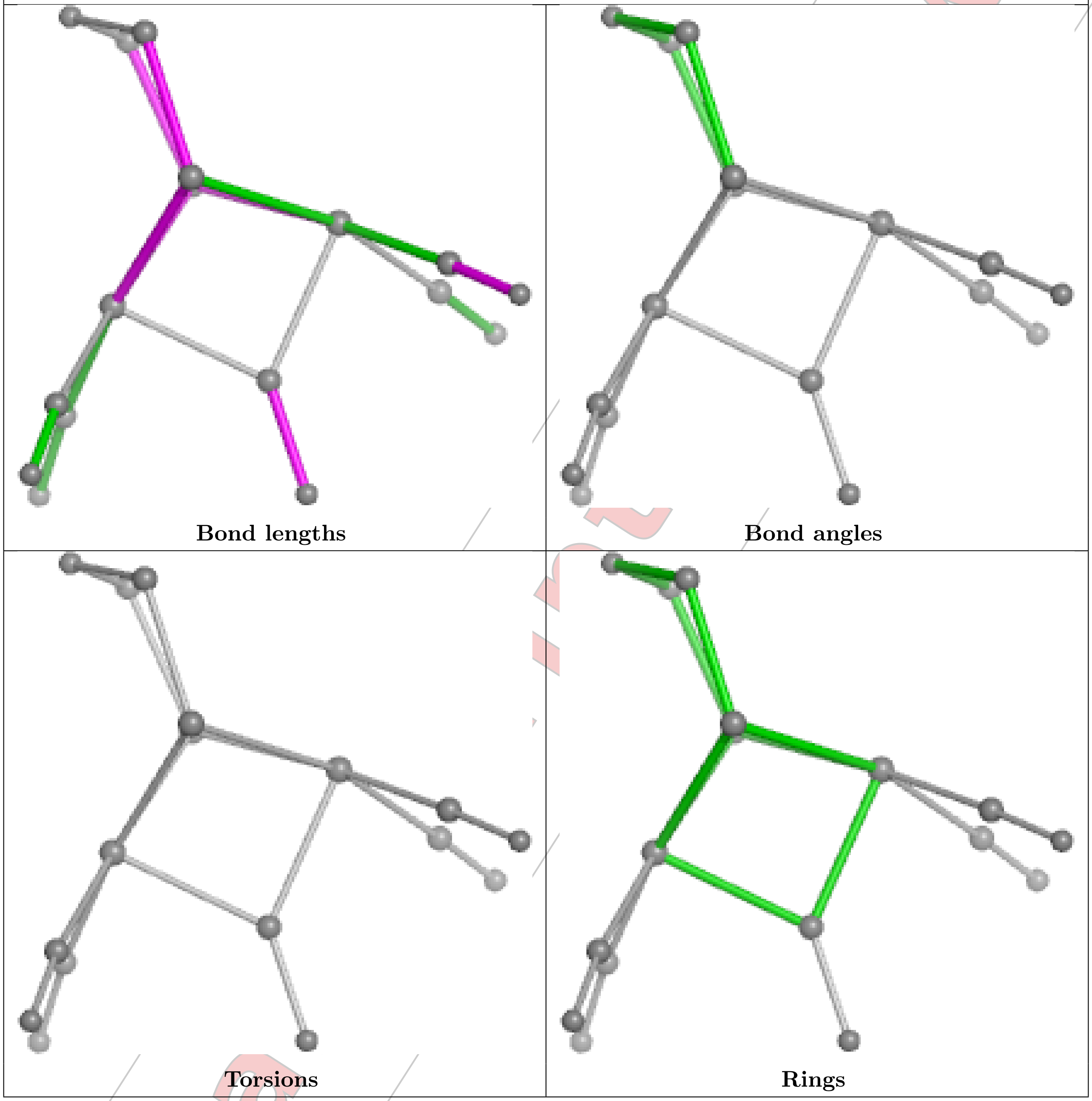

For Manuscript Review

Ligand SF4 A 508

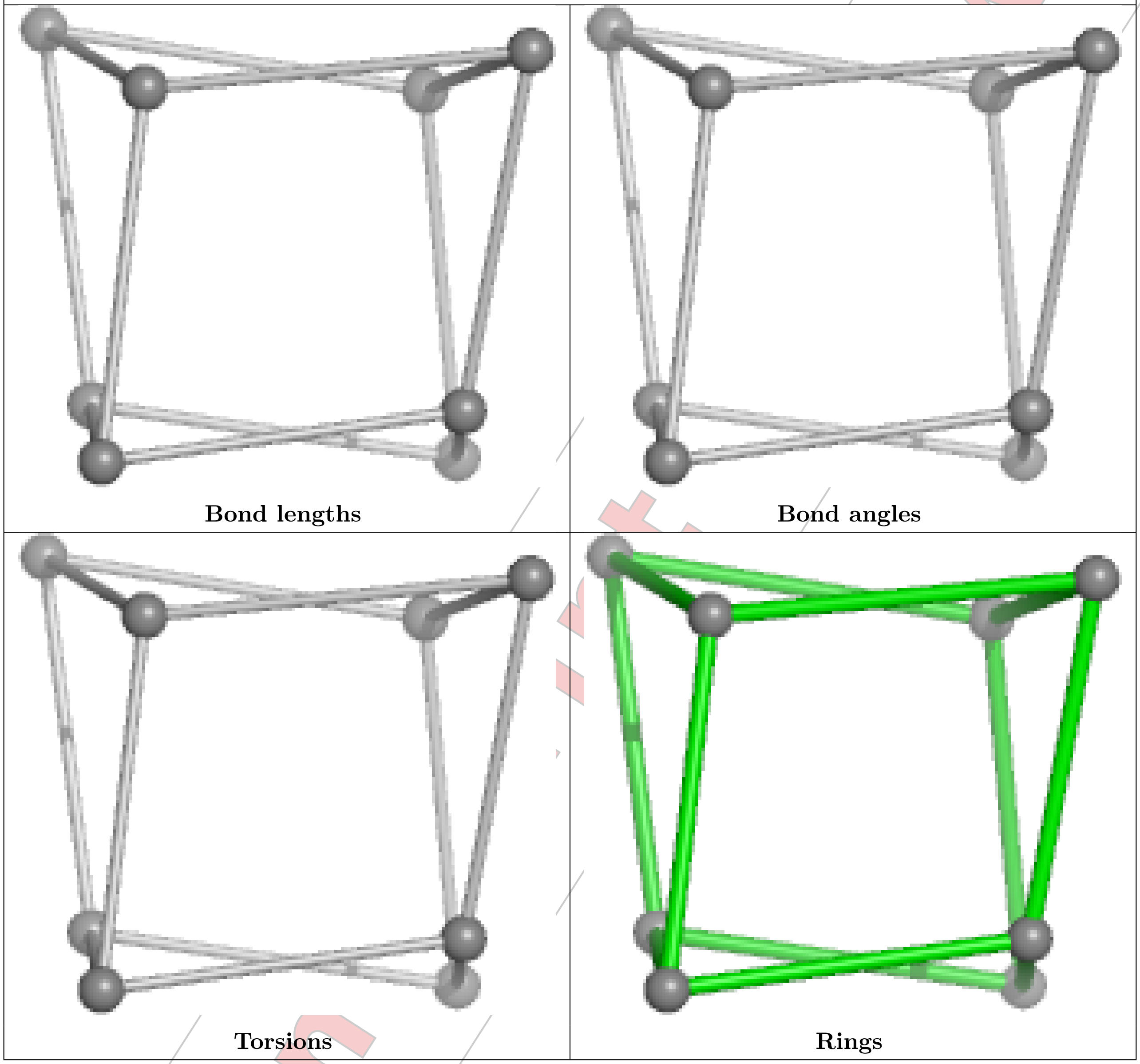

5.7 Other polymers (i)

There are no such residues in this entry.

5.8 Polymer linkage issues (i)

There are no chain breaks in this entry.

For Manuscript Review

6 Fit of model and data (i)

6.1 Protein, DNA and RNA chains (i)

In the following table, the column labelled '#RSRZ> 2' contains the number (and percentage) of RSRZ outliers, followed by percent RSRZ outliers for the chain as percentile scores relative to all X-ray entries and entries of similar resolution. The OWAB column contains the minimum, median, 95th percentile and maximum values of the occupancy-weighted average B-factor per residue. The column labelled 'Q< 0.9' lists the number of (and percentage) of residues with an average occupancy less than 0.9.

Mol	Chain	Analysed	<RSRZ>	#RSRZ>2			OWAB(Å^2)	Q<0.9
1	A	478/490 (97%)	0.54	30 (6%)	20	19	24, 35, 69, 133	0
1	B	480/490 (97%)	0.62	46 (9%)	8	7	26, 40, 67, 134	0
All	All	958/980 (97%)	0.58	76 (7%)	12	12	24, 38, 67, 134	0

All (76) RSRZ outliers are listed below:

Mol	Chain	Res	Type	RSRZ
1	A	393	LEU	9.1
1	A	395	GLN	8.1
1	B	390	ARG	7.4
1	B	393	LEU	7.0
1	A	465	LEU	6.4
1	B	394	PRO	6.1
1	A	392	LYS	6.1
1	B	465	LEU	5.7
1	A	396	VAL	5.7
1	A	391	HIS	5.6
1	A	394	PRO	5.6
1	B	392	LYS	5.6
1	B	396	VAL	5.1
1	B	395	GLN	4.8
1	A	2	SER	4.6
1	A	473	PHE	4.4
1	B	391	HIS	4.4
1	B	398	ALA	4.2
1	A	456	VAL	4.0
1	A	459	LEU	3.8
1	A	3	ARG	3.7
1	B	481	SER	3.7
1	B	137	LEU	3.6
1	B	434	GLU	3.5

Continued on next page...

For Manuscript Review

Continued from previous page...

Mol	Chain	Res	Type	RSRZ
1	B	122	VAL	3.5
1	B	439	HIS	3.5
1	B	397	LYS	3.4
1	B	2	SER	3.3
1	A	476	PRO	3.3
1	B	96	LYS	3.2
1	B	139	PHE	3.2
1	B	216	ALA	3.0
1	B	461	THR	3.0
1	A	367	LYS	2.9
1	B	285	LEU	2.9
1	A	442	HIS	2.9
1	B	278	PRO	2.9
1	B	456	VAL	2.6
1	B	433	LEU	2.6
1	B	479	TYR	2.6
1	B	432	TYR	2.6
1	B	4	THR	2.5
1	A	439	HIS	2.5
1	B	272	ILE	2.5
1	B	426	LYS	2.5
1	A	4	THR	2.5
1	A	397	LYS	2.5
1	B	314	VAL	2.5
1	B	448	HIS	2.5
1	B	223	PRO	2.4
1	B	133	ALA	2.4
1	B	442	HIS	2.4
1	B	98	GLY	2.3
1	A	466	PRO	2.3
1	B	399	ALA	2.3
1	A	479	TYR	2.3
1	A	73	LEU	2.2
1	A	464	LYS	2.2
1	B	220	LYS	2.2
1	A	19	LYS	2.2
1	A	63	ILE	2.2
1	A	54	MET	2.2
1	B	54	MET	2.2
1	B	209	ALA	2.2
1	A	51	PHE	2.1
1	B	99	LYS	2.1

Continued on next page...

For Manuscript Review

Continued from previous page...

Mol	Chain	Res	Type	RSRZ
1	A	455	GLY	2.1
1	B	274	PHE	2.1
1	B	463	GLY	2.1
1	A	28	VAL	2.1
1	B	210	LEU	2.1
1	A	72	CYS	2.1
1	B	415	PRO	2.0
1	B	435	LYS	2.0
1	B	437	LEU	2.0
1	A	478	PRO	2.0

6.2 Non-standard residues in protein, DNA, RNA chains (i)

There are no non-standard protein/DNA/RNA residues in this entry.

6.3 Carbohydrates (i)

There are no monosaccharides in this entry.

6.4 Ligands (i)

In the following table, the Atoms column lists the number of modelled atoms in the group and the number defined in the chemical component dictionary. The B-factors column lists the minimum, median, 95th percentile and maximum values of B factors of atoms in the group. The column labelled 'Q< 0.9' lists the number of atoms with occupancy less than 0.9.

Mol	Type	Chain	Res	Atoms	RSCC	RSR	B-factors($Å^2$)	Q<0.9
2	MPD	A	503	8/8	0.67	0.29	68,72,72,77	2
2	MPD	A	504	8/8	0.67	0.28	60,70,73,73	2
2	MPD	A	502	8/8	0.83	0.15	68,75,78,84	2
2	MPD	B	502	8/8	0.91	0.16	63,68,72,72	2
2	MPD	B	501	8/8	0.93	0.10	49,53,57,58	2
2	MPD	A	501	8/8	0.93	0.14	49,56,61,61	2
4	SF4	A	508	8/8	0.95	0.10	33,40,43,46	0
4	SF4	A	507	8/8	0.97	0.12	33,37,39,41	0
3	402	B	504	17/17	0.98	0.12	22,31,37,37	1
4	SF4	A	506	8/8	0.98	0.15	27,28,29,30	0
4	SF4	B	503	8/8	0.98	0.14	26,27,28,29	0
4	SF4	B	505	8/8	0.98	0.14	29,31,33,33	0
3	402	A	505	17/17	0.99	0.11	21,26,28,29	1

Continued on next page...

For Manuscript Review

Continued from previous page...

Mol	Type	Chain	Res	Atoms	RSCC	RSR	B-factors(Å^2)	Q<0.9
4	SF4	B	506	8/8	0.99	0.14	26,27,28,29	0

The following is a graphical depiction of the model fit to experimental electron density of all instances of the Ligand of Interest. In addition, ligands with molecular weight > 250 and outliers as shown on the geometry validation Tables will also be included. Each fit is shown from different orientation to approximate a three-dimensional view.

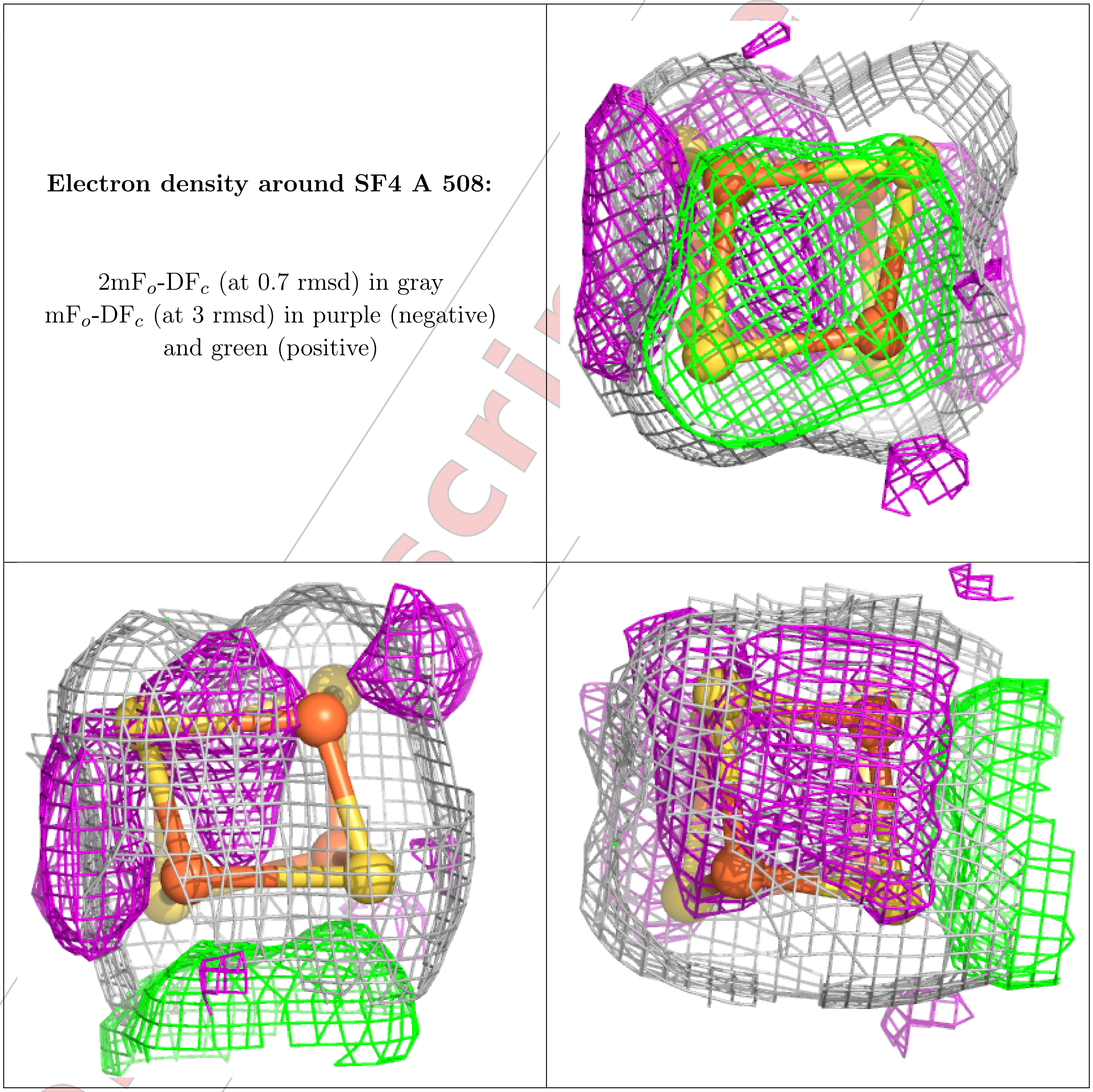

Electron density around SF4 A 508:

2mF$_o$-DF$_c$ (at 0.7 rmsd) in gray
mF$_o$-DF$_c$ (at 3 rmsd) in purple (negative)
and green (positive)

For Manuscript Review

Electron density around SF4 A 507:

2mF$_o$-DF$_c$ (at 0.7 rmsd) in gray
mF$_o$-DF$_c$ (at 3 rmsd) in purple (negative)
and green (positive)

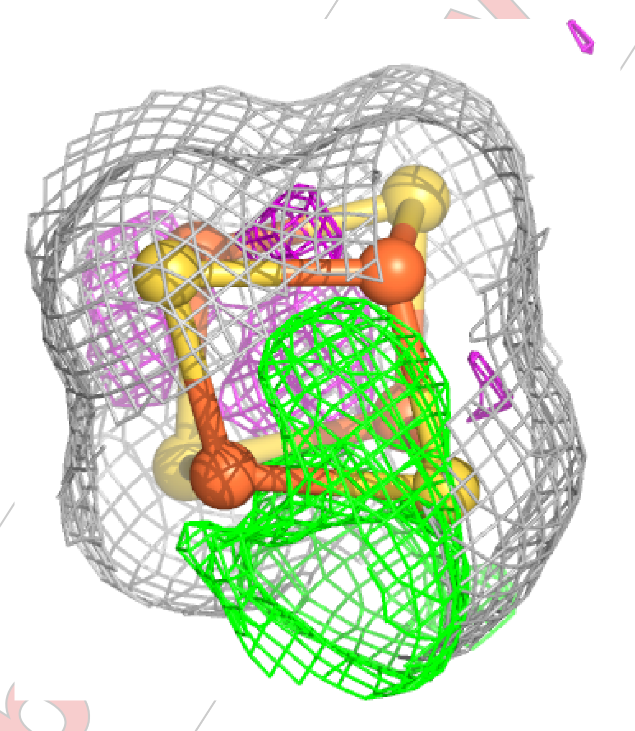

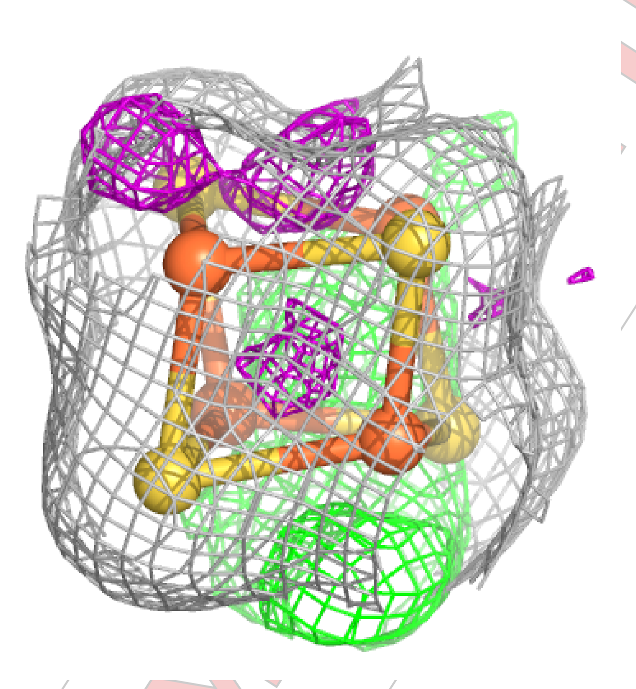

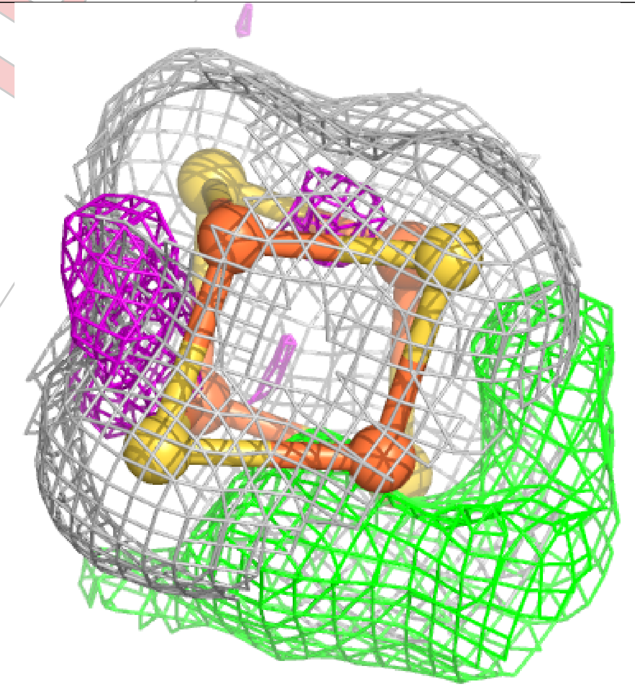

Electron density around 402 B 504:

2mF$_o$-DF$_c$ (at 0.7 rmsd) in gray
mF$_o$-DF$_c$ (at 3 rmsd) in purple (negative)
and green (positive)

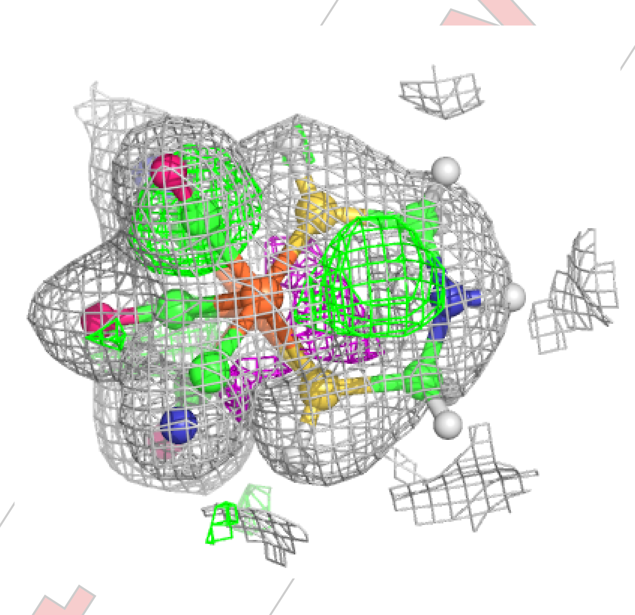

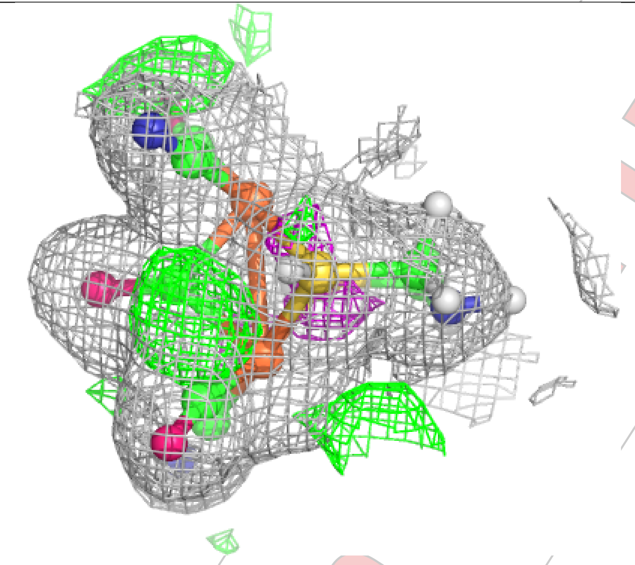

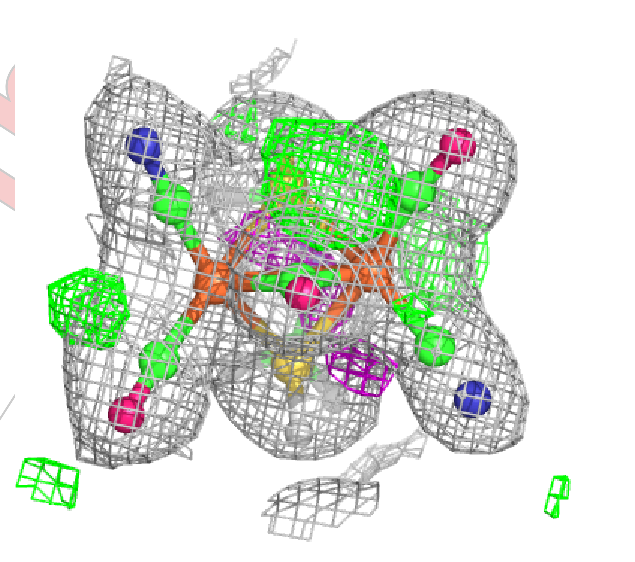

For Manuscript Review

Electron density around SF4 A 506:

$2mF_o$-DF_c (at 0.7 rmsd) in gray
mF_o-DF_c (at 3 rmsd) in purple (negative)
and green (positive)

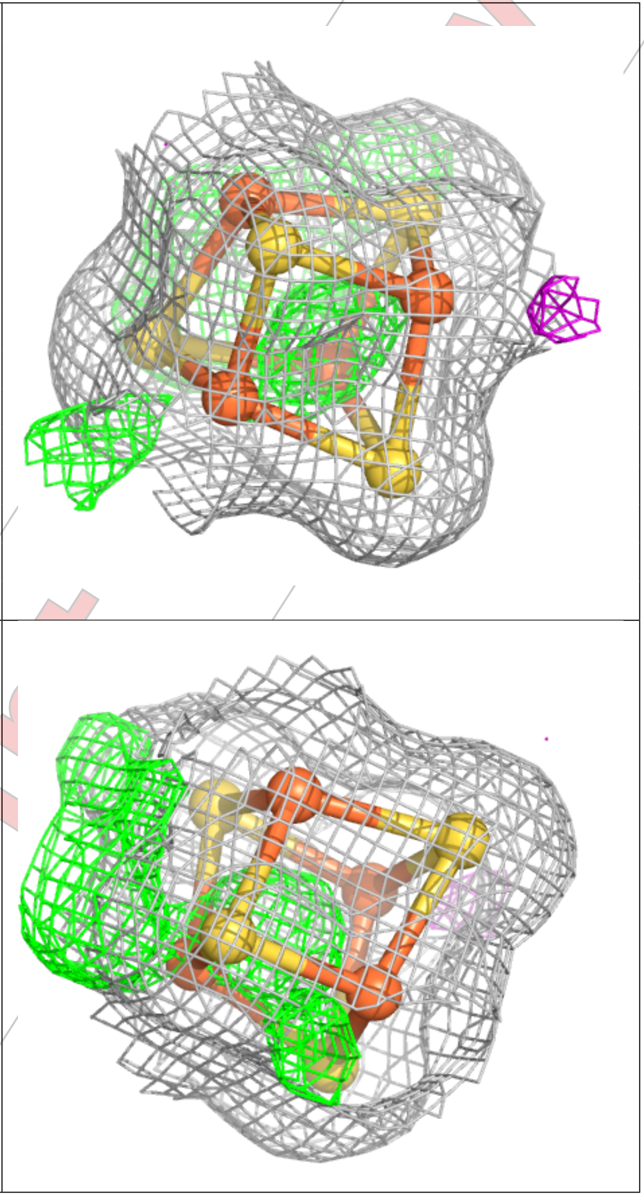

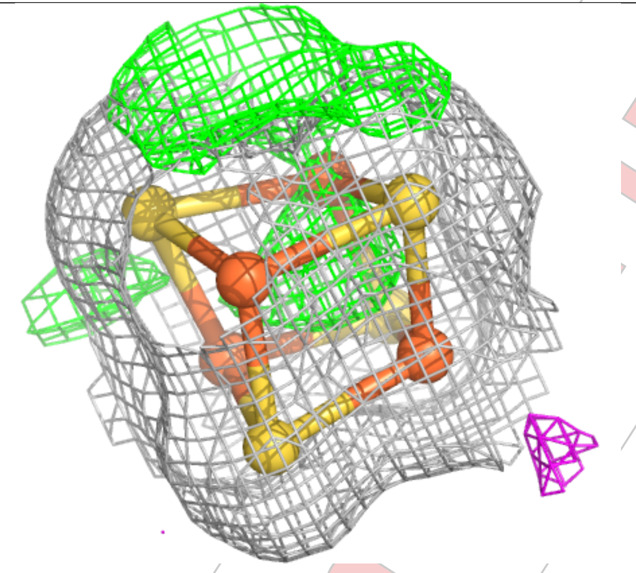

For Manuscript Review

Electron density around SF4 B 503:

2mF$_o$-DF$_c$ (at 0.7 rmsd) in gray
mF$_o$-DF$_c$ (at 3 rmsd) in purple (negative)
and green (positive)

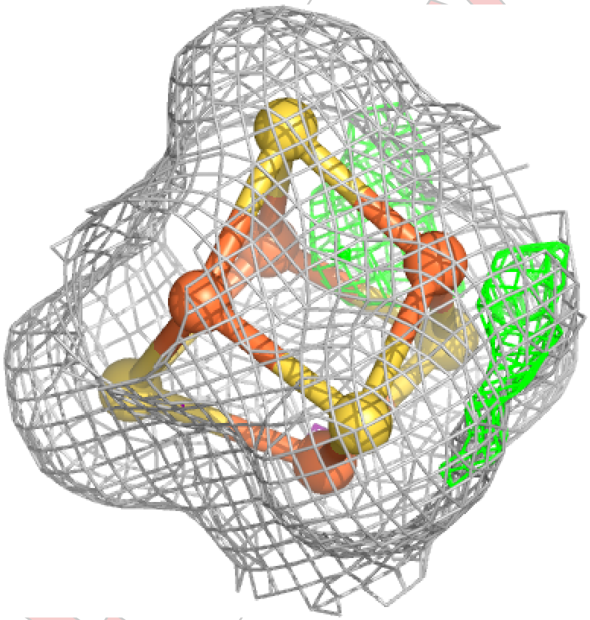

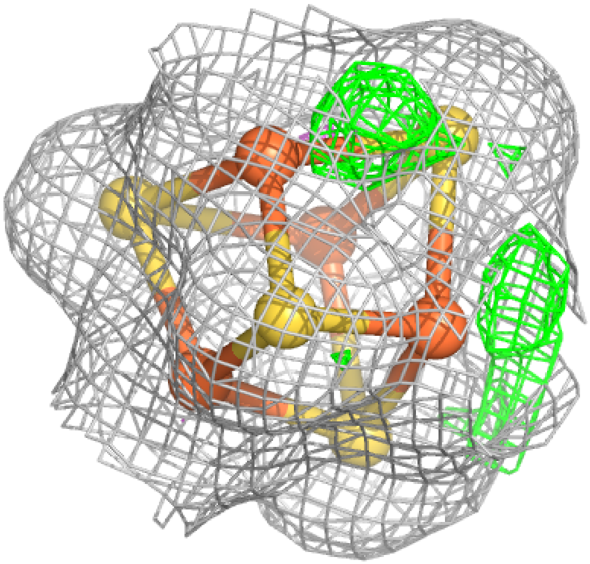

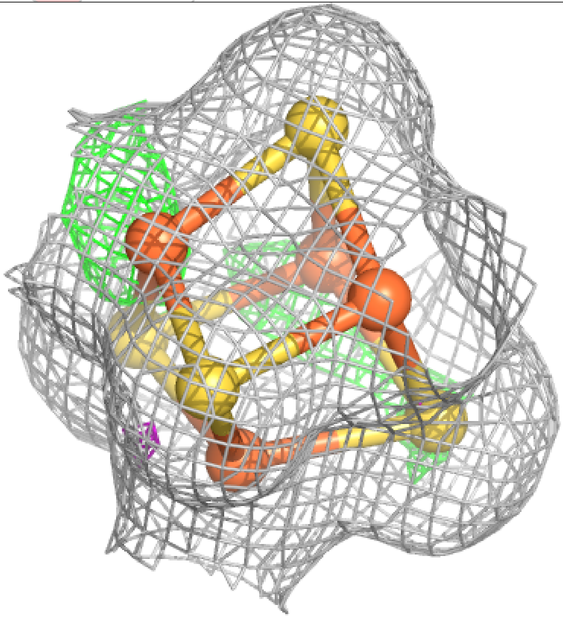

For Manuscript Review

Electron density around SF4 B 505:

$2mF_o$-DF_c (at 0.7 rmsd) in gray
mF_o-DF_c (at 3 rmsd) in purple (negative)
and green (positive)

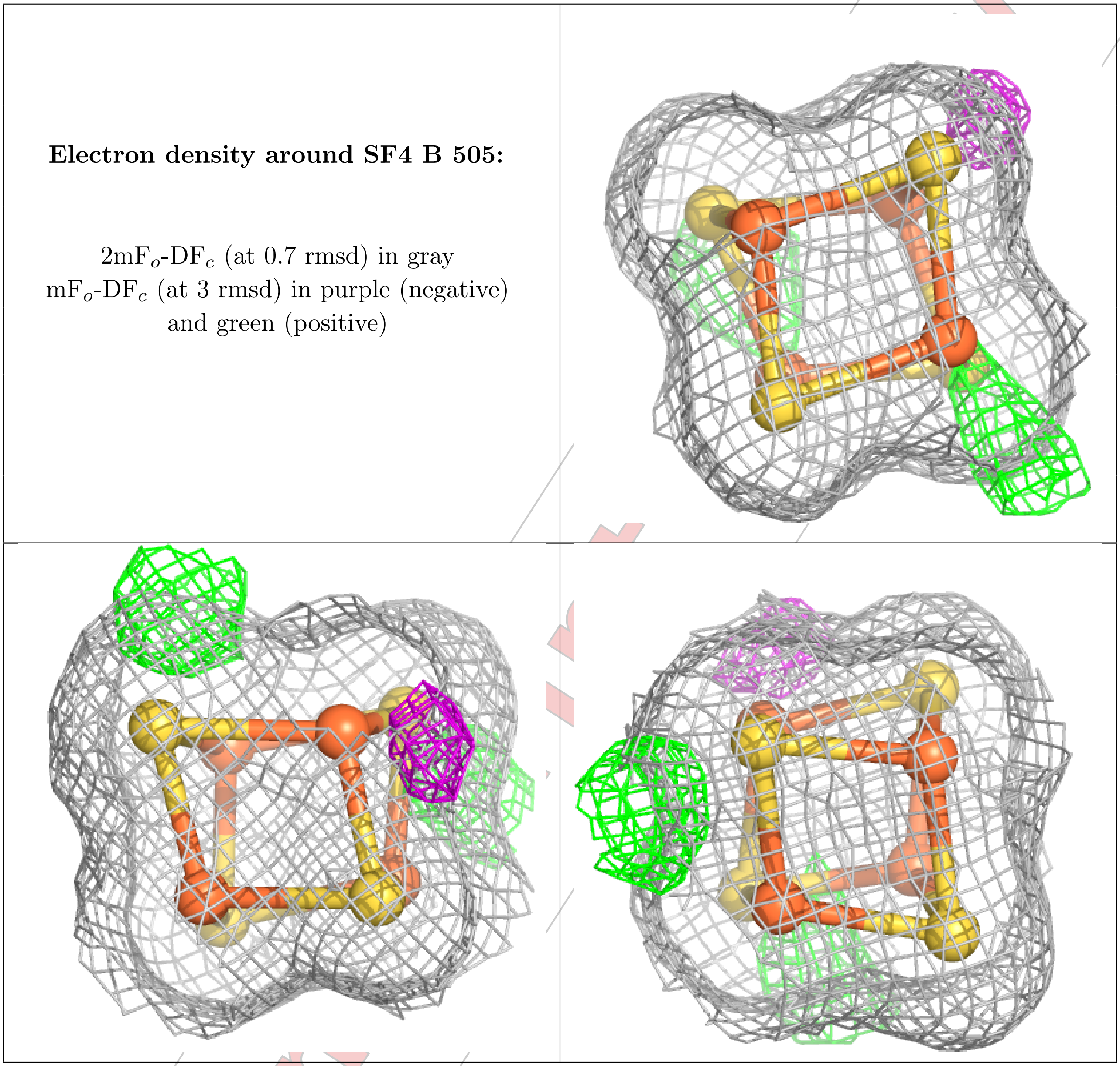

For Manuscript Review

Electron density around 402 A 505:

$2mF_o$-DF_c (at 0.7 rmsd) in gray
mF_o-DF_c (at 3 rmsd) in purple (negative) and green (positive)

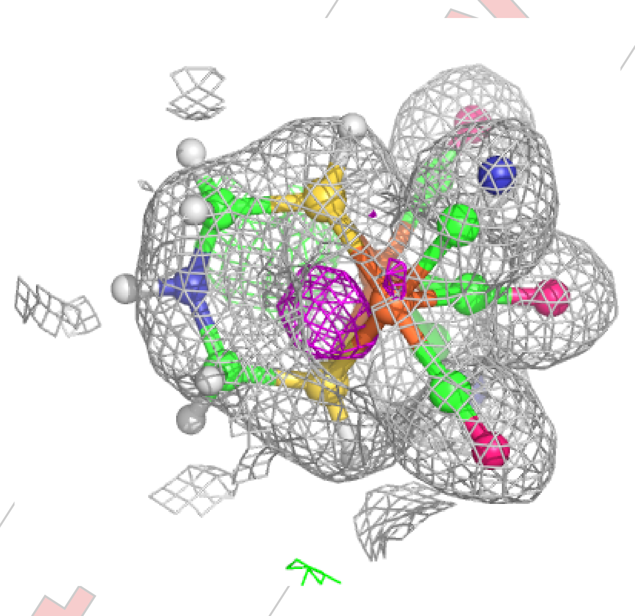

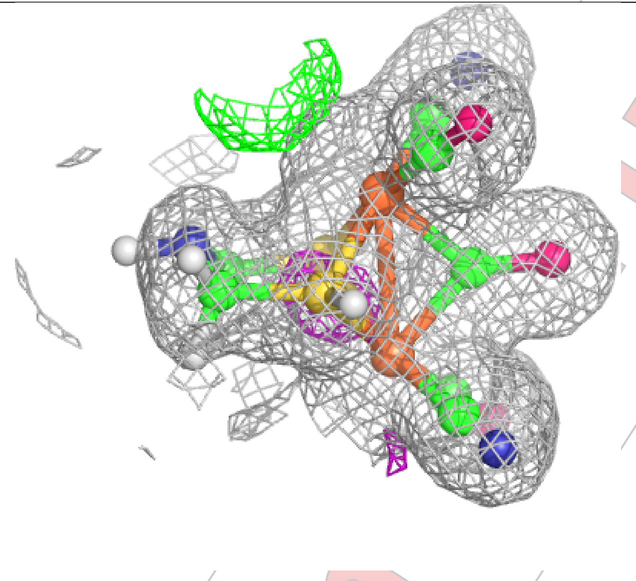

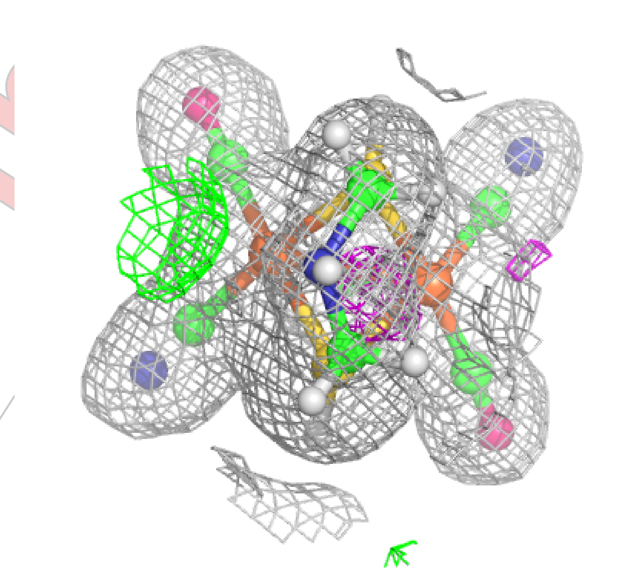

For Manuscript Review

Electron density around SF4 B 506:

$2mF_o$-DF_c (at 0.7 rmsd) in gray
mF_o-DF_c (at 3 rmsd) in purple (negative)
and green (positive)

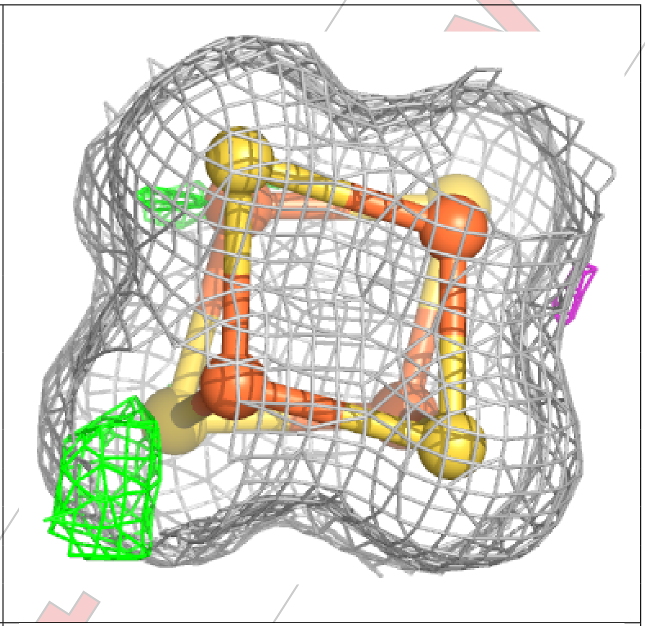

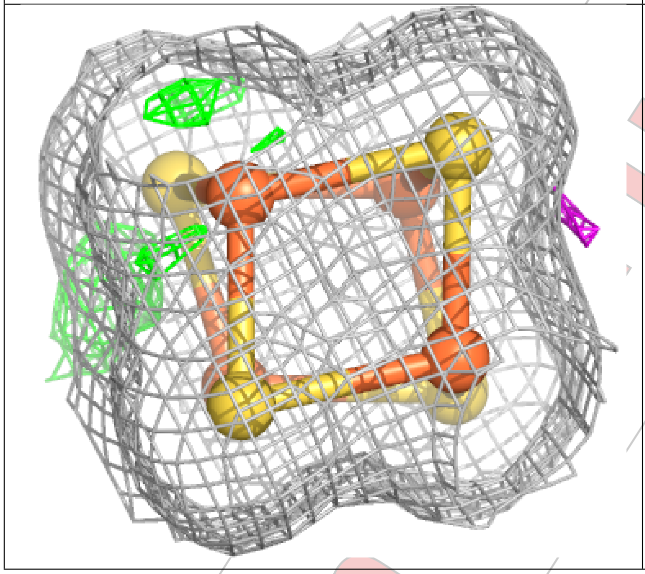

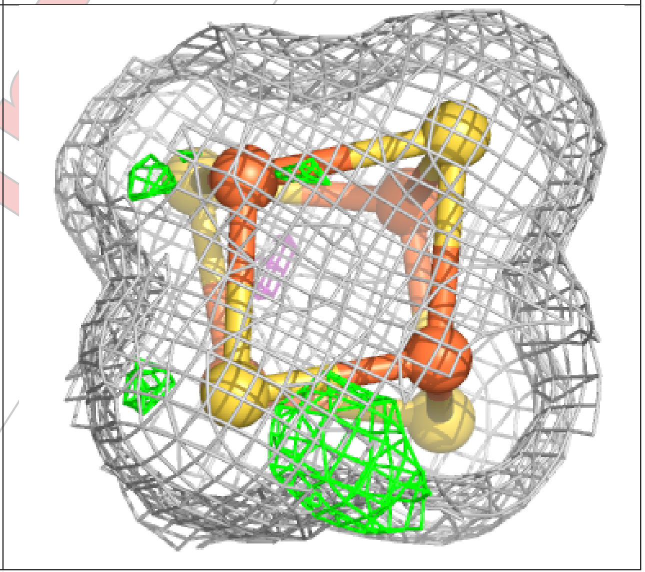

6.5 Other polymers (i)

There are no such residues in this entry.

For Manuscript Review

Full wwPDB X-ray Structure Validation Report (i)

Feb 13, 2024 – 12:08 am GMT

PDB ID	:	8RYH
Title	:	Desulfovibrio desulfuricans [FeFe]-hydrogenase variant with both subunits linked by a linker peptide derived from a group A1 type [FeFe]-hydrogenase of Veillonella atypica
Deposited on	:	2024-02-08
Resolution	:	1.77 Å(reported)

This wwPDB validation report is for manuscript review

This is a Full wwPDB X-ray Structure Validation Report.

This report is produced by the wwPDB biocuration pipeline after annotation of the structure.

We welcome your comments at *validation@mail.wwpdb.org*
A user guide is available at
https://www.wwpdb.org/validation/2017/XrayValidationReportHelp
with specific help available everywhere you see the (i) symbol.

The types of validation reports are described at
http://www.wwpdb.org/validation/2017/FAQs#types.

The following versions of software and data (see references (i)) were used in the production of this report:

MolProbity	:	4.02b-467
Mogul	:	1.8.4, CSD as541be (2020)
Xtriage (Phenix)	:	1.13
EDS	:	2.36
buster-report	:	1.1.7 (2018)
Percentile statistics	:	20191225.v01 (using entries in the PDB archive December 25th 2019)
Refmac	:	5.8.0158
CCP4	:	7.0.044 (Gargrove)

For Manuscript Review

1 Overall quality at a glance (i)

The following experimental techniques were used to determine the structure:
X-RAY DIFFRACTION

The reported resolution of this entry is 1.77 Å.

Percentile scores (ranging between 0-100) for global validation metrics of the entry are shown in the following graphic. The table shows the number of entries on which the scores are based.

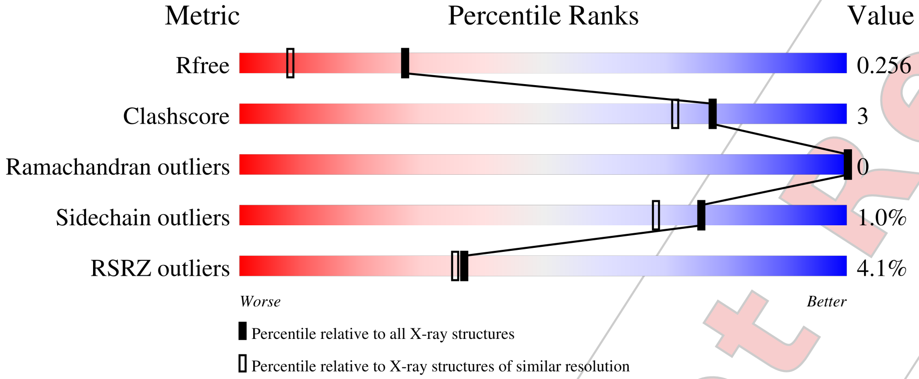

Metric	Whole archive (#Entries)	Similar resolution (#Entries, resolution range(Å))
R_{free}	130704	9185 (1.80-1.76)
Clashscore	141614	10184 (1.80-1.76)
Ramachandran outliers	138981	10051 (1.80-1.76)
Sidechain outliers	138945	10050 (1.80-1.76)
RSRZ outliers	127900	9032 (1.80-1.76)

The table below summarises the geometric issues observed across the polymeric chains and their fit to the electron density. The red, orange, yellow and green segments of the lower bar indicate the fraction of residues that contain outliers for >=3, 2, 1 and 0 types of geometric quality criteria respectively. A grey segment represents the fraction of residues that are not modelled. The numeric value for each fraction is indicated below the corresponding segment, with a dot representing fractions <=5% The upper red bar (where present) indicates the fraction of residues that have poor fit to the electron density. The numeric value is given above the bar.

Mol	Chain	Length	Quality of chain
1	A	490	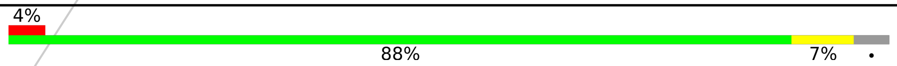

Ideal geometry (proteins) : Engh & Huber (2001)
Ideal geometry (DNA, RNA) : Parkinson et al. (1996)
Validation Pipeline (wwPDB-VP) : 2.36

For Manuscript Review

2 Entry composition (i)

There are 4 unique types of molecules in this entry. The entry contains 7374 atoms, of which 3595 are hydrogens and 0 are deuteriums.

In the tables below, the ZeroOcc column contains the number of atoms modelled with zero occupancy, the AltConf column contains the number of residues with at least one atom in alternate conformation and the Trace column contains the number of residues modelled with at most 2 atoms.

- Molecule 1 is a protein called Periplasmic [Fe] hydrogenase large subunit,Periplasmic [Fe] hydrogenase small subunit.

Mol	Chain	Residues	Atoms						ZeroOcc	AltConf	Trace
			Total	C	H	N	O	S			
1	A	469	7209	2297	3588	615	676	33	117	3	0

There are 23 discrepancies between the modelled and reference sequences:

Chain	Residue	Modelled	Actual	Comment	Reference
A	390	ARG	-	linker	UNP P07598
A	391	VAL	-	linker	UNP P07598
A	392	LYS	-	linker	UNP P07598
A	393	LEU	-	linker	UNP P07598
A	394	PRO	-	linker	UNP P07598
A	395	MET	-	linker	UNP P07598
A	396	ALA	-	linker	UNP P07598
A	397	ASP	-	linker	UNP P07598
A	398	LYS	-	linker	UNP P07598
A	399	ALA	-	linker	UNP P07598
A	400	ARG	-	linker	UNP P07598
A	401	GLU	-	linker	UNP P07598
A	402	ALA	-	linker	UNP P07598
A	481	SER	-	expression tag	UNP P07603
A	482	ALA	-	expression tag	UNP P07603
A	483	TRP	-	expression tag	UNP P07603
A	484	SER	-	expression tag	UNP P07603
A	485	HIS	-	expression tag	UNP P07603
A	486	PRO	-	expression tag	UNP P07603
A	487	GLN	-	expression tag	UNP P07603
A	488	PHE	-	expression tag	UNP P07603
A	489	GLU	-	expression tag	UNP P07603
A	490	LYS	-	expression tag	UNP P07603

- Molecule 2 is IRON/SULFUR CLUSTER (three-letter code: SF4) (formula: Fe_4S_4) (labeled as "Ligand of Interest" by depositor).

For Manuscript Review

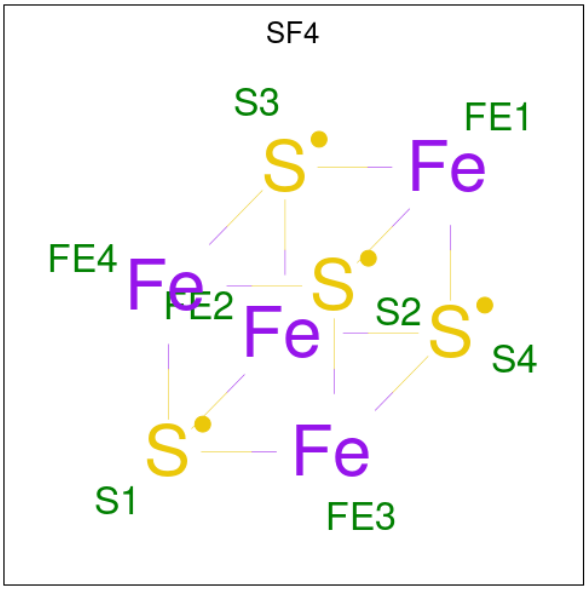

Mol	Chain	Residues	Atoms	ZeroOcc	AltConf
2	A	1	Total Fe S<br>8 4 4	0	0
2	A	1	Total Fe S<br>8 4 4	0	0
2	A	1	Total Fe S<br>8 4 4	0	0

- Molecule 3 is dicarbonyl[bis(cyanide-kappaC)]-mu-(iminodimethanethiolatato-1kappaS:2kappaS)-mu-(oxomethylidene)diiron(2+) (three-letter code: 402) (formula: $C_7H_5Fe_2N_3O_3S_2$) (labeled as "Ligand of Interest" by depositor).

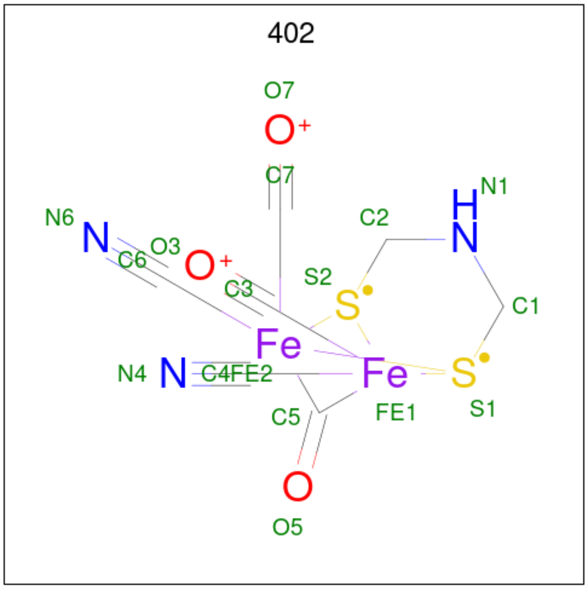

For Manuscript Review

Mol	Chain	Residues	Atoms							ZeroOcc	AltConf
			Total	C	Fe	H	N	O	S		
3	A	1	24	7	2	7	3	3	2	1	0

- Molecule 4 is water.

Mol	Chain	Residues	Atoms		ZeroOcc	AltConf
			Total	O		
4	A	117	117	117	0	0

For Manuscript Review

3 Residue-property plots (i)

These plots are drawn for all protein, RNA, DNA and oligosaccharide chains in the entry. The first graphic for a chain summarises the proportions of the various outlier classes displayed in the second graphic. The second graphic shows the sequence view annotated by issues in geometry and electron density. Residues are color-coded according to the number of geometric quality criteria for which they contain at least one outlier: green = 0, yellow = 1, orange = 2 and red = 3 or more. A red dot above a residue indicates a poor fit to the electron density (RSRZ > 2). Stretches of 2 or more consecutive residues without any outlier are shown as a green connector. Residues present in the sample, but not in the model, are shown in grey.

• Molecule 1: Periplasmic [Fe] hydrogenase large subunit,Periplasmic [Fe] hydrogenase small subunit

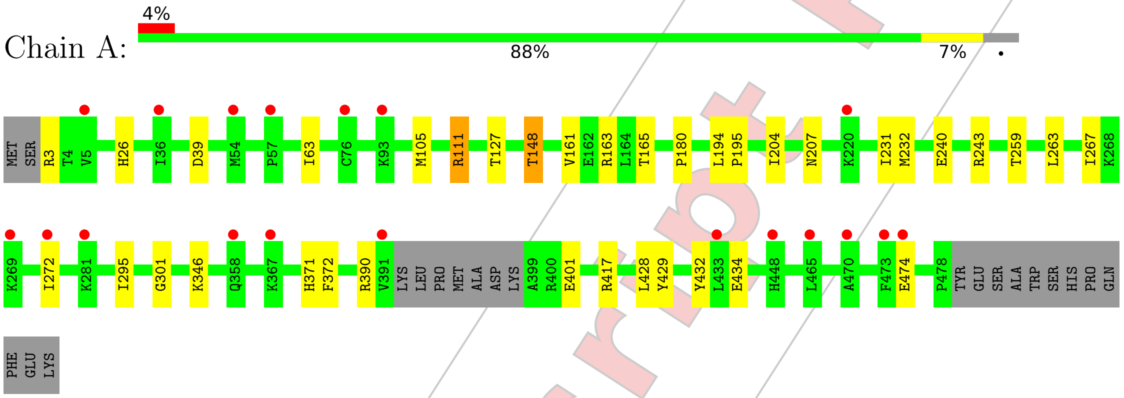

For Manuscript Review

4 Data and refinement statistics (i)

Property	Value	Source
Space group	C 1 2 1	Depositor
Cell constants<br>a, b, c, α, β, γ	106.12Å 50.60Å 86.64Å<br>90.00° 105.33° 90.00°	Depositor
Resolution (Å)	45.40 – 1.77<br>45.36 – 1.77	Depositor<br>EDS
% Data completeness<br>(in resolution range)	96.3 (45.40-1.77)<br>96.3 (45.36-1.77)	Depositor<br>EDS
R_{merge}	0.16	Depositor
R_{sym}	(Not available)	Depositor
$< I/\sigma(I) >$ [1]	2.56 (at 1.77Å)	Xtriage
Refinement program	REFMAC 5.8.0425	Depositor
R, R_{free}	0.205 , 0.246<br>0.211 , 0.256	Depositor<br>DCC
R_{free} test set	2085 reflections (5.01%)	wwPDB-VP
Wilson B-factor (Å^2)	15.8	Xtriage
Anisotropy	0.149	Xtriage
Bulk solvent k_{sol}(e/Å^3), B_{sol}(Å^2)	0.34 , 36.7	EDS
L-test for twinning[2]	$< \|L\| > = 0.48$, $< L^2 > = 0.31$	Xtriage
Estimated twinning fraction	No twinning to report.	Xtriage
F_o,F_c correlation	0.93	EDS
Total number of atoms	7374	wwPDB-VP
Average B, all atoms (Å^2)	20.0	wwPDB-VP

Xtriage's analysis on translational NCS is as follows: *The largest off-origin peak in the Patterson function is 6.78% of the height of the origin peak. No significant pseudotranslation is detected.*

[1] Intensities estimated from amplitudes.
[2] Theoretical values of $< |L| >$, $< L^2 >$ for acentric reflections are 0.5, 0.333 respectively for untwinned datasets, and 0.375, 0.2 for perfectly twinned datasets.

For Manuscript Review

5 Model quality (i)

5.1 Standard geometry (i)

Bond lengths and bond angles in the following residue types are not validated in this section: SF4, 402

The Z score for a bond length (or angle) is the number of standard deviations the observed value is removed from the expected value. A bond length (or angle) with $|Z| > 5$ is considered an outlier worth inspection. RMSZ is the root-mean-square of all Z scores of the bond lengths (or angles).

Mol	Chain	Bond lengths		Bond angles	
		RMSZ	#$\|Z\|$ >5	RMSZ	#$\|Z\|$ >5
1	A	0.43	0/3721	0.81	3/5031 (0.1%)

Chiral center outliers are detected by calculating the chiral volume of a chiral center and verifying if the center is modelled as a planar moiety or with the opposite hand.A planarity outlier is detected by checking planarity of atoms in a peptide group, atoms in a mainchain group or atoms of a sidechain that are expected to be planar.

Mol	Chain	#Chirality outliers	#Planarity outliers
1	A	0	2

There are no bond length outliers.

All (3) bond angle outliers are listed below:

Mol	Chain	Res	Type	Atoms	Z	Observed(^{o})	Ideal(^{o})
1	A	3	ARG	NE-CZ-NH2	-7.47	116.56	120.30
1	A	111	ARG	CB-CA-C	-7.14	96.11	110.40
1	A	163	ARG	NE-CZ-NH2	-5.56	117.52	120.30

There are no chirality outliers.

All (2) planarity outliers are listed below:

Mol	Chain	Res	Type	Group
1	A	243	ARG	Sidechain
1	A	417	ARG	Sidechain

5.2 Too-close contacts (i)

In the following table, the Non-H and H(model) columns list the number of non-hydrogen atoms and hydrogen atoms in the chain respectively. The H(added) column lists the number of hydrogen

For Manuscript Review

atoms added and optimized by MolProbity. The Clashes column lists the number of clashes within the asymmetric unit, whereas Symm-Clashes lists symmetry-related clashes.

Mol	Chain	Non-H	H(model)	H(added)	Clashes	Symm-Clashes
1	A	3621	3588	3558	21	0
2	A	24	0	0	1	0
3	A	17	7	5	0	0
4	A	117	0	0	0	0
All	All	3779	3595	3563	21	0

The all-atom clashscore is defined as the number of clashes found per 1000 atoms (including hydrogen atoms). The all-atom clashscore for this structure is 3.

All (21) close contacts within the same asymmetric unit are listed below, sorted by their clash magnitude.

Atom-1	Atom-2	Interatomic distance (Å)	Clash overlap (Å)
1:A:474:GLU:O	1:A:474:GLU:HG3	1.44	1.13
1:A:346:LYS:H	1:A:371:HIS:HD2	1.31	0.78
1:A:474:GLU:O	1:A:474:GLU:CG	2.20	0.76
1:A:111:ARG:HG2	1:A:127:THR:OG1	1.94	0.68
1:A:26:HIS:CD2	1:A:63:ILE:HG22	2.37	0.60
1:A:26:HIS:CD2	1:A:63:ILE:CG2	2.91	0.53
1:A:295:ILE:O	1:A:301:GLY:HA3	2.10	0.52
1:A:105:MET:HE3	1:A:204:ILE:HD11	1.93	0.51
1:A:180:PRO:HB3	1:A:240:GLU:HB2	1.95	0.48
1:A:161:VAL:O	1:A:165:THR:HG23	2.14	0.48
1:A:194:LEU:N	1:A:195:PRO:CD	2.79	0.46
1:A:267:ILE:HG23	1:A:272:ILE:HB	1.98	0.46
1:A:180:PRO:HD2	2:A:503:SF4:S2	2.56	0.45
1:A:429:TYR:O	1:A:432:TYR:O	2.34	0.45
1:A:346:LYS:H	1:A:371:HIS:CD2	2.22	0.43
1:A:432:TYR:O	1:A:434:GLU:N	2.48	0.43
1:A:148:THR:HG21	1:A:207:ASN:HA	2.01	0.42
1:A:231:ILE:HG21	1:A:263:LEU:HD22	2.01	0.42
1:A:401:GLU:N	1:A:401:GLU:OE1	2.52	0.42
1:A:232:MET:O	1:A:259:THR:HA	2.20	0.42
1:A:428:LEU:O	1:A:432:TYR:O	2.38	0.41

There are no symmetry-related clashes.

For Manuscript Review

5.3 Torsion angles (i)

5.3.1 Protein backbone (i)

In the following table, the Percentiles column shows the percent Ramachandran outliers of the chain as a percentile score with respect to all X-ray entries followed by that with respect to entries of similar resolution.

The Analysed column shows the number of residues for which the backbone conformation was analysed, and the total number of residues.

Mol	Chain	Analysed	Favoured	Allowed	Outliers	Percentiles	
1	A	468/490 (96%)	458 (98%)	10 (2%)	0	100	100

There are no Ramachandran outliers to report.

5.3.2 Protein sidechains (i)

In the following table, the Percentiles column shows the percent sidechain outliers of the chain as a percentile score with respect to all X-ray entries followed by that with respect to entries of similar resolution.

The Analysed column shows the number of residues for which the sidechain conformation was analysed, and the total number of residues.

Mol	Chain	Analysed	Rotameric	Outliers	Percentiles	
1	A	384/401 (96%)	380 (99%)	4 (1%)	76	68

All (4) residues with a non-rotameric sidechain are listed below:

Mol	Chain	Res	Type
1	A	39	ASP
1	A	148	THR
1	A	372	PHE
1	A	390	ARG

Sometimes sidechains can be flipped to improve hydrogen bonding and reduce clashes. All (3) such sidechains are listed below:

Mol	Chain	Res	Type
1	A	196	HIS
1	A	371	HIS
1	A	405	ASN

For Manuscript Review

5.3.3 RNA (i)

There are no RNA molecules in this entry.

5.4 Non-standard residues in protein, DNA, RNA chains (i)

There are no non-standard protein/DNA/RNA residues in this entry.

5.5 Carbohydrates (i)

There are no monosaccharides in this entry.

5.6 Ligand geometry (i)

4 ligands are modelled in this entry.

In the following table, the Counts columns list the number of bonds (or angles) for which Mogul statistics could be retrieved, the number of bonds (or angles) that are observed in the model and the number of bonds (or angles) that are defined in the Chemical Component Dictionary. The Link column lists molecule types, if any, to which the group is linked. The Z score for a bond length (or angle) is the number of standard deviations the observed value is removed from the expected value. A bond length (or angle) with $|Z| > 2$ is considered an outlier worth inspection. RMSZ is the root-mean-square of all Z scores of the bond lengths (or angles).

Mol	Type	Chain	Res	Link	Bond lengths			Bond angles		
					Counts	RMSZ	$\#\|Z\| > 2$	Counts	RMSZ	$\#\|Z\| > 2$
2	SF4	A	501	1	0,12,12	-	-	-		
2	SF4	A	502	1	0,12,12	-	-	-		
3	402	A	504	1	13,19,19	4.90	6 (46%)	2,36,36	0.71	0
2	SF4	A	503	1	0,12,12	-	-	-		

In the following table, the Chirals column lists the number of chiral outliers, the number of chiral centers analysed, the number of these observed in the model and the number defined in the Chemical Component Dictionary. Similar counts are reported in the Torsion and Rings columns. '-' means no outliers of that kind were identified.

Mol	Type	Chain	Res	Link	Chirals	Torsions	Rings
2	SF4	A	501	1	-	-	0/6/5/5
2	SF4	A	502	1	-	-	0/6/5/5
3	402	A	504	1	-	-	0/5/3/3
2	SF4	A	503	1	-	-	0/6/5/5

All (6) bond length outliers are listed below:

For Manuscript Review

Mol	Chain	Res	Type	Atoms	Z	Observed(Å)	Ideal(Å)
3	A	504	402	C1-S1	-10.96	1.65	1.85
3	A	504	402	C2-S2	-9.62	1.67	1.85
3	A	504	402	O5-C5	5.79	1.27	1.17
3	A	504	402	S2-FE1	-5.22	2.18	2.26
3	A	504	402	S2-FE2	-4.66	2.19	2.26
3	A	504	402	S1-FE2	-3.87	2.20	2.26

There are no bond angle outliers.

There are no chirality outliers.

There are no torsion outliers.

There are no ring outliers.

1 monomer is involved in 1 short contact:

Mol	Chain	Res	Type	Clashes	Symm-Clashes
2	A	503	SF4	1	0

The following is a two-dimensional graphical depiction of Mogul quality analysis of bond lengths, bond angles, torsion angles, and ring geometry for all instances of the Ligand of Interest. In addition, ligands with molecular weight > 250 and outliers as shown on the validation Tables will also be included. For torsion angles, if less then 5% of the Mogul distribution of torsion angles is within 10 degrees of the torsion angle in question, then that torsion angle is considered an outlier. Any bond that is central to one or more torsion angles identified as an outlier by Mogul will be highlighted in the graph. For rings, the root-mean-square deviation (RMSD) between the ring in question and similar rings identified by Mogul is calculated over all ring torsion angles. If the average RMSD is greater than 60 degrees and the minimal RMSD between the ring in question and any Mogul-identified rings is also greater than 60 degrees, then that ring is considered an outlier. The outliers are highlighted in purple. The color gray indicates Mogul did not find sufficient equivalents in the CSD to analyse the geometry.

For Manuscript Review

Ligand SF4 A 501

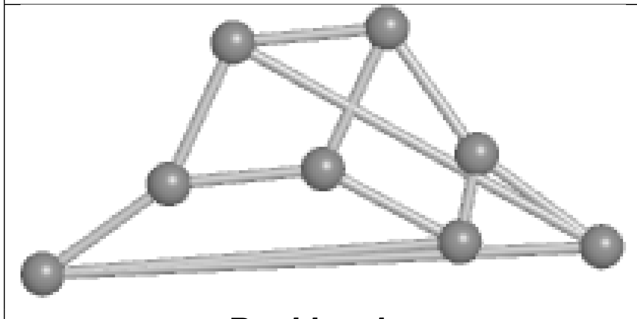

Bond lengths

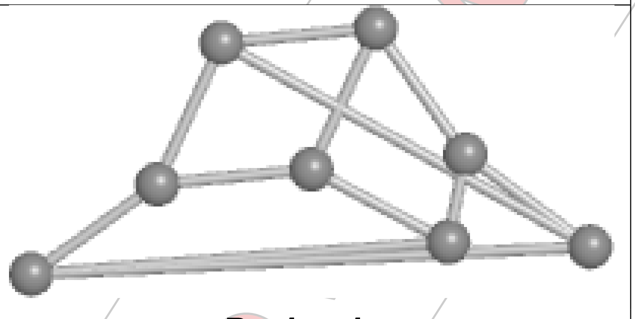

Bond angles

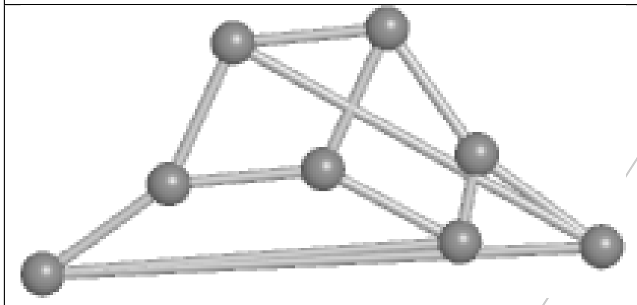

Torsions

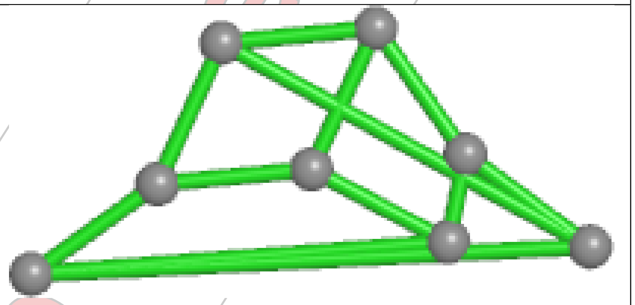

Rings

Ligand SF4 A 502

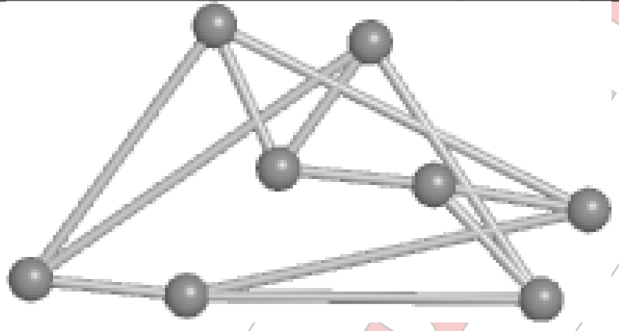

Bond lengths

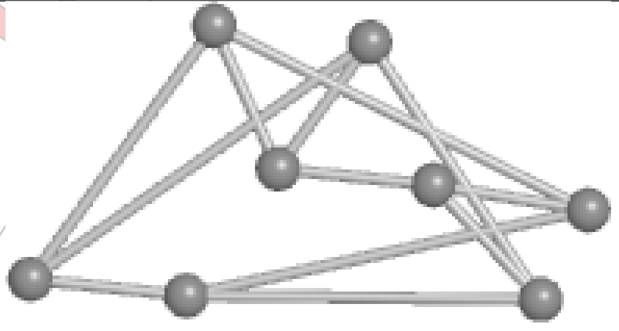

Bond angles

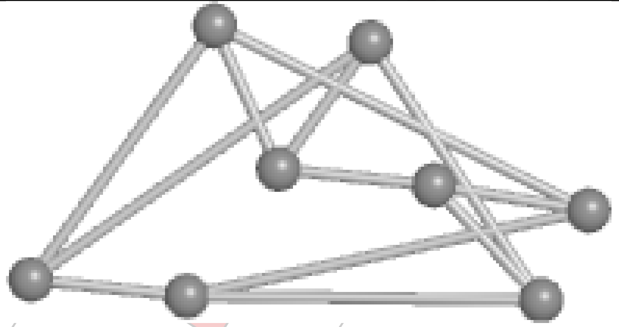

Torsions

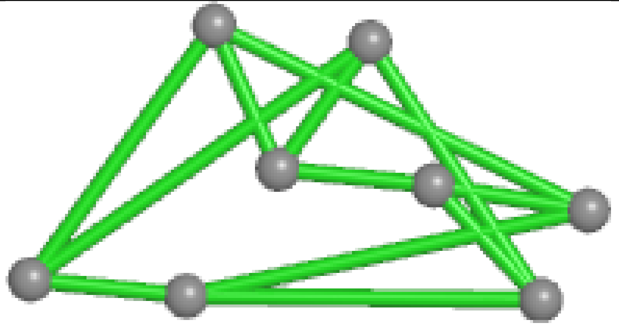

Rings

For Manuscript Review

Ligand 402 A 504

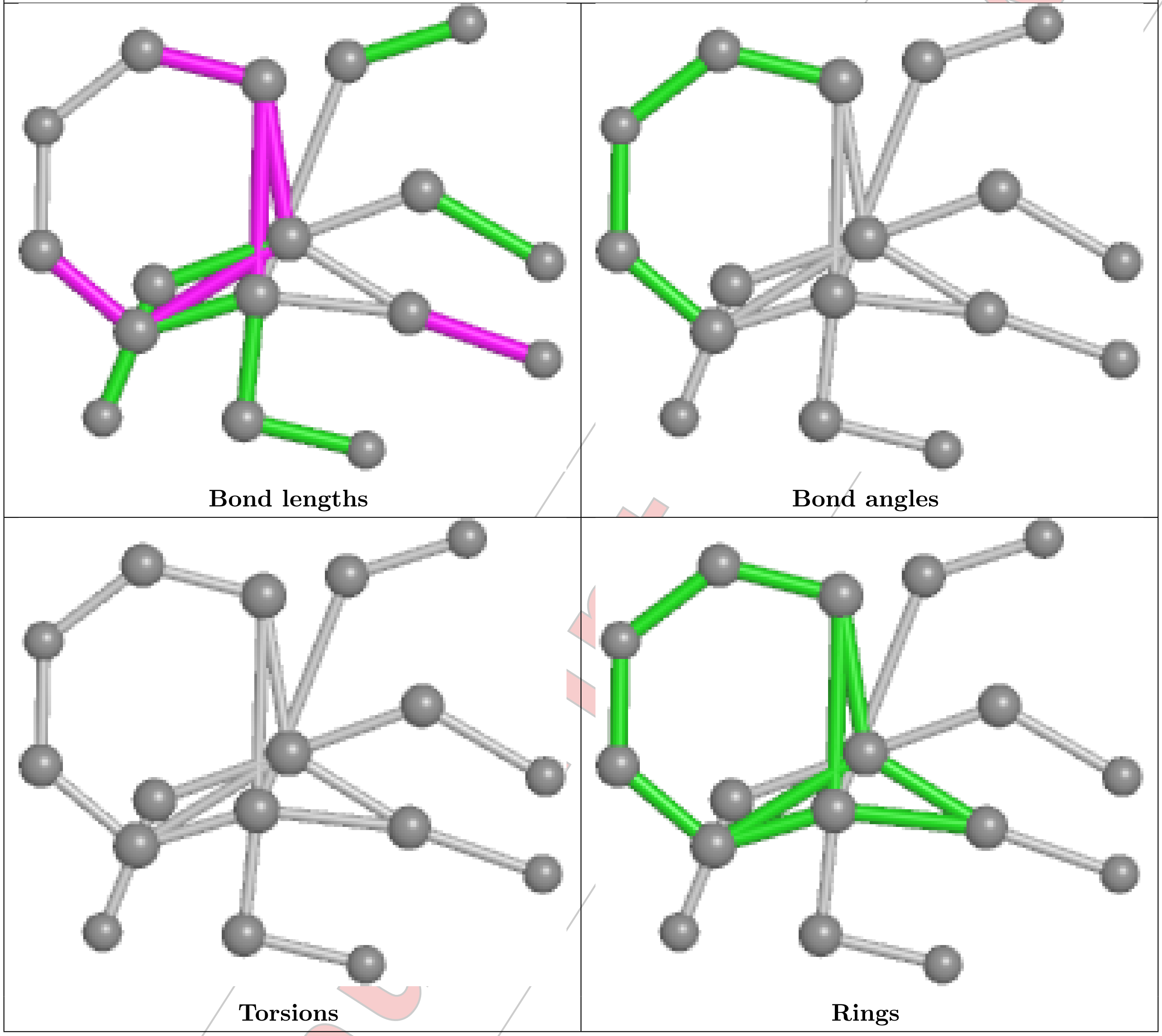

For Manuscript Review

Ligand SF4 A 503

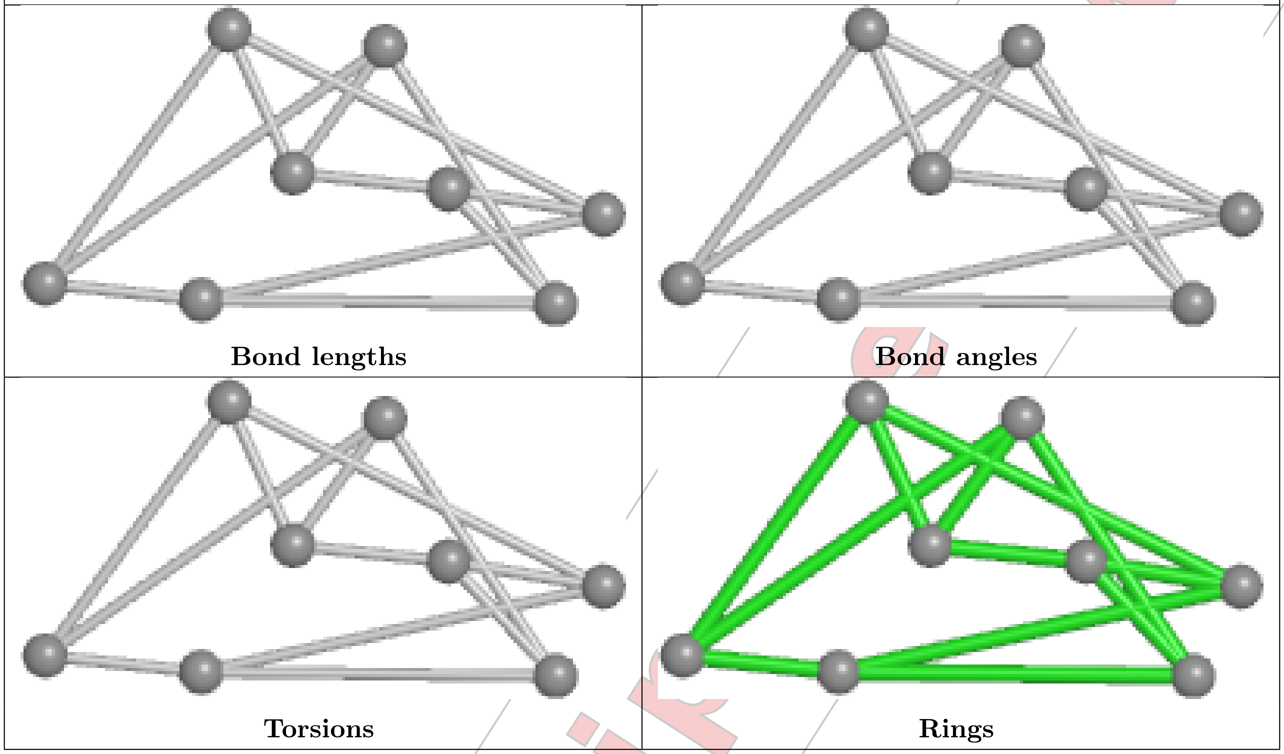

5.7 Other polymers (i)

There are no such residues in this entry.

5.8 Polymer linkage issues (i)

There are no chain breaks in this entry.

For Manuscript Review

6 Fit of model and data (i)

6.1 Protein, DNA and RNA chains (i)

In the following table, the column labelled '#RSRZ> 2' contains the number (and percentage) of RSRZ outliers, followed by percent RSRZ outliers for the chain as percentile scores relative to all X-ray entries and entries of similar resolution. The OWAB column contains the minimum, median, 95th percentile and maximum values of the occupancy-weighted average B-factor per residue. The column labelled 'Q< 0.9' lists the number of (and percentage) of residues with an average occupancy less than 0.9.

Mol	Chain	Analysed	<RSRZ>	#RSRZ>2			OWAB(Å^2)	Q<0.9
1	A	469/490 (95%)	0.40	19 (4%)	37	35	11, 19, 33, 59	0

All (19) RSRZ outliers are listed below:

Mol	Chain	Res	Type	RSRZ
1	A	391	VAL	6.5
1	A	367	LYS	4.6
1	A	54	MET	4.0
1	A	470	ALA	3.9
1	A	57	PRO	3.1
1	A	448[A]	HIS	3.0
1	A	36	ILE	3.0
1	A	76	CYS	2.7
1	A	93	LYS	2.7
1	A	272	ILE	2.5
1	A	5	VAL	2.5
1	A	358	GLN	2.5
1	A	473	PHE	2.4
1	A	281	LYS	2.3
1	A	433	LEU	2.2
1	A	474	GLU	2.1
1	A	465	LEU	2.1
1	A	220	LYS	2.1
1	A	269	LYS	2.1

6.2 Non-standard residues in protein, DNA, RNA chains (i)

There are no non-standard protein/DNA/RNA residues in this entry.

For Manuscript Review

6.3 Carbohydrates (i)

There are no monosaccharides in this entry.

6.4 Ligands (i)

In the following table, the Atoms column lists the number of modelled atoms in the group and the number defined in the chemical component dictionary. The B-factors column lists the minimum, median, 95th percentile and maximum values of B factors of atoms in the group. The column labelled 'Q< 0.9' lists the number of atoms with occupancy less than 0.9.

Mol	Type	Chain	Res	Atoms	RSCC	RSR	B-factors(Å^2)	Q<0.9
2	SF4	A	502	8/8	0.97	0.08	16,18,19,20	0
3	402	A	504	17/17	0.97	0.12	10,14,16,17	1
2	SF4	A	501	8/8	0.98	0.08	14,15,17,17	0
2	SF4	A	503	8/8	0.99	0.11	11,11,12,13	0

The following is a graphical depiction of the model fit to experimental electron density of all instances of the Ligand of Interest. In addition, ligands with molecular weight > 250 and outliers as shown on the geometry validation Tables will also be included. Each fit is shown from different orientation to approximate a three-dimensional view.

For Manuscript Review

Electron density around SF4 A 502:

2mF$_o$-DF$_c$ (at 0.7 rmsd) in gray
mF$_o$-DF$_c$ (at 3 rmsd) in purple (negative)
and green (positive)

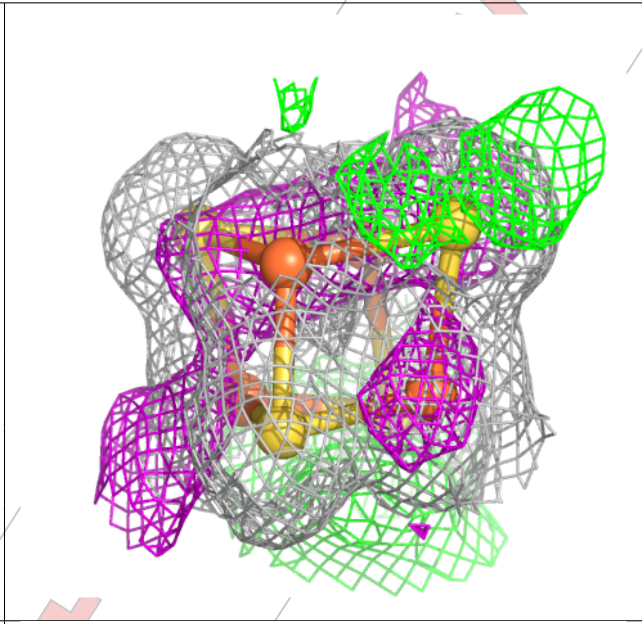

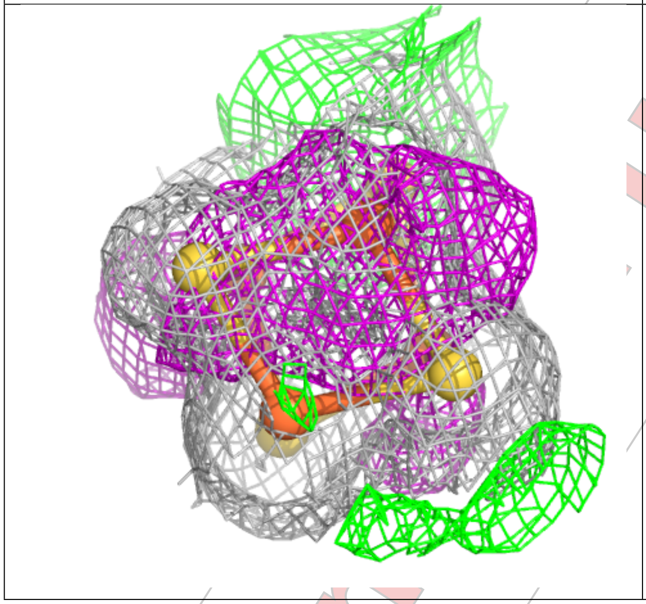

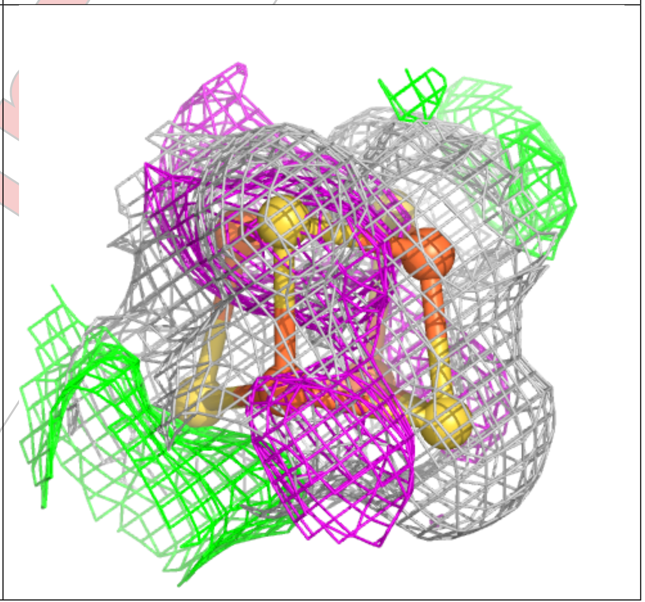

For Manuscript Review

Electron density around 402 A 504:

$2mF_o$-DF_c (at 0.7 rmsd) in gray
mF_o-DF_c (at 3 rmsd) in purple (negative)
and green (positive)

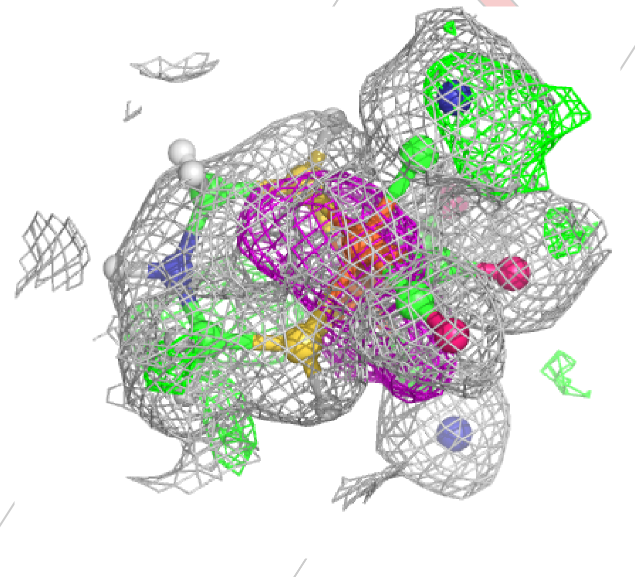

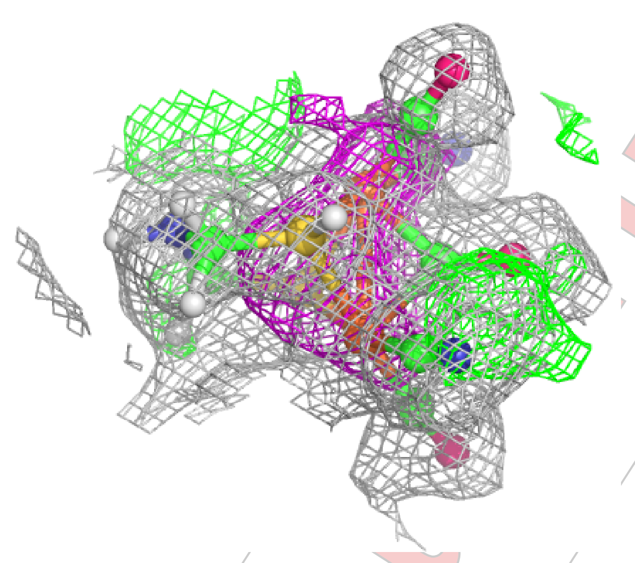

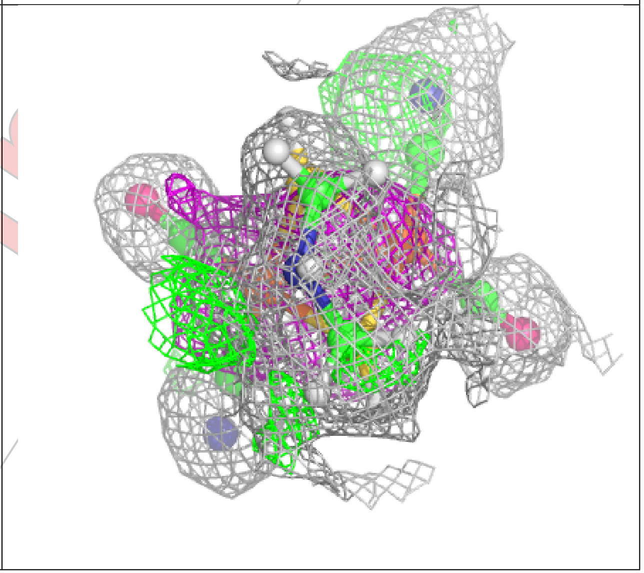

Electron density around SF4 A 501:

$2mF_o$-DF_c (at 0.7 rmsd) in gray
mF_o-DF_c (at 3 rmsd) in purple (negative)
and green (positive)

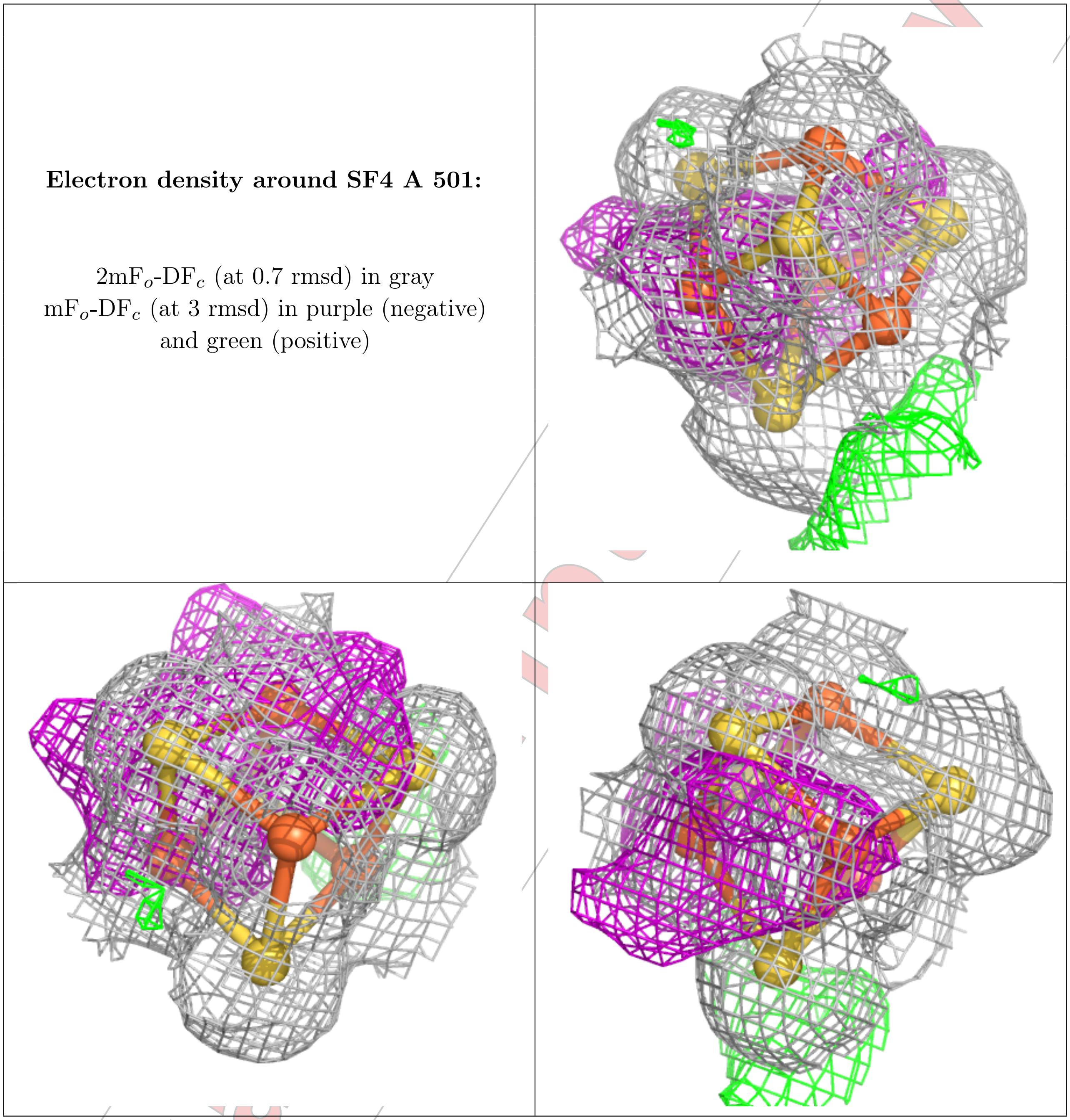

For Manuscript Review

Electron density around SF4 A 503:

2mF$_o$-DF$_c$ (at 0.7 rmsd) in gray
mF$_o$-DF$_c$ (at 3 rmsd) in purple (negative)
and green (positive)

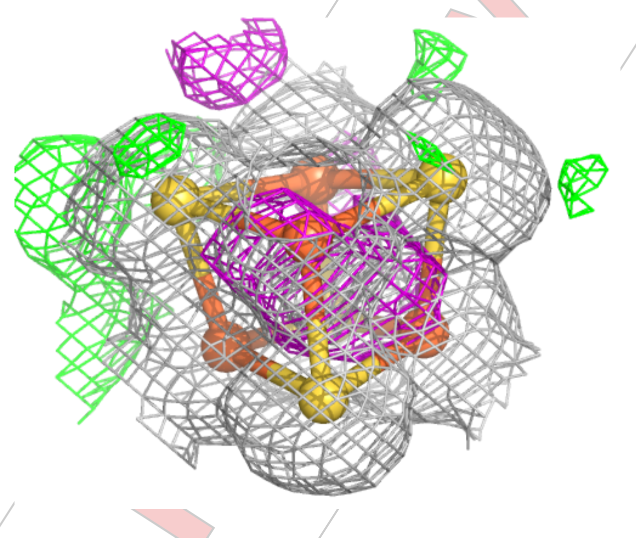

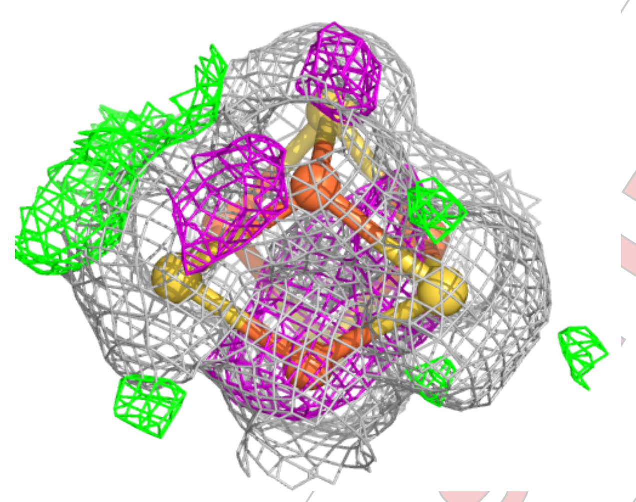

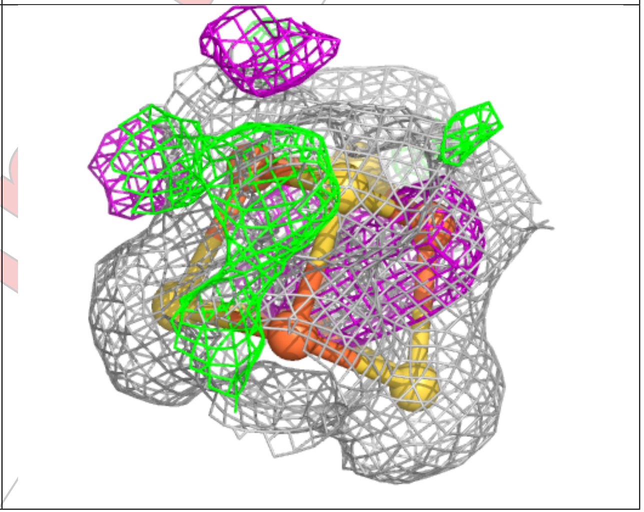

6.5 Other polymers (i)

There are no such residues in this entry.

For Manuscript Review

Full wwPDB X-ray Structure Validation Report (i)

Jan 15, 2025 – 02:26 pm GMT

PDB ID	:	8RTG
Title	:	Desulfovibrio desulfuricans [FeFe] hydrogenase in apo form
Deposited on	:	2024-01-26
Resolution	:	1.46 Å(reported)

This wwPDB validation report is for manuscript review

This is a Full wwPDB X-ray Structure Validation Report.

This report is produced by the wwPDB biocuration pipeline after annotation of the structure.

We welcome your comments at *validation@mail.wwpdb.org*
A user guide is available at
https://www.wwpdb.org/validation/2017/XrayValidationReportHelp
with specific help available everywhere you see the (i) symbol.

The types of validation reports are described at
http://www.wwpdb.org/validation/2017/FAQs#types.

The following versions of software and data (see references (i)) were used in the production of this report:

MolProbity	:	4.02b-467
Mogul	:	1.8.4, CSD as541be (2020)
Xtriage (Phenix)	:	1.13
EDS	:	3.0
buster-report	:	1.1.7 (2018)
Percentile statistics	:	20231227.v01 (using entries in the PDB archive December 27th 2023)
CCP4	:	9.0.003 (Gargrove)
Density-Fitness	:	1.0.11
Ideal geometry (proteins)	:	Engh & Huber (2001)
Ideal geometry (DNA, RNA)	:	Parkinson et al. (1996)

For Manuscript Review

1 Overall quality at a glance ⓘ

The following experimental techniques were used to determine the structure:
X-RAY DIFFRACTION

The reported resolution of this entry is 1.46 Å.

Percentile scores (ranging between 0-100) for global validation metrics of the entry are shown in the following graphic. The table shows the number of entries on which the scores are based.

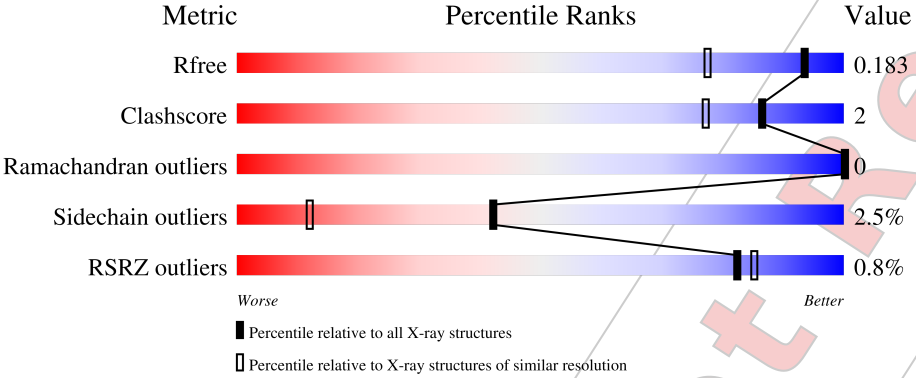

Metric	Whole archive (#Entries)	Similar resolution (#Entries, resolution range(Å))
R_{free}	164625	1556 (1.46-1.46)
Clashscore	180529	1653 (1.46-1.46)
Ramachandran outliers	177936	1635 (1.46-1.46)
Sidechain outliers	177891	1635 (1.46-1.46)
RSRZ outliers	164620	1556 (1.46-1.46)

The table below summarises the geometric issues observed across the polymeric chains and their fit to the electron density. The red, orange, yellow and green segments of the lower bar indicate the fraction of residues that contain outliers for >=3, 2, 1 and 0 types of geometric quality criteria respectively. A grey segment represents the fraction of residues that are not modelled. The numeric value for each fraction is indicated below the corresponding segment, with a dot representing fractions <=5% The upper red bar (where present) indicates the fraction of residues that have poor fit to the electron density. The numeric value is given above the bar.

Mol	Chain	Length	Quality of chain
1	A	405	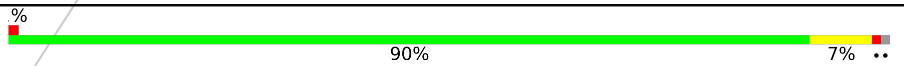
2	B	88	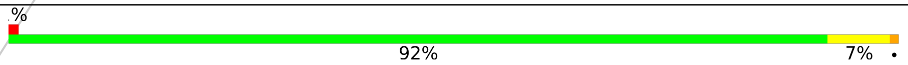

For Manuscript Review

2 Entry composition (i)

There are 6 unique types of molecules in this entry. The entry contains 7917 atoms, of which 3766 are hydrogens and 0 are deuteriums.

In the tables below, the ZeroOcc column contains the number of atoms modelled with zero occupancy, the AltConf column contains the number of residues with at least one atom in alternate conformation and the Trace column contains the number of residues modelled with at most 2 atoms.

- Molecule 1 is a protein called Periplasmic [Fe] hydrogenase large subunit.

Mol	Chain	Residues	Atoms						ZeroOcc	AltConf	Trace
			Total	C	H	N	O	S			
1	A	400	6112	1950	3042	511	574	35	254	4	0

There are 8 discrepancies between the modelled and reference sequences:

Chain	Residue	Modelled	Actual	Comment	Reference
A	398	TRP	-	expression tag	UNP P07598
A	399	SER	-	expression tag	UNP P07598
A	400	HIS	-	expression tag	UNP P07598
A	401	PRO	-	expression tag	UNP P07598
A	402	GLN	-	expression tag	UNP P07598
A	403	PHE	-	expression tag	UNP P07598
A	404	GLU	-	expression tag	UNP P07598
A	405	LYS	-	expression tag	UNP P07598

- Molecule 2 is a protein called Periplasmic [Fe] hydrogenase small subunit.

Mol	Chain	Residues	Atoms						ZeroOcc	AltConf	Trace
			Total	C	H	N	O	S			
2	B	88	1450	466	724	124	135	1	50	3	0

- Molecule 3 is IRON/SULFUR CLUSTER (three-letter code: SF4) (formula: Fe_4S_4) (labeled as "Ligand of Interest" by depositor).

For Manuscript Review

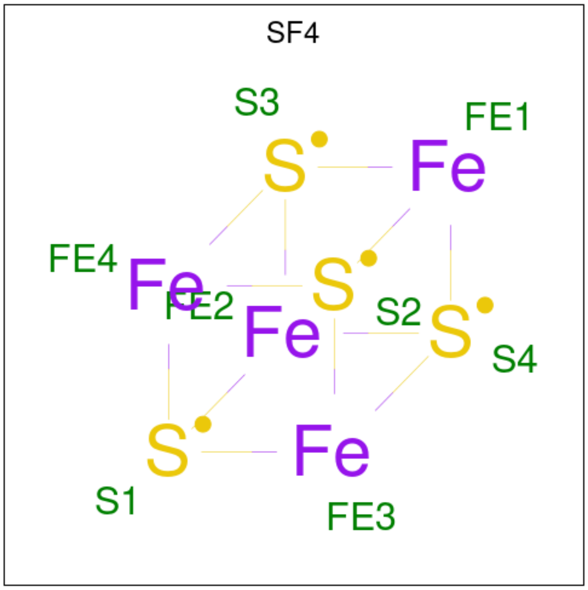

Mol	Chain	Residues	Atoms	ZeroOcc	AltConf
3	A	1	Total Fe S<br>8 4 4	0	0
3	A	1	Total Fe S<br>8 4 4	0	0
3	A	1	Total Fe S<br>8 4 4	0	0

- Molecule 4 is SODIUM ION (three-letter code: NA) (formula: Na).

Mol	Chain	Residues	Atoms	ZeroOcc	AltConf
4	A	1	Total Na<br>1 1	0	0

- Molecule 5 is CHLORIDE ION (three-letter code: CL) (formula: Cl).

Mol	Chain	Residues	Atoms	ZeroOcc	AltConf
5	A	1	Total Cl<br>1 1	0	0

- Molecule 6 is water.

Mol	Chain	Residues	Atoms	ZeroOcc	AltConf
6	A	254	Total O<br>254 254	0	0
6	B	75	Total O<br>75 75	0	0

For Manuscript Review

3 Residue-property plots (i)

These plots are drawn for all protein, RNA, DNA and oligosaccharide chains in the entry. The first graphic for a chain summarises the proportions of the various outlier classes displayed in the second graphic. The second graphic shows the sequence view annotated by issues in geometry and electron density. Residues are color-coded according to the number of geometric quality criteria for which they contain at least one outlier: green = 0, yellow = 1, orange = 2 and red = 3 or more. A red dot above a residue indicates a poor fit to the electron density (RSRZ > 2). Stretches of 2 or more consecutive residues without any outlier are shown as a green connector. Residues present in the sample, but not in the model, are shown in grey.

- Molecule 1: Periplasmic [Fe] hydrogenase large subunit

Chain A:

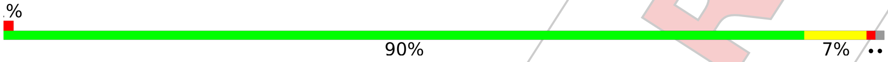

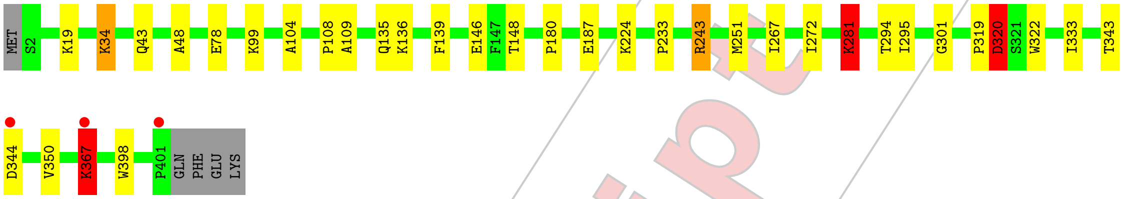

- Molecule 2: Periplasmic [Fe] hydrogenase small subunit

Chain B:

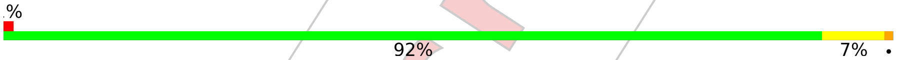

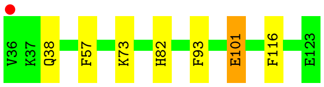

For Manuscript Review

4 Data and refinement statistics (i)

Property	Value	Source
Space group	P 21 21 21	Depositor
Cell constants<br>a, b, c, α, β, γ	49.15Å 84.79Å 88.04Å<br>90.00° 90.00° 90.00°	Depositor
Resolution (Å)	44.06 – 1.46<br>44.06 – 1.46	Depositor<br>EDS
% Data completeness<br>(in resolution range)	99.9 (44.06-1.46)<br>99.9 (44.06-1.46)	Depositor<br>EDS
R_{merge}	0.45	Depositor
R_{sym}	(Not available)	Depositor
$< I/\sigma(I) >$ [1]	0.98 (at 1.46Å)	Xtriage
Refinement program	REFMAC 5.8.0425, REFMAC 5.8.0425	Depositor
R, R_{free}	0.165 , 0.189<br>0.158 , 0.183	Depositor<br>DCC
R_{free} test set	3166 reflections (4.91%)	wwPDB-VP
Wilson B-factor ($Å^2$)	14.4	Xtriage
Anisotropy	0.218	Xtriage
Bulk solvent k_{sol}(e/$Å^3$), B_{sol}($Å^2$)	0.40 , 33.5	EDS
L-test for twinning[2]	$< \|L\| >$ = 0.49, $< L^2 >$ = 0.32	Xtriage
Estimated twinning fraction	0.017 for -h,l,k	Xtriage
F_o,F_c correlation	0.97	EDS
Total number of atoms	7917	wwPDB-VP
Average B, all atoms ($Å^2$)	16.0	wwPDB-VP

Xtriage's analysis on translational NCS is as follows: *The largest off-origin peak in the Patterson function is 7.08% of the height of the origin peak. No significant pseudotranslation is detected.*

[1]Intensities estimated from amplitudes.

[2]Theoretical values of $< |L| >$, $< L^2 >$ for acentric reflections are 0.5, 0.333 respectively for untwinned datasets, and 0.375, 0.2 for perfectly twinned datasets.

For Manuscript Review

5 Model quality (i)

5.1 Standard geometry (i)

Bond lengths and bond angles in the following residue types are not validated in this section: SF4, NA, CL

The Z score for a bond length (or angle) is the number of standard deviations the observed value is removed from the expected value. A bond length (or angle) with $|Z| > 5$ is considered an outlier worth inspection. RMSZ is the root-mean-square of all Z scores of the bond lengths (or angles).

Mol	Chain	Bond lengths		Bond angles	
		RMSZ	$\#\|Z\| >5$	RMSZ	$\#\|Z\| >5$
1	A	1.04	9/3162 (0.3%)	1.10	15/4280 (0.4%)
2	B	3.56	5/763 (0.7%)	1.72	6/1030 (0.6%)
All	All	1.83	14/3925 (0.4%)	1.24	21/5310 (0.4%)

Chiral center outliers are detected by calculating the chiral volume of a chiral center and verifying if the center is modelled as a planar moiety or with the opposite hand.A planarity outlier is detected by checking planarity of atoms in a peptide group, atoms in a mainchain group or atoms of a sidechain that are expected to be planar.

Mol	Chain	#Chirality outliers	#Planarity outliers
1	A	0	3
2	B	0	2
All	All	0	5

All (14) bond length outliers are listed below:

Mol	Chain	Res	Type	Atoms	Z	Observed(Å)	Ideal(Å)
2	B	101[A]	GLU	CD-OE1	66.49	1.98	1.25
2	B	101[B]	GLU	CD-OE1	66.49	1.98	1.25
1	A	19	LYS	CD-CE	36.22	2.41	1.51
1	A	320	ASP	CB-CG	18.46	1.90	1.51
1	A	367	LYS	C-O	-18.27	0.88	1.23
2	B	101[A]	GLU	CD-OE2	-17.68	1.06	1.25
2	B	101[B]	GLU	CD-OE2	-17.68	1.06	1.25
1	A	343	THR	CB-OG1	-12.38	1.18	1.43
1	A	367	LYS	CA-C	10.89	1.81	1.52
1	A	136	LYS	CE-NZ	9.51	1.72	1.49
1	A	135	GLN	CD-OE1	-9.46	1.03	1.24
1	A	367	LYS	CA-CB	6.61	1.68	1.53
1	A	367	LYS	N-CA	6.07	1.58	1.46
2	B	73	LYS	CD-CE	-5.36	1.37	1.51

For Manuscript Review

All (21) bond angle outliers are listed below:

Mol	Chain	Res	Type	Atoms	Z	Observed(^{o})	Ideal(^{o})
2	B	101[A]	GLU	OE1-CD-OE2	-30.23	87.03	123.30
2	B	101[B]	GLU	OE1-CD-OE2	-30.23	87.03	123.30
1	A	320	ASP	CB-CG-OD1	-29.54	91.71	118.30
1	A	320	ASP	CB-CG-OD2	-20.71	99.66	118.30
1	A	367	LYS	CA-CB-CG	13.92	144.03	113.40
1	A	19	LYS	CG-CD-CE	-12.76	73.64	111.90
2	B	101[A]	GLU	CG-CD-OE2	11.29	140.88	118.30
2	B	101[B]	GLU	CG-CD-OE2	11.29	140.88	118.30
1	A	367	LYS	CA-C-N	-9.68	95.89	117.20
2	B	101[A]	GLU	CG-CD-OE1	-9.11	100.08	118.30
2	B	101[B]	GLU	CG-CD-OE1	-9.11	100.08	118.30
1	A	344	ASP	CB-CG-OD2	7.92	125.43	118.30
1	A	367	LYS	N-CA-CB	-6.13	99.57	110.60
1	A	343	THR	CA-CB-OG1	5.76	121.10	109.00
1	A	367	LYS	CB-CA-C	5.72	121.84	110.40
1	A	281	LYS	CA-CB-CG	5.61	125.73	113.40
1	A	367	LYS	N-CA-C	5.50	125.84	111.00
1	A	243	ARG	NE-CZ-NH2	-5.29	117.66	120.30
1	A	344	ASP	OD1-CG-OD2	-5.21	113.41	123.30
1	A	135	GLN	CG-CD-NE2	-5.09	104.47	116.70
1	A	251	MET	CG-SD-CE	-5.06	92.10	100.20

There are no chirality outliers.

All (5) planarity outliers are listed below:

Mol	Chain	Res	Type	Group
1	A	243	ARG	Sidechain
1	A	320	ASP	Sidechain
1	A	367	LYS	Mainchain
2	B	101[A]	GLU	Sidechain
2	B	101[B]	GLU	Sidechain

5.2 Too-close contacts (i)

In the following table, the Non-H and H(model) columns list the number of non-hydrogen atoms and hydrogen atoms in the chain respectively. The H(added) column lists the number of hydrogen atoms added and optimized by MolProbity. The Clashes column lists the number of clashes within the asymmetric unit, whereas Symm-Clashes lists symmetry-related clashes.

For Manuscript Review

Mol	Chain	Non-H	H(model)	H(added)	Clashes	Symm-Clashes
1	A	3070	3042	3011	15	4
2	B	726	724	707	3	0
3	A	24	0	0	1	0
4	A	1	0	0	0	0
5	A	1	0	0	0	0
6	A	254	0	0	1	0
6	B	75	0	0	0	0
All	All	4151	3766	3718	15	4

The all-atom clashscore is defined as the number of clashes found per 1000 atoms (including hydrogen atoms). The all-atom clashscore for this structure is 2.

All (15) close contacts within the same asymmetric unit are listed below, sorted by their clash magnitude.

Atom-1	Atom-2	Interatomic distance (Å)	Clash overlap (Å)
1:A:43:GLN:NE2	6:A:601:HOH:O	2.01	0.88
1:A:48:ALA:HA	2:B:116:PHE:CE2	2.45	0.52
1:A:295:ILE:O	1:A:301:GLY:HA3	2.10	0.51
1:A:108:PRO:HG3	1:A:146:GLU:HG3	1.96	0.47
1:A:267:ILE:HG23	1:A:272:ILE:HB	1.98	0.46
1:A:320:ASP:CG	1:A:320:ASP:CA	2.84	0.45
1:A:333:ILE:HG12	1:A:350[B]:VAL:HG13	1.99	0.45
1:A:109:ALA:HB3	1:A:233:PRO:HG3	1.98	0.45
1:A:34:LYS:HE2	1:A:398:TRP:CD1	2.52	0.44
1:A:48:ALA:HA	2:B:116:PHE:CD2	2.54	0.43
1:A:187:GLU:HB3	2:B:93:PHE:O	2.19	0.42
1:A:319:PRO:HD2	1:A:322:TRP:CZ3	2.54	0.42
1:A:34:LYS:HE2	1:A:78:GLU:O	2.21	0.40
1:A:104:ALA:HB2	1:A:139:PHE:CG	2.56	0.40
1:A:180:PRO:HD2	3:A:501:SF4:S1	2.61	0.40

All (4) symmetry-related close contacts are listed below. The label for Atom-2 includes the symmetry operator and encoded unit-cell translations to be applied.

Atom-1	Atom-2	Interatomic distance (Å)	Clash overlap (Å)
1:A:281:LYS:HE3	1:A:320:ASP:O[4_554]	1.03	0.57
1:A:281:LYS:CE	1:A:320:ASP:O[4_554]	1.68	0.52
1:A:281:LYS:NZ	1:A:320:ASP:O[4_554]	2.11	0.09
1:A:281:LYS:HE3	1:A:320:ASP:C[4_554]	1.55	0.05

For Manuscript Review

5.3 Torsion angles (i)

5.3.1 Protein backbone (i)

In the following table, the Percentiles column shows the percent Ramachandran outliers of the chain as a percentile score with respect to all X-ray entries followed by that with respect to entries of similar resolution.

The Analysed column shows the number of residues for which the backbone conformation was analysed, and the total number of residues.

Mol	Chain	Analysed	Favoured	Allowed	Outliers	Percentiles	
1	A	402/405 (99%)	393 (98%)	9 (2%)	0	100	100
2	B	89/88 (101%)	87 (98%)	2 (2%)	0	100	100
All	All	491/493 (100%)	480 (98%)	11 (2%)	0	100	100

There are no Ramachandran outliers to report.

5.3.2 Protein sidechains (i)

In the following table, the Percentiles column shows the percent sidechain outliers of the chain as a percentile score with respect to all X-ray entries followed by that with respect to entries of similar resolution.

The Analysed column shows the number of residues for which the sidechain conformation was analysed, and the total number of residues.

Mol	Chain	Analysed	Rotameric	Outliers	Percentiles	
1	A	329/330 (100%)	322 (98%)	7 (2%)	48	17
2	B	79/76 (104%)	76 (96%)	3 (4%)	28	4
All	All	408/406 (100%)	398 (98%)	10 (2%)	42	12

All (10) residues with a non-rotameric sidechain are listed below:

Mol	Chain	Res	Type
1	A	34	LYS
1	A	99	LYS
1	A	148	THR
1	A	224	LYS
1	A	281	LYS
1	A	294	THR
1	A	367	LYS
2	B	38	GLN

Continued on next page...

For Manuscript Review

Continued from previous page...

Mol	Chain	Res	Type
2	B	57	PHE
2	B	82	HIS

Sometimes sidechains can be flipped to improve hydrogen bonding and reduce clashes. All (3) such sidechains are listed below:

Mol	Chain	Res	Type
1	A	43	GLN
1	A	174	GLN
1	A	225	GLN

5.3.3 RNA ⓘ

There are no RNA molecules in this entry.

5.4 Non-standard residues in protein, DNA, RNA chains ⓘ

There are no non-standard protein/DNA/RNA residues in this entry.

5.5 Carbohydrates ⓘ

There are no oligosaccharides in this entry.

5.6 Ligand geometry ⓘ

Of 5 ligands modelled in this entry, 2 are monoatomic - leaving 3 for Mogul analysis.

In the following table, the Counts columns list the number of bonds (or angles) for which Mogul statistics could be retrieved, the number of bonds (or angles) that are observed in the model and the number of bonds (or angles) that are defined in the Chemical Component Dictionary. The Link column lists molecule types, if any, to which the group is linked. The Z score for a bond length (or angle) is the number of standard deviations the observed value is removed from the expected value. A bond length (or angle) with $|Z| > 2$ is considered an outlier worth inspection. RMSZ is the root-mean-square of all Z scores of the bond lengths (or angles).

Mol	Type	Chain	Res	Link	Bond lengths			Bond angles		
					Counts	RMSZ	$\#\|Z\| > 2$	Counts	RMSZ	$\#\|Z\| > 2$
3	SF4	A	503	1	0,12,12	-	-	-		
3	SF4	A	501	1	0,12,12	-	-	-		
3	SF4	A	502	1	0,12,12	-	-	-		

For Manuscript Review

In the following table, the Chirals column lists the number of chiral outliers, the number of chiral centers analysed, the number of these observed in the model and the number defined in the Chemical Component Dictionary. Similar counts are reported in the Torsion and Rings columns. '-' means no outliers of that kind were identified.

Mol	Type	Chain	Res	Link	Chirals	Torsions	Rings
3	SF4	A	503	1	-	-	0/6/5/5
3	SF4	A	501	1	-	-	0/6/5/5
3	SF4	A	502	1	-	-	0/6/5/5

There are no bond length outliers.

There are no bond angle outliers.

There are no chirality outliers.

There are no torsion outliers.

There are no ring outliers.

1 monomer is involved in 1 short contact:

Mol	Chain	Res	Type	Clashes	Symm-Clashes
3	A	501	SF4	1	0

The following is a two-dimensional graphical depiction of Mogul quality analysis of bond lengths, bond angles, torsion angles, and ring geometry for all instances of the Ligand of Interest. In addition, ligands with molecular weight > 250 and outliers as shown on the validation Tables will also be included. For torsion angles, if less then 5% of the Mogul distribution of torsion angles is within 10 degrees of the torsion angle in question, then that torsion angle is considered an outlier. Any bond that is central to one or more torsion angles identified as an outlier by Mogul will be highlighted in the graph. For rings, the root-mean-square deviation (RMSD) between the ring in question and similar rings identified by Mogul is calculated over all ring torsion angles. If the average RMSD is greater than 60 degrees and the minimal RMSD between the ring in question and any Mogul-identified rings is also greater than 60 degrees, then that ring is considered an outlier. The outliers are highlighted in purple. The color gray indicates Mogul did not find sufficient equivalents in the CSD to analyse the geometry.

For Manuscript Review

Ligand SF4 A 503

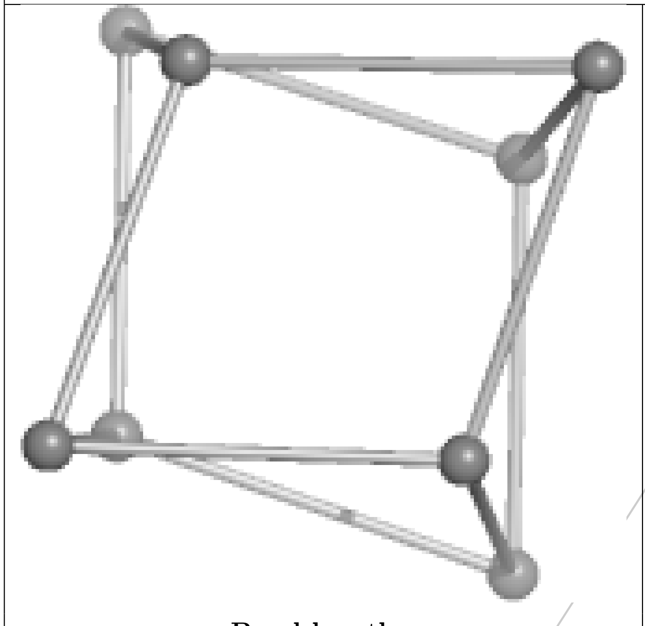

Bond lengths

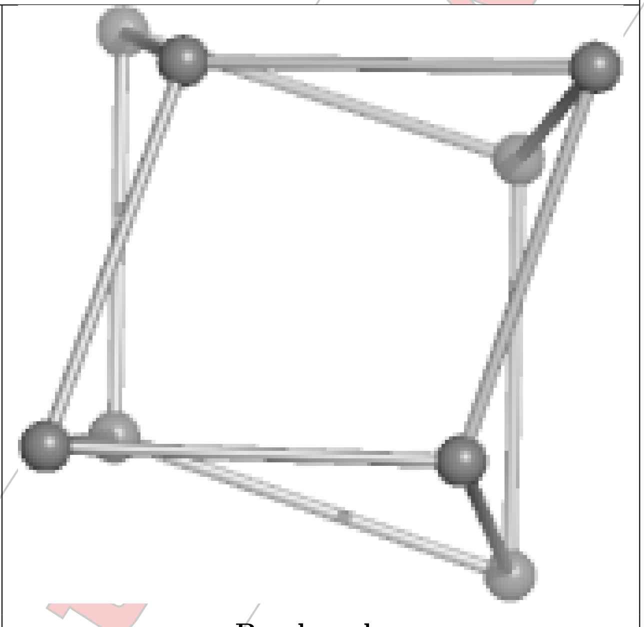

Bond angles

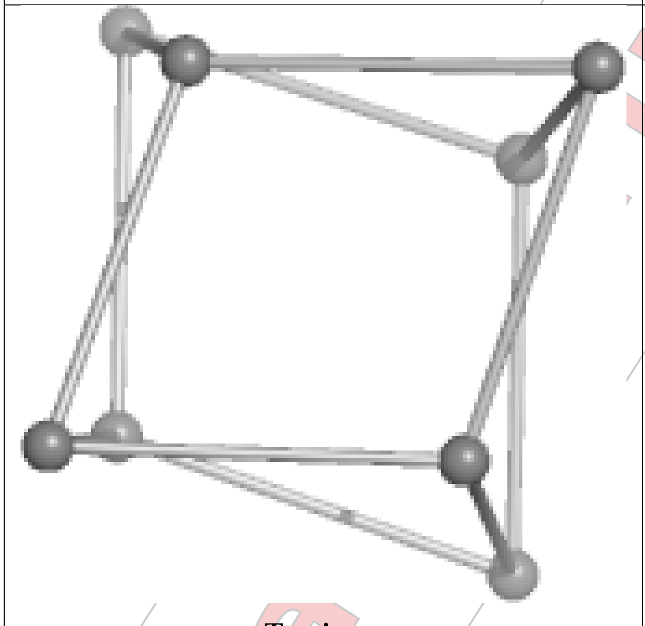

Torsions

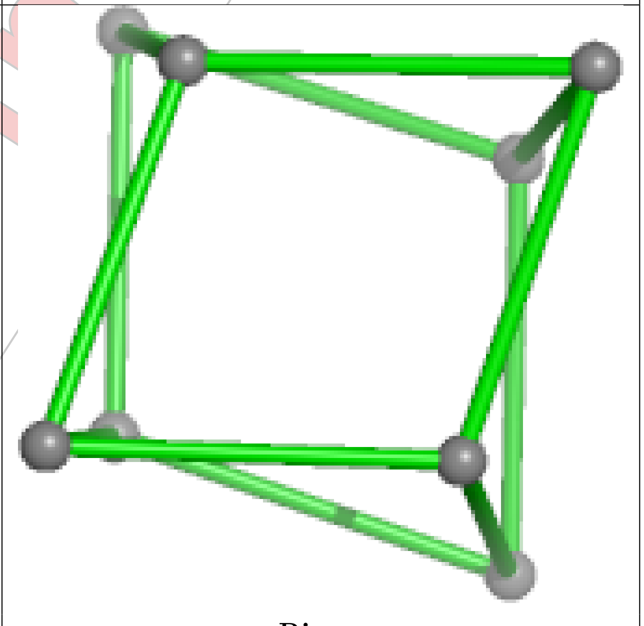

Rings

For Manuscript Review

Ligand SF4 A 501

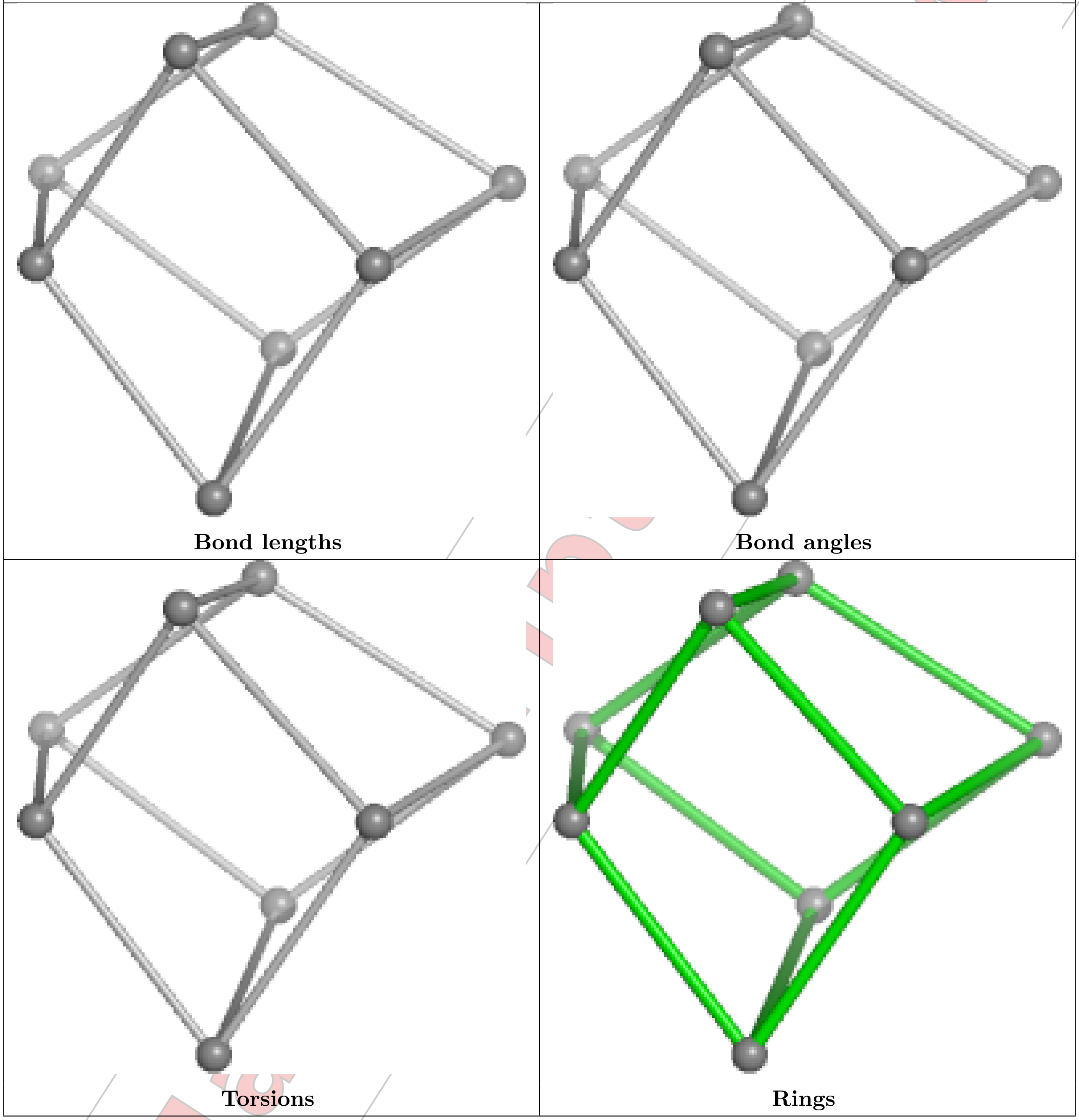

For Manuscript Review

Ligand SF4 A 502

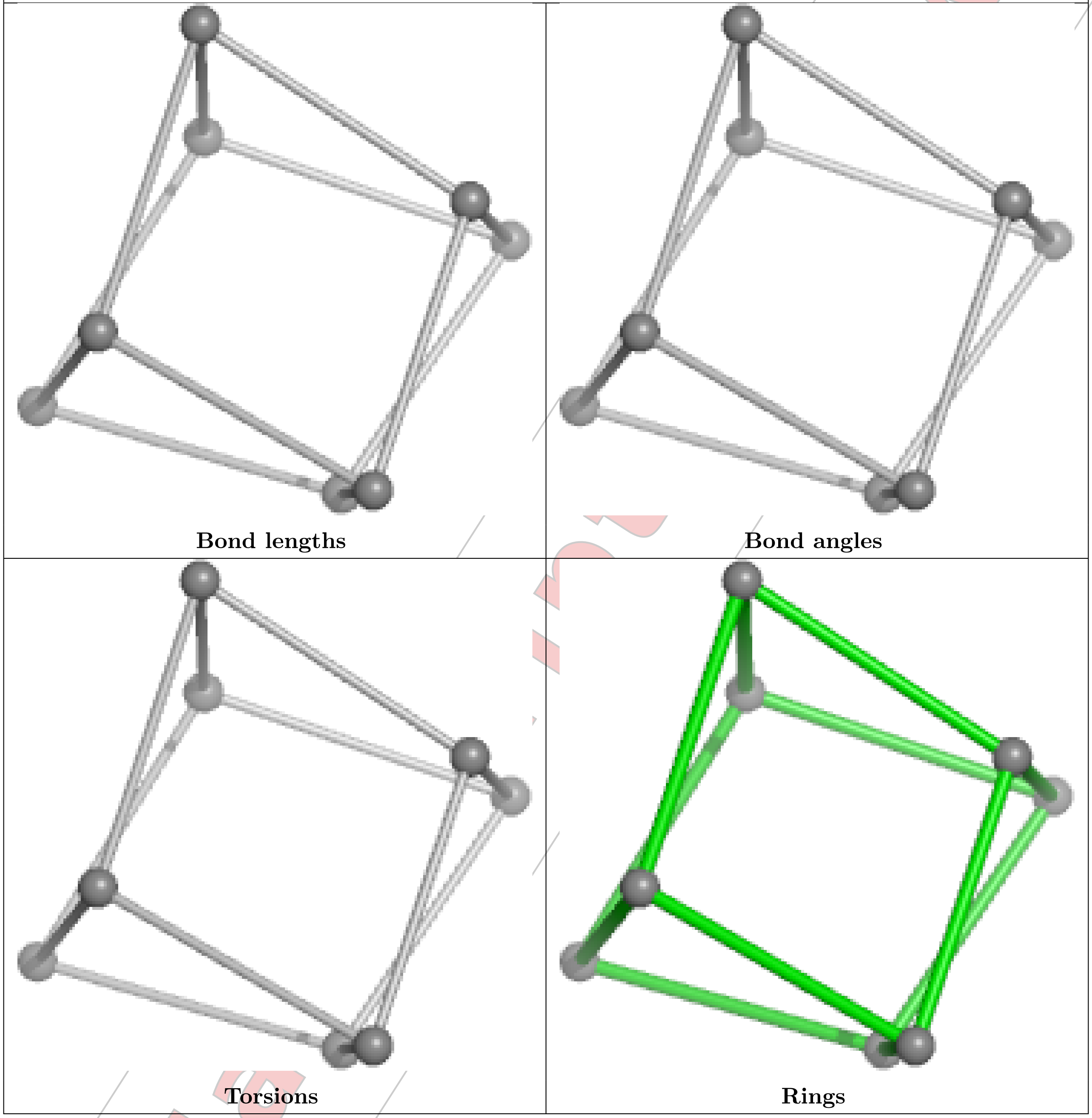

5.7 Other polymers (i)

There are no such residues in this entry.

5.8 Polymer linkage issues (i)

There are no chain breaks in this entry.

For Manuscript Review

6 Fit of model and data (i)

6.1 Protein, DNA and RNA chains (i)

In the following table, the column labelled '#RSRZ> 2' contains the number (and percentage) of RSRZ outliers, followed by percent RSRZ outliers for the chain as percentile scores relative to all X-ray entries and entries of similar resolution. The OWAB column contains the minimum, median, 95th percentile and maximum values of the occupancy-weighted average B-factor per residue. The column labelled 'Q< 0.9' lists the number of (and percentage) of residues with an average occupancy less than 0.9.

Mol	Chain	Analysed	<RSRZ>	#RSRZ>2			OWAB(Å^2)	Q<0.9
1	A	400/405 (98%)	-0.47	3 (0%)	82	85	5, 13, 24, 63	28 (7%)
2	B	88/88 (100%)	-0.42	1 (1%)	77	81	6, 14, 24, 31	6 (6%)
All	All	488/493 (98%)	-0.46	4 (0%)	82	85	5, 13, 24, 63	34 (6%)

All (4) RSRZ outliers are listed below:

Mol	Chain	Res	Type	RSRZ
1	A	367	LYS	4.2
2	B	36	VAL	2.9
1	A	401	PRO	2.1
1	A	344	ASP	2.1

6.2 Non-standard residues in protein, DNA, RNA chains (i)

There are no non-standard protein/DNA/RNA residues in this entry.

6.3 Carbohydrates (i)

There are no monosaccharides in this entry.

6.4 Ligands (i)

In the following table, the Atoms column lists the number of modelled atoms in the group and the number defined in the chemical component dictionary. The B-factors column lists the minimum, median, 95th percentile and maximum values of B factors of atoms in the group. The column labelled 'Q< 0.9' lists the number of atoms with occupancy less than 0.9.

Mol	Type	Chain	Res	Atoms	RSCC	RSR	B-factors(Å^2)	Q<0.9
4	NA	A	504	1/1	0.99	0.03	14,14,14,14	0

Continued on next page...

For Manuscript Review

Continued from previous page...

Mol	Type	Chain	Res	Atoms	RSCC	RSR	B-factors($Å^2$)	Q<0.9
5	CL	A	505	1/1	0.99	0.03	18,18,18,18	0
3	SF4	A	503	8/8	1.00	0.01	11,12,13,13	0
3	SF4	A	501	8/8	1.00	0.01	10,11,11,11	0
3	SF4	A	502	8/8	1.00	0.02	10,10,11,11	0

The following is a graphical depiction of the model fit to experimental electron density of all instances of the Ligand of Interest. In addition, ligands with molecular weight > 250 and outliers as shown on the geometry validation Tables will also be included. Each fit is shown from different orientation to approximate a three-dimensional view.

Electron density around SF4 A 503:

$2mF_o$-DF_c (at 0.7 rmsd) in gray
mF_o-DF_c (at 3 rmsd) in purple (negative)
and green (positive)

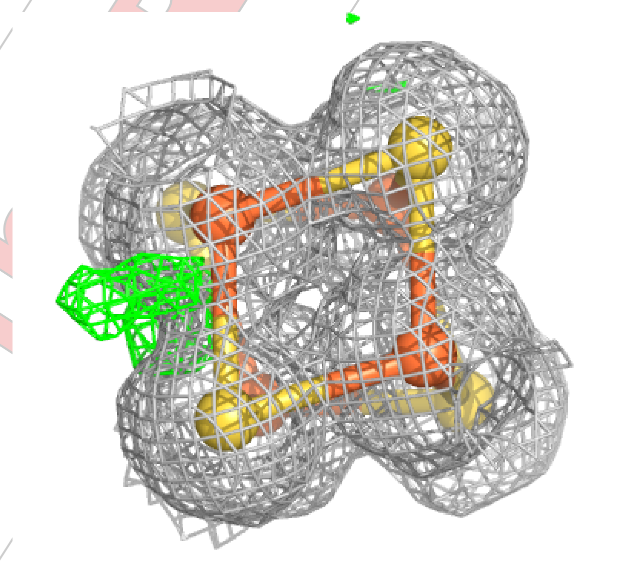

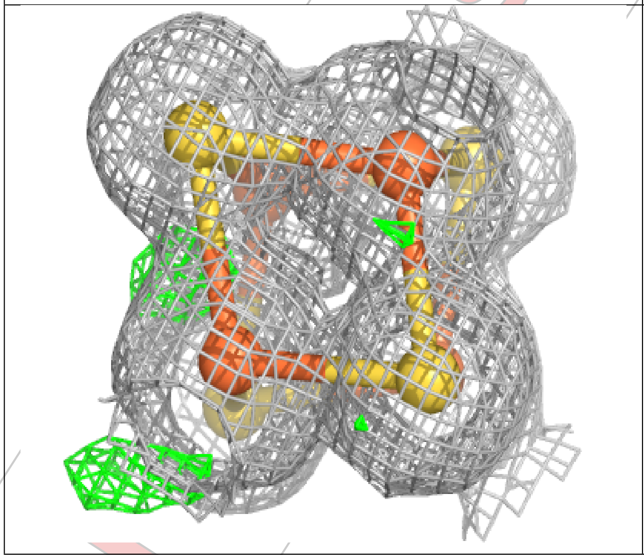

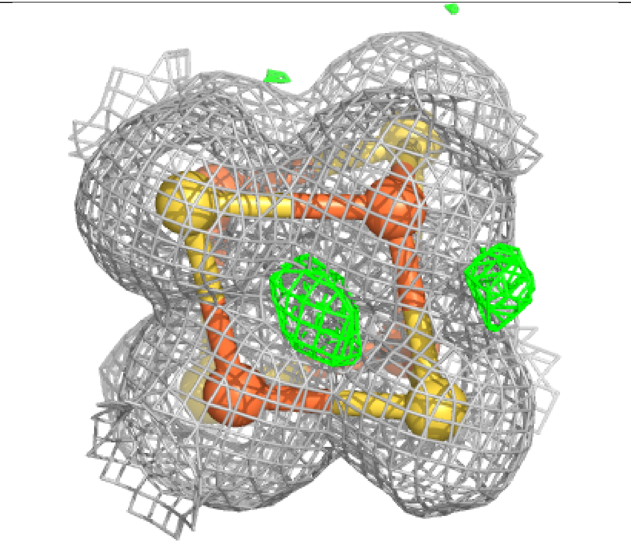

For Manuscript Review

Electron density around SF4 A 501:

2mF$_o$-DF$_c$ (at 0.7 rmsd) in gray
mF$_o$-DF$_c$ (at 3 rmsd) in purple (negative)
and green (positive)

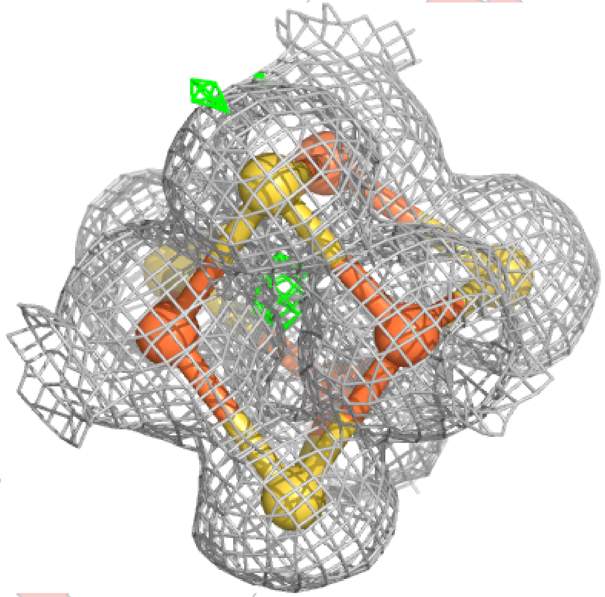

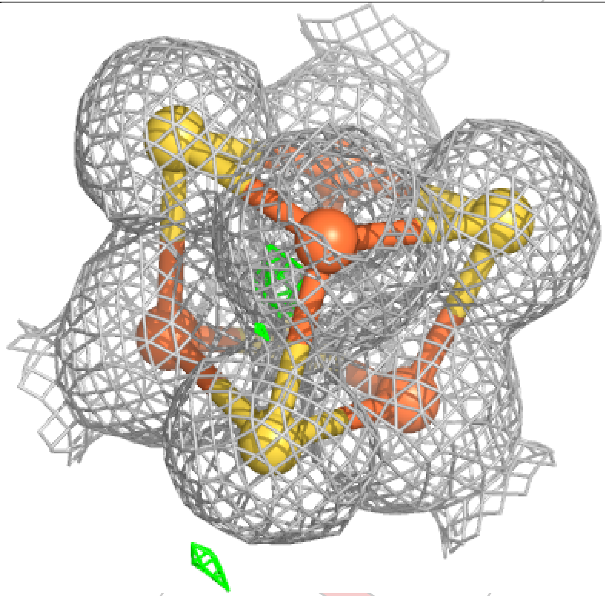

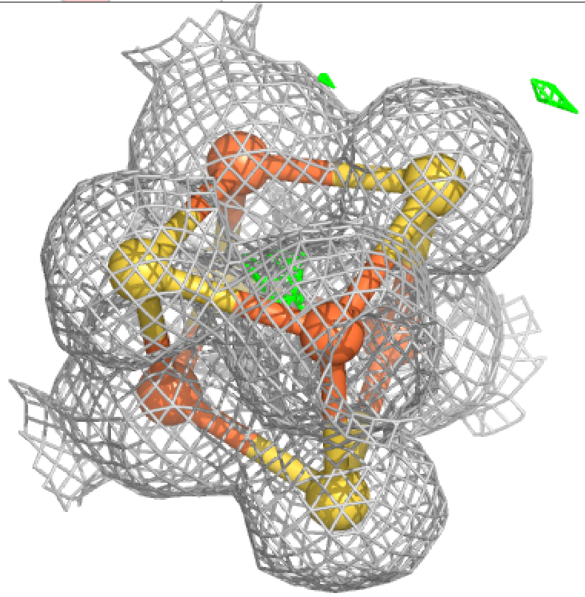

For Manuscript Review

Electron density around SF4 A 502:

$2mF_o$-DF_c (at 0.7 rmsd) in gray
mF_o-DF_c (at 3 rmsd) in purple (negative)
and green (positive)

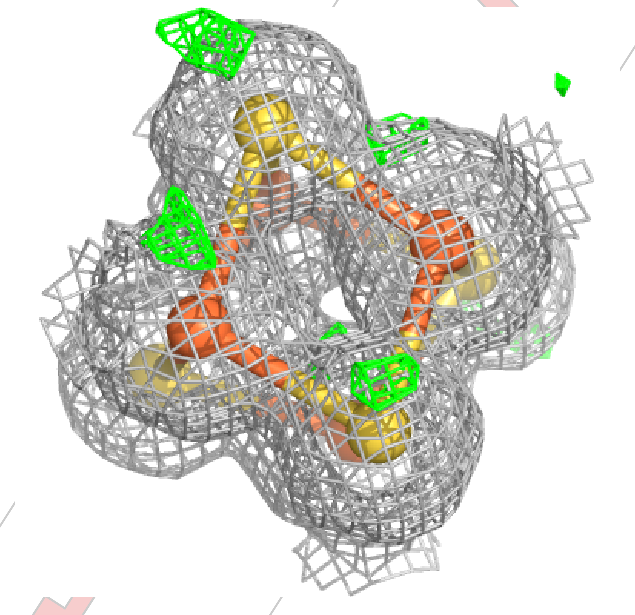

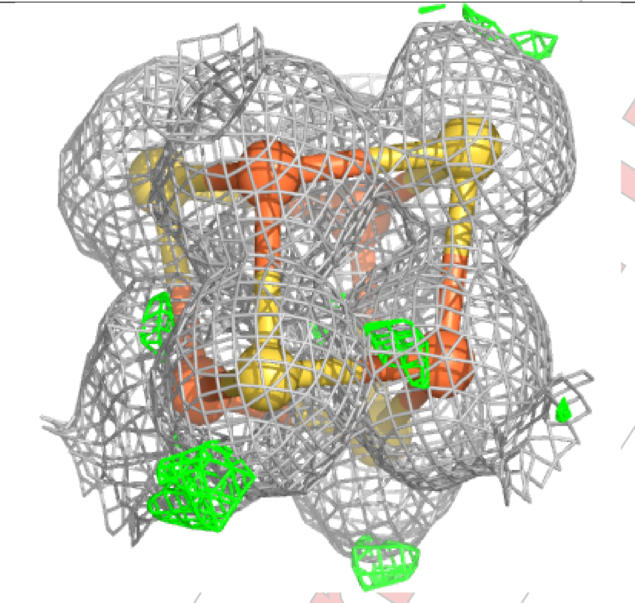

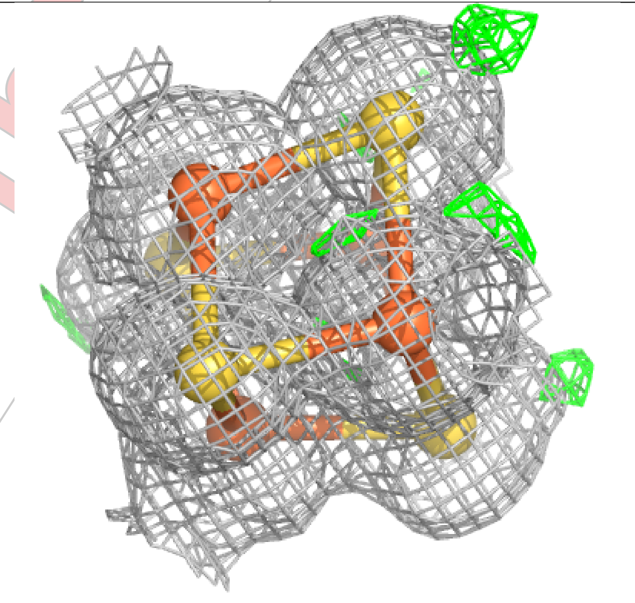

6.5 Other polymers ⓘ

There are no such residues in this entry.

Full wwPDB X-ray Structure Validation Report (i)

Mar 10, 2025 – 03:57 pm GMT

PDB ID	:	9QD6
Title	:	High resolution structure of the artificially maturated [FeFe]-hydrogenase from Nitratidesulfovibrio vulgaris str. Hildenborough
Deposited on	:	2025-03-06
Resolution	:	1.18 Å(reported)

This wwPDB validation report is for manuscript review

This is a Full wwPDB X-ray Structure Validation Report.

This report is produced by the wwPDB biocuration pipeline after annotation of the structure.

We welcome your comments at *validation@mail.wwpdb.org*
A user guide is available at
https://www.wwpdb.org/validation/2017/XrayValidationReportHelp
with specific help available everywhere you see the (i) symbol.

The types of validation reports are described at
http://www.wwpdb.org/validation/2017/FAQs#types.

For Manuscript Review

The following versions of software and data (see references (i)) were used in the production of this report:

MolProbity	:	4.02b-467
Mogul	:	1.8.4, CSD as541be (2020)
Xtriage (Phenix)	:	1.13
EDS	:	3.0
buster-report	:	1.1.7 (2018)
Percentile statistics	:	20231227.v01 (using entries in the PDB archive December 27th 2023)
CCP4	:	9.0.003 (Gargrove)
Density-Fitness	:	1.0.11
Ideal geometry (proteins)	:	Engh & Huber (2001)

1 Overall quality at a glance (i)

The following experimental techniques were used to determine the structure:
X-RAY DIFFRACTION

The reported resolution of this entry is 1.18 Å.

Percentile scores (ranging between 0-100) for global validation metrics of the entry are shown in the following graphic. The table shows the number of entries on which the scores are based.

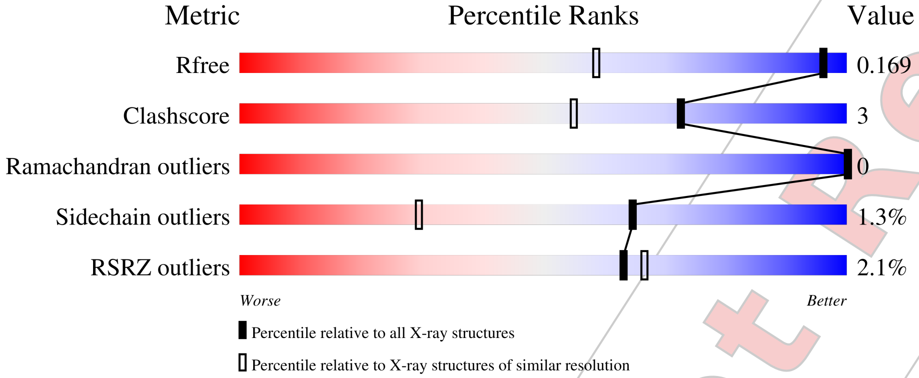

Metric	Whole archive (#Entries)	Similar resolution (#Entries, resolution range(Å))
R_{free}	164625	1569 (1.20-1.16)
Clashscore	180529	1711 (1.20-1.16)
Ramachandran outliers	177936	1657 (1.20-1.16)
Sidechain outliers	177891	1657 (1.20-1.16)
RSRZ outliers	164620	1568 (1.20-1.16)

The table below summarises the geometric issues observed across the polymeric chains and their fit to the electron density. The red, orange, yellow and green segments of the lower bar indicate the fraction of residues that contain outliers for >=3, 2, 1 and 0 types of geometric quality criteria respectively. A grey segment represents the fraction of residues that are not modelled. The numeric value for each fraction is indicated below the corresponding segment, with a dot representing fractions <=5% The upper red bar (where present) indicates the fraction of residues that have poor fit to the electron density. The numeric value is given above the bar.

Mol	Chain	Length	Quality of chain
1	B	88	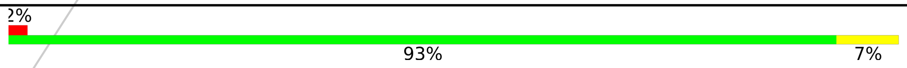
2	A	405	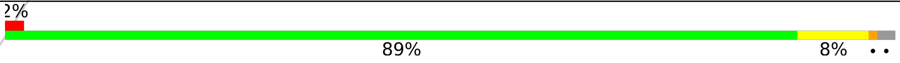

The following table lists non-polymeric compounds, carbohydrate monomers and non-standard

Ideal geometry (DNA, RNA) : Parkinson et al. (1996)
Validation Pipeline (wwPDB-VP) : 2.41

For Manuscript Review

residues in protein, DNA, RNA chains that are outliers for geometric or electron-density-fit criteria:

Mol	Type	Chain	Res	Chirality	Geometry	Clashes	Electron density
5	TAM	A	505	-	-	X	-

For Manuscript Review

2 Entry composition (i)

There are 7 unique types of molecules in this entry. The entry contains 8072 atoms, of which 3839 are hydrogens and 0 are deuteriums.

In the tables below, the ZeroOcc column contains the number of atoms modelled with zero occupancy, the AltConf column contains the number of residues with at least one atom in alternate conformation and the Trace column contains the number of residues modelled with at most 2 atoms.

- Molecule 1 is a protein called Periplasmic [Fe] hydrogenase small subunit.

Mol	Chain	Residues	Atoms						ZeroOcc	AltConf	Trace
			Total	C	H	N	O	S			
1	B	88	1461	469	734	124	133	1	36	4	0

- Molecule 2 is a protein called Periplasmic [Fe] hydrogenase large subunit.

Mol	Chain	Residues	Atoms						ZeroOcc	AltConf	Trace
			Total	C	H	N	O	S			
2	A	396	6129	1948	3063	508	571	39	220	11	0

There are 8 discrepancies between the modelled and reference sequences:

Chain	Residue	Modelled	Actual	Comment	Reference
A	398	TRP	-	expression tag	UNP P07598
A	399	SER	-	expression tag	UNP P07598
A	400	HIS	-	expression tag	UNP P07598
A	401	PRO	-	expression tag	UNP P07598
A	402	GLN	-	expression tag	UNP P07598
A	403	PHE	-	expression tag	UNP P07598
A	404	GLU	-	expression tag	UNP P07598
A	405	LYS	-	expression tag	UNP P07598

- Molecule 3 is dicarbonyl[bis(cyanide-kappaC)]-mu-(iminodimethanethiolatato-1kappaS:2kappaS)-mu-(oxomethylidene)diiron(2+) (three-letter code: 402) (formula: $C_7H_5Fe_2N_3O_3S_2$) (labeled as "Ligand of Interest" by depositor).

For Manuscript Review

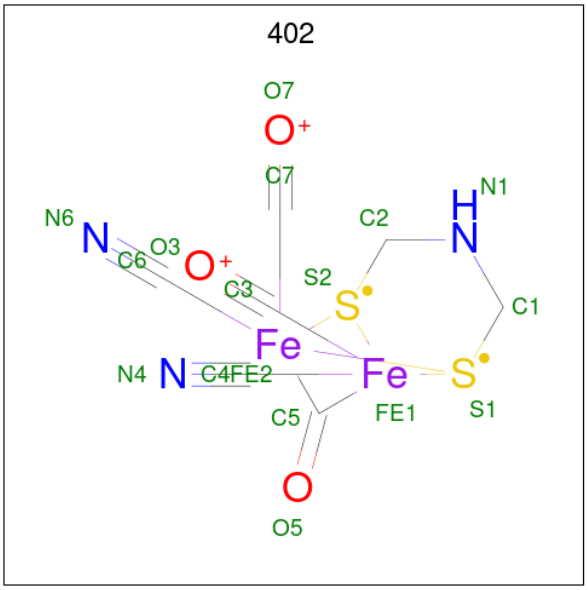

Mol	Chain	Residues	Atoms							ZeroOcc	AltConf
			Total	C	Fe	H	N	O	S		
3	A	1	24	7	2	7	3	3	2	1	0

- Molecule 4 is IRON/SULFUR CLUSTER (three-letter code: SF4) (formula: Fe_4S_4) (labeled as "Ligand of Interest" by depositor).

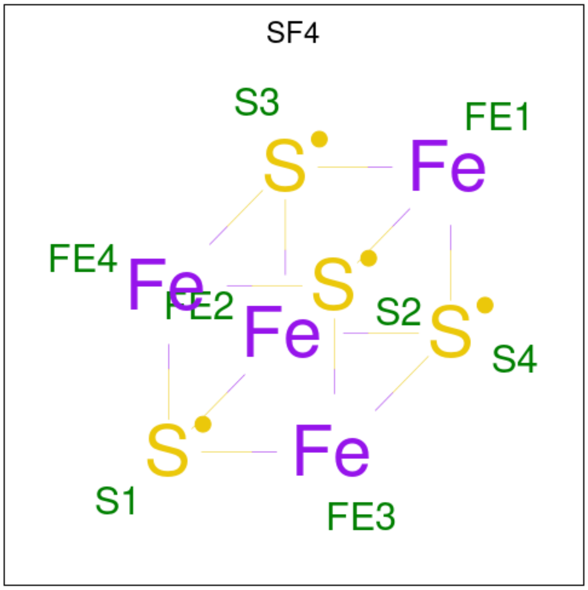

Mol	Chain	Residues	Atoms			ZeroOcc	AltConf
			Total	Fe	S		
4	A	1	8	4	4	0	0
4	A	1	8	4	4	0	0

Continued on next page...

For Manuscript Review

Continued from previous page...

Mol	Chain	Residues	Atoms			ZeroOcc	AltConf
			Total	Fe	S		
4	A	1	8	4	4	0	0

- Molecule 5 is TRIS(HYDROXYETHYL)AMINOMETHANE (three-letter code: TAM) (formula: $C_7H_{17}NO_3$).

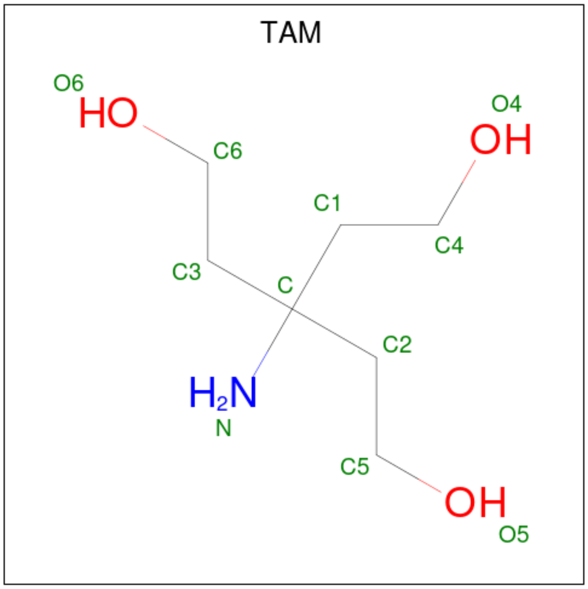

Mol	Chain	Residues	Atoms					ZeroOcc	AltConf
			Total	C	H	N	O		
5	A	1	28	7	17	1	3	5	0

- Molecule 6 is TETRAETHYLENE GLYCOL (three-letter code: PG4) (formula: $C_8H_{18}O_5$).

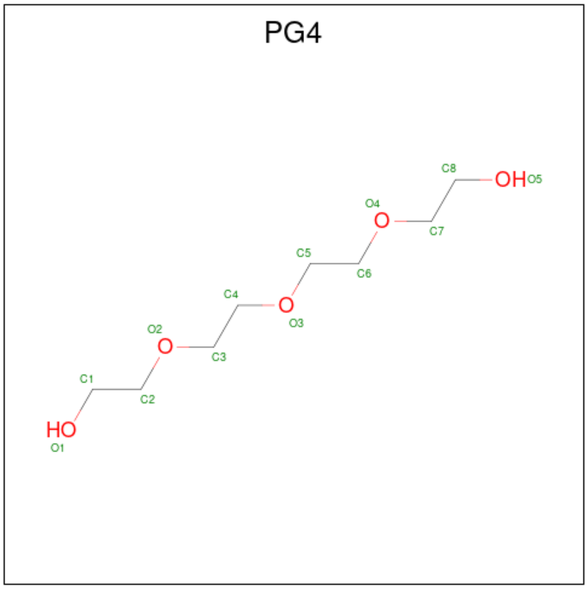

Mol	Chain	Residues	Atoms				ZeroOcc	AltConf
			Total	C	H	O		
6	A	1	31	8	18	5	2	0

- Molecule 7 is water.

Mol	Chain	Residues	Atoms		ZeroOcc	AltConf
			Total	O		
7	B	76	76	76	0	0
7	A	299	299	299	0	0

For Manuscript Review

3 Residue-property plots (i)

These plots are drawn for all protein, RNA, DNA and oligosaccharide chains in the entry. The first graphic for a chain summarises the proportions of the various outlier classes displayed in the second graphic. The second graphic shows the sequence view annotated by issues in geometry and electron density. Residues are color-coded according to the number of geometric quality criteria for which they contain at least one outlier: green = 0, yellow = 1, orange = 2 and red = 3 or more. A red dot above a residue indicates a poor fit to the electron density (RSRZ > 2). Stretches of 2 or more consecutive residues without any outlier are shown as a green connector. Residues present in the sample, but not in the model, are shown in grey.

- Molecule 1: Periplasmic [Fe] hydrogenase small subunit

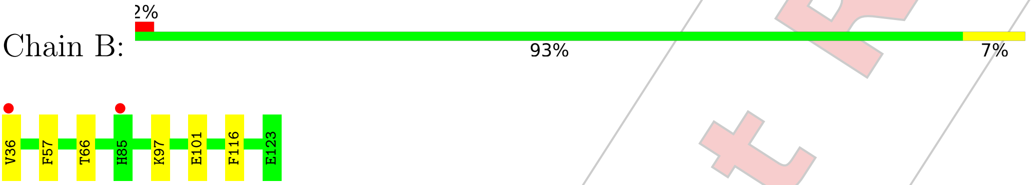

- Molecule 2: Periplasmic [Fe] hydrogenase large subunit

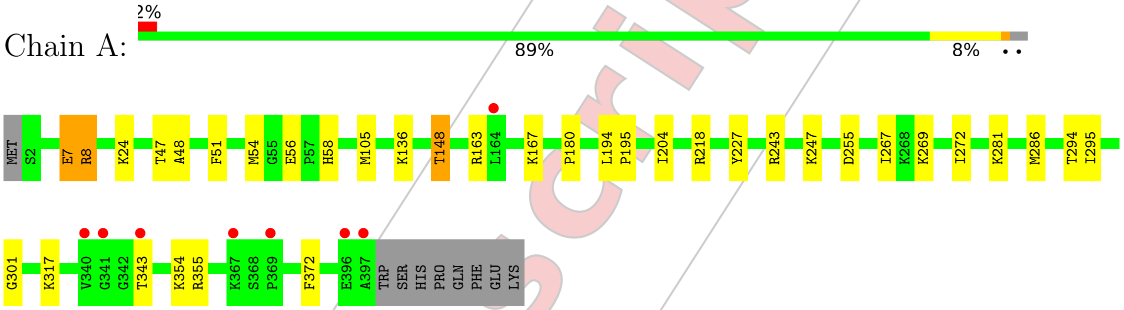

For Manuscript Review

4 Data and refinement statistics (i)

Property	Value	Source
Space group	P 21 21 21	Depositor
Cell constants<br>a, b, c, α, β, γ	49.44Å 87.27Å 89.17Å<br>90.00° 90.00° 90.00°	Depositor
Resolution (Å)	44.62 – 1.18<br>44.62 – 1.18	Depositor<br>EDS
% Data completeness<br>(in resolution range)	99.9 (44.62-1.18)<br>99.8 (44.62-1.18)	Depositor<br>EDS
R_{merge}	0.18	Depositor
R_{sym}	(Not available)	Depositor
$< I/\sigma(I) >$ [1]	1.67 (at 1.18Å)	Xtriage
Refinement program	REFMAC 5.8.0425	Depositor
R, R_{free}	0.151 , 0.169<br>0.153 , 0.169	Depositor<br>DCC
R_{free} test set	6464 reflections (5.09%)	wwPDB-VP
Wilson B-factor (Å^2)	9.6	Xtriage
Anisotropy	0.103	Xtriage
Bulk solvent k_{sol}(e/Å^3), B_{sol}(Å^2)	0.39 , 31.6	EDS
L-test for twinning[2]	$< \|L\| >$ = 0.49, $< L^2 >$ = 0.32	Xtriage
Estimated twinning fraction	0.016 for -h,l,k	Xtriage
F_o,F_c correlation	0.98	EDS
Total number of atoms	8072	wwPDB-VP
Average B, all atoms (Å^2)	12.0	wwPDB-VP

Xtriage's analysis on translational NCS is as follows: *The largest off-origin peak in the Patterson function is 6.71% of the height of the origin peak. No significant pseudotranslation is detected.*

[1] Intensities estimated from amplitudes.

[2] Theoretical values of $< |L| >$, $< L^2 >$ for acentric reflections are 0.5, 0.333 respectively for untwinned datasets, and 0.375, 0.2 for perfectly twinned datasets.

For Manuscript Review

5 Model quality (i)

5.1 Standard geometry (i)

Bond lengths and bond angles in the following residue types are not validated in this section: TAM, SF4, PG4, 402

The Z score for a bond length (or angle) is the number of standard deviations the observed value is removed from the expected value. A bond length (or angle) with $|Z| > 5$ is considered an outlier worth inspection. RMSZ is the root-mean-square of all Z scores of the bond lengths (or angles).

Mol	Chain	Bond lengths		Bond angles	
		RMSZ	$\#\|Z\| > 5$	RMSZ	$\#\|Z\| > 5$
1	B	0.70	1/767 (0.1%)	0.99	1/1038 (0.1%)
2	A	0.67	4/3178 (0.1%)	1.04	19/4298 (0.4%)
All	All	0.67	5/3945 (0.1%)	1.03	20/5336 (0.4%)

Chiral center outliers are detected by calculating the chiral volume of a chiral center and verifying if the center is modelled as a planar moiety or with the opposite hand.A planarity outlier is detected by checking planarity of atoms in a peptide group, atoms in a mainchain group or atoms of a sidechain that are expected to be planar.

Mol	Chain	#Chirality outliers	#Planarity outliers
2	A	0	2

All (5) bond length outliers are listed below:

Mol	Chain	Res	Type	Atoms	Z	Observed(Å)	Ideal(Å)
2	A	269	LYS	CD-CE	7.79	1.70	1.51
1	B	101	GLU	CD-OE2	-6.23	1.18	1.25
2	A	54[A]	MET	CG-SD	-6.23	1.65	1.81
2	A	54[B]	MET	CG-SD	-6.23	1.65	1.81
2	A	24	LYS	CE-NZ	-5.31	1.35	1.49

All (20) bond angle outliers are listed below:

Mol	Chain	Res	Type	Atoms	Z	Observed(^{o})	Ideal(^{o})
2	A	247	LYS	CD-CE-NZ	11.79	138.81	111.70
2	A	54[A]	MET	CG-SD-CE	-10.28	83.75	100.20
2	A	54[B]	MET	CG-SD-CE	-10.28	83.75	100.20
2	A	355	ARG	NE-CZ-NH2	-7.80	116.40	120.30
2	A	286	MET	CG-SD-CE	-7.45	88.28	100.20
2	A	355	ARG	NH1-CZ-NH2	6.40	126.44	119.40

Continued on next page...

For Manuscript Review

Continued from previous page...

Mol	Chain	Res	Type	Atoms	Z	Observed(°)	Ideal(°)
2	A	54[A]	MET	CB-CG-SD	6.30	131.29	112.40
2	A	54[B]	MET	CB-CG-SD	6.30	131.29	112.40
2	A	7	GLU	CG-CD-OE2	-6.29	105.71	118.30
2	A	317	LYS	N-CA-CB	-6.29	99.28	110.60
2	A	355	ARG	NE-CZ-NH1	-6.29	117.16	120.30
2	A	8[A]	ARG	NE-CZ-NH2	-6.07	117.26	120.30
2	A	8[B]	ARG	NE-CZ-NH2	-6.07	117.26	120.30
2	A	7	GLU	CG-CD-OE1	5.39	129.08	118.30
2	A	372	PHE	CB-CG-CD1	5.36	124.55	120.80
1	B	101	GLU	OE1-CD-OE2	5.30	129.66	123.30
2	A	56	GLU	CG-CD-OE1	5.19	128.68	118.30
2	A	148[A]	THR	CA-CB-CG2	-5.18	105.15	112.40
2	A	148[B]	THR	CA-CB-CG2	-5.18	105.15	112.40
2	A	163	ARG	CD-NE-CZ	5.11	130.75	123.60

There are no chirality outliers.

All (2) planarity outliers are listed below:

Mol	Chain	Res	Type	Group
2	A	218	ARG	Sidechain
2	A	243	ARG	Sidechain

5.2 Too-close contacts ⓘ

In the following table, the Non-H and H(model) columns list the number of non-hydrogen atoms and hydrogen atoms in the chain respectively. The H(added) column lists the number of hydrogen atoms added and optimized by MolProbity. The Clashes column lists the number of clashes within the asymmetric unit, whereas Symm-Clashes lists symmetry-related clashes.

Mol	Chain	Non-H	H(model)	H(added)	Clashes	Symm-Clashes
1	B	727	734	717	5	2
2	A	3066	3063	3028	17	2
3	A	17	7	5	0	0
4	A	24	0	0	1	0
5	A	11	17	16	6	0
6	A	13	18	18	2	0
7	A	299	0	0	4	0
7	B	76	0	0	2	0
All	All	4233	3839	3784	23	2

The all-atom clashscore is defined as the number of clashes found per 1000 atoms (including

For Manuscript Review

hydrogen atoms). The all-atom clashscore for this structure is 3.

All (23) close contacts within the same asymmetric unit are listed below, sorted by their clash magnitude.

Atom-1	Atom-2	Interatomic distance (Å)	Clash overlap (Å)
5:A:505:TAM:O5	7:A:601:HOH:O	1.84	0.95
1:B:66[A]:THR:HG22	7:B:259:HOH:O	1.74	0.88
2:A:227:TYR:HD1	5:A:505:TAM:H61	1.42	0.83
2:A:354:LYS:HE3	7:A:607:HOH:O	1.89	0.72
2:A:105[B]:MET:HE3	2:A:204:ILE:HD11	1.76	0.66
2:A:47:THR:C	6:A:506:PG4:H12	2.18	0.63
2:A:227:TYR:CD1	5:A:505:TAM:H61	2.31	0.60
2:A:7:GLU:O	2:A:8[A]:ARG:HB2	2.04	0.57
2:A:255:ASP:OD1	5:A:505:TAM:H52	2.08	0.54
2:A:295:ILE:O	2:A:301:GLY:HA3	2.10	0.51
2:A:194:LEU:N	2:A:195[B]:PRO:CD	2.74	0.50
5:A:505:TAM:O6	5:A:505:TAM:H21	2.12	0.49
1:B:116:PHE:CE2	2:A:48:ALA:HA	2.49	0.48
1:B:97:LYS:HB3	1:B:97:LYS:HE3	1.61	0.46
2:A:136:LYS:HG3	7:A:705:HOH:O	2.16	0.46
5:A:505:TAM:H62	7:A:839:HOH:O	2.17	0.45
2:A:7:GLU:O	2:A:8[A]:ARG:CB	2.65	0.44
1:B:116:PHE:CD2	2:A:48:ALA:HA	2.54	0.43
2:A:267:ILE:HG23	2:A:272:ILE:HB	2.03	0.41
1:B:66[A]:THR:CG2	7:B:259:HOH:O	2.51	0.41
2:A:180:PRO:HD2	4:A:502:SF4:S3	2.61	0.41
2:A:51:PHE:O	2:A:58:HIS:HA	2.21	0.40
2:A:47:THR:HA	6:A:506:PG4:H12	2.04	0.40

All (2) symmetry-related close contacts are listed below. The label for Atom-2 includes the symmetry operator and encoded unit-cell translations to be applied.

Atom-1	Atom-2	Interatomic distance (Å)	Clash overlap (Å)
1:B:36:VAL:H2	2:A:343:THR:OG1[3_655]	1.42	0.18
1:B:36:VAL:N	2:A:343:THR:OG1[3_655]	2.07	0.13

5.3 Torsion angles (i)

5.3.1 Protein backbone (i)

In the following table, the Percentiles column shows the percent Ramachandran outliers of the chain as a percentile score with respect to all X-ray entries followed by that with respect to entries

For Manuscript Review

of similar resolution.

The Analysed column shows the number of residues for which the backbone conformation was analysed, and the total number of residues.

Mol	Chain	Analysed	Favoured	Allowed	Outliers	Percentiles	
1	B	90/88 (102%)	88 (98%)	2 (2%)	0	100	100
2	A	405/405 (100%)	396 (98%)	9 (2%)	0	100	100
All	All	495/493 (100%)	484 (98%)	11 (2%)	0	100	100

There are no Ramachandran outliers to report.

5.3.2 Protein sidechains (i)

In the following table, the Percentiles column shows the percent sidechain outliers of the chain as a percentile score with respect to all X-ray entries followed by that with respect to entries of similar resolution.

The Analysed column shows the number of residues for which the sidechain conformation was analysed, and the total number of residues.

Mol	Chain	Analysed	Rotameric	Outliers	Percentiles	
1	B	80/76 (105%)	79 (99%)	1 (1%)	65	29
2	A	332/330 (101%)	327 (98%)	5 (2%)	60	25
All	All	412/406 (102%)	406 (98%)	6 (2%)	65	25

All (6) residues with a non-rotameric sidechain are listed below:

Mol	Chain	Res	Type
1	B	57	PHE
2	A	148[A]	THR
2	A	148[B]	THR
2	A	167	LYS
2	A	281	LYS
2	A	294	THR

Sometimes sidechains can be flipped to improve hydrogen bonding and reduce clashes. There are no such sidechains identified.

5.3.3 RNA (i)

There are no RNA molecules in this entry.

For Manuscript Review

5.4 Non-standard residues in protein, DNA, RNA chains (i)

There are no non-standard protein/DNA/RNA residues in this entry.

5.5 Carbohydrates (i)

There are no oligosaccharides in this entry.

5.6 Ligand geometry (i)

6 ligands are modelled in this entry.

In the following table, the Counts columns list the number of bonds (or angles) for which Mogul statistics could be retrieved, the number of bonds (or angles) that are observed in the model and the number of bonds (or angles) that are defined in the Chemical Component Dictionary. The Link column lists molecule types, if any, to which the group is linked. The Z score for a bond length (or angle) is the number of standard deviations the observed value is removed from the expected value. A bond length (or angle) with $|Z| > 2$ is considered an outlier worth inspection. RMSZ is the root-mean-square of all Z scores of the bond lengths (or angles).

Mol	Type	Chain	Res	Link	Bond lengths			Bond angles		
					Counts	RMSZ	$\#\|Z\| > 2$	Counts	RMSZ	$\#\|Z\| > 2$
6	PG4	A	506	-	12,12,12	0.22	0	11,11,11	0.87	0
4	SF4	A	503	2	0,12,12	-	-	-		
3	402	A	501	2	13,19,19	4.45	4 (30%)	2,36,36	1.17	0
4	SF4	A	502	2	0,12,12	-	-	-		
5	TAM	A	505	-	7,10,10	1.49	1 (14%)	9,12,12	1.65	2 (22%)
4	SF4	A	504	2	0,12,12	-	-	-		

In the following table, the Chirals column lists the number of chiral outliers, the number of chiral centers analysed, the number of these observed in the model and the number defined in the Chemical Component Dictionary. Similar counts are reported in the Torsion and Rings columns. '-' means no outliers of that kind were identified.

Mol	Type	Chain	Res	Link	Chirals	Torsions	Rings
6	PG4	A	506	-	-	8/10/10/10	-
4	SF4	A	503	2	-	-	0/6/5/5
3	402	A	501	2	-	-	0/5/3/3
5	TAM	A	505	-	-	2/12/12/12	-
4	SF4	A	502	2	-	-	0/6/5/5
4	SF4	A	504	2	-	-	0/6/5/5

All (5) bond length outliers are listed below:

For Manuscript Review

Mol	Chain	Res	Type	Atoms	Z	Observed(Å)	Ideal(Å)
3	A	501	402	C1-S1	-11.11	1.65	1.85
3	A	501	402	C2-S2	-8.63	1.69	1.85
3	A	501	402	O5-C5	6.18	1.28	1.17
3	A	501	402	C4-N4	-3.14	1.09	1.15
5	A	505	TAM	C1-C4	-2.78	1.46	1.52

All (2) bond angle outliers are listed below:

Mol	Chain	Res	Type	Atoms	Z	Observed(^{o})	Ideal(^{o})
5	A	505	TAM	O6-C6-C3	2.91	119.16	111.39
5	A	505	TAM	C3-C-N	2.15	114.22	108.09

There are no chirality outliers.

All (10) torsion outliers are listed below:

Mol	Chain	Res	Type	Atoms
5	A	505	TAM	C-C1-C4-O4
6	A	506	PG4	O2-C3-C4-O3
6	A	506	PG4	O1-C1-C2-O2
6	A	506	PG4	O4-C7-C8-O5
6	A	506	PG4	O3-C5-C6-O4
6	A	506	PG4	C6-C5-O3-C4
6	A	506	PG4	C5-C6-O4-C7
6	A	506	PG4	C4-C3-O2-C2
6	A	506	PG4	C3-C4-O3-C5
5	A	505	TAM	C1-C-C3-C6

There are no ring outliers.

3 monomers are involved in 9 short contacts:

Mol	Chain	Res	Type	Clashes	Symm-Clashes
6	A	506	PG4	2	0
4	A	502	SF4	1	0
5	A	505	TAM	6	0

The following is a two-dimensional graphical depiction of Mogul quality analysis of bond lengths, bond angles, torsion angles, and ring geometry for all instances of the Ligand of Interest. In addition, ligands with molecular weight > 250 and outliers as shown on the validation Tables will also be included. For torsion angles, if less then 5% of the Mogul distribution of torsion angles is within 10 degrees of the torsion angle in question, then that torsion angle is considered an outlier. Any bond that is central to one or more torsion angles identified as an outlier by Mogul will be highlighted in the graph. For rings, the root-mean-square deviation (RMSD) between the ring

For Manuscript Review

in question and similar rings identified by Mogul is calculated over all ring torsion angles. If the average RMSD is greater than 60 degrees and the minimal RMSD between the ring in question and any Mogul-identified rings is also greater than 60 degrees, then that ring is considered an outlier. The outliers are highlighted in purple. The color gray indicates Mogul did not find sufficient equivalents in the CSD to analyse the geometry.

Ligand SF4 A 503

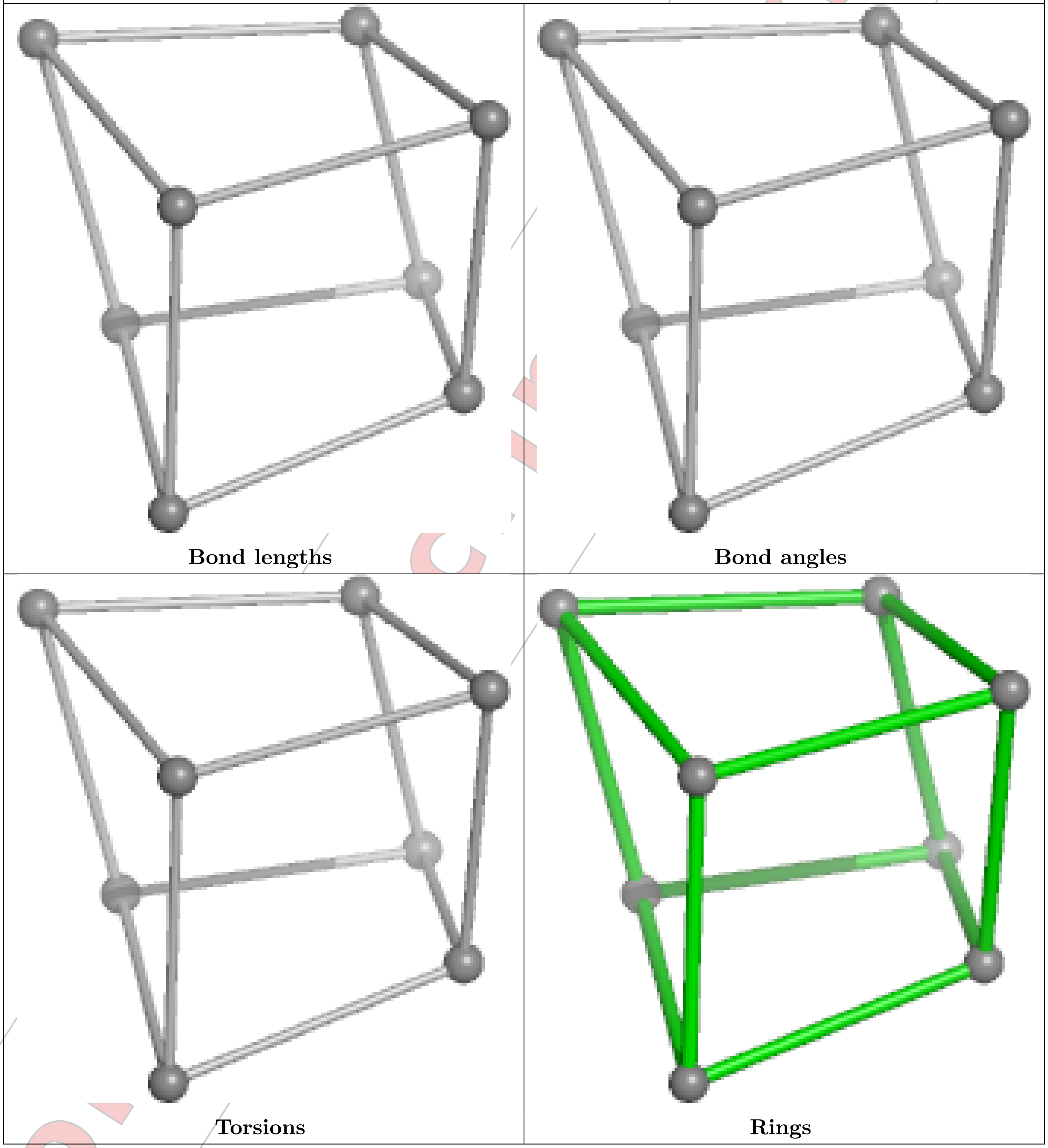

Ligand 402 A 501

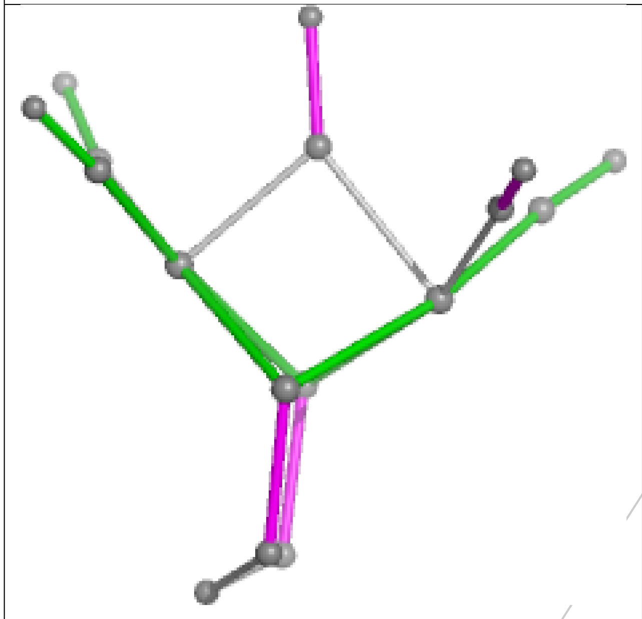

Bond lengths

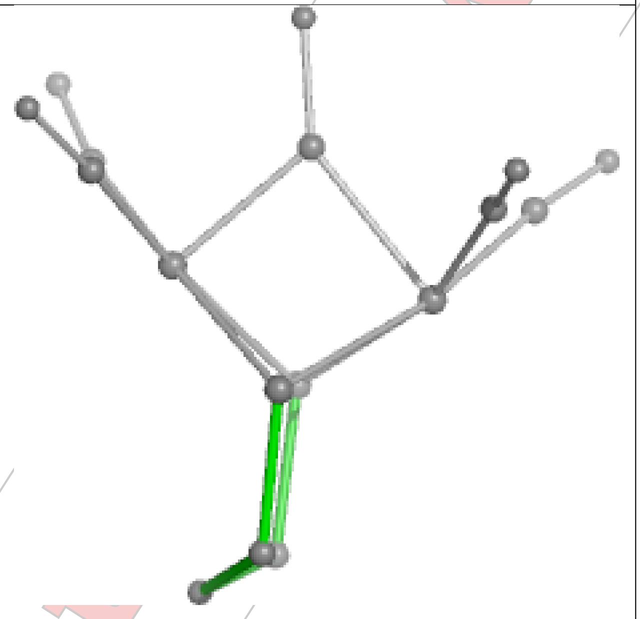

Bond angles

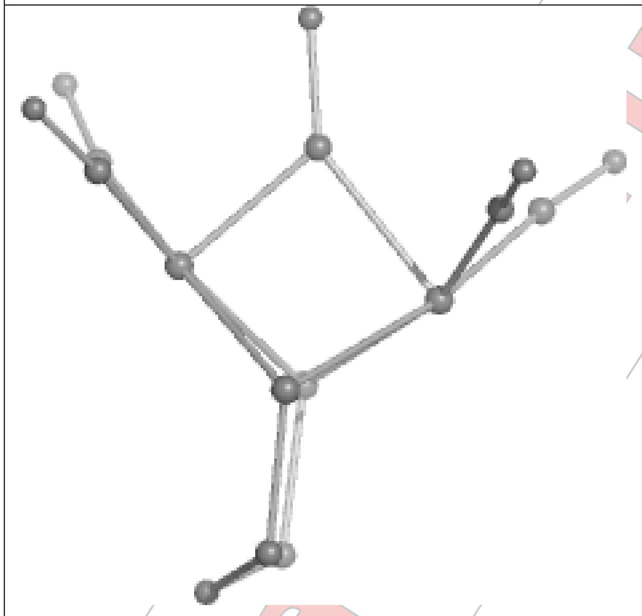

Torsions

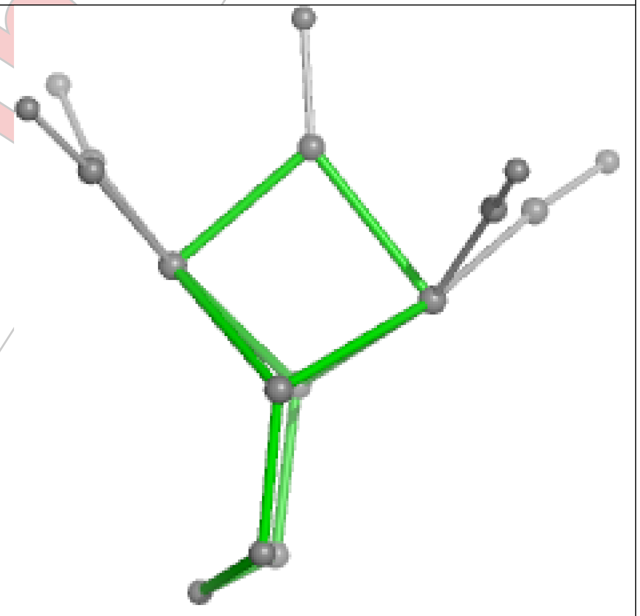

Rings

For Manuscript Review

Ligand SF4 A 502

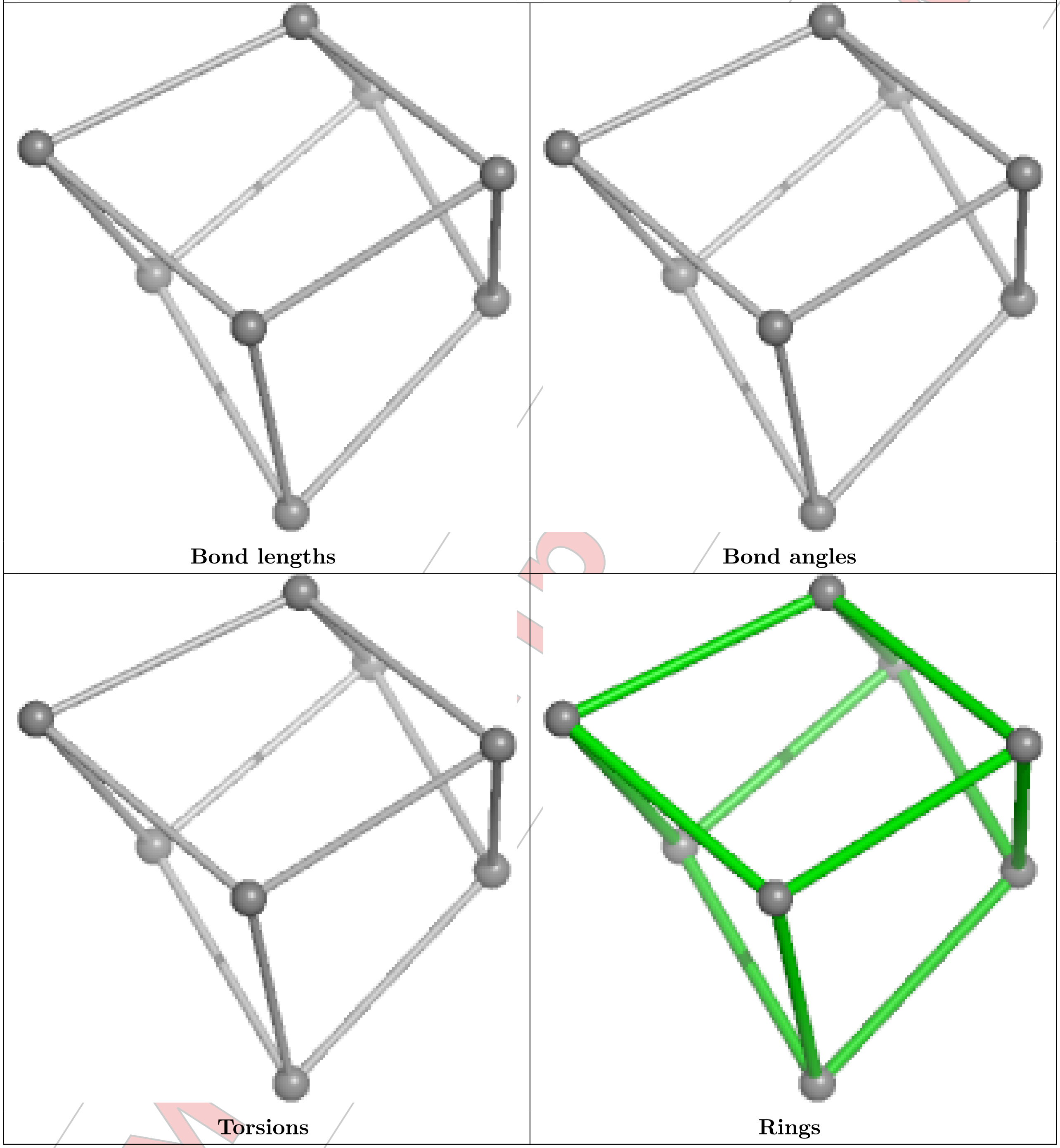

For Manuscript Review

Ligand SF4 A 504

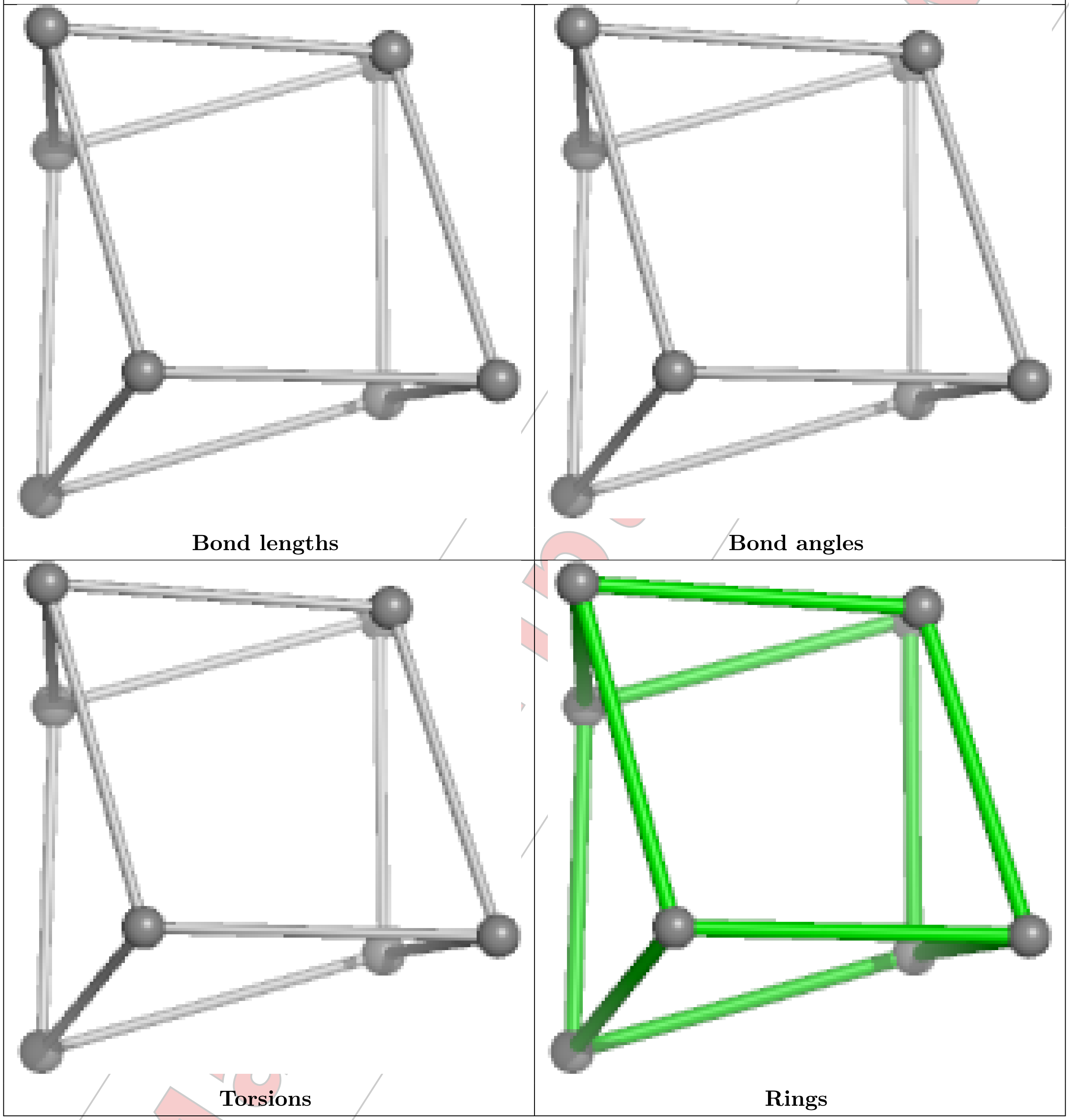

5.7 Other polymers (i)

There are no such residues in this entry.

5.8 Polymer linkage issues (i)

There are no chain breaks in this entry.

For Manuscript Review

6 Fit of model and data (i)

6.1 Protein, DNA and RNA chains (i)

In the following table, the column labelled '#RSRZ> 2' contains the number (and percentage) of RSRZ outliers, followed by percent RSRZ outliers for the chain as percentile scores relative to all X-ray entries and entries of similar resolution. The OWAB column contains the minimum, median, 95th percentile and maximum values of the occupancy-weighted average B-factor per residue. The column labelled 'Q< 0.9' lists the number of (and percentage) of residues with an average occupancy less than 0.9.

Mol	Chain	Analysed	<RSRZ>	#RSRZ>2			OWAB(Å^2)	Q<0.9
1	B	88/88 (100%)	0.19	2 (2%)	61	63	5, 12, 21, 25	5 (5%)
2	A	396/405 (97%)	-0.08	8 (2%)	64	68	4, 10, 20, 46	25 (6%)
All	All	484/493 (98%)	-0.03	10 (2%)	63	67	4, 11, 21, 46	30 (6%)

All (10) RSRZ outliers are listed below:

Mol	Chain	Res	Type	RSRZ
2	A	397	ALA	5.5
2	A	343	THR	3.5
2	A	164	LEU	3.5
2	A	341	GLY	3.0
1	B	36	VAL	2.7
2	A	396	GLU	2.6
1	B	85	HIS	2.5
2	A	369	PRO	2.4
2	A	340	VAL	2.0
2	A	367	LYS	2.0

6.2 Non-standard residues in protein, DNA, RNA chains (i)

There are no non-standard protein/DNA/RNA residues in this entry.

6.3 Carbohydrates (i)

There are no monosaccharides in this entry.

For Manuscript Review

6.4 Ligands (i)

In the following table, the Atoms column lists the number of modelled atoms in the group and the number defined in the chemical component dictionary. The B-factors column lists the minimum, median, 95th percentile and maximum values of B factors of atoms in the group. The column labelled 'Q< 0.9' lists the number of atoms with occupancy less than 0.9.

Mol	Type	Chain	Res	Atoms	RSCC	RSR	B-factors(Å^2)	Q<0.9
6	PG4	A	506	13/13	0.76	0.17	31,38,50,51	2
5	TAM	A	505	11/11	0.84	0.13	12,20,34,40	5
3	402	A	501	17/17	0.97	0.09	4,9,12,12	24
4	SF4	A	504	8/8	1.00	0.03	8,9,9,9	0
4	SF4	A	502	8/8	1.00	0.02	6,7,7,7	0
4	SF4	A	503	8/8	1.00	0.02	7,7,7,7	0

The following is a graphical depiction of the model fit to experimental electron density of all instances of the Ligand of Interest. In addition, ligands with molecular weight > 250 and outliers as shown on the geometry validation Tables will also be included. Each fit is shown from different orientation to approximate a three-dimensional view.

For Manuscript Review

Electron density around 402 A 501:

2mF$_o$-DF$_c$ (at 0.7 rmsd) in gray
mF$_o$-DF$_c$ (at 3 rmsd) in purple (negative)
and green (positive)

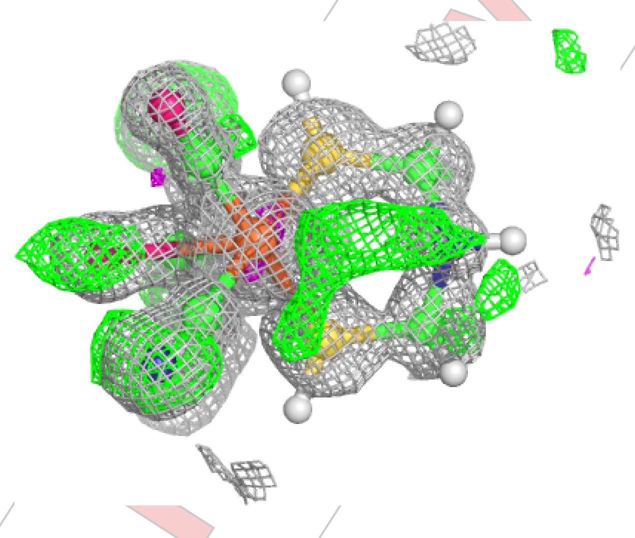

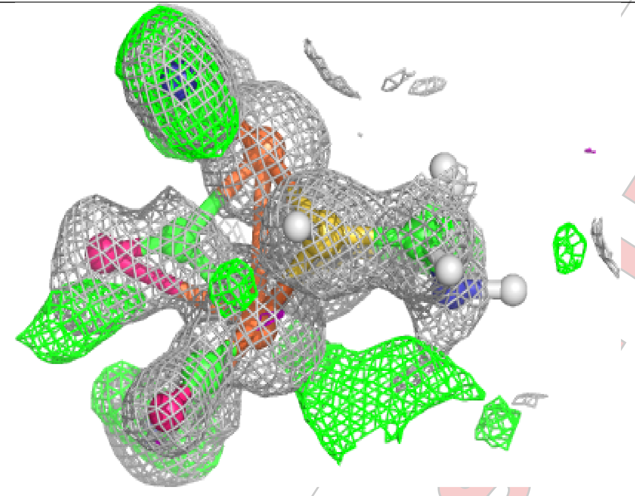

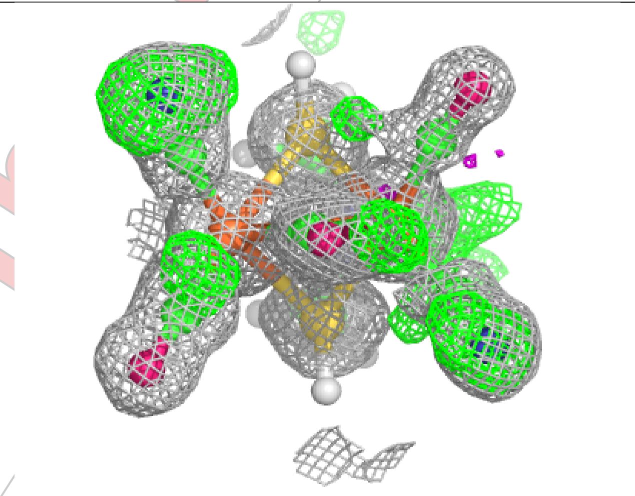

For Manuscript Review

Electron density around SF4 A 504:

$2mF_o$-DF_c (at 0.7 rmsd) in gray
mF_o-DF_c (at 3 rmsd) in purple (negative)
and green (positive)

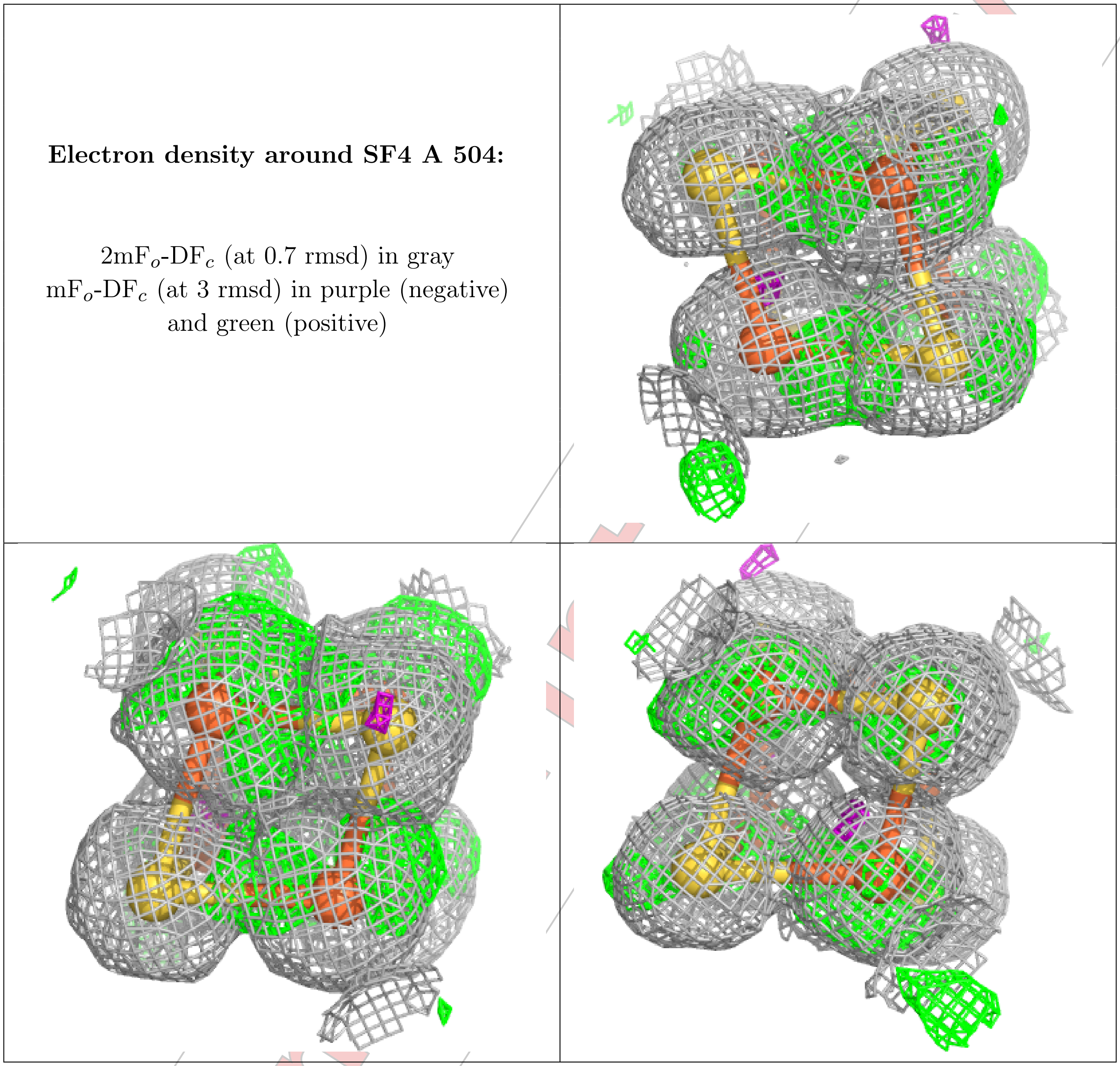

For Manuscript Review

Electron density around SF4 A 502:

2mF$_o$-DF$_c$ (at 0.7 rmsd) in gray
mF$_o$-DF$_c$ (at 3 rmsd) in purple (negative)
and green (positive)

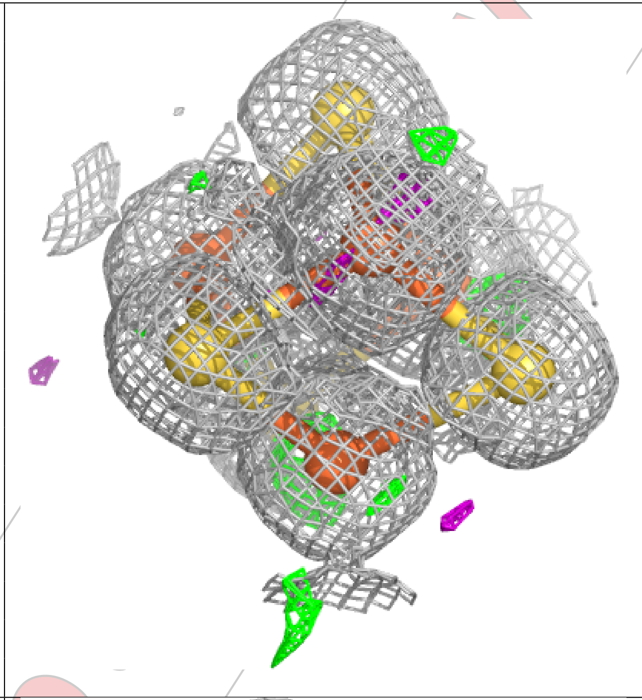

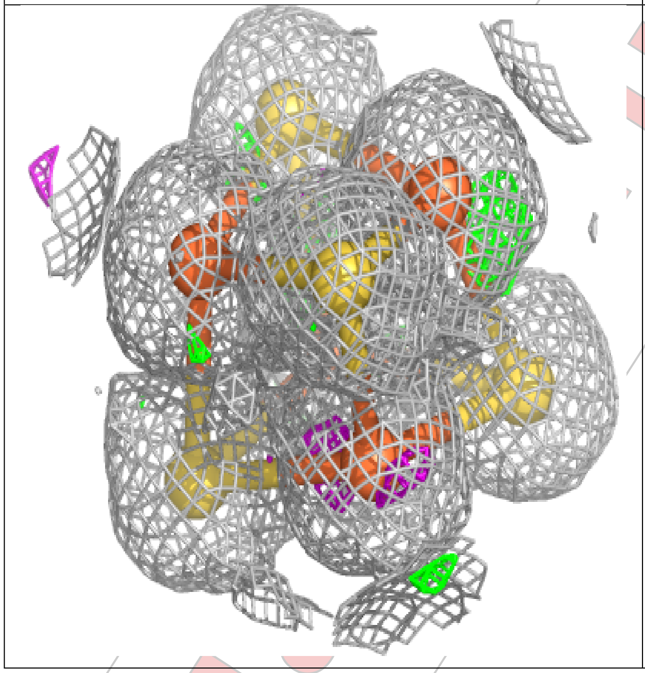

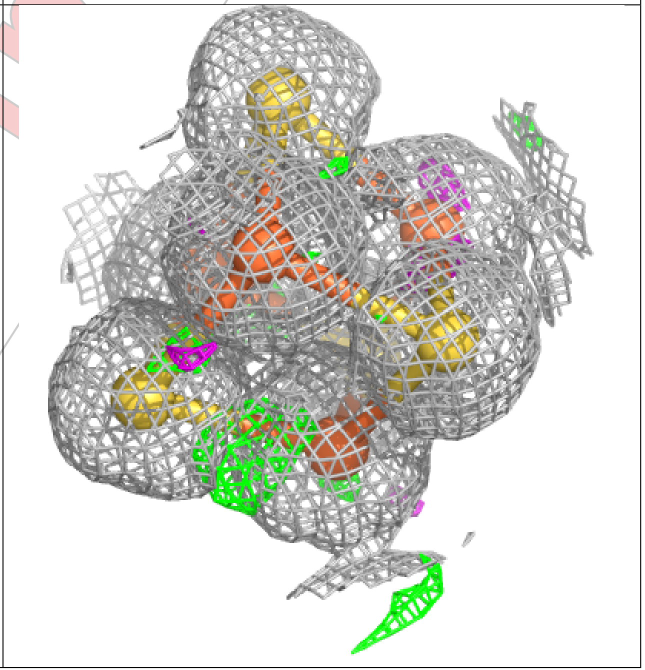

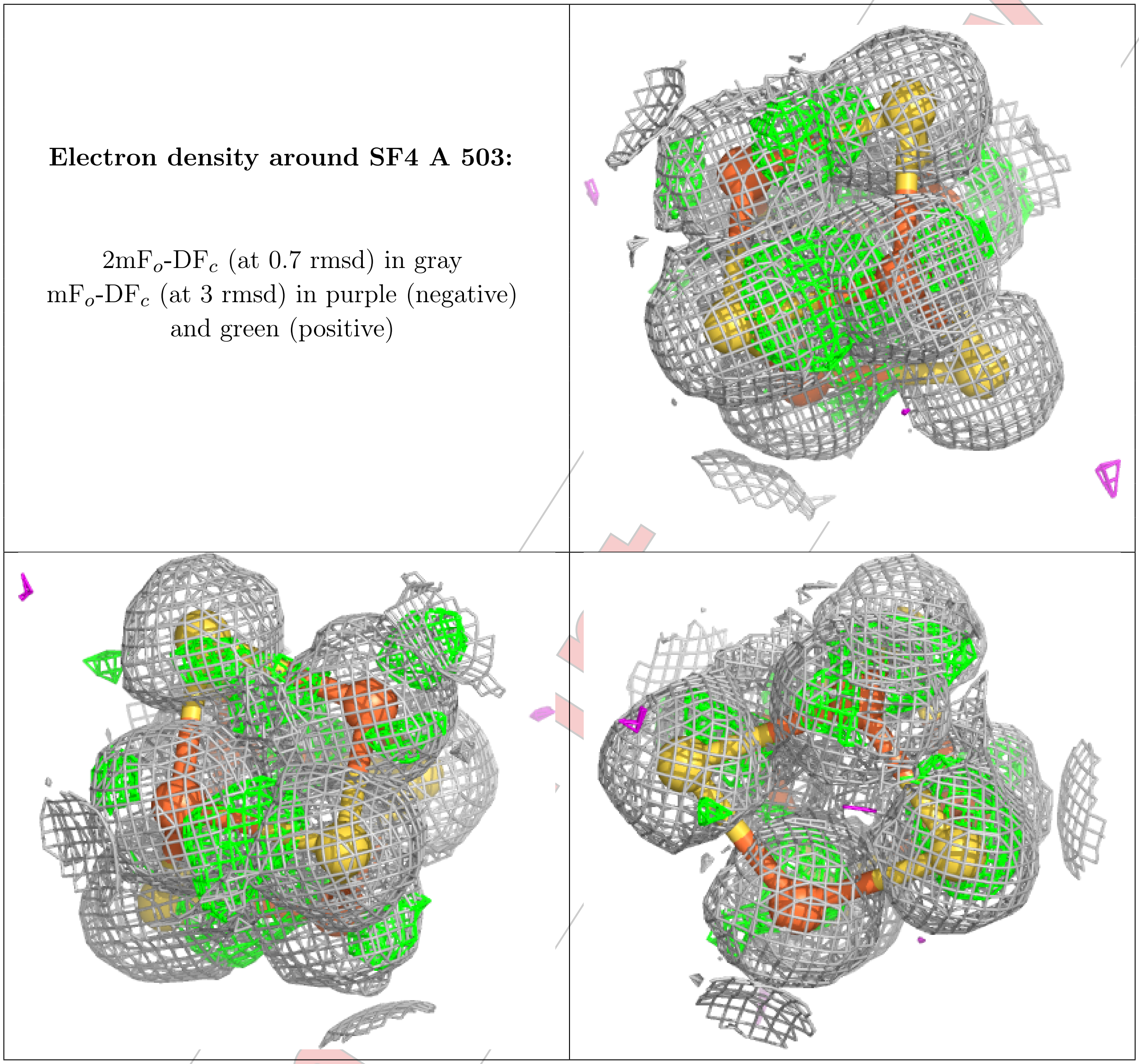

Electron density around SF4 A 503:

2mF$_o$-DF$_c$ (at 0.7 rmsd) in gray
mF$_o$-DF$_c$ (at 3 rmsd) in purple (negative)
and green (positive)

6.5 Other polymers ⓘ

There are no such residues in this entry.

For Manuscript Review
