# Supplementary material for: Subunit fusion unlocks rapid in vitro maturation for slowly activating heterodimeric [FeFe]-hydrogenases
Source: Chem Sci. 2026 Feb 3;17(15):7678–89. doi: 10.1039/d5sc07299a (PMC12933642; doi:10.1039/d5sc07299a)
Supplement: SC-017-D5SC07299A-s003 [file SC-017-D5SC07299A-s003.pdf]

Supplementary Information (SI) for Chemical Science.
This journal is © The Royal Society of Chemistry 2026

Supplementary Information 2

Subunit fusion unlocks rapid in vitro maturation for slowly activating heterodimeric [FeFe]-hydrogenases

Jan Jaenecke [a], Konstantin Bikbaev[b], Miriam Malagnini[c], Julia Bronold[a], Shanika Yadav[d], Ulf-Peter Apfel[d,e], Christophe Léger[c], James A. Birrell[f], Ingrid Span[b], Nicolas Plumeré[a*], Martin Winkler [a,g*]

Genomic data and polypeptide sequence features of large and small subunits in heterodimeric (periplasmic) M2-type [FeFe]-hydrogenases

a. *Technical University of Munich, Campus Straubing for Biotechnology and Sustainability, Professorship for Electrobiotechnology, Uferstrasse 53, 94315 Straubing, Germany.*
b. *Friedrich-Alexander-Universität Erlangen-Nürnberg, Bioinorganic Chemistry, Egerlandstrasse 1, 91058 Erlangen, Germany.*
c. *Aix Marseille Univ, CNRS, Laboratoire de Bioénergétique et Ingénierie des Protéines, 13009 Marseille, France*
d. *Activation of Small Molecules/Technical Electrochemistry, Faculty of Chemistry and Biochemistry, Ruhr-University Bochum, Universitätsstrasse 150, 44801 Bochum, Germany*
e. *Department of Electrosynthesis, Fraunhofer UMSICHT, 46047 Oberhausen, Germany*
f. *School of Life Sciences, University of Essex, Wivenhoe Park, Colchester, CO4 3SQ, UK*
g. *g. Inorganic Spectroscopy, Energy Converting Enzymes, Max-Planck-Institute for Chemical Energy Conversion, Stiftstrasse 34-36, 45470 Mühlheim an der Ruhr, Germany*
* Corresponding authors: martin-h.winkler@tum.de, nicolas.plumere@tum.de

I.) Phylogeny and putative origin of periplasmic M2-type [FeFe]-hydrogenases

II.) Annotated Genomic and Polypeptide Sequences

1.) *Desulfovibrio vulgaris subsp. vulgaris* str. Hildenborough (δ-Proteobacteria)

2.) *Desulfonauticus submarinus* strain DSM 15269 (δ-Proteobacteria)

3.) *Campylobacter rectus* strain ATCC 33238 (ε-Proteobacteria)

4.) *Sutterella wadsworthensis* (β-Proteobacteria)

5.) *Thermodesulfovibrio yellowstonii* DSM 11347 (Nitrospira)

6.) *Sporomusa ovata* (Bacillota; Negativicutes)

7.) *Syntrophomonas zehnderi* OL4 (Bacillota; Clostridia)

8.) *Dehalobacter sp.* TBBPA1 (Bacillota; Clostridia)

III.) Sequence alignments and annotations

a.) LSUs

b.) SSUs

IV.) Structure models and possible implications for *in vivo* maturation

1.) DdH of *Desulfovibrio vulgaris subsp. vulgaris* str. Hildenborough (δ-Proteobacteria)

2.) HydA of *Campylobacter rectus* strain ATCC 33238 (ε-Proteobacteria)

3.) HydA of *Sporomusa ovata* (Bacillota; Negativicutes)

4.) *Dehalobacter sp.* TBBPA1 (Bacillota; Clostridia)

I. Phylogeny and putative origin of periplasmic M2-type [FeFe]-hydrogenases

1. Succession and short intergenic segment between LSU and SSU genes

The genome of *Desulfovibrio vulgaris* encodes two [FeFe]-hydrogenases in close distance from each other, one heterodimeric and the other one monomeric (*Dd*HydA-γ (GenBank: AAS96248.1) (see SI2 section II.1). The genes of $2Fe_H$ subcluster maturases HydF, HydE and HydG are located nearby. The heterodimeric *Dd*HydAB which is encoded by the consecutive genes *LSU* and *SSU*, is known to be translocated into the periplasm.[1] The N-terminus of the SSU clearly carries a canonical Tat (twin-arginine translocation) transporter signal, which is required for the translocation of a fully maturated enzyme to the periplasm.[2] It usually consists of the motif **S/T-RRxFLK** (n-region), being followed by a hydrophobic region (h-region) and a signal peptidase recognition site similar to **A**-x-**A** (C- region) for cleavage after transport.[3] This Tat-transporter signal is lacking in the monomeric M3-type [FeFe]-hydrogenase *Dd*HydA-γ and in all $2Fe_H$ maturases, suggesting their location and functional purpose being confined to the cytoplasm. The consecutive genes for the LSU and SSU of *Dd*HydAB are separated by merely 11 nucleotides (between the stop codon (TAG) of the ORF encoding the LSU and the start codon (ATG) of the *SSU* gene). In between there is a characteristic Shine-Dalgarno (SD) sequence with a spacer of 7 nucleotides (nt) relative to the start codon of the LSU, being within the usual range of 5-10 nt.

Interestingly, the separation into LSU and SSU as well as the close genomic succession of both genes seems to be a general feature of [FeFe]-hydrogenases that exhibit a Tat-transporter signal. To support the hypothesis that this genetic constellation is characteristic for periplasmic [FeFe]-hydrogenases, we collected and compared genomic and polypeptide sequence data for further heterodimeric [FeFe]-hydrogenases of subtype M2 (Supplementary Information 2; section II). Just as for *Dd*HydAB^{WT}, the 7 further cases that were examined in more detail, show the consecutive genes for LSU and SSU with only a short segment in between that includes a typical Shine-Dalgarno sequence (analyzed via RBS Calculator[4]) which is consistent with a strong ribosome retention after LSU translation and an immediate reinitiation of translation at the SSU gene (section II.2-8). This conserved configuration enables a near-stoichiometric co-expression of LSU and SSU, rendering the translation of the *SSU* gene strongly dependent

on a preceding translation of the upstream *LSU* gene. In all cases only the SSU sequence carries a characteristic Tat transporter signal, suggesting that the strict co-expression of both subunits may serve the purpose of limiting the SSU fraction that is independently translocated to the periplasm.

2. How rapid maturation can be accomplished *in vivo* in the cytoplasmic pre-state

According to a signal peptide screening via the online prediction platform SignalP6 [5], neither of the three H-cluster maturases (Hyd-E,-G and -F) carries any signal peptide for a translocation into the periplasmic space (section II-1c), suggesting that the biosynthesis and transfer of the $2Fe_H$ precursor from loaded-HydF to “apo”-hydrogenase already occurs within the cytoplasm and thus prior to the enzyme’s export into the periplasm. Furthermore, peptide segments of both subunits are required for the stable closure of the H-cluster binding site after $2Fe_H$-cofactor insertion. LSU and SSU of *Dd*HydAB therefore must be assembled in the cytoplasm to enable the export of the fully maturated enzyme.

The large subunit (LSU) of *Dd*HydAB originally carries a carboxy-terminal extension of 24 aa that is missing in the final holoenzyme which can be isolated from the periplasm[6] , suggesting it to be removed as soon as the enzyme enters the periplasmic space. However, only the polypeptide sequence of the small subunit (SSU) provides a typical N-terminal leader peptide for the twin-arginine translocation (Tat) pathway required for exporting fully folded and cofactor-containing protein into the periplasm before this 38 aa long segment is likewise proteolytically removed (section II-1c and Fig. SI2-2).[7]

In vivo, the uptake of the $2Fe_H$-precursor accordingly happens in the presence of both the N-terminal SSU-Tat-leader and the extended C-terminus of the LSU. *In silico* modelling experiments done with the AI-ML-tool Boltz-2 suggest that the termini that are lacking under the conditions of *in vitro* maturation investigated here, likely form alpha-helices which interact at a crossing point (see structures of the 5 best Boltz-2 [8] models in section IV, Fig. SI2-3(1)). During *in vivo* maturation, this contact site to the C-terminus of the LSU may affect the dynamics of lock reconfiguration in the SSU by providing the right balance between stability and flexibility needed to effectively support a fast lockage of the H-cluster binding site. Subunit-fusion likely attains a similar stabilization effect during $2Fe_H$-binding site lockage under the conditions of *in vitro* maturation.
The observation that the SSU of periplasmic [FeFe]-hydrogenases has evolved to provide a Tat transporter signal at its N-terminus and thus basically right at the start of the lock element that reconfigures after $2Fe_H$ insertion and binding site closure, while neither the LSU nor any $2Fe_H$ maturase carries such a signal may suggest the following hypothetical regulatory concept:

1.) After the concerted LSU- and SSU-gene expression, heterodimerization of LSU and SSU occurs in the cytosol, preventing an independent export of the SSU.
2.) H-cluster maturation and holo-enzyme formation happens in the cytosol as none of the maturases carries a corresponding signal peptide that would suggest them to be likewise exported into the periplasm.

3.) To prevent the futile export of the heterodimer in its inactive apo-state, the Tat-transporter system may only recognize the Tat signal on the SSU if both $2Fe_H$ cofactor insertion and binding site closure have successfully occurred. To enable this conditional export, the Tat-signal may be inaccessible prior to cofactor insertion.
4.) As the N-terminal peptide segment of the SSU and the C-terminal extension of the LSU are likely to interact they may very well provide the necessary support for the lock-element to effectively reconfigure. The structural rearrangement connected to the final lockage of the occupied binding site could in turn affect the structural features of the SSU's N-terminus, leading to the exposure of the formerly obstructed Tat-translocase signal. This would finally allow the export of the dimeric [FeFe]-hydrogenase only in its fully maturated holo-state. Albeit rather speculative, this hypothesis would offer a plausible explanation for the fact that the separation of the H-domain into LSU and SSU seems to be highly specific for periplasmic M2-type [FeFe]-hydrogenases.

3. Origin and distribution of heterodimeric M2 type [FeFe]-hydrogenases

To gain further insight into the distribution and origin of the heterodimeric M2-type [FeFe]-hydrogenases a phylogenetic tree was generated based on a multiple sequence alignment of 48 proteins, comprising 22 monomeric M2-type [FeFe]-hydrogenases that show no indication of a periplasmic location (i.e., they lack Sec or Tat-translocase signal peptides) and 26 LSU subunits of heterodimeric [FeFe]-hydrogenases. The corresponding genotypes of the latter group all show the characteristic LSU–SSU gene arrangement with short intergenic regions that carry the SD sequence (see below), and the N-termini of their SSUs provide a typical Tat translocase signal peptide (sections II.2-8). Nearly all heterodimeric M2-type [FeFe]-hydrogenases can be assigned to gram-negative species, including β-, γ-, and δ-Proteobacteria (the latter including *Dd*HydAB), as well as Nitrospira and Negativicutes. The class of Negativicutes represents a gram-negative subgroup within the otherwise gram-positive phylum of Bacillota (section I, Fig. SI2-1a). As exemplified for the [FeFe]-hydrogenases of *Campylobacter rectus* and *Sporomusa ovata,* which belong to the branches of ε-Proteobacteria and Negativicutes, the heterodimeric M2-subtype here seems to adopt a similar cytoplasmic pre-state with extensions on both subunits likely to interact during binding site closure (section IV, Figs. SI2-2 and SI2-3(2+3)). The periplasmic heterodimeric [FeFe]-hydrogenases appear to have a monophyletic origin. Mapping organisms that encode periplasmic M2-type [FeFe]-hydrogenases onto a 16S rRNA phylogram, relative to organisms that lack this heterodimeric type, suggests an origin in a phylogenetic branch that includes the aforementioned clades, in addition to the gram-positive Bacillota -most of which appear to have lost this subtype- and Cyanobacteria, which lack [FeFe]-hydrogenases in general (section I, Fig. SI2-1b). Species of the genera *Dehalobacter*, *Thermosynthropha*, and *Syntrophomonas*, which belong to the majority of gram-positive Bacillota, represent the only exceptions. They still possess the heterodimeric subtype including the N-terminal Tat-translocase signal peptide in the SSU. However, in contrast to other representatives of the M2 subtype, their LSU

isoforms appear to lack the characteristic C-terminal extension. As exemplified for the corresponding enzyme of *Dehalobater sp.* TBBPA1 in these cases the Tat-transporter signal peptide of the SSU seems to adopt a complex fold that may invoke similar structural flexibility features at the terminus of the lock element as assumed for the other cases with interacting extensions from both subunits (section IV, Fig. SI2-3(4)). These enzymes seem to be exported into the thin periplasmic space below the significantly thicker cell wall of gram-positive bacteria but may be too large (>50kDa) to permeate through the multiple peptidoglycan layers into the external medium and thus may still be an integral part of the periplasmic metabolism.

I.) Phylogeny and putative origin of periplasmic M2-type [FeFe]-hydrogenases

a

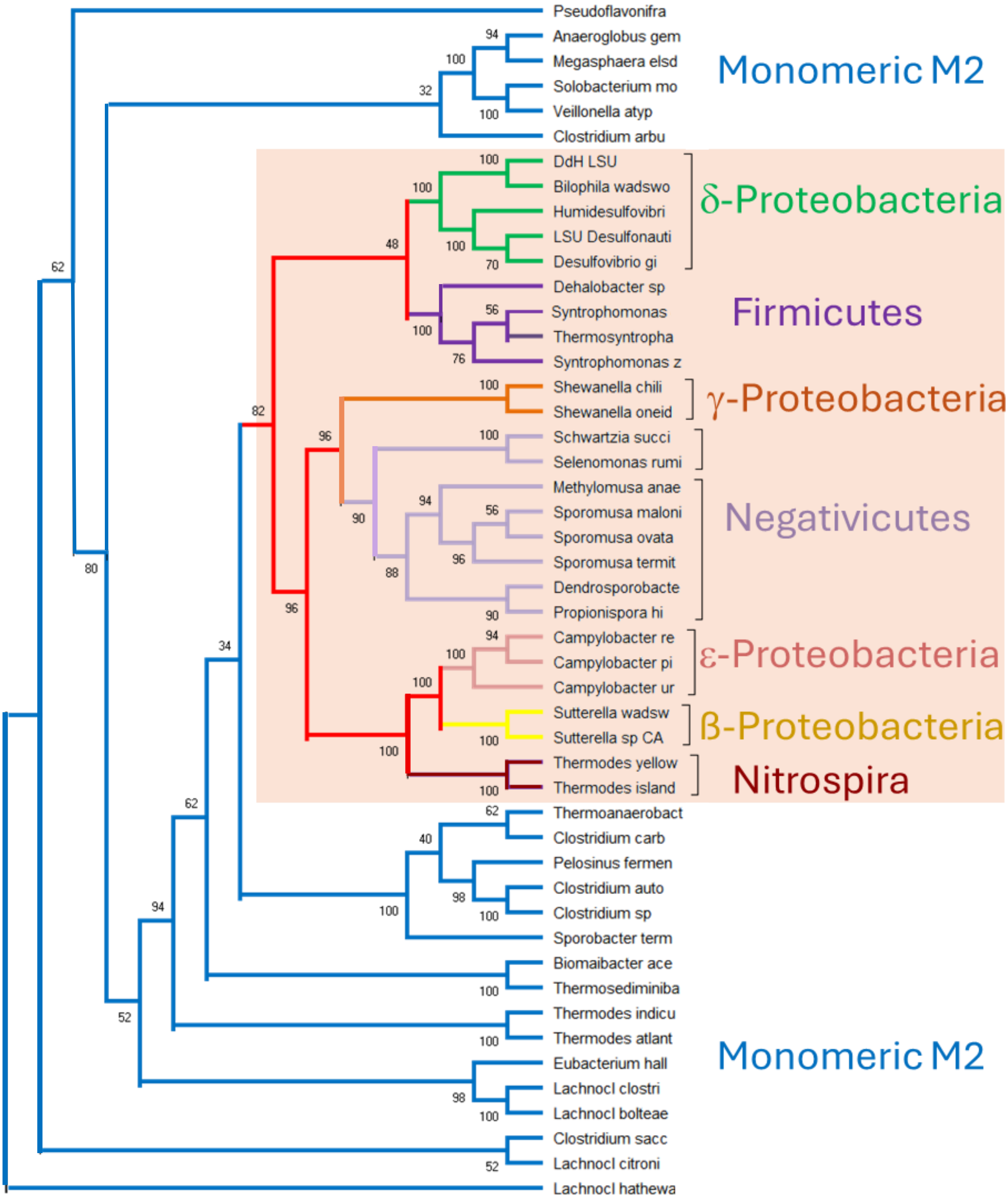

b

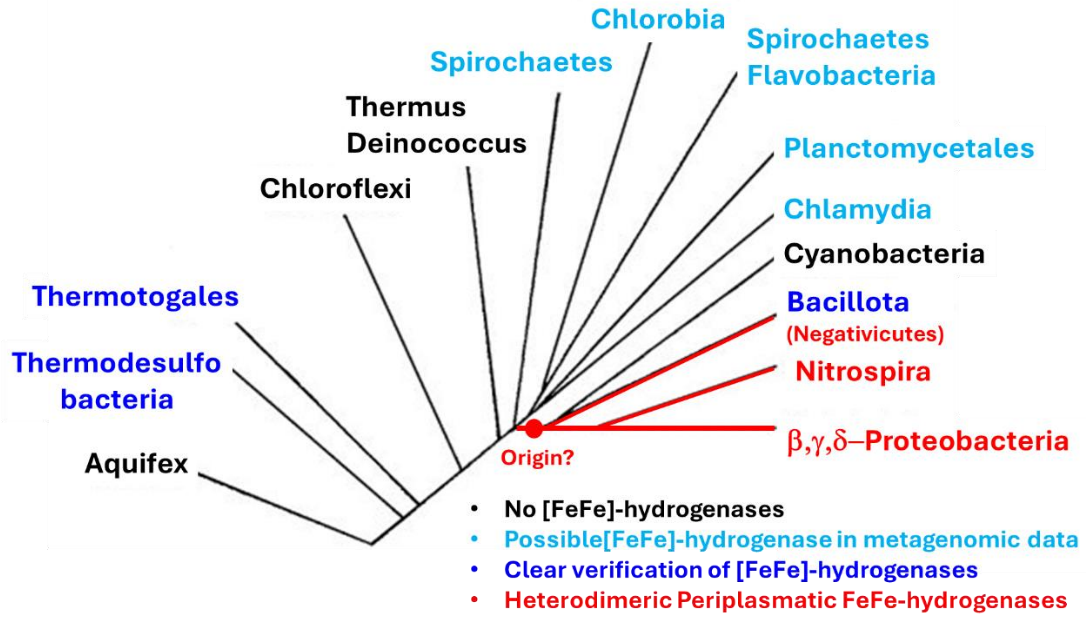

Fig. S2-1| Tracing the origin of periplasmic M2-type [FeFe]-hydrogenases.

(a) From a multiple sequence alignment of 22 monomeric [FeFe]-hydrogenases and the LSU sequences of 26 heterodimeric [FeFe]-hydrogenases a phylogenetic tree based on the maximum parsimony algorithm was generated suggesting a monophyletic origin for the heterodimeric M2-subtype. The evolutionary history was inferred using the Maximum Parsimony method. Tree #1 out of 2 most parsimonious trees (length = 5088) is shown. The consistency index is 0.577126, the retention index is 0.632542, and the composite index is 0.372837 for all sites and 0.365056 for parsimony-informative sites. The percentage of replicate trees in which the associated taxa clustered together in the bootstrap test (50 replicates) are shown next to the branches.[9] The MP tree was obtained using the Subtree-Pruning-Regrafting (SPR) algorithm (pg. 126 in ref. [10]) with search level 1 in which the initial trees were obtained by the random addition of sequences (10 replicates). The analysis involved 48 amino acid sequences. There was a total of 641 positions in the final dataset. Evolutionary analyses were conducted in MEGA X.[11] The peach colored box marks those species that exhibit a heterodimeric M2-type [FeFe]-hydrogenase **(b)** A phylogenetic 16S RNA tree depicting the relationships among the major eubacterial phyla, illustrates that according to the distribution of species that provide heterodimeric M2-type [FeFe]-hydrogenases, the origin of this subtype may be located at the bottom of a branch that splits into Bacillota, Cyanobacteria, Nitrospira and Proteobacteria.

II.) Annotated Genomic and Polypeptide Sequences

For each of the 8 exemplary heterodimeric M2-type [FeFe]-hydrogenases the following is presented:

a.) The genomic section covering the successive genes for LSU and SSU,
b.) An annotated zoomed view on the short intergenic section between LSU and SSU
c.) The annotated polypeptide sequences of LSU and SSU along with the results of SignalP6 predictions for the presence of potential leader signals
d.) For *Desulfovibrio vulgaris subsp. vulgaris str. Hildenborough* (aka *Nitratidesulfovibrio vulgaris*) the polypeptide sequence and SignalP6 analyses of the likewise encoded monomeric [FeFe]-hydrogenase HydA-γ and the SignalP6 anlysis of maturases HydE, G and F have been added to section II.1c.

1.) ***Desulfovibrio vulgaris subsp. vulgaris str.*** **Hildenborough (δ-Proteobacteria)**

GenBank: AE017285.1
GenBank Graphics

a.)1832483-1836204 *Desulfovibrio vulgaris* subsp. vulgaris str. Hildenborough, complete genome

```
   1 atgagccgta ccgtcatgga gcgcatcgaa tatgagatgc acactccgga ccccaaggcc gatccggaca agctccactt cgtccagatc gacgaggcaa agtgcatagg ctgcgacacc tgttcgcagt actgccccac cgccgccatc ttcggcgaaa
     >>.....................................DdHydAB-LSU......................................>
      m  s  r   t  v  m   e  r  i  e   y  e  m   h  t  p   d  p  k  a   d  p  d   k  l  h   f  v  q  i   d  e  a   k  c  i   g  c  d  t   c  s  q   y  c  p   t  a  a  i   f  g  e

 161 tgggcgaacc gcactccatt ccccacatcg aggcgtgcat caactgcggc cagtgcctca cgcactgccc cgagaacgcc atctacgagg cacagtcgtg ggtgcctgaa gtcgagaaga agctgaagga cggcaaggtg aaatgcatcg ccatgcccgc
     >......................................DdHydAB-LSU......................................>
      m  g  e   p  h  s  i   p  h  i   e  a  c   i  n  c  g   q  c  l   t  h  c   p  e  n  a   i  y  e   a  q  s   w  v  p  e   v  e  k   k  l  k   d  g  k  v   k  c  i   a  m  p

 321 ccccgccgtg cgctatgcac tgggcgacgc cttcggcatg cccgtcggtt ccgtcaccac cggcaagatg ctcgcggccc tgcagaagct cggcttcgct cattgctggg acaccgagtt caccgctgac gtgaccatct gggaagaggg gtccgagttc
     >......................................DdHydAB-LSU......................................>
     a  p  a  v   r  y  a   l  g  d   a  f  g  m   p  v  g   s  v  t   t  g  k  m   l  a  a   l  q  k   l  g  f  a   h  c  w   d  t  e   f  t  a  d   v  t  i   w  e  e   g  s  e  f

 481 gtggaacgcc tcaccaagaa gagcgacatg ccgctgccgc agttcacctc gtgctgcccc ggctggcaga agtatgccga gacctactac cccgaactgc tgccgcactt ctccacgtgc aagtcgccca tcggcatgaa cggcgcactg gcgaagacct
     >......................................DdHydAB-LSU......................................>
      v  e  r   l  t  k   k  s  d  m   p  l  p   q  f  t   s  c  c  p   g  w  q   k  y  a   e  t  y  y   p  e  l   l  p  h   f  s  t  c   k  s  p   i  g  m   n  g  a  l   a  k  t

 641 acggcgcaga gcggatgaag tacgacccca agcaggtcta caccgtctcc atcatgccct gcatcgcaaa gaagtacgaa gggttgcgtc ccgaactgaa gtccagcggc atgcgcgaca tcgacgccac gctgaccacc cgtgagctgg cctacatgat
     >......................................DdHydAB-LSU......................................>
      y  g  a   e  r  m  k   y  d  p   k  q  v   y  t  v  s   i  m  p   c  i  a   k  k  y  e   g  l  r   p  e  l   k  s  s  g   m  r  d   i  d  a   t  l  t  t   r  e  l   a  y  m

 801 caagaaggcc ggtatcgact tcgcgaaact ccccgacggc aagcgtgaca gcctcatggg tgaatccacc ggcggtgcca ccatcttcgg cgtcaccggc ggcgtcatgg aagcggcact ccgcttcgcc tacgaagccg tcaccggcaa gaagcccgac
     >......................................DdHydAB-LSU......................................>
     i  k  k  a   g  i  d   f  a  k   l  p  d  g   k  r  d   s  l  m   g  e  s  t   g  g  a   t  i  f   g  v  t  g   g  v  m   e  a  a   l  r  f  a   y  e  a   v  t  g   k  k  p  d

 961 agctgggact tcaaggccgt gcgcggtctt gatggcatca aggaagccac cgtcaacgtc ggcggtaccg acgtcaaggt cgccgtggtg cacggggcca agcggttcaa gcaggtctgc gacgatgtga aggcgggcaa gtcgccctat cacttcatcg
     >>.....................................DdHydAB-LSU......................................>
      s  w  d   f  k  a   v  r  g  l   d  g  i   k  e  a   t  v  n  v   g  g  t   d  v  k   v  a  v  v   h  g  a   k  r  f   k  q  v  c   d  d  v   k  a  g   k  s  p  y   h  f  i

1121 aatacatggc ctgccccggc ggctgcgtct gtggcggcgg tcagcccgtc atgcccggcg tgctcgaagc catggaccgc accaccaccc gcctttacgc gggcctgaag aagcgcctcg ccatggcgag cgccaacaag gcataggagg aaacgccatg
     >..................................DdHydAB-LSU..........................................>>
      e  y  m   a  c  p  g   g  c  v   c  g  g   g  q  p  v   m  p  g   v  l  e   a  m  d  r   t  t  t   r  l  y   a  g  l  k   k  r  l   a  m  a   s  a  n  k   a  -
                                                                                                                                                        DdHydAB-SSU >>>
                                                                                                                                                                      m

1281 cagatagcca gcatcacccg gcgcggcttc ctcaaggtcg cctgcgtcac gacgggcgca gccctcatcg gcattcgcat gaccggaaag gccgttgccg ccgtcaagca gatcaaggac tacatgcttg accgcatcaa cggcgtctac ggggcggatg
     >......................................DdHydAB-SSU......................................>
       q  i  a   s  i  t   r  r  g  f   l  k  v   a  c  v   t  t  g  a   a  l  i   g  i  r   m  t  g  k   a  v  a   a  v  k   q  i  k  d   y  m  l   d  r  i   n  g  v  y   g  a  d

1441 ccaagttccc cgttcgcgcc tcgcaggaca acacgcaggt caaggctctc tacaagagct accttgagaa gcctctcggt cacaagtcgc acgacctgct gcacacgcac tggttcgaca agtccaaggg cgtcaaggaa ctcaccacgg caggcaagtt
     >......................................DdHydAB-SSU......................................>
      a  k  f   p  v  r  a   s  q  d   n  t  q   v  k  a  l   y  k  s   y  l  e   k  p  l  g   h  k  s   h  d  l   l  h  t  h   w  f  d   k  s  k   g  v  k  e   l  t  t   a  g  k

1601 gcccaacccg cgtgcttccg agttcgaagg tccgtacccc tacgaatagc gccagaacgt atacggaagg cataaacgca catccgtgat gccggaaccg cctgcggcga gggcctcttg gcctgacctc agggcgattc ctccggcaac gcagatgcat
     >..................DdHydAB-SSU..................>>
     l  p  n  p   r  a  s   e  f  e   g  p  y  p   y  e  -

1761 ctgccccacg atctcatgac acgaagacac ttaaagtaat acattactgt tttcgtgaag cctgtccctt gtggcgcggc gacagaaaag ccccccgacg cgcagcgcag gggggcggaa aacgtcagca cctgtccacg atcaatcaga gtcgccccgt
                                                                                                                                                   <<..DdHyd-Gamma....<
                                                                                                                                                    -  d  s   d  g  r

1921 acccgcctgc caagggtcat ctcatgccag atgtcgcgca tggtgtaggc gacttcgctc ttgcggtcgc cataccgggt gtgcagcaga cggtgagaca aattcgaaca gggctcgcca aggaatgatt cgtagacctt gccgatgagc ggattgttgt
     <......................................DdHyd-Gamma......................................<
      v  r  r   g  l  t  m   e  h  w   i  d  r   m  t  y  a   v  e  s   k  r  d   g  y  r  t   h  l  l   r  h  s   l  n  s  c   p  e  g   l  f  s   e  y  v  k   g  i  l   p  n  n

2081 gcgactgccg cagtgcgttt tccgcatcga gcgagaaaag ggcggcacgt cgcgcctgcg cgttggggtt gtaggcgcgc ttcgacctcg gctgaccgcc tccgtccatg catccacccg ggcatgccat gacctccacg aacacatggt cggccttccc
     <......................................DdHyd-Gamma......................................<
     h  s  q  r   l  a  n   e  a  d   l  s  f  l   a  a  r   r  a  q   a  n  p  n   y  a  r   k  s  r   p  q  g  g   g  d  m   c  g  g   p  c  a  m   v  e  v   f  v  h   d  a  k  g

2241 tgcaagaacc gcctcgacca tctgccgggc agccttgagc ccatgcacca cggcgacctt cacggaaccg ttaccctcac caagcgggac gacagcctca cgcacgttct cgtatccgcg cagggcatgc agttctactg ggcgagttc cttgccgttc
     <......................................DdHyd-Gamma......................................<
       a  l  v   a  e  v   m  q  r  a   a  k  l   g  h  v   v  a  v  k   v  s  g   n  g  e   g  l  p  v   v  a  e   r  v  n   e  y  g  r   l  a  h   l  e  v   p  a  l  e   k  g  n

2401 agcacatggt agaccgtacg cagtgccgcc tccattaccc cgcctgtcgt accgaagatg acagccgccc cggttgcccg ccccatcagg gggtcgtcgc agggcgaggg ttcgagtccg gcgaggtcta tgccctcacg ccggagaaga cgggcgaact
     <......................................DdHyd-Gamma......................................<
      l  v  h   y  v  t  r   l  a  a   e  m  v   g  g  t  t   g  f  i   v  a  a   g  t  a  r   g  m  l   p  d  d   c  p  s  p   e  l  g   a  l  d   i  g  e  r   r  l  l   r  a  f

2561 cacgcgtggt gagcactgcg tccacatccc ggacaccgtc gcgcctgaat tcgggccgtg cggcctcttc cttcttcgcc gtgcagggca tcaacgatac gacgcgcatc ctctccggtg cgacgttcat ggtgcgcgca agataggtct tggccaatgc
     <......................................DdHyd-Gamma......................................<
     e  r  t  t   l  v  a   d  v  d   r  v  g  d   r  r  f   e  p  r   a  a  e  e   k  k  a   t  c  p   m  l  s  v   v  r  m   r  e  p   a  v  n  m   t  r  a   l  y  t   k  a  l  a

2721 gccaaggcac tgctgaggcg agcgtgtggt cgagacatgc ggcaggatgt cggggaggtg cttctcggcg aaattcaccc agccggggca gcaggaggtg aagagcggca gcttcgcccc gccccgaaga cgttgcagga gttcggtgcc ctcctccatg
     <......................................DdHyd-Gamma......................................<
      g  l  c   q  q  p   s  r  t  t   s  v  h   p  l  i   d  p  l  h   k  e  a   f  n  v   w  g  p  c   c  s  t   f  l  p   l  k  a  g   g  r  l   r  q  l   l  e  t  g   e  e  m

2881 atgacgaggt cggctgcgaa gttggtatcg agtaccacgt ctgccccgag aaggcgcaag gccgtgggca cctgcccttc cacgcttgaa ccgggaggca gcccgaactc ctcaccgagg cccacccgca ccgccggggc gaactggaac acggtgacga
     <......................................DdHyd-Gamma......................................<
      i  v  l   d  a  a  f   n  t  d   l  v  v   d  a  g  l   l  r  l   a  t  p   v  q  g  e   v  s  s   g  p  p   l  g  f  e   e  g  l   g  v  r   v  a  p  a   f  q  f   v  t  v

3041 tttcggggtc gtagagatag tcgatgacac gctccacgtc gtcccgtcca gccaatgccc ccacagggca gaccaatgtg cactggccac agcccacaca gtcggacgca ctctggctac gattgtgccg cagcccgatt tcggtgccga tgccgtttcc
     <......................................DdHyd-Gamma......................................<
     i  e  p  d   y  l  y   d  i  v   r  e  v  d   d  r  g   a  l  a   g  v  p  c   v  l  t   c  q  g   c  g  v  c   d  s  a   s  q  s   r  n  h  r   l  g  i   e  t  g   i  g  n  g

3201 cgtcaccacg agggcatcga cgccctgtac gttgcggcac acggcgacac agcgcaggca cctgatgcac ttgcccatgt cgcgcacgac ggacggcgaa gagacatcac gggtgcggct gcgggcatag tccggaaaat ggtgacgcgt accggtaaga
     <......................................DdHyd-Gamma......................................<
       t  v  v   l  a  d   v  g  q  v   n  r  c   v  a  v   c  r  l  c   r  i  c   k  g  m   d  r  v  v   s  p  s   s  v  d   r  t  r  s   r  a  y   d  p  f   h  h  r  t   g  t  l

3361 cccacgaatt gtgccacatc ctgcaactcg cagtctccat gacgggcgca ggctgcgcag tcatggtcat ggtcggccag cagcagttcg acctgtagcc gctgcatcct gcgtacttca ggggtacgcg tgaagatgcg cattccctcc tcgacggggg
     <......................................DdHyd-Gamma......................................<
      g  v  f   q  a  v  d   q  l  e   c  d  g   h  r  a  c   a  a  c   d  h  d   h  d  a  l   l  l  e   v  q  l   r  q  m  r   r  v  e   p  t  r   t  f  i  r   m  g  e   e  v  p

3521 tcgtacagga ggtgacaatc tgcggcccgg cctccttgtc acgccatatc tcgaccagac agacccggca cgtcccgggt gcatgaccga tgtcggcaag ttcgcacaac gtgggaatga agtgcccgtt ctcgcgtgcg gcctcaagta tcgtcctgcc
     <......................................DdHyd-Gamma......................................<
     t  t  c  s   t  v  i   q  p  g   a  e  k  d   r  w  i   e  v  l   c  v  r  c   t  g  p   a  h  g   i  d  a  l   e  c  l   t  p  i   f  h  g  n   e  r  a   a  e  l   i  t  r  g

3681 cggttcacac cggacttcct tgccattgat gaacgcgttc at
     <...............DdHyd-Gamma...............<<
      p  e  c   r  v  e   k  g  n  i   f  a  n  m
```

b.)Segment between Stop codon of LSU and ATG of SSU including SD-sequence

```
aagcgcctcg ccatggcgag cgccaacaag gcataggagg aaacgccatg cagatagcca gcatcacccg
 k  r  l   a  m  a   s  a  n  k   a  -  e   e  t  p   c  r  -  p   a  s  p
  s  a  s   p  w  r   a  p  t   r  h  r  r   k  r  h   a  d  s   q  h  h  p
   a  p   r  h  g  e   r  q  q   g  i  g   g  n  a  m   q  i  a   s  i  t
        LSU                                              SSU
```

c.)Polypeptide sequences and SignalP-analysis for LSU and SSU

>*Dd*HydAB-LSU (GenBank:AAS96246.1)

```
MSRTVMERIEYEMHTPDPKADPDKLHFVQIDEAKCIGCDTCSQYCPTAAIFGEMGEPHSIPHIEACINCGQCLTHCPENAIYE
AQSWVPEVEKKLKDGKVKCIAMPAPAVRYALGDAFGMPVGSVTTGKMLAALQKLGFAHCWDTEFTADVTIWEEGSEFVERLTK
KSDMPLPQFTSCCPGWQKYAETYYPELLPHFSTCKSPIGMNGALAKTYGAERMKYDPKQVYTVSIMPCIAKKYEGLRPELKSS
GMRDIDATLTTRELAYMIKKAGIDFAKLPDGKRDSLMGESTGGATIFGVTGGVMEAALRFAYEAVTGKKPDSWDFKAVRGLDG
```

IKEATVNVGGTDVKVAVVHGAKRFKQVCDDVKAGKSPYHFIEYMACPGGCVCGGGQPVMPGVLEA↓MDRTTTRLYAGLKKRLA
MASANKA

Sequence
Prediction: Other

Protein type	Other	Signal Peptide (Sec/SPI)	Lipoprotein signal peptide (Sec/SPII)	TAT signal peptide (Tat/SPI)	TAT Lipoprotein signal peptide (Tat/SPII)	Pilin-like signal peptide (Sec/SPIII)
**Likelihood**	1	0	0	0	0	0

Download: PNG / EPS / Tabular

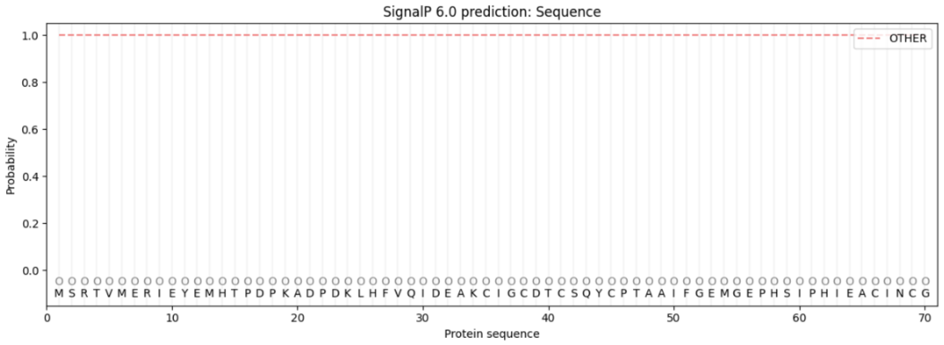

>*Dd*HydAB-SSU (GenBank:AAS96247.1)

MQIASI**TRR**G**FLK**VACVTTGAALIGIRMTGK**AVA**↓AVKQIKDYMLDRINGVYGADAKFPVRASQDNTQVKALYKSYLEKPLGH
KSHDLLHTHWFDKSKGVKELTTAGKLPNPRASEFEGPYPYE

Sequence
Prediction: TAT signal peptide (Tat/SPI)

Cleavage site between pos. 34 and 35.
Probability 0.882517

Protein type	Other	Signal Peptide (Sec/SPI)	Lipoprotein signal peptide (Sec/SPII)	TAT signal peptide (Tat/SPI)	TAT Lipoprotein signal peptide (Tat/SPII)	Pilin-like signal peptide (Sec/SPIII)
**Likelihood**	0	0	0	1	0	0

Download: PNG / EPS / Tabular

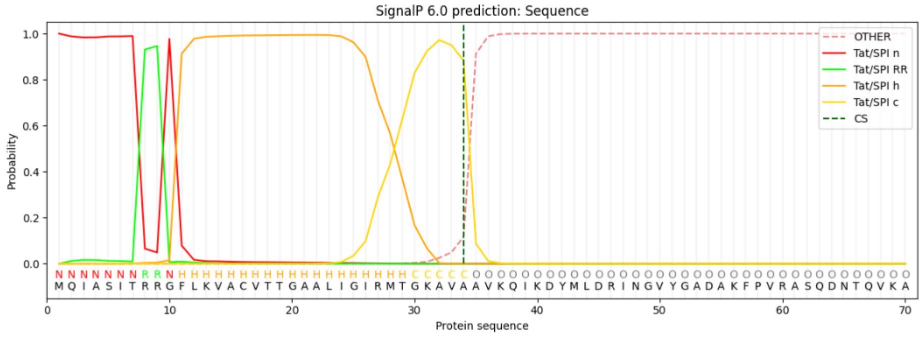

>*Dd*HydA-γ (GenBank: AAS96248.1)

MNAFINGKEVRCEPGRTILEAARENGHFIPTLCELADIGHAPGTCRVCLVEIWRDKEAGPQIVTSCTTPVEEGMRIFTRTPEV
RRMQRLQVELLLADHDHDCAACARHGDCELQDVAQFVGLTGTRHHFPDYARSRTRDVSSPSVVRDMGKCIRCLRCVAVCRNVQ
GVDALVVTGNGIGTEIGLRHNRSQSASDCVGCGQCTLVCPVGALAGRDDVERVIDYLYDPEIVTVFQFAPAVRVGLGEEFGLP
PGSSVEGQVPTALRLLGADVVLDTNFAADLVIMEEGTELLQRLRGGAKLPLFTSCCPGWVNFAEKHLPDILPHVSTTRSPQQC
LGALAKTYLARTMNVAPERMRVVSLMPCTAKKEEAARPEFRRDGVRDVDAVLTTREFARLLRREGIDLAGLEPSPCDDPLMGR
ATGAAVIFGTTGGVMEAALRTVYHVLNGKELAPVELHALRGYENVREAVVPLGEGNGSVKVAVVHGLKAARQMVEAVLAGKAD
HVFVEVMACPGGCMDGGGQPRSKRAYNPNAQARRAALFSLDAENALRQSHNNPLIGKVYESFLGEPCSNLSHRLLHTRYGDRK
SEVAYTMRDIWHEMTLGRRVRGDSD

DdHydA-γ

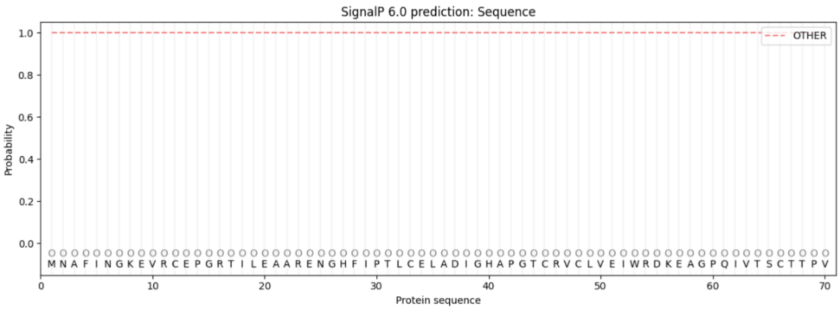

Maturases HydF,G and E

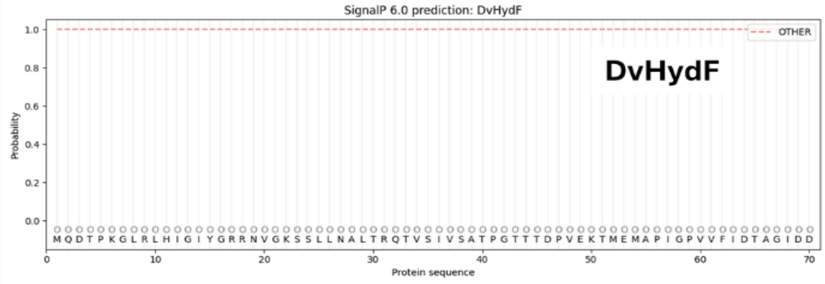

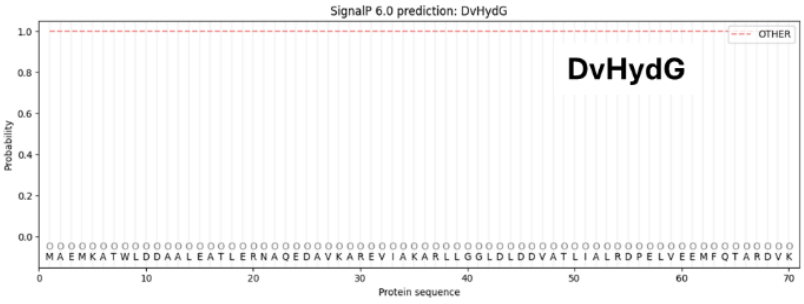

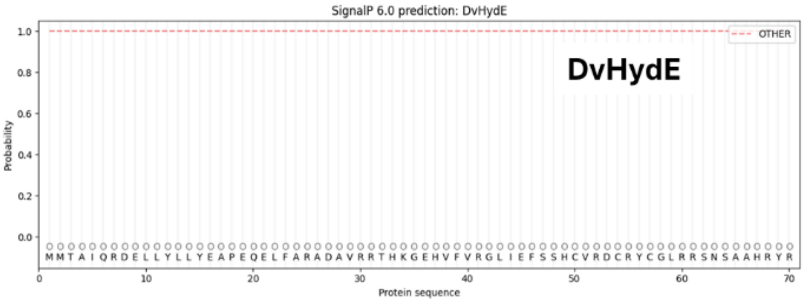

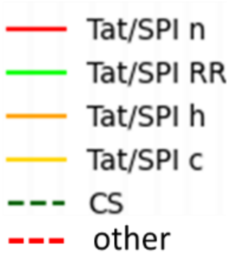

2.)*Desulfonauticus submarinus* strain DSM 15269 (δ-Proteobacteria)

GenBank: FNIN01000009.1

GenBank Graphics

a.) 47152-48857 *Desulfonauticus submarinus* strain DSM 15269 genome assembly, contig: Ga0070487-109, whole genome shotgun sequence

```
   1  atggctgctt gtagagttaa ggaaaagcct aaagttttgc cggtggattt atccgctata attccagaaa aggagggaac gatgaggaag atggaagacg ttatttattt aaataatgct ccacaccatg aagagccaga taatatttat tttgtccaag
      >>..................................................................DS-LSU..................................................................>
       m  a  a  c  r  v  k  e  k  p  k  v  l  p  v  d  l  s  a  i  i  p  e  k  e  g  t  m  r  k  m  e  d  v  i  y  l  n  n  a  p  h  h  e  e  p  d  n  i  y  f  v  q

 161  tagatcctac aaagtgtcag ggatgtggag aatgtgagga gcattgtgct acgggagcta ttcaatctat aaatgaagaa ggtattcacc aggtgttaag tccttcagct tgtatgaatt gtggacagtg tttagcgaac tgtccttatg ggctattta
      >...................................................................DS-LSU..................................................................>
      v  d  p  t  k  c  q  g  c  g  e  c  e  e  h  c  a  t  g  a  i  q  s  i  n  e  e  g  i  h  q  v  l  s  p  s  a  c  m  n  c  g  q  c  l  a  n  c  p  y  g  a  i

 321  tgagggagtt tcttttgtgg atgaagtttt tgagaagtta agggatcctg agactgtagt tgtttctatg cctgccccgg ctgttcgtta tgctcttgga gaatgttttg gatattctcc tggtacatat gtaggaggta aaatgcatgc tgctttaaga
      >...................................................................DS-LSU..................................................................>
      y  e  g  v  s  f  v  d  e  v  f  e  k  l  r  d  p  e  t  v  v  v  s  m  p  a  p  a  v  r  y  a  l  g  e  c  f  g  y  s  p  g  t  y  v  g  g  k  m  h  a  a  l  r

 481  aagcttggat ttgattatat ctgggataat gaatttgctg ccgacttaac tattatggag gaaggaaccg agttaatcga gcgaattaaa cacccaagta aggataagcc tctacctcag tttacctcat gttgtcctgg ttgggtaaag ttttgtgaat
      >...................................................................DS-LSU..................................................................>
       k  l  g  f  d  y  i  w  d  n  e  f  a  a  d  l  t  i  m  e  e  g  t  e  l  i  e  r  i  k  h  p  s  k  d  k  p  l  p  q  f  t  s  c  c  p  g  w  v  k  f  c  e

 641  ctttttatcc agatcttttg ccatatcttt ctacttgtaa atctcctatt ggtatgttga gtgcattagc taaaacctat ggtgcgcacc aaactcatac acctggtcaa aagatttata cagtatctat tatgccttgt attgctaaaa agtatgaagg
      >...................................................................DS-LSU..................................................................>
       s  f  y  p  d  l  l  p  y  l  s  t  c  k  s  p  i  g  m  l  s  a  l  a  k  t  y  g  a  h  q  t  h  t  p  g  q  k  i  y  t  v  s  i  m  p  c  i  a  k  k  y  e

 801  tcttagacca gaaatggcag atagtgggtt tagagatata gatgcaacta ttaatactag agaattggct tatatgatta aaaaggctgg tattgatttt agaagtttgc ctagtcaaga ccctgatcct gtacttggga tgtctacggg cgctgctact
      >...................................................................DS-LSU..................................................................>
      g  l  r  p  e  m  a  d  s  g  f  r  d  i  d  a  t  i  n  t  r  e  l  a  y  m  i  k  k  a  g  i  d  f  r  s  l  p  s  q  d  p  d  p  v  l  g  m  s  t  g  a  a  t

 961  atttttggga ctagtggagg agttatggag gctgctcttc gtttggctta tgaagtttta tctgggcaaa aacttactaa accggatata aaaattgttc gtacccatga aggaataaag gctgcggata ttaagatccc taagtttgga accattaaag
      >...................................................................DS-LSU..................................................................>
       i  f  g  t  s  g  g  v  m  e  a  a  l  r  l  a  y  e  v  l  s  g  q  k  l  t  k  p  d  i  k  i  v  r  t  h  e  g  i  k  a  a  d  i  k  i  p  k  f  g  t  i  k

1121  tagctgttgc tagtgggctt aaaaatgctg ctaaactttg tgaagaagtt agagcaggta aatctcctta ccattttata gaaattatgg cttgtcctgg tggttgtgta aatgggggag gacagccctt agacccaagt atccgtgagg aagcttcatt
      >...................................................................DS-LSU..................................................................>
       v  a  v  a  s  g  l  k  n  a  a  k  l  c  e  e  v  r  a  g  k  s  p  y  h  f  i  e  i  m  a  c  p  g  g  c  v  n  g  g  g  q  p  l  d  p  s  i  r  e  e  a  s

1281  atttaaaagg atgattgcaa agataaataa acggtataaa ggacgtaaac ccactattag ttaagaataa ggaggtatgt tatgaaacga atagtaacaa taagtagacg tagttttctt aaaacagcag gtattgcagt aggttatatg gttttaggtt
      >............................DS-LSU............................>>
      l  f  k  r  m  i  a  k  i  n  k  r  y  k  g  r  k  p  t  i  s  -
                                                                                                 >>...................................DS-SSU.....................................>
                                                                                                   m  k  r  i  v  t  i  s  r  r  s  f  l  k  t  a  g  i  a  v  g  y  m  v  l  g

1441  ttaatttaac taagcaggct gtggctgcaa ctatggagtt tattggcttg aggcagaagt ctgtttatga ggctgatagt aaggtatata aaattagaaa gtctcaagag aatcctatga ttaaaaagat ttatgacaag aaacatggtt ttttacatga
      >...................................................................DS-SSU..................................................................>
       f  n  l  t  k  q  a  v  a  a  t  m  e  f  i  g  l  r  q  k  s  v  y  e  a  d  s  k  v  y  k  i  r  k  s  q  e  n  p  m  i  k  k  i  y  d  k  k  h  g  f  l  h

1601  aggtccttgc ggacatatgt ctcatcgctt attgcatact aattattatg atcgtagtgc cagagtgaag gctttggaag aaaaaggagt taaattggcc atttag
      >................................................DS-SSU................................................>>
      e  g  p  c  g  h  m  s  h  r  l  l  h  t  n  y  y  d  r  s  a  r  v  k  a  l  e  e  k  g  v  k  l  a  i  -
```

b.)Segment between Stop codon of LSU and ATG of SSU including SD-sequence

```
acggtataaa ggacgtaaac ccactattag ttaagaataa ggaggtatgt tatgaaacga atagtaacaa taagtagacg
  t  v  -  r  t  -  t  h  y  -  l  r  i  r  r  y  v  m  k  r  i  v  t  i  s  r
 r  y  k  g  r  k  p  t  i  s  -  e  -  g  g  m  l  -  n  e  -  -  q  -  v  d
g  i  k  d  v  n  p  l  l  v  k  n  k  e  v  c  y  e  t  n  s  n  n  k  -  t
              LSU                                                SSU
```

c.) Polypeptide sequences and SignalP-analysis for LSU and SSU

>*DS*-LSU (GenBank: SDN85747.1)

MAACRVKEKPKVLPVDLSAIIPEKEGTMRKMEDVIYLNNAPHHEEPDNIYFVQVDPTKCQGCGECEEHCATGAIQSINEEGIH
QVLSPSACMNCGQCLANCPYGAIYEGVSFVDEVFEKLRDPETVVVSMPAPAVRYALGECFGYSPGTYVGGKMHAALRKLGFDY
IWDNEFAADLTIMEEGTELIERIKHPSKDKPLPQFTSCCPGWVKFCESFYPDLLPYLSTCKSPIGMLSALAKTYGAHQTHTPG
QKIYTVSIMPCIAKKYEGLRPEMADSGFRDIDATINTRELAYMIKKAGIDFRSLPSQDPDPVLGMSTGAATIFGTSGGVMEAA
LRLAYEVLSGQKLTKPDIKIVRTHEGIKAADIKIPKFGTIKVAVASGLKNAAKLCEEVRAGKSPYHFIEIMACPGGCVNGGGQ
PLDPSIREEASLFKRMIAKINKRYKGRKPTIS

	Protein type	Other	Signal Peptide (Sec/SPI)	Lipoprotein signal peptide (Sec/SPII)	TAT signal peptide (Tat/SPI)	TAT Lipoprotein signal peptide (Tat/SPII)	Pilin-like signal peptide (Sec/SPIII)
**Sequence**<br>**Prediction:** Other	Likelihood	1	0	0	0	0	0

Download: PNG / EPS / Tabular

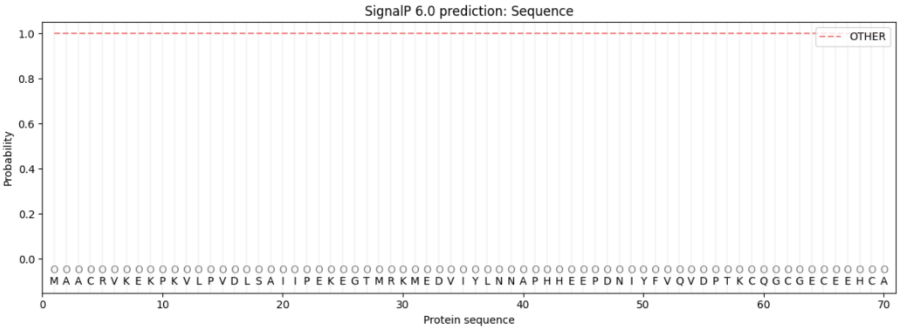

>*DS*-SSU (GenBank: SDN85790.1)

MKRIVTI**SRR**S**FLK**TAGIAVGYMVLGFNLTKQ**AVA**ATMEFIGLRQKSVYEADSKVYKIRKSQENPMIKKIYDKKHGFLHEGPC
GHMSHRLLHTNYYDRSARVKALEEKGVKLAI

	Protein type	Other	Signal Peptide (Sec/SPI)	Lipoprotein signal peptide (Sec/SPII)	TAT signal peptide (Tat/SPI)	TAT Lipoprotein signal peptide (Tat/SPII)	Pilin-like signal peptide (Sec/SPIII)
**Sequence**<br>**Prediction:** TAT signal peptide (Tat/SPI)<br>Cleavage site between pos. 35 and 36. Probability 0.843821	Likelihood	0	0	0	0.9837	0.0162	0

Download: PNG / EPS / Tabular

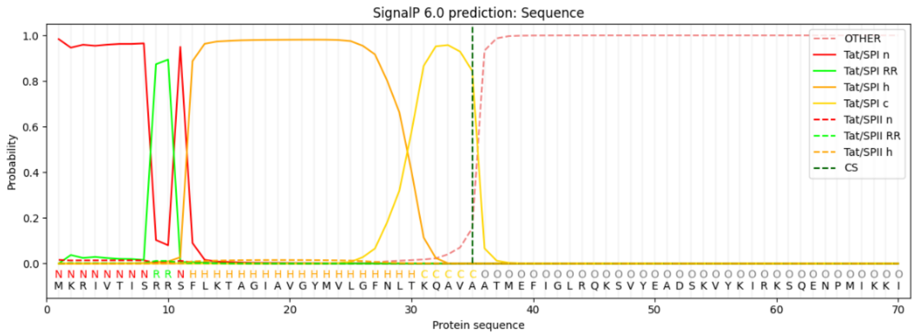

3.) *Campylobacter rectus* strain ATCC 33238 (ε–Proteobacteria)

GenBank:CP012543.1
GenBank Graphics

a.)1499101-1500812 *Campylobacter rectus* strain ATCC 33238,complete genome

```
   1 atgcttagtg tctataagat caaaactttc cctccggcgc ctaattcaag gggcggggaa gctgattttg aaggtactta tagaaaaggc gaattacgcg gtattataag aatagatcaa gatagctgtg tcggatgcga tacctgtcgc tcctttttgcc
     >>.....................................................................CR-LSU...................................................................>
      m  l  s  v  y  k  I  k  t  f  p  p  a  p  n  s  r  g  g  e  a  d  f  e  g  t  y  r  k  g  e  l  r  g  I  I  r  I  d  q  d  s  c  v  g  c  d  t  c  r  s  f  c

 161 ctacagatgc catagacggc tctctcggag ttgctcacaa aatagatcaa aatttatgcg tagcctgtgg acagtgcctc ataaactgcc cgtttgccgt catagagcaa atgagctttg tcgatgaggt tatgcaaaag cttgacgacg aaaagacctt
     >......................................................................CR-LSU...................................................................>
      p  t  d  a  I  d  g  s  l  g  v  a  h  k  I  d  q  n  l  c  v  a  c  g  q  c  l  I  n  c  p  f  a  v  I  e  q  m  s  f  v  d  e  v  m  q  k  l  d  d  e  k  t

 321 tgtcgtagct caccccttcgc ctgcggttag ggtttctttg gctgaagaat tcggtggaaa acccggagaa ctaaccgtaa ataagatgta taacgccttc gaaaaagccg gctttaatat gtacgacgta aattttgcgg ccgatcagac gatattggag
     >......................................................................CR-LSU...................................................................>
     f  v  v  a  h  p  s  p  a  v  r  v  s  l  a  e  e  f  g  g  k  p  g  e  l  t  v  n  k  m  y  n  a  f  e  k  a  g  f  n  m  y  d  v  n  f  a  a  d  q  t  I  l  e

 481 gaaggaaccg agctaattaa aaagataaaa tattggctac tcggcgaacg tagtcatgat ttagagcacg tttcgcacca tcctttcccg cattttacga gctgctgtcc ggcatgggta agaaatgccg aaattttcca tccggagctg atacctcata
     >......................................................................CR-LSU...................................................................>
      e  g  t  e  l  I  k  k  I  k  y  w  l  l  g  e  r  s  h  d  l  e  h  v  s  h  h  p  f  p  h  f  t  s  c  c  p  a  w  v  r  n  a  e  I  f  h  p  e  l  I  p  h

 641 tctcgggcgc aaaatctccg atacaaatgg gcgggcctct tgctaaaacc tgggcggcta aattcgtttg ggataaagat ccgcgcgata tttacgtagc tacggttact ccctgtactg caaaaatcta cgaggcaagc agaccggaat ttaactccgc
     >......................................................................CR-LSU...................................................................>
      I  s  g  a  k  s  p  I  q  m  g  g  p  l  a  k  t  w  a  a  k  f  v  w  d  k  d  p  r  d  I  y  v  a  t  v  t  p  c  t  a  k  I  y  e  a  s  r  p  e  f  n  s

 801 atacgagtat ctgaaagaaa gaggtgaaat acccgccgat acaaaaagct ttcccgatat agatgcaacg cttacggctc gcgatatagc tgaaattttg cgtaaaaaag gcataaatcc tcttgagatg tcggatgaat accctgaaaa aacaatgaat
     >......................................................................CR-LSU...................................................................>
     a  y  e  y  l  k  e  r  g  e  I  p  a  d  t  k  s  f  p  d  I  d  a  t  l  t  a  r  d  I  a  e  I  l  r  k  k  g  I  n  p  l  e  m  s  d  e  y  p  e  k  t  m  n

 961 gtttataccg gcggaggtac tatattcgga aatagcggcg gcgttatgga ggcggcgctt agaacggctt attttctact ctcgggacaa gagctaaaag atcccgacct aactcccgta agaggctacg ataaggatct aacggaagcc gtaataccga
     >......................................................................CR-LSU...................................................................>
      v  y  t  g  g  g  t  I  f  g  n  s  g  g  v  m  e  a  a  l  r  t  a  y  f  l  l  s  g  q  e  l  k  d  p  d  l  t  p  v  r  g  y  d  k  d  l  t  e  a  v  I  p

1121 tccctctaaa agactacgac ggcaagacgc tcgaactaaa agtagccgta gtaaacggtg cttcaagaaa tttaaacacc atcttaaaac atattactaa agatagcaac agatatcact tcatagaggt gatgaactgc ccgggcggat gcgtaaacgg
```

```
      >..........................................................................CR-LSU...........................................................................>
       I  p  l   k  d  y  d   g  k  t   l  e  l   k  v  a  v   v  n  g   a  s  r   n  l  n  t   I  l  k   h  I  t   k  d  s  n   r  y  h   f  I  e   v  m  n  c   p  g  g   c  v  n

1281  cggcggacag cccgtgcatg ctatgggaac atcgtggctt cattcgctac tgccgcttcc gttaaaagct taaaaggata agaaatgaga tatcaattta tagaaaaacc tgtaggaaaa atttttcaa gaagggattt tttaaaggtt agcggcgttt
      >................................CR-LSU.................................>>
      g  g  g  q   p  v  h   a  m  g   t  s  w  l   h  s  l   l  p  l   p  l  k  a   -
                                                                                          >>..................................CR-SSU.....................................>
                                                                                            m  r   y  q  f   I  e  k   p  v  g  k   I  f  s   r  r  d   f  l  k  v   s  g  v

1441  tgacctcgat tattgcgatt agcggctatg cgataacgga tatcatcaaa agacgtaaat cttatatcgc gatgcgccaa gagggcttat acaaagacga taagcgctgt caagacaaaa aactaatcgg ctcccaccaa aaccctagtt gcgctcaatg
      >..........................................................................CR-SSU...........................................................................>
       l  t  s   I  I  a  I   s  g  y   a  I  t   d  I  I  k   r  r  k   s  y  I   a  m  r  q   e  g  l   y  k  d   d  k  r  c   q  d  k   k  l  I   g  s  h  q   n  p  s   c  a  q

1601  ttatgccgat ttaaatacgg agcctatggg cgaagtagcg gaaaaactgc ttcatacaag cgcctacttc gatcgaaaaa atttgatctt aaaaggagct agccatgcat ga
      >.........................................................CR-SSU.......................................................>>
      c  y  a  d   l  n  t   e  p  m   g  e  v  a   e  k  l   l  h  t   s  a  y  f   d  r  k   n  l  I   l  k  g  a   s  h  a   -
```

b.)Segment between Stop codon of LSU and ATG of SSU including SD-sequence

```
tgccgcttcc gttaaaagct  taaaaggata agaaatgaga tatcaattta tagaaaaacc tgtaggaaaa
 l  p  l   p  l  k  a   -  k  d   k  k  -   d  I  n  l   -  k  n   l  -  e
 c  r  f   r  -  k   l  k  r  I   r  n  e   I  s  I   y  r  k  t   c  r  k
t  a  a  s   v  k  s   l  k  g   -  e  m  r   y  q  f   I  e  k   p  v  g  k
      LSU                                                SSU
```

c.)Polypeptide sequences and SignalP-analysis for LSU and SSU

>*CR*-LSU

MLSVYKIKTFPPAPNSRGGEADFEGTYRKGELRGIIRIDQDSCVGCDTCRSFCPTDAIDGSLGVAHKIDQNLCVACGQCLINC
PFAVIEQMSFVDEVMQKLDDEKTFVVAHPSPAVRVSLAEEFGGKPGELTVNKMYNAFEKAGFNMYDVNFAADQTILEEGTELI
KKIKYWLLGERSHDLEHVSHHPFPHFTSCCPAWVRNAEIFHPELIPHISGAKSPIQMGGPLAKTWAAKFVWDKDPRDIYVATV
TPCTAKIYEASRPEFNSAYEYLKERGEIPADTKSFPDIDATLTARDIAEILRKKGINPLEMSDEYPEKTMNVYTGGGTIFGNS
GGVMEAALRTAYFLLSGQELKDPDLTPVRGYDKDLTEAVIPIPLKDYDGKTLELKVAVVNGASRNLNTILKHITKDSNRYHFI
EVMNCPGGCVNGGGQPVHAMGTSWLHSLLPLPLKA

Sequence
Prediction: Other

Protein type	Other	Signal Peptide (Sec/SPI)	Lipoprotein signal peptide (Sec/SPII)	TAT signal peptide (Tat/SPI)	TAT Lipoprotein signal peptide (Tat/SPII)	Pilin-like signal peptide (Sec/SPIII)
Likelihood	1	0	0	0	0	0

Download: PNG / EPS / Tabular

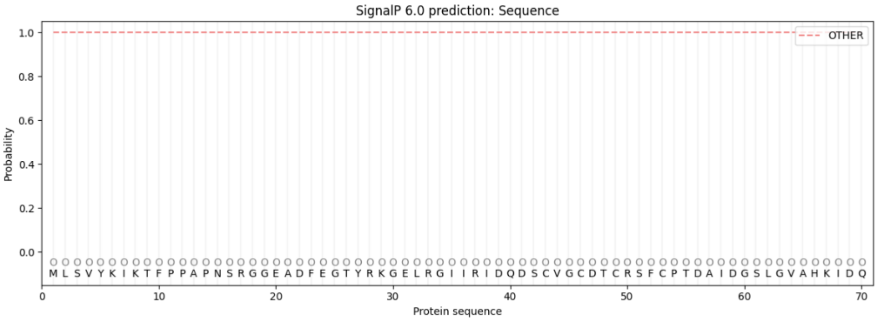

>*CR*-SSU

MRYQFIEKPVGKIF**SRR**D**FLK**VSGVLTSIIAISGY**AIT**DIIKRRKSYIAMRQEGLYKDDKRCQDKKLIGSHQNPSCAQCYADL
NTEPMGEVAEKLLHTSAYFDRKNLILKGASHA

Sequence
Prediction: TAT signal peptide (Tat/SPI)

Cleavage site between pos. 38 and 39. Probability 0.299688

Protein type	Other	Signal Peptide (Sec/SPI)	Lipoprotein signal peptide (Sec/SPII)	TAT signal peptide (Tat/SPI)	TAT Lipoprotein signal peptide (Tat/SPII)	Pilin-like signal peptide (Sec/SPIII)
**Likelihood**	0.1619	0.0084	0.0026	0.6716	0.1544	0.0011

Download: PNG / EPS / Tabular

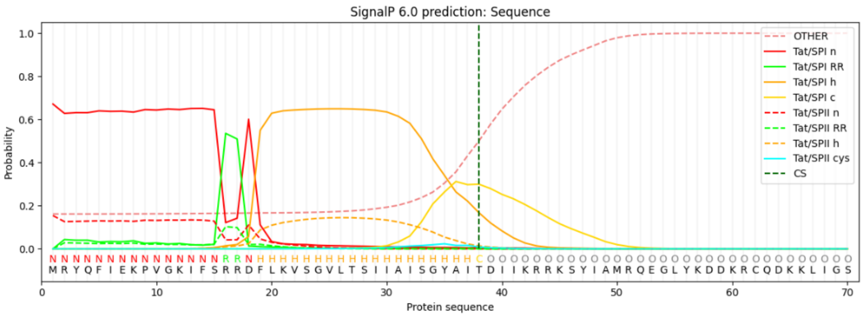

4.)*Sutterella wadsworthensis* (β–Proteobacteria)

GenBank: ATCF01000038.1
GenBank Graphics

a.)5259-7004 *Sutterella wadsworthensis* HGA0223 acAqX-supercont1.3.C38, whole genome shotgun sequence

```
   1 atgttcaagg atacgatgac gatccacacc ttcgggccgg gcgaaaacgc atacggtcag aacggtggcg cctacgaagg caatctgcgc aagggtgagc tgcgcgggat tattcacatc aacaaagatc actgcgttgg gtgcgacacc tgccgcaagt
     >>...............................................................................SW-LSU...............................................................................>
       m  f  k   d  t  m   t  I  h  t   f  g  p   g  e  n   a  y  g  q   n  g  g   a  y  e   g  n  l  r   k  g  e   l  r  g   I  I  h  I   n  k  d   h  c  v   g  c  d  t   c  r  k

 161 tctgcccgac agatgccatt aagggtggtc tcggcgccaa acacgagagc attgacgatg cgtgccttta ttgcggccag tgccttgtgg cctgcccgtt caatgccatt gagcagatga gcttcgttga tgaggtcgaa cgcgttcttg acgctaagga
     >................................................................................SW-LSU...............................................................................>
      f  c  p   t  d  a  I   k  g  g   l  g  a   k  h  e  s   I  d  d   a  c  l   y  c  g  q   c  l  v   a  c  p   f  n  a  I   e  q  m   s  f  v   d  e  v  e   r  v  l   d  a  k

 321 ccgcatcgtg gttgcccagc cgtcgccagc cgtgcgcgta tcgatctgcg aagaattcgg tggggaaccc ggtgaactct cgaccgagca aatggtcaac gctctggaag ccctcggctg cgtcacgtac gactgcaaca gctctgcgga ccagacgatt
     >................................................................................SW-LSU...............................................................................>
     d  r  I  v   v  a  q   p  s  p   a  v  r  v   s  I  c   e  e  f   g  g  e  p   g  e  l   s  t  e   q  m  v  n   a  l  e   a  l  g   c  v  t  y   d  c  n   s  s  a   d  q  t  i

 481 atcgaagaag gcaccgaatt cgtgaagaag gtgcaatact gggtactcgg cgaacgtggg cctgaagtcg acgagcaggg taaacacccg ttcccgcatt tcacatcttg ctgcccgggc tgggtgaagt acgccgaaac ttatgcggcc gacatgctcc
     >................................................................................SW-LSU...............................................................................>
       I  e  e   g  t  e   f  v  k  k   v  q  y   w  v  l   g  e  r  g   p  e  v   d  e  q   g  k  h  p   f  p  h   f  t  s   c  c  p  g   w  v  k   y  a  e   t  y  a  a   d  m  l

 641 cccacctttc caccgcgaaa tcccctctgc agatgggtgg cacgctcgcg aagacgtggg cagccaagca cattctcaag tgcgatcccc gcaaggtcta ctttgtttcg atgacgccct gcacggccaa gatcttcgag gcttcccgtc ccgaaatgaa
     >................................................................................SW-LSU...............................................................................>
      p  h  l   s  t  a  k   s  p  l   q  m  g   g  t  l  a   k  t  w   a  a  k   h  I  l  k   c  d  p   r  k  v   y  f  v  s   m  t  p   c  t  a   k  I  f  e   a  s  r   p  e  m

 801 taccgcctgg cgttggctca ttgagcacaa ggaaatcccg gctaacacgc cttccttcca agatattgac gcctctctca cggcgcgcga ccttgccgaa cttttccgtc gcaagggcat caacccgctc cttatgccga agacccgcaa gcgcgacagc
     >................................................................................SW-LSU...............................................................................>
     n  t  a  w   r  w  l   I  e  h   k  e  I  p   a  n  t   p  s  f   q  d  I  d   a  s  l   t  a  r   d  l  a  e   l  f  r   r  k  g   I  n  p  l   l  m  p   k  t  r   k  r  d  s

 961 gaaacacatc cgcttgaggt ctattctggc gctggcacga tcttcggctg ctccggcggc gtgatggaag cggcgctgcg caccgcctac tttgccctcg ctggcaagga actcgataac aaggacattg aggtcgttcg cggccacaac aacgcgatta
     >................................................................................SW-LSU...............................................................................>
       e  t  h   p  l  e   v  y  s  g   a  g  t   I  f  g   c  s  g  g   v  m  e   a  a  l   r  t  a  y   f  a  l   a  g  k   e  l  d  n   k  d  I   e  v  v   r  g  h  n   n  a  i

1121 ttgaagccac aatccccgtt cccgttaagg aactcggcgg caagattttt gaagtccgcg tttgtgtcgt taacggctgc aaccagggta tcgctgaagt gctccaccgt gttcgcgtcg ataagaaccg ctaccacttc atcgaagtca tgaactgtcc
     >................................................................................SW-LSU...............................................................................>
      I  e  a   t  I  p  v   p  v  k   e  l  g   g  k  I  f   e  v  r   v  c  v   v  n  g  c   n  q  g   I  a  e   v  l  h  r   v  r  v   d  k  n   r  y  h  f   I  e  v   m  n  c

1281 tggcggctgc gtcaacggcg gcggccagcc ggttcagcca gttggtacat cttggctcaa gcccaccaca ccgcttcccc tgcgcgtcta atttttagag gttctcaaaa aaatgttctc tcaaaactat tcctacgcgg aacgtcctgc ggccttgatt
     >.........................................SW-LSU..........................................>>
     p  g  g  c   v  n  g   g  g  q   p  v  q  p   v  g  t   s  w  l   k  p  t  t   p  l  p   l  r  v   -
                                                                                                                                >>...................SW-SSU.....................>
                                                                                                                                  m  f   s  q  n  y   s  y  a   e  r  p   a  a  l  i

1441 ctcggccgtc gaggcttcct gaaggtgagc ggcctctgca tcggcgccgc agtcgtttgc ggctgggcaa ttggcgacat ggtgtcgcgc cgcagttcaa tcatccttgc tcgtcaagcc ggtctctatc aagatgacaa gctttgtcag gcaatgggac
     >................................................................................SW-SSU...............................................................................>
       l  g  r   r  g  f   l  k  v  s   g  l  c   I  g  a   a  v  v  c   g  w  a   I  g  d   m  v  s  r   r  s  s   I  I  l   a  r  q  a   g  l  y   q  d  d   k  l  c  q   a  m  g

1601 ttgcctcttc gcacaacaat ccggttgtga tgtctgtcta taagaccatg aaggccaagc ccgtggatca tacgatgcac gagctcctgc acacccattt ctactcccgt tccatgctag ccatgacgga ggctgctcat gtctga
     >........................................................................SW-SSU.........................................................................>>
      l  a  s   s  h  n  n   p  v  v   m  s  v   y  k  t  m   k  a  k   p  v  d   h  t  m  h   e  l  l   h  t  h   f  y  s  r   s  m  l   a  m  t   e  a  a  h   v  -
```

b.)Segment between Stop codon of LSU and ATG of SSU including SD-sequence

```
gcccaccaca ccgcttcccc tgcgcgtcta atttttagag gttctcaaaa aaatgttctc tcaaaactat tcctacgcgg
   a   h   h   t   a   s   p   a   r   l   I   f   r   g   s   q   k   n   v   l   s   k   l   f   l   r
    p   t   t   p   l   p   l   r   v   -   f   l   e   v   l   k   k  m   f   s   q   n   y   s   y   a
     p   p   h   r   f   p   c   a   s   n   f   -   r   f   s   k   k   c   s   l   k   t   I   p   t   r
           LSU                                                                        SSU
```

c.)Polypeptide sequences and SignalP-analysis for LSU and SSU

>*SW*-LSU (GenBank: EPD97437.1)

MFKDTMTIHTFGPGENAYGQNGGAYEGNLRKGELRGIIHINKDHCVGCDTCRKFCPTDAIKGGLGAKHESIDDACLYCGQCLV
ACPFNAIEQMSFVDEVERVLDAKDRIVVAQPSPAVRVSICEEFGGEPGELSTEQMVNALEALGCVTYDCNSSADQTIIEEGTE
FVKKVQYWVLGERGPEVDEQGKHPFPHFTSCCPGWVKYAETYAADMLPHLSTAKSPLQMGGTLAKTWAAKHILKCDPRKVYFV
SMTPCTAKIFEASRPEMNTAWRWLIEHKEIPANTPSFQDIDASLTARDLAELFRRKGINPLLMPKTRKRDSETHPLEVYSGAG
TIFGCSGGVMEAALRTAYFALAGKELDNKDIEVVRGHNNAIIEATIPVPVKELGGKIFEVRVCVVNGCNQGIAEVLHRVRVDK
NRYHFIEVMNCPGGCVNGGGQPVQPVGTSWLKPTTPLPLRV

Sequence
Prediction: Other

Protein type	Other	Signal Peptide (Sec/SPI)	Lipoprotein signal peptide (Sec/SPII)	TAT signal peptide (Tat/SPI)	TAT Lipoprotein signal peptide (Tat/SPII)	Pilin-like signal peptide (Sec/SPIII)
**Likelihood**	1	0	0	0	0	0

Download: PNG / EPS / Tabular

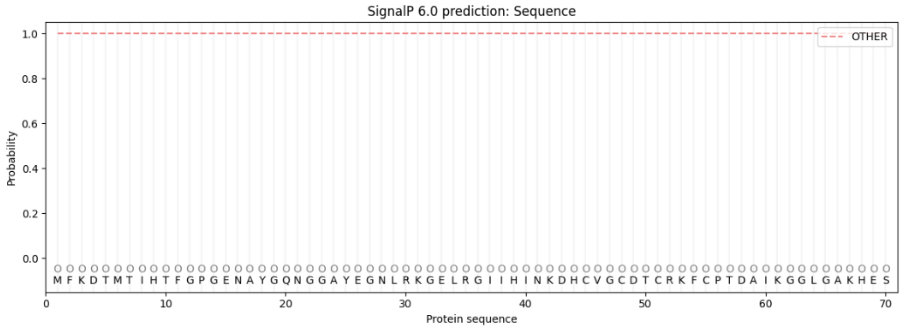

>*SW*-SSU(GenBank: EPD97438.1)

MFSQNYSYAERPAALILG**RR**G**FLK**VSGLCIGAAVVCGW**AIG**DMVSRRSSIILARQAGLYQDDKLCQAMGLASSHNNPVVMSVY
KTMKAKPVDHTMHELLHTHFYSRSMLAMTEAAHV

Sequence
Prediction: TAT signal peptide (Tat/SPI)

Cleavage site between pos. 41 and 42.
Probability 0.550727

Protein type	Other	Signal Peptide (Sec/SPI)	Lipoprotein signal peptide (Sec/SPII)	TAT signal peptide (Tat/SPI)	TAT Lipoprotein signal peptide (Tat/SPII)	Pilin-like signal peptide (Sec/SPIII)
**Likelihood**	0.0058	0.0001	0.0001	0.8534	0.1406	0

Download: PNG / EPS / Tabular

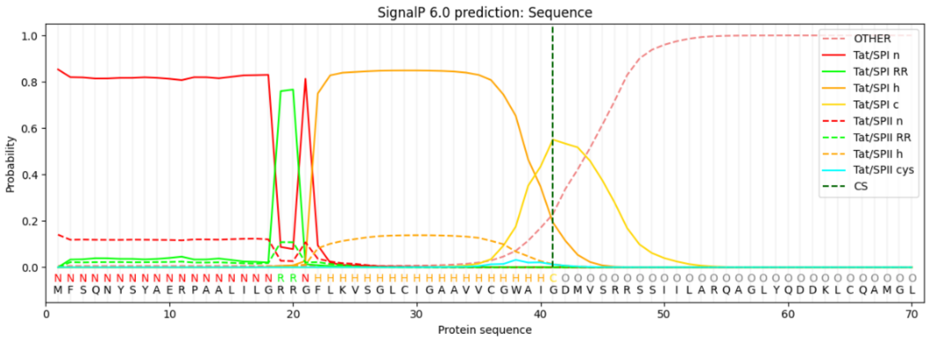

5.) *Thermodesulfovibrio yellowstonii* DSM 11347 (Nitrospira)

GenBank: CP001147.1

GenBank Graphics

a.)1657329-1655609 *Thermodesulfovibrio yellowstonii* DSM 11347, complete genome

```
      atggtaagaa aagtaaacac attcaagggt aacggtgcag tgaagtcaca gacaggaact tacagagcag gagagctaag aggaataatc aaaatcaacg agggtaattg tgttggctgt catacctgta gcagtgtttg tcctgcagga gcagtaaaag
      >>..........................................TY-LSU..........................................>
       m  v  r  k  v  n  t  f  k  g  n  g  a  v  k  s  q  t  g  t  y  r  a  g  e  l  r  g  I  I  k  I  n  e  g  n  c  v  g  c  h  t  c  s  s  v  c  p  a  g  a  v  k
 161  gctccttcgg agacaaacac agcattgacc ttgacaaatg tatcaactgt ggtcagtgcc tgcttaactg tccttttggt gcagttgaac agatgagctt tgttgatgtg gtaatggcaa aacttaaaga caaaaaaact aaggttgttg caattattgc
      >...........................................TY-LSU..........................................>
       g  s  f  g  d  k  h  s  I  d  l  d  k  c  I  n  c  g  q  c  l  l  n  c  p  f  g  a  v  e  q  m  s  f  v  d  v  v  m  a  k  l  k  d  k  k  t  k  v  v  a  I  i
 321  tccggctgtg agagttgcca ttggagaaga gtttggtgct gagccaggca cacttacagt gggaagacta tgggctgctt tggaaaaagc aggtttttta atctatgaca acaactttgc cgctgaccag acaattcttg aggaaggcac agagttactt
      >...........................................TY-LSU..........................................>
      a  p  a  v  r  v  a  I  g  e  e  f  g  a  e  p  g  t  l  t  v  g  r  l  w  a  a  l  e  k  a  g  f  l  I  y  d  n  n  f  a  a  d  q  t  I  l  e  e  g  t  e  l  l
 481  gcaaaagtag cagcccatgc agggcttaaa cagttacctg ttgaactctg gggaaaaaaa ataactcttg atatcaaaga gttctctcat catcccctac cacagtttac ttcctgctgt cccgcatggg taagatatgt agaagttttc tatccaaaac
      >...........................................TY-LSU..........................................>
       a  k  v  a  a  h  a  g  l  k  q  l  p  v  e  l  w  g  k  k  I  t  l  d  I  k  e  f  s  h  h  p  l  p  q  f  t  s  c  c  p  a  w  v  r  y  v  e  v  f  y  p  k
 641  ttattcccta cctttcttca gccaaatcac cccagcagat ggcaggagca acagcaaaga cctacggtgc aaagctttgg ggagcgaaac ctgagaatat tttcaccgtt ggagtaatgc cctgtacagc aaaaatattt gaagcctccc gtcctgaatt
      >...........................................TY-LSU..........................................>
       l  I  p  y  l  s  s  a  k  s  p  q  q  m  a  g  a  t  a  k  t  y  g  a  k  l  w  g  a  k  p  e  n  I  f  t  v  g  v  m  p  c  t  a  k  I  f  e  a  s  r  p  e
 801  tgattctgca ggaaagtatt taaagaagtc aggtatgaga gatgtggatg cagttctaac aacaagagac cttgcagaac ttctaaagcg tatgaacata gacccaatga aaatgtctga ggatgcaagc agaaaacctg agatgttcaa attttactca
      >...........................................TY-LSU..........................................>
      f  d  s  a  g  k  y  l  k  k  s  g  m  r  d  v  d  a  v  l  t  t  r  d  l  a  e  l  l  k  r  m  n  I  d  p  m  k  m  s  e  d  a  s  r  k  p  e  m  f  k  f  y  s
 961  ggtggtgcta ctatatttgg cacaagcggt ggagttatgg aagcagctgt aagatttgcc tttcatgttc tctccgggca ggagccacag gcaatgagtc ctaaatggga ttttgaaggt gtgagaggtt ttacaaaacc tgttgtctca gcaactattc
      >...........................................TY-LSU..........................................>
       g  g  a  t  I  f  g  t  s  g  g  v  m  e  a  a  v  r  f  a  f  h  v  l  s  g  q  e  p  q  a  m  s  p  k  w  d  f  e  g  v  r  g  f  t  k  p  v  v  s  a  t  i
1121  ctgttcctct aagagaagaa tatcagaagg ctttcggaac aaaagaactt caggtcaagg tctgtgtagt aaatggcata ggaacagatg cagcacatct gaagccaatt gttgaagaag ttttagcagg taaaagcccc tatcacttca tagaggtaat
      >...........................................TY-LSU..........................................>
       p  v  p  l  r  e  e  y  q  k  a  f  g  t  k  e  l  q  v  k  v  c  v  v  n  g  I  g  t  d  a  a  h  l  k  p  I  v  e  e  v  l  a  g  k  s  p  y  h  f  I  e  v
1281  gaactgtcct ggtggctgta ttaacggagg tggacagcca gttcatccaa ttgaactttc tctcttagac cagctttttt attctcttgt aaaaaacttt gaaggaggca aactatgaaa atcagaaagc ttacaagaag gtcttttcta aaattagcag
      >............................TY-LSU.............................>>
      m  n  c  p  g  g  c  I  n  g  g  g  q  p  v  h  p  I  e  l  s  l  l  d  q  l  f  y  s  l  v  k  n  f  e  g  g  k  l  -
                                                                                                   >>............TY-SSU............>
                                                                                                     m  k  I  r  k  l  t  r  r  s  f  l  k  l  a
1441  gtgctggaat tatcagcctt tcctttacaa agccatcttt tgctggtgat acagcagaga aaaacttttt tgagacaaag gtcggaaaag aaagacttaa actcataaaa gcaagacagt ccgggcagta taaggatgac gttatatcaa gagagaaatt
      >...........................................TY-SSU..........................................>
       g  a  g  I  I  s  l  s  f  t  k  p  s  f  a  g  d  t  a  e  k  n  f  f  e  t  k  v  g  k  e  r  l  k  l  I  k  a  r  q  s  g  q  y  k  d  d  v  I  s  r  e  k
1601  taaaatggcc gcatcccatg aaaacccgat gataaaaagg ttttactctg agtttgctca tcatccttta agtgaagtaa gtgaagcatt gttgcatacg cattacaagg caagggtata g
      >...............................TY-SSU.................................>>
      f  k  m  a  a  s  h  e  n  p  m  I  k  r  f  y  s  e  f  a  h  h  p  l  s  e  v  s  e  a  l  l  h  t  h  y  k  a  r  v  -
```

b.) Segment between Stop codon of LSU and ATG of SSU including SD-sequence

```
tcttgtaaaa aactttgaag gaggcaaact atgaaaatca gaaagcttac aagaaggtct tttctaaaat
   s  c  k  k  l  -  r  r  q  t  m  k  I  r  k  l  t  r  r  s  f  l  k
   l  v  k  n  f  e  g  g  k  l  -  k  s  e  s  l  q  e  g  l  f  -  n
   l  -  k  t  l  k  e  a  n  y  e  n  q  k  a  y  k  k  v  f  s  k
          LSU                              SSU
```

c.)Polypeptide sequences and SignalP-analysis for LSU and SSU

>*TY*-LSU (GenBank: ACI20377.1)

MVRKVNTFKGNGAVKSQTGTYRAGELRGIIKINEGNCVGCHTCSSVCPAGAVKGSFGDKHSIDLDKCINCGQCLLNCPFGAVE
QMSFVDVVMAKLKDKKTKVVAIIAPAVRVAIGEEFGAEPGTLTVGRLWAALEKAGFLIYDNNFAADQTILEEGTELLAKVAAH
AGLKQLPVELWGKKITLDIKEFSHHPLPQFTSCCPAWVRYVEVFYPKLIPYLSSAKSPQQMAGATAKTYGAKLWGAKPENIFT
VGVMPCTAKIFEASRPEFDSAGKYLKKSGMRDVDAVLTTRDLAELLKRMNIDPMKMSEDASRKPEMFKFYSGGATIFGTSGGV
MEAAVRFAFHVLSGQEPQAMSPKWDFEGVRGFTKPVVSATIPVPLREEYQKAFGTKELQVKVCVVNGIGTDAAHLKPIVEEVL
AGKSPYHFIEVMNCPGGCINGGGQPVHPIELSLLDQLFYSLVKNFEGGKL

Sequence Prediction: Other

Protein type	Other	Signal Peptide (Sec/SPI)	Lipoprotein signal peptide (Sec/SPII)	TAT signal peptide (Tat/SPI)	TAT Lipoprotein signal peptide (Tat/SPII)	Pilin-like signal peptide (Sec/SPIII)
Likelihood	1	0	0	0	0	0

Download: PNG / EPS / Tabular

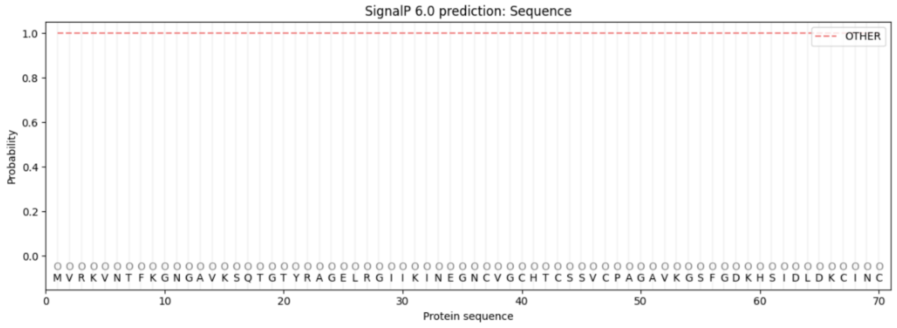

>*TY*-SSU (GenBank: ACI21316.1)

MKIRKL**<u>TRR</u>**S**FLK**LAGAGIISLSFTKP**SFA**GDTAEKNFFETKVGKERLKLIKARQSGQYKDDVISREKFKMAASHENPMIKRF
YSEFAHHPLSEVSEALLHTHYKARV

Sequence
Prediction: TAT signal peptide (Tat/SPI)

Cleavage site between pos. 30 and 31.
Probability 0.855254

Protein type	Other	Signal Peptide (Sec/SPI)	Lipoprotein signal peptide (Sec/SPII)	TAT signal peptide (Tat/SPI)	TAT Lipoprotein signal peptide (Tat/SPII)	Pilin-like signal peptide (Sec/SPIII)
**Likelihood**	0.0001	0.0001	0.0001	0.9643	0.0355	0

Download: PNG / EPS / Tabular

SignalP 6.0 prediction: Sequence

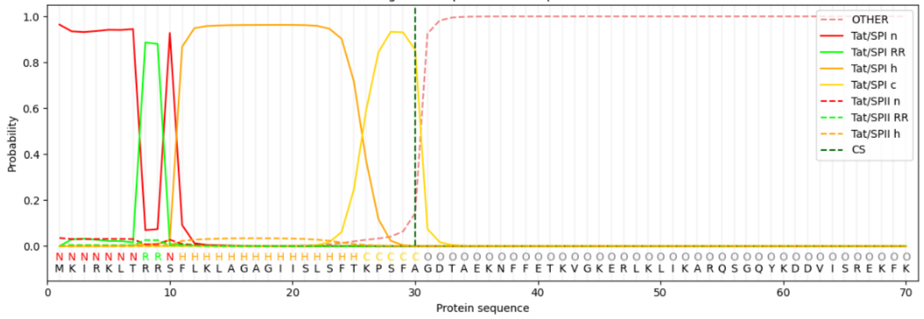

6.) *Sporomusa ovata* (Bacillota; Negativicutes)

GenBank: CTRP01000003.1
GenBank Graphics

>217261-218844 Sporomusa ovata isolate Sporomusa ovata strain An4 genome assembly, contig: SPAN4DRAFT-scaffold-2, whole genome shotgun sequence

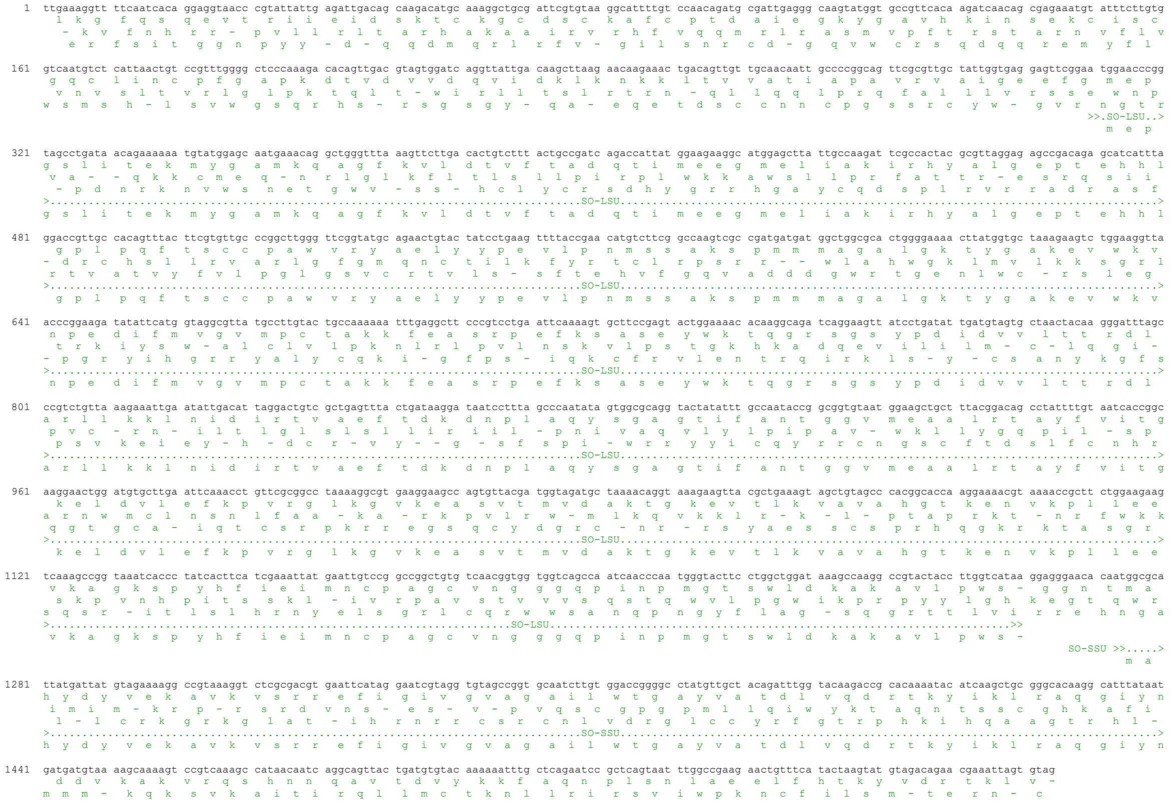

- - c k s k s p s k p - q s g s y - c v q k i c s e s a q - f g r r t v s y - v c r q n e i s v
>..SO-SSU..>>
d d v k a k v r q s h n n q a v t d v y k k f a q n p l s n l a e e l f h t k y v d r t k l v -

b.)Segment between Stop codon of LSU and ATG of SSU including SD-sequence

1 ccgtactacc ttggtca**taa ggagg**gaaca ca**atg**gcgca ttatgattat gtagaaaagg
p y y l g h k e g t q w r i m i m - k r
r t t l v i r r e h n g a l - l c r k
v l p w s - g g n t **m a h y d y v e k**
LSU **SSU**

c.)Polypeptide sequences and SignalP-analysis for LSU and SSU

>*SO*-LSU
MKGFQSQEVTRIIEIDSKTCKGCDSCKAFCPTDAIEGKYGAVHKINSEKCISCGQCLINCPFGAPKDTVDVVDQVIDKLKNKK
LTVVATIAPAVRVAIGEEFGMEPGSLITEKMYGAMKQAGFKVLDTVFTADQTIMEEGMELIAKIRHYALGEPTEHHLGPLPQF
TSCCPAWVRYAELYYPEVLPNMSSAKSPMMMAGALGKTYGAKEVWKVNPEDIFMVGVMPCTAKKFEASRPEFKSASEYWKTQG
RSGSYPDIDVVLTTRDLARLLKKLNIDIRTVAEFTDKDNPLAQYSGAGTIFANTGGVMEAALRTAYFVITGKELDVLEFKPVR
GLKGVKEASVTMVDAKTGKEVTLKVAVAHGTKENVKPLLEEVKAGKSPYHFIEIMNCPAGCVNGGGQPINPMGTSWLDKAKAV
LPWS

Sequence
Prediction: Other

Protein type	Other	Signal Peptide (Sec/SPI)	Lipoprotein signal peptide (Sec/SPII)	TAT signal peptide (Tat/SPI)	TAT Lipoprotein signal peptide (Tat/SPII)	Pilin-like signal peptide (Sec/SPIII)
**Likelihood**	1	0	0	0	0	0

Download: PNG / EPS / Tabular

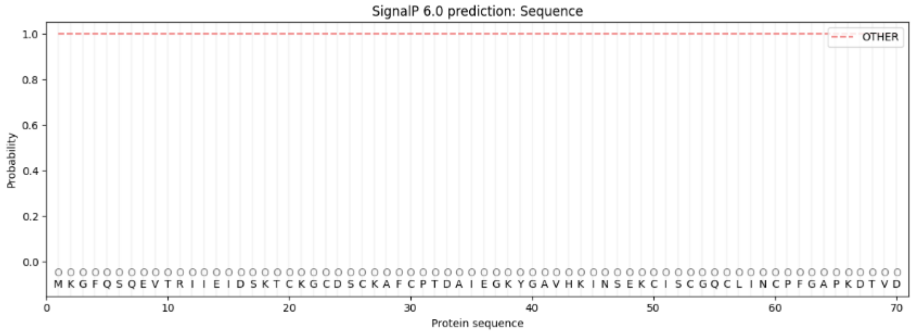

>*SO*-SSU
MAHYDYVEKAVKV**SRREFIG**IVGVAGAILWTGAYVATDLVQDRTKYIKLRAQGIYNDDVKAKVRQSHNNQAVTDVYKKFAQNP
LSNLAEELFHTKYVDRTKLV

Sequence
Prediction: Other

Protein type	Other	Signal Peptide (Sec/SPI)	Lipoprotein signal peptide (Sec/SPII)	TAT signal peptide (Tat/SPI)	TAT Lipoprotein signal peptide (Tat/SPII)	Pilin-like signal peptide (Sec/SPIII)
**Likelihood**	0.8212	0.0829	0.0151	0.0419	0.0162	0.0227

Download: PNG / EPS / Tabular

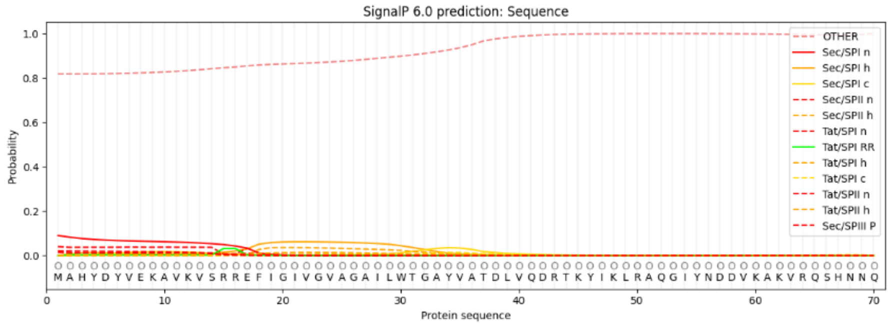

7.) *Syntrophomonas zehnderi* OL4 (Bacillota, Clostridia)

GenBank: CGIH01000013.1

FASTA Graphics

```
   1 atggaggagg gaaatggttt gagcacagct acattggcaa atacagggat aatacaggta actaaaaagt gtaagtcctg tgatcattgt acgtctattt gcccaaccgg tgctattcat gggaagctgg gacaaaaaca tcatatcgat cccaaactat
      m  e  e   g  n  g   l  s  t  a   t  l  a   n  t  g   i  i  q  v   t  k  k   c  k  s   c  d  h  c   t  s  i   c  p  t   g  a  i  h   g  k  l   g  q  k   h  h  i  d   p  k  l
       w  r  r   e  m  v   -  a  q   l  h  w  q   i  q  g   -  y  r   -  l  k  s   v  s  p   v  i  i   v  r  l  f   a  q  p   v  l  f   m  g  s  w   d  k  n   i  i  s   i  p  n  y
        g  g   g  k  w  f   e  h  s   y  i  g   k  y  r  d   n  t  g   n  -  k   v  -  v  l   -  s  l   y  v  y   l  p  n  r   c  y  s   w  e  a   g  t  k  t   s  y  r   s  q  t
    >>...........................................................LSU............................................................>
      m  e  e   g  n  g   l  s  t  a   t  l  a   n  t  g   i  i  q  v   t  k  k   c  k  s   c  d  h  c   t  s  i   c  p  t   g  a  i  h   g  k  l   g  q  k   h  h  i  d   p  k  l

 161 gtattaattg cggacaatgt ttgattaatt gcccgttcgg ggcgataact gacaccagca tggtaaaaga ggtcaaaaag gccctggcag accccagcaa atatgttgtg gttcaggaag ccccggcagt cagagtggct ttaggggaag aatttggcat
      c  i  n   c  g  q  c   l  i  n   c  p  f   g  a  i  t   d  t  s   m  v  k   e  v  k  k   a  l  a   d  p  s   k  y  v  v   v  q  e   a  p  a   v  r  v  a   l  g  e   e  f  g
       v  l  i   a  d  n   v  -  l  i   a  r  s   g  r  -   l  t  p  a   w  -  k   r  s  k   r  p  w  q   t  p  a   n  m  l   w  f  r  k   p  r  q   s  e  w   l  -  g  k   n  l  a
    m  y  -  l   r  t  m   f  d  -   l  p  v  r   g  d  n   -  h  q   h  g  k  r   g  q  k   g  p  g   r  p  q  q   i  c  c   g  s  g   s  p  g  s   q  s  g   f  r  g   r  i  w  h
    >............................................................LSU.................................---------------
..............................................>
      c  i  n   c  g  q  c   l  i  n   c  p  f   g  a  i  t   d  t  s   m  v  k   e  v  k  k   a  l  a   d  p  s   k  y  v  v   v  q  e   a  p  a   v  r  v  a   l  g  e   e  f  g

 321 ggatcccggc accaatgtga agggaaaaat gtacgcttct ttacgcaagc tggggtttga taaagtctac gatacggaat ttgctgccga cctgacaatt atggaggaag gcactgaatt aatccaccgg gtttttaagg cggttggcgc tcccggttac
    m  d  p  g   t  n  v   k  g  k   m  y  a  s   l  r  k   l  g  f   d  k  v  y   d  t  e   f  a  a   d  l  t  i   m  e  e   g  t  e   l  i  h  r   v  f  k   a  v  g   a  p  g  y
     w  i  p   a  p  m  -   r  e  k   c  t  l   l  y  a  s   w  g  l   i  k  s   t  i  r  n   l  l  p   t  -  q   l  w  r  k   a  l  n   -  s  t   g  f  l  r   r  l  a   l  p  v
      g  s  r   h  q  c   e  g  k  n   v  r  f   f  t  q   a  g  v  -   -  s  l   r  y  g   i  c  c  r   p  d  n   y  g  g   r  h  -  i   n  p  p   g  f  -   g  g  w  r   s  r  l
    >............................................................LSU.............................................................>
    m  d  p  g   t  n  v   k  g  k   m  y  a  s   l  r  k   l  g  f   d  k  v  y   d  t  e   f  a  a   d  l  t  i   m  e  e   g  t  e   l  i  h  r   v  f  k   a  v  g   a  p  g  y

 481 gaatccagcg gccctctgcc ccaatttaca tcctgctgcc cggcctggat aaaatacgca gaagatcact atccggctat tctacccaat ctatcatcag ctaaatcccc gcagcagatg tttggcgctg tagccaagac ctatgtggct gaaaagctgg
      e  s  s   g  p  l   p  q  f  t   s  c  c   p  a  w   i  k  y  a   e  d  h   y  p  a   i  l  p  n   l  s  s   a  k  s   p  q  q  m   f  g  a   v  a  k   t  y  v  a   e  k  l
    t  n  p  a   a  l  c   p  n  l   h  p  a  a   r  p  g   -  n  t   q  k  i  t   i  r  l   f  y  p   i  y  h  q   l  n  p   r  s  r   c  l  a  l   -  p  r   p  m  w   l  k  s  w
     r  i  q   r  p  s  a   p  i  y   i  l  l   p  g  l  d   k  i  r   r  r  s   l  s  g  y   s  t  q   s  i  i   s  -  i  p   a  a  d   v  w  r   c  s  q  d   l  c  g   -  k  a
    >............................................................LSU.............................................................>
      e  s  s   g  p  l   p  q  f  t   s  c  c   p  a  w   i  k  y  a   e  d  h   y  p  a   i  l  p  n   l  s  s   a  k  s   p  q  q  m   f  g  a   v  a  k   t  y  v  a   e  k  l

 641 gagtcaaacc ggcagatatg gtttcagttt cggttatgcc ctgtacggct aaaaaatacg aatgtaaccg gccggaaatg atagccagcg gatatcagga tgttgattat gtaataacta cccgcgaact ggcagatatg atcaaagaaa agggcataga
      g  v  k   p  a  d  m   v  s  v   s  v  m   p  c  t  a   k  k  y   e  c  n   r  p  e  m   i  a  s   g  y  q   d  v  d  y   v  i  t   t  r  e   l  a  d  m   i  k  e   k  g  i
       e  s  n   r  q  i   w  f  q  f   r  l  c   p  v  r   l  k  n  t   n  v  t   g  r  k   -  -  p  a   d  i  r   m  l  i   m  -  -  l   p  a  n   w  q  i   -  s  k  k   r  a  -
    g  s  q  t   g  r  y   g  f  s   f  g  y  a   l  y  g   -  k  i   r  m  -  p   a  g  n   d  s  q   r  i  s  g   c  -  l   c  n  n   y  p  r  t   g  r  y   d  q  r   k  g  h  r
    >............................................................LSU.............................................................>
      g  v  k   p  a  d  m   v  s  v   s  v  m   p  c  t  a   k  k  y   e  c  n   r  p  e  m   i  a  s   g  y  q   d  v  d  y   v  i  t   t  r  e   l  a  d  m   i  k  e   k  g  i

 801 ctttaacagt ctccccaatg aagaagccga tcgcctggtg ggagcttcta ccggtgctgc cactatcttt ggggtaaccg gcggggtcat ggaagctgct ttgcggacgg cctatgaact gctcagcggc caatcactgg ggaaagtcga attcaaatcc
    d  f  n  s   l  p  n   e  e  a   d  r  l  v   g  a  s   t  g  a   a  t  i  f   g  v  t   g  g  v   m  e  a  a   l  r  t   a  y  e   l  l  s  g   q  s  l   g  k  v   e  f  k  s
     t  l  t   v  s  p  m   k  k  p   i  a  w   w  e  l  l   p  v  l   p  l  s   l  g  -  p   a  g  s   w  k  l   l  c  g  r   p  m  n   c  s  a   a  n  h  w   g  k  s   n  s  n
      l  -  q   s  p  q   -  r  s  r   s  p  g   g  s  f   y  r  c  c   h  y  l   w  g  n   r  r  g  h   g  s  c   f  a  d   g  l  -  t   a  q  r   p  i  t   g  e  s  r   i  q  i
    >............................................................LSU.............................................................>
    d  f  n  s   l  p  n   e  e  a   d  r  l  v   g  a  s   t  g  a   a  t  i  f   g  v  t   g  g  v   m  e  a  a   l  r  t   a  y  e   l  l  s  g   q  s  l   g  k  v   e  f  k  s

 961 gtacgcggcc aaaaacctat tcgcgaagca actgtagaga tccctattaa agatctggga actacgttac cggtaaaagt atgtgtcgtt accggaacca aatatgtagg acgggtaatt gaggatgttc tggccggacg tagctcatac cactttatcg
      v  r  g   q  k  p   i  r  e  a   t  v  e   i  p  i   k  d  l  g   t  t  l   p  v  k   v  c  v  v   t  g  t   k  y  v   g  r  v  i   e  d  v   l  a  g   r  s  s  y   h  f  i
    p  y  a  a   k  n  l   f  a  k   q  l  -  r   s  l  l   k  i  w   e  l  r  y   r  -  k   y  v  s   l  p  e  p   n  m  -   d  g  -   l  r  m  f   w  p  d   v  a  h   t  t  l  s
     r  t  r   p  k  t  y   s  r  s   n  c  r   d  p  y  -   r  s  g   n  y  v   t  g  k  s   m  c  r   y  r  n   q  i  c  r   t  g  n   -  g  c   s  g  r  t   -  l  i   p  l  y
    >............................................................LSU.............................................................>
      v  r  g   q  k  p   i  r  e  a   t  v  e   i  p  i   k  d  l  g   t  t  l   p  v  k   v  c  v  v   t  g  t   k  y  v   g  r  v  i   e  d  v   l  a  g   r  s  s  y   h  f  i

1121 aagtgatgaa ctgtccgggc ggttgcataa atggcggcgg gcagcctata cgccgcgata tgtattaaga gagggggatg taaatgagta tttttagaga aaaaggcacc ttcacccgca ggcagttttt caaaggctcg ggcatgctgg cagcaaccat
      e  v  m   n  c  p  g   g  c  i   n  g  g   g  q  p  i   r  r  d   m  y  -   e  r  g  m   -  m  s   i  f  r   e  k  g  t   f  t  r   r  q  f   f  k  g  s   g  m  l   a  a  t
       k  -  -   t  v  r   a  v  a  -   m  a  a   g  s  l   y  a  a  i   c  i  k   r  g  g   c  k  -  v   f  l  e   k  k  a   p  s  p  a   g  s  f   s  k  a   r  a  c  w   q  q  p
    r  s  d  e   l  s  g   r  l  h   k  w  r  r   a  a  y   t  p  r   y  v  l  r   e  g  d   v  n  e   y  f  -  r   k  r  h   l  h  p   q  a  v  f   q  r  l   g  h  a   g  s  n  h
    >...............................LSU...............................>>
      e  v  m   n  c  p  g   g  c  i   n  g  g   g  q  p  i   r  r  d   m  y  -
                                                                                           >>...................................SSU......................................>
                                                                                            m  s   i  f  r   e  k  g  t   f  t  r   r  q  f   f  k  g  s   g  m  l   a  a  t

1281 cgtaataagc ggcgttttcg ctaaatttgg ttacgatgcc tgggcagcca gcgatgcata catagaaaaa cgtatcagtg gtctttatac cctggatgaa aaaatggcga tcagaaaatc tcatcagaat ccagaaatac tgcaaattta caaggatttt
    i  v  i  s   g  v  f   a  k  f   g  y  d  a   w  a  a   s  d  a   y  i  e  k   r  i  s   g  l  y   t  l  d  e   k  m  a   i  r  k   s  h  q  n   p  e  i   l  q  i   y  k  d  f
     s  -  -   a  a  f  s   l  n  l   v  t  m   p  g  q  p   a  m  h   t  -  k   n  v  s  v   v  f  i   p  w  m   k  k  w  r   s  e  n   l  i  r   i  q  k  y   c  k  f   t  r  i
      r  n  k   r  r  f   r  -  i  w   l  r  c   l  g  s   q  r  c  i   h  r  k   t  y  q   w  s  l  y   p  g  -   k  n  g   d  q  k  i   s  s  e   s  r  n   t  a  n  l   q  g  f
    >............................................................SSU.............................................................>
    i  v  i  s   g  v  f   a  k  f   g  y  d  a   w  a  a   s  d  a   y  i  e  k   r  i  s   g  l  y   t  l  d  e   k  m  a   i  r  k   s  h  q  n   p  e  i   l  q  i   y  k  d  f

1441 ctgtctccgg gagaagttaa gcctgtaagt gaaaaagctc atcatctcct gcacaccaaa tacggccagg acattcctga attgatcaaa gaactgcatg ctcaccagca tgacgccgcg tag
      l  s  p   g  e  v   k  p  v  s   e  k  a   h  h  l   l  h  t  k   y  g  q   d  i  p   e  l  i  k   e  l  h   a  h  q   h  d  a  a   -
    f  c  l  r   e  k  l   s  l  -   v  k  k  l   i  i  s   c  t  p   n  t  a  r   t  f  l   n  -  s   k  n  c  m   l  t  s   m  t  p   r
     s  v  s   g  r  s  -   a  c  k   -  k  s   s  s  s  p   a  h  q   i  r  p   g  h  s  -   i  d  q   r  t  a   c  s  p  a   -  r  r   v
    >.......................................SSU..........................................>>
      l  s  p   g  e  v   k  p  v  s   e  k  a   h  h  l   l  h  t  k   y  g  q   d  i  p   e  l  i  k   e  l  h   a  h  q   h  d  a  a   -
```

b.) Segment between Stop codon of LSU and ATG of SSU including SD-sequence

```
cgccgcgata tgtattaaga gagggggatg taaatgagta tttttagaga
  r  r  d   m  y  -   e  r  g  m   -  m  s   i  f  r
   a  a  i   c  i  k   r  g  g   c  k  -  v   f  l  e
    p  r   y  v  l  r   e  g  d   v  n  e   y  f  -  r
      LSU                                  SSU
```

c.)Polypeptide sequences and SignalP-analysis for LSU and SSU

>*SZ*-LSU

MEEGNGLSTATLANTGIIQVTKKCKSCDHCTSICPTGAIHGKLGQKHHIDPKLCINCGQCLINCPFGAITDTSMVKEVKKALA
DPSKYVVVQEAPAVRVALGEEFGMDPGTNVKGKMYASLRKLGFDKVYDTEFAADLTIMEEGTELIHRVFKAVGAPGYESSGPL
PQFTSCCPAWIKYAEDHYPAILPNLSSAKSPQQMFGAVAKTYVAEKLGVKPADMVSVSVMPCTAKKYECNRPEMIASGYQDVD
YVITTRELADMIKEKGIDFNSLPNEEADRLVGASTGAATIFGVTGGVMEAALRTAYELLSGQSLGKVEFKSVRGQKPIREATV
EIPIKDLGTTLPVKVCVVTGTKYVGRVIEDVLAGRSSYHFIEVMNCPGGCINGGGQPIRRDMY

**Sequence**	
**Prediction:** Other	

Protein type	Other	Signal Peptide (Sec/SPI)	Lipoprotein signal peptide (Sec/SPII)	TAT signal peptide (Tat/SPI)	TAT Lipoprotein signal peptide (Tat/SPII)	Pilin-like signal peptide (Sec/SPIII)
**Likelihood**	1	0	0	0	0	0

Download: PNG / EPS / Tabular

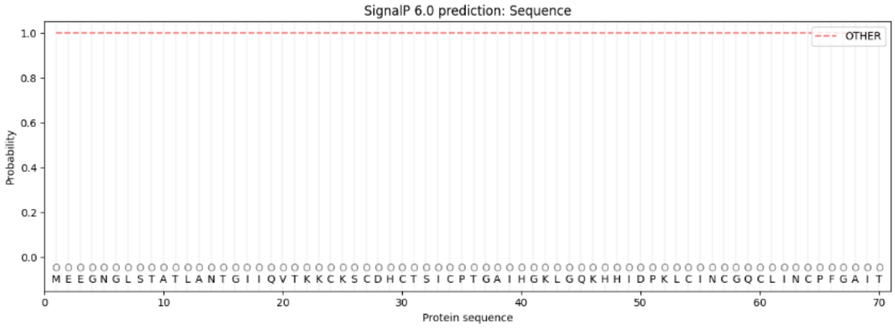

>*SZ*-SSU

MSIFREKGTF**TRRQFFK**GSGMLAATIVISGVFAKFGYDAWA↓ASDAYIEKRISGLYTLDEKMAIRKSHQNPEILQIYKDFLSP
GEVKPVSEKAHHLLHTKYGQDIPELIKELHAHQHDAA

Sequence
Prediction: TAT signal peptide (Tat/SPI)

Cleavage site between pos. 41 and 42. Probability 0.822816

Protein type	Other	Signal Peptide (Sec/SPI)	Lipoprotein signal peptide (Sec/SPII)	TAT signal peptide (Tat/SPI)	TAT Lipoprotein signal peptide (Tat/SPII)	Pilin-like signal peptide (Sec/SPIII)
**Likelihood**	0.0001	0.0001	0	0.9741	0.0257	0

Download: PNG / EPS / Tabular

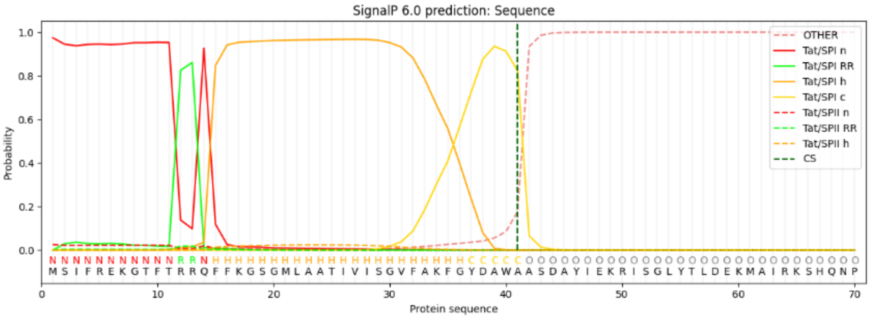

8.) *Dehalobacter* sp. TBBPA1

GenBank: CP162385.1
FASTA Graphics

a.) 213918-215482| Dehalobacter sp. TBBPA1 chromosome, complete genome.

```
  1 atgagttcat tatacgccga aaaaattatt caaatatctg atcgctgccg gtcctgtgat cattgtactt cggtttgtcc aagcggcgct attgccggga aactaggcga acagcactac attgaccata ataaatgctt aaattgcggg cagtgtctga
     m  s  s  l  y  a  e  k  i  i  q  i  s  d  r  c  r  s  c  d  h  c  t  s  v  c  p  s  g  a  i  a  g  k  l  g  e  q  h  y  i  d  h  n  k  c  l  n  c  g  q  c  l
      -  v  h  y  t  p  k  k  l  f  k  y  l  i  a  a  g  p  v  i  i  v  l  r  f  v  q  a  a  l  l  p  g  n  -  a  n  s  t  t  l  t  i  i  n  a  -  i  a  g  s  v  -
       e  f  i  i  r  r  k  n  y  s  n  i  -  s  l  p  v  l  -  s  l  y  f  g  l  s  k  r  r  y  c  r  e  t  r  r  t  a  l  h  -  p  -  -  m  l  k  l  r  a  v  s
    >>...............................................................LSU...............................................................>
     m  s  s  l  y  a  e  k  i  i  q  i  s  d  r  c  r  s  c  d  h  c  t  s  v  c  p  s  g  a  i  a  g  k  l  g  e  q  h  y  i  d  h  n  k  c  l  n  c  g  q  c  l

161 ttaattgccc tgcggaaata attaaagact tgagcatggc tgaagaagta aaggcggcct tggctaaccc gaaaaaattt gttattgttc agactgctcc gtcagtcagg gttgcattag gtgaagagtt cggcaaggcc cctggctata atgccaaagg
     i  n  c  p  a  e  i  i  k  d  l  s  m  a  e  e  v  k  a  a  l  a  n  p  k  k  f  v  i  v  q  t  a  p  s  v  r  v  a  l  g  e  e  f  g  k  a  p  g  y  n  a  k
      l  i  a  l  r  k  -  l  k  t  -  a  w  l  k  k  -  r  r  p  w  l  t  r  k  n  l  l  l  f  r  l  l  r  q  s  g  l  h  -  v  k  s  s  a  r  p  l  a  i  m  p  k
    d  -  l  p  c  g  n  n  -  r  l  e  h  g  -  r  s  k  g  g  l  g  -  p  e  k  i  c  y  c  s  d  c  s  v  s  q  g  c  i  r  -  r  v  r  q  g  p  w  l  -  c  q  r
    >................................................................LSU...............................................................>
     i  n  c  p  a  e  i  i  k  d  l  s  m  a  e  e  v  k  a  a  l  a  n  p  k  k  f  v  i  v  q  t  a  p  s  v  r  v  a  l  g  e  e  f  g  k  a  p  g  y  n  a  k

321 taaaatgttt gcatctttaa gaaaattagg atttgataaa gtttacgata cggagtttac cgccgatctg accattatgg aagagggcag tgaattaatt aacagagtat ttaaagcagt cgggcagccg ggctatgaaa caagcggccc cttgcctcag
    g  k  m  f  a  s  l  r  k  l  g  f  d  k  v  y  d  t  e  f  t  a  d  l  t  i  m  e  e  g  s  e  l  i  n  r  v  f  k  a  v  g  q  p  g  y  e  t  s  g  p  l  p  q
     v  k  c  l  h  l  -  e  n  -  d  l  i  k  f  t  i  r  s  l  p  p  i  -  p  l  w  k  r  a  v  n  -  l  t  e  y  l  k  q  s  g  s  r  a  m  k  q  a  a  p  c  l
      -  n  v  c  i  f  k  k  i  r  i  -  -  s  l  r  y  g  v  y  r  r  s  d  h  y  g  r  g  q  -  i  n  -  q  s  i  -  s  s  r  a  a  g  l  -  n  k  r  p  l  a  s
    >................................................................LSU...............................................................>
    g  k  m  f  a  s  l  r  k  l  g  f  d  k  v  y  d  t  e  f  t  a  d  l  t  i  m  e  e  g  s  e  l  i  n  r  v  f  k  a  v  g  q  p  g  y  e  t  s  g  p  l  p  q

481 tttacaagct gctgcccggc ttgggtaaaa tatgcggaag ataattaccc gaaaatattg ccccatgtgt cttcagcaaa atctccgcag caaatgttcg gcgctgtagc aaagacatat ttgcctcaaa aattgaatat tgaacccgcc gatatttttt
     f  t  s  c  c  p  a  w  v  k  y  a  e  d  n  y  p  k  i  l  p  h  v  s  s  a  k  s  p  q  q  m  f  g  a  v  a  k  t  y  l  p  q  k  l  n  i  e  p  a  d  i  f
    s  l  q  a  a  a  r  l  g  -  n  m  r  k  i  i  t  r  k  y  c  p  m  c  l  q  q  n  l  r  s  k  c  s  a  l  -  q  r  h  i  c  l  k  n  -  i  l  n  p  p  i  f  f
```

```
      v y k  l l p g  l g k  i c g  r - l p  e n i  a p c  v f s k  i s a  a n v  r r c s  k d i  f a s  k i e y  - t r  r y f
     >...............................................................LSU...............................................................>
      f t s  c c p  a w v k  y a e  d n y  p k i l  p h v  s s a  k s p q  q m f  g a v  a k t y  l p q  k l n  i e p a  d i f

641  gtgtttcggt catgccttgt accgctaaaa aatacgaatg cagccgtccg gaaatgatca gcagcggtta ccaggatgtt aatgctgtcc tgactacgcg ggaattggcc caactaatta aagaagccgg gatcgatttt gacagtctgc ctgaggaagc
      c v s  v m p c  t a k  k y e  c s r p  e m i  s s g  y q d v  n a v  l t t  r e l a  q l i  k e a  g i d f  d s l  p e e
      v f r  s c l  v p l k  n t n  a a v  r k - s  a a v  t r m  l m l s  - l r  g n w  p n - l  k k p  g s i  l t v c  l r k
     l c f g  h a l  y r -  k i r m  q p s  g n d  q q r l  p g c  - c c  p d y a  g i g  p t n  - r s r  d r f  - q s  a - g s
     >...............................................................LSU...............................................................>
      c v s  v m p c  t a k  k y e  c s r p  e m i  s s g  y q d v  n a v  l t t  r e l a  q l i  k e a  g i d f  d s l  p e e

801  tgcggattct ttcgtgggac agtccacagg cgcggcgaca atattcggtg ctaccggagg tgttatggaa gcggcgttgc gtacggcgta tgaactgtta agcggtcagt ctctggaaca aattgatttt aaagctgtgc gtggtctaag tccggtaaga
     a a d s  f v g  q s t  g a a t  i f g  a t g  g v m e  a a l  r t a  y e l l  s g q  s l e  q i d f  k a v  r g l  s p v r
      l r i  l s w d  s p q  a r r  q y s v  l p e  v l w  k r r c  v r r  m n c  - a v s  l w n  k l i  l k l c  v v -  v r -
      c g f  f r g  t v h r  r g d  n i r  c y r r  c y g  s g v  a y g v  - t v  k r s  v s g t  n - f  - s c  a w s k  s g k
     >...............................................................LSU...............................................................>
     a a d s  f v g  q s t  g a a t  i f g  a t g  g v m e  a a l  r t a  y e l l  s g q  s l e  q i d f  k a v  r g l  s p v r

961  gaagctacgg tttcgattcc gataaaagct ctgggtggtg cggttctgcc ggtaaaagtg gcgatcgtaa caggaactaa acatgtggcc tcgctcattg aagatgtctt agcgggacgc agtaattatc attttattga agtcatgaat tgtccgggcg
      e a t  v s i  p i k a  l g g  a v l  p v k v  a i v  t g t  k h v a  s l i  e d v  l a g r  s n y  h f i  e v m n  c p g
     e k l r  f r f  r - k  l w v v  r f c  r - k  w r s -  q e l  n m w  p r s l  k m s  - r d  a v i i  i l l  k s -  i v r a
      r s y  g f d s  d k s  s g w  c g s a  g k s  g d r  n r n -  t c g  l a h  - r c l  s g t  q - l  s f y -  s h e  l s g
     >...............................................................LSU...............................................................>
      e a t  v s i  p i k a  l g g  a v l  p v k v  a i v  t g t  k h v a  s l i  e d v  l a g r  s n y  h f i  e v m n  c p g

1121 gatgcattaa cggcggcgga caaccgatcc gccgggatgc ttaaatagaa aatagagaaa gggagaaaac gggatggcta ttttttacga gaaagatggg attaataggc ggcagttttt aaagggagcg ggtgcggtaa taatcgccgc tgcttttcct
      g c i  n g g g  q p i  r r d  a - i e  n r e  r e k  t g w l  f f t  r k m  g l i g  g s f  - r e  r v r -  - s p  l l f
      d a l  t a a  d n r s  a g m  l k -  k i e k  g r k  r d g  y f l r  e r w  d - -  a a v f  k g s  g c g  n n r r  c f s
     r m h -  r r r  t t d  p p g c  l n r  k - r  k g e n  g m a  i f y  e k d g  i n r  r q f  l k g a  g a v  i i a  a a f p
     >...................LSU.....................>>
      g c i  n g g g  q p i  r r d  a -
                                                                              >>........................................SSU...............................................>
                                                                               m a  i f y  e k d g  i n r  r q f  l k g a  g a v  i i a  a a f p

1281 gggatcttta caaagatcgg gcttggccgg actcataaaa gcaccgaata tattgcgcag agagctgccg gtttatattc gttagatgaa aagatgacta tccgcaaatc acacgaaaat tcggaagttt tgcaattata caaagacttt ctctctccgg
     l g s l  q r s  g l a  g l i k  a p n  i l r  r e l p  v y i  r - m  k r - l  s a n  h t k  i r k f  c n y  t k t  f s l r
      w d l  y k d r  a w p  d s -  k h r i  y c a  e s c  r f i f  v r -  k d d  y p q i  t r k  f g s  f a i i  q r l  s l s
      g i f  t k i  g l g r  t h k  s t e  y i a q  r a a  g l y  s l d e  k m t  i r k  s h e n  s e v  l q l  y k d f  l s p
     >...............................................................SSU...............................................................>
      g i f  t k i  g l g r  t h k  s t e  y i a q  r a a  g l y  s l d e  k m t  i r k  s h e n  s e v  l q l  y k d f  l s p

1441 gagaggtgaa gccgctcagc tcgacttccc atcatttgct gcataccaag tatggcaagg aagttacggc ttatattgaa gaattaaagg atgagagtgc cggtaaggaa aaagttgctt cttaa
      e r -  s r s  a r l p  i i c  c i p  s m a r  k l r  l i l  k n - r  m r v  p v r  k k l l  l
     g r g e  a a q  l d f  p s f a  a y q  v w q  g s y g  l y -  r i k  g - e c  r - g  k s c  f l
      g e v  k p l s  s t s  h h l  l h t k  y g k  e v t  a y i e  e l k  d e s  a g k e  k v a  s -
     >.....................................................SSU.......................................................>>
      g e v  k p l s  s t s  h h l  l h t k  y g k  e v t  a y i e  e l k  d e s  a g k e  k v a  s -
```

b.)Segment between Stop codon of LSU and ATG of SSU including SD-sequence

```
1  caaccgatcc gccgggatgc ttaaatagaa aatagagaaa gggagaaaac gggatggcta ttttttacga
    q  p  i   r  r  d  a  -  i  e  n  r  e  r  e  k  t  g  w  l  f  f  t
     n  r  s  a  g  m  l  k  -  k  i  e  k  g  r  k  r  d  g  y  f  l  r
      t  d  p  p  g  c  l  n  r  k  -  r  k  g  e  n  g  m  a  i  f  y
```

c.)Polypeptide sequences and SignalP-analysis for LSU and SSU

>*D*-LSU (protein-id: XDG72785.1)

MSSLYAEKIIQISDRCRSCDHCTSVCPSGAIAGKLGEQHYIDHNKCLNCGQCLINCPAEIIKDLSMAEEVKAALANPKKFVIV
QTAPSVRVALGEEFGKAPGYNAKGKMFASLRKLGFDKVYDTEFTADLTIMEEGSELINRVFKAVGQPGYETSGPLPQFTSCCP
AWVKYAEDNYPKILPHVSSAKSPQQMFGAVAKTYLPQKLNIEPADIFCVSVMPCTAKKYECSRPEMISSGYQDVNAVLTTREL
AQLIKEAGIDFDSLPEEAADSFVGQSTGAATIFGATGGVMEAALRTAYELLSGQSLEQIDFKAVRGLSPVREATVSIPIKALG
GAVLPVKVAIVTGTKHVASLIEDVLAGRSNYHFIEVMNCPGGCINGGGQPIRRDA

Sequence
Prediction: Other

Protein type	Other	Signal Peptide (Sec/SPI)	Lipoprotein signal peptide (Sec/SPII)	TAT signal peptide (Tat/SPI)	TAT Lipoprotein signal peptide (Tat/SPII)	Pilin-like signal peptide (Sec/SPIII)
**Likelihood**	1	0	0	0	0	0

Download: PNG / EPS / Tabular

SignalP 6.0 prediction: Sequence

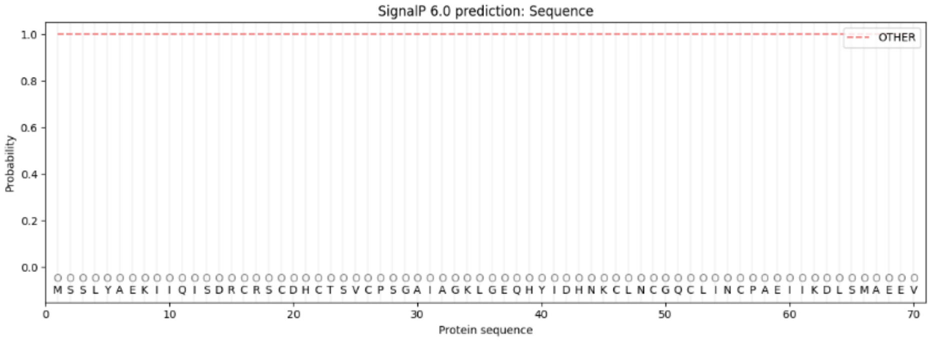

>*D*-SSU(protein-id: XDG72786.1)

MAIFYEKDGIN**RR**Q**FLK**GAGAVIIAAAFPGIFTKIGLG↓RTHKSTEYIAQRAAGLYSLDEKMTIRKSHENSEVLQLYKDFLSP
GEVKPLSSTSHHLLHTKYGKEVTAYIEELKDESAGKEKVAS

Sequence
Prediction: TAT signal peptide (Tat/SPI)

Cleavage site between pos. 38 and 39. Probability 0.470870

Protein type	Other	Signal Peptide (Sec/SPI)	Lipoprotein signal peptide (Sec/SPII)	TAT signal peptide (Tat/SPI)	TAT Lipoprotein signal peptide (Tat/SPII)	Pilin-like signal peptide (Sec/SPIII)
Likelihood	0.0023	0.0001	0.0001	0.8739	0.1236	0

Download: PNG / EPS / Tabular

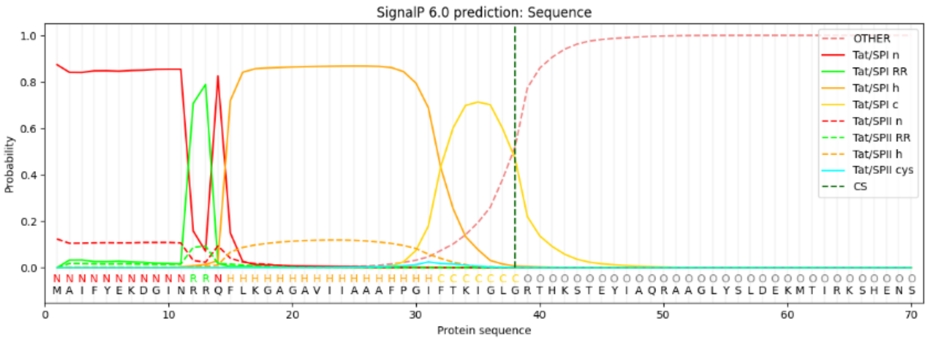

III.) Sequence alignments of all large and small subunits

(a) Alignment large subunits (LSUs)

```
DdH-LSU    1 MSRTVMERIEYEMHTPDPKADPDKLH------------------------FVQIDEAKCIGCDTCSQYCPTAAIFG-EMGEPHSIPHIEACINCGQCLTHCPENAIYEA
DS-LSU     1 -MAACRVKEKPKVLPVDLSAIIPEKEGTMRKMEDVIYLNNAPHHEEPDNIYFVQVDPTKCQGCGECEEHCATGAIQSINEEGIHQVLSPSACMNCGQCLANCPYGAIYEG
TY-LSU     1 MVR---KVNTF-----KGNGAVKSQTGTYRAGE----LRG-----------IIKINEGNCVGCHTCSSVCPAGAVKG-SFGDKHSIDL-DKCINCGQCLLNCPFGAVEQ-
CR-LSU     1 -MLSVYKIKTFPPAPNSRGGEAD-FEGTYRKGE----LRG-----------IIRIDQDSCVGCDTCRSFCPTDAIDG-SLGVAHKIDQ-NLCVACGQCLINCPFAVIEQ-
SW-LSU     1 MFKDTMTIHTFGPGENAYGQNGGAYEGNLRKGE----LRG-----------IIHINKDHCVGCDTCRKFCPTDAIKG-GLGAKHESID-DACLYCGQCLVACPFNAIEQ-
SO-LSU     1 -----------------------MKG-FQSQE----VTR-----------IIEIDSKTCKGCDSCKAFCPTDAIEG-KYGAVHKINS-EKCISCGQCLINCPFGAPKDT
SZ-LSU     1 --------------MEEGNGLSTATLAN----------TG-----------IIQVT-KKCKSCDHCTSICPTGAIHG-KLGQKHHIDP-KLCINCGQCLINCPFGAITD-
Dsp-LSU    1 ----------------------MSSLYA----------EK-----------IIQIS-DRCRSCDHCTSVCPSGAIAG-KLGEQHYIDH-NKCLNCGQCLINCPAEIIKD-

DdH-LSU   85 QSWVPEVEKKLKDGKVKCIAMPAPAVRYALGDAFGMPVGSVTTGKMLAALQKLGFAHCWDTEFTADVTIWEEGSEF---------VERLTKKS--------------DM
DS-LSU   110 VSFVDEVFEKLRDPETVVVSMPAPAVRYALGECFGYSPGTYVGGKMHAALRKLGFDYIWDNEFAADLTIMEEGTEL---------IERIKHPSKDK-------------
TY-LSU    85 MSFVDVVMAKLKDKKTKVVAIIAPAVRVAIGEEFGAEPGTLTVGRLWAALEKAGF-LIYDNNFAADQTILEEGTELLAKVAAHAGLKQLPVELWGKKITLDIKEFSHH--
CR-LSU    91 MSFVDEVMQKLDDEKTFVVAHPSPAVRVSLAEEFGGKPGELTVNKMYNAFEKAGF-NMYDVNFAADQTILEEGTEL---------IKKIKYWLLGER-SHDLEHVSHH--
SW-LSU    93 MSFVDEVERVLDAKDRIVVAQPSPAVRVSICEEFGGEPGELSTEQMVNALEALGC-VTYDCNSSADQTIIEEGTEF---------VKKVQYWVLGER-GPEVDEQGKH--
SO-LSU    69 VDVVDQVIDKLKNKKLTVVATIAPAVRVAIGEEFGMEPGSLITEKMYGAMKQAGF-KVLDTVFTADQTIMEEGMEL---------IAKIRHYALGE-------PTEHHLG
SZ-LSU    72 TSMVKEVKKALADPSKYVVVQEAPAVRVALGEEFGMDPGTNVKGKMYASLRKLGFDKVYDTEFAADLTIMEEGTEL---------IHRV-FKAVG-APGYE----SSG--
Dsp-LSU   64 LSMAEEVKAALANPKKFVIVQTAPSVRVALGEEFGKAPGYNAKGKMFASLRKLGFDKVYDTEFTADLTIMEEGSEL---------INRV-FKAVG-QPGYE----TSG--

DdH-LSU  171 PLPQFTSCCPGWQKYAETYYPELLPHFSTCKSPIGMNGALAKTYGAERM-KYDPKQVYTVSIMPCIAKKYEGLRPELKSSG--------------MRDIDATLTTRELA
DS-LSU   197 PLPQFTSCCPGWVKFCESFYPDLLPYLSTCKSPIGMLSALAKTYGAHQT-HTPGQKIYTVSIMPCIAKKYEGLRPEMADSG--------------FRDIDATINTRELA
TY-LSU   192 PLPQFTSCCPAWVRYVEVFYPKLIPYLSSAKSPQQMAGATAKTYGAK-LWGAKPENIFTVGVMPCTAKIFEASRPEFDSAGKYLKKSG--------MRDVDAVLTTRDLA
CR-LSU   188 PFPHFTSCCPAWVRNAEIFHPELIPHISGAKSPIQMGGPLAKTWAAKFVWDKDPRDIYVATVTPCTAKIYEASRPEFNSAYEYLKERGEIPADTKSFPDIDATLTARDIA
SW-LSU   190 PFPHFTSCCPGWVKYAETYAADMLPHLSTAKSPLQMGGTLAKTWAAKHILKCDPRKVYFVSMTPCTAKIFEASRPEMNTAWRWLIEHKEIPANTPSFQDIDASLTARDLA
SO-LSU   162 PLPQFTSCCPAWVRYAELYYPEVLPNMSSAKSPMMMAGALGKTYGAKEVWKVNPEDIFMVGVMPCTAKKFEASRPEFKSASEYWKTQG----RSGSYPDIDVVLTTRDLA
SZ-LSU   165 PLPQFTSCCPAWIKYAEDHYPAILPNLSSAKSPQQMFGAVAKTYVAEKL-GVKPADMVSVSVMPCTAKKYECNRPEMIASG--------------YQDVDYVITTRELA
Dsp-LSU  157 PLPQFTSCCPAWVKYAEDNYPKILPHVSSAKSPQQMFGAVAKTYLPQKL-NIEPADIFCVSVMPCTAKKYECSRPEMISSG--------------YQDVNAVLTTRELA
```

```
                                       PLUG                                 LID
DdH_LSU  265 YMIKKAGIDFAKLPDGKR-------DSLMGESTGGATIFGVTGGVMEAALRFAYEAVTGKK----PDSWDFKAVRGLDG-IKEATVNV-----GGT-------DVKVAVV
DS-LSU   291 YMIKKAGIDFRSLPS-------QDPIPVLGMSTGAATIFGTSGGVMEAALRLAYEVLSGQKL----TKPDIKIVR-THEGIKAADIKIP-----------KFGTIKVAVA
TY-LSU   293 ELLKRMNIDPMKMSE----DASRKPE-MFKFYSGGATIFGTSGGVMEAAVRFAFHVLSGQEPQAMSPKWDFEGVRGFTKPVVSATIPVPLREEYQKAFGTKELQVKVCVV
CR-LSU   298 EILRKKGINPLEM-------SDEYPEKTMNVYTGGGTIFGNSGGVMEAALRTAYFLLSGQE---LKDP-DLTPVRGYDKDLTEAVIPIPLKDYDGKTL-----ELKVAVV
SW-LSU   300 ELFRRKGINPLLMPKTRKRDSE---THPLEVYSGAGTIFGCSGGVMEAALRTAYFALAGKE---LDNK-DIEVVRGHNNAIIEATIPVPVKELGGKIF-----EVRVCVV
SO-LSU   268 RLLKKLNIDIRTVAEFTD------KDNPLAQYSGAGTIFANTGGVMEAALRTAYFVITGKEL----DVLEFKPVRGLK-GVKEASVTM-VDAKTGKEV-----TLKVAVA
SZ-LSU   259 DMIKEKGIDFNSLPN-------EEADRLVGASTGAATIFGVTGGVMEAALRTAYELLSGQSL----GKVEFKSVRGQ-KPIREATVEIPIKDLG-TTL-----PVKVCVV
Dsp-LSU  251 QLIKEAGIDFDSLPE-------EAADSFVGQSTGAATIFGATGGVMEAALRTAYELLSGQSL----EQIDFKAVRGL-SPVREATVSIPIKALGGAVL-----PVKVAIV

DdH_LSU  351 HGAKRFKQ----VCDDVKAGKSPYHFIEYMACPGGCVCGGGQPVMPGVLEA↓MDRTTTRLYAGLKKRLAMASANKA
DS-LSU   378 SGLKNAA----KLCEEVRAGKSPYHFIEIMACPGGCVNGGGQPLDPSIREEA--SLFKRMIAKINKRYKGRKPTIS----
TY-LSU   398 NGIGTDAAHLKPIVEEVLAGKSPYHFIEVMNCPGGCINGGGQPVHPIEL-----SLLDQLFYSLVKNFEGGKL-------
CR-LSU   392 NGASRNLNT---ILKHITKDSNRYHFIEVMNCPGGCVNGGGQPVHAMGT-----SWLHSLLPLPLKA-------------
SW-LSU   398 NGCNQGIAE---VLHRVRVDKNRYHFIEVMNCPGGCVNGGGQPVQPVGT-----SWLKPTTPLPLRV-------------
SO-LSU   361 HGTKE---NVKPLLEEVKAGKSPYHFIEIMNCPAGCVNGGGQPINPMGT-----SWLDKAKAVLPWS-------------
SZ-LSU   351 TGTKYVG----RVIEDVLAGRSSYHFIEVMNCPGGCINGGGQPIRRDMY-------------------------------
Dsp-LSU  344 TGTKHVA----SLIEDVLAGRSNYHFIEVMNCPGGCINGGGQPIRRDA--------------------------------
```

(b) Alignment small subunits (SSUs)

```
                          |n-region|      h-region      | c-region |                   LOCK
DdH-SSU   1 -----------MQIASITRRGFLKVACVTTGAALIGIRMTG---KAVAA↓----VKQIKDYMLDRINGVYGADAKFP-VRA
DS-SSU    1 ---------MKRIV-TISRRSFLKTAGIAVGYMVLGFNLT---KQAVAA--------TMEFIGLRQKSVYEADSKVYKIRK
TY-SSU    1 -----------MKIRKLTRRSFLKLAGAGIISLSFTKPSFAGDTAEKNFFETKVGKERLKLIKARQSGQYK-DDVIS-REK
CR-SSU    1 ---MRYQFIEKPVGKIFSRRDFLKVSGVLTSIIAISGY-------AITD----IIKRRKSYIAMRQEGLYK-DDKRC-QDK
SW-SSU    1 MFSQNYSYAERPAALILGRRGFLKVSGLCIGAAVVCGW-------AIGD----MVSRRSSIILARQAGLYQ-DDKLC-QAM
SO-SSU    1 --MAHYDYVEK--AVKVSRREFIGIVGVAGAILWTGAY-------VATD----LVQDRTKYIKLRAQGIYNDDVKAK-VRQ
SZ SSU    1 ----MSIFREK---GTFTRRQFFKGSGMLAATIVISGVFAKFGYDAWAA--------SDAYIEKRISGLYTLDEKMA-IRK
Dsp-SSU   1 ----MAIFYEK---DGINRRQFLKGAGAVIIAAAFPGIFT---KIGLGR-----THKSTEYIAQRAAGLYSLDEKMT-IRK

DdH-SSU  60 -----SQDNTQVKALYKSYLE----KPLGHKSHDLLHTHWFDKSKGVKELTTAGKLPNPRASEFEGPYPYE
DS-SSU   59 -----SQENPMIKKIYDKKHGFLHEGPCGHMSHRLLHTNYY----DRSARVKALEEKGVKLAI--------
TY-SSU   67 FKMAASHENPMIKRFYSEFAHH----PLSEVSEALLHTH---YKARV------------------------
CR-SSU   64 K-LIGSHQNPSCAQCYADL----NTEPMGEVAEKLLHTS-AYFDRKNLILKGASHA---------------
SW-SSU   67 G-LASSHNNPVVMSVYKTM----KAKPVDHTMHELLHTH--FYSRSMLAMTEAAHV---------------
SO-SSU   64 -----SHNNQAVTDVYKKFAQ----NPLSNLAEELFHTK-YV---DRTKLV--------------------
SZ SSU   64 -----SHQNPEILQIYKDFLSPGEVKPVSEKAHHLLHTK---YGQDIPELIKELH---AHQHDAA------
Dsp-SSU  64 -----SHENSEVLQLYKDFLSPGEVKPLSSTSHHLLHTK---YGKEVTAYIEELKDESAGKEKVAS-----
```

Fig.SI2-2| Multiple sequence alignments with large (a) and small subunits (b) of characteristic periplasmic [FeFe]-hydrogenases. Sequence elements which contribute to the stable H-cluster binding site closure mechanism upon $2Fe_H$ insertion are indicated in blue (Plug), pink (Lid) and red (Lock). Hydrophobic residues in the terminal extensions of LSU and SSU, which according to modelling data contribute to hydrophobic interactions between both extensions (see Fig.S2.3), are indicated in bold letters on gray background. DdH: *Desulfovibrio vulgaris subsp. vulgaris* str. Hildenborough; CR: *Campylobacter rectus* strain ATCC 33238; SW: *Sutterella wadsworthensis;* TY: *Thermodesulfovibrio yellowstonii* DSM 11347; DS: *Desulfonauticus submarinus* strain DSM 15269

IV.) Exemplary structure models for heterodimeric M2-type [FeFe]-hydrogenases

1.) *Desulfovibrio vulgaris subsp. vulgaris* str. Hildenborough (δ-Proteobacteria)

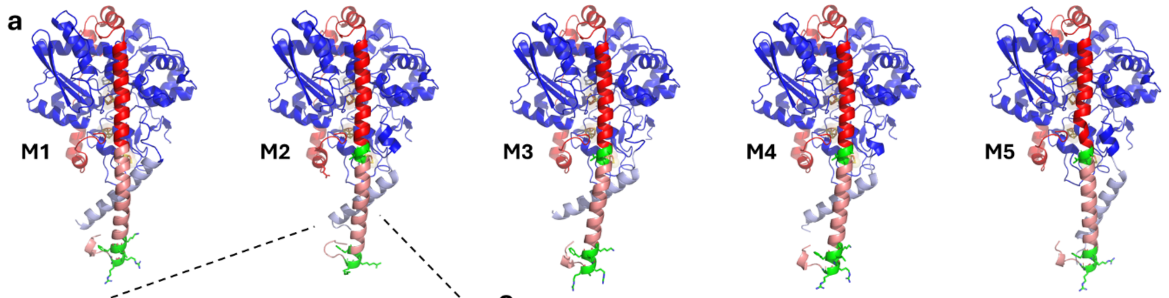

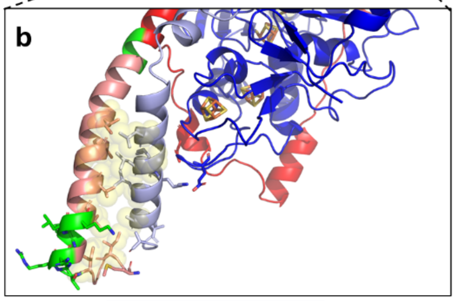

c

DdH-LSU

MSRTVMERIEYEMHTPDPKADPDKLHFVQIDEAKCIGCDTCSQYCPTAAIFGEM
GEPHSIPHIEACINCGQCLTHCPENAIYEAQSWVPEVEKKLKDGKVKCIAMPAPA
VRYALGDAFGMPVGSVTTGKMLAALQKLGFAHCWDTEFTADVTIWEEGSEFVER
LTKKSDMPLPQFTSCCPGWQKYAETYYPELLPHFSTCKSPIGMNGALAKTYGAER
MKYDPKQVYTVSIMPCIAKKYEGLRPELKSSGMRDIDATLTTRELAYMIKKAGIDFA
KLPDGKRDSLMGESTGGATIFGVTGGVMEAALRFAYEAVTGKKPDSWDFKAVRG
LDGIKEATVNVGGTDVKVAVVHGAKRFKQVCDDVKAGKSPYHFIEYMACPGGCV
CGGGQPVMPGVLEA↓MDRTTTRLYAGLKKRLAMASANKA

DdH-SSU

MQIASI**TRR**G**FLK**VACVTTGAALIGIRMTGK**AVA**A↓**VKQIKDYMLDRINGVYG**
ADAKFPVRASQDNTQVKALYKSYLEKPLGHKSHDLLHTHWFDKSKGVKELTTA
GKLPNPRASEFEGPYPYE

LSU **with** **C-terminal extension**
SSU with **N-terminal leader** including **TAT-consensus motif** (T/SRRXFLK) and **Sec avoidance motif** (**AXA**)

2.) *Campylobacter rectus* strain ATCC 33238 (ε-Proteobacteria)

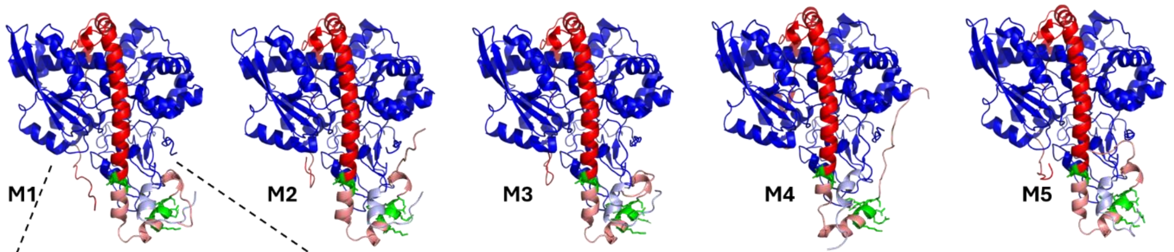

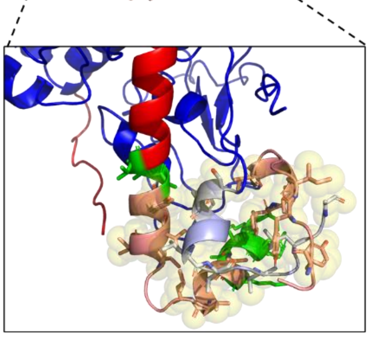

CR-LSU

MLSVYKIKTFPPAPNSRGGEADFEGTYRKGELRGIIRIDQDSCVGCDTCRSFCPTD
AIDGSLGVAHKIDQNLCVACGQCLINCPFAVIEQMSFVDEVMQKLDDEKTFVVAHP
SPAVRVSLAEEFGGKPGELTVNKMYNAFEKAGFNMYDVNFAADQTILEEGTELIKKI
KYWLLGERSHDLEHVSHHPFPHFTSCCPAWVRNAEIFHPELIPHISGAKSPIQMGG
PLAKTWAAKFVWDKDPRDIYVATVTPCTAKIYEASRPEFNSAYEYLKERGEIPADTK
SFPDIDATLTARDIAEILRKKGINPLEMSDEYPEKTMNVYTGGGTIFGNSGGVMEAA
LRTAYFLLSGQELKDPDLTPVRGYDKDLTEAVIPIPLKDYDGKTLELKVAVVNGASR
NLNTILKHITKDSNRYHFIEVMNCPGGCVNGGGQPVHAMGTSWLHSLLPLPLKA

CR-SSU

MRYQFIEKPVGKIF**SRR**DFL**K**VSGVLTSIIAISGY**AIT**DIIKRRKSYIAMRQEGLYKDD
KRCQDKKLIGSHQNPSCAQCYADLNTEPMGEVAEKLLHTSAYFDRKNLILKGASHA

3.) *Sporomusa ovata* (Bacillota; Negativicutes)

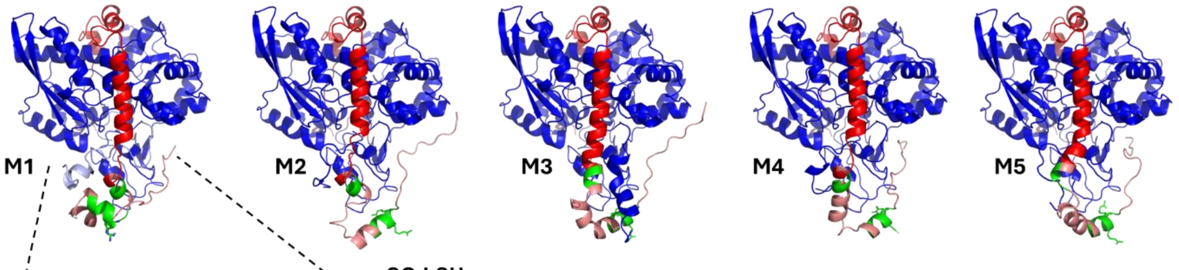

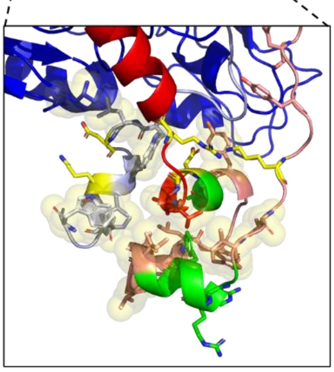

SO-LSU

MKGFQSQEVTRIIEIDSKTCKGCDSCKAFCPTDAIEGKYGAVHKINSEKCISCGQCLINC
PFGAPKDTVDVVDQVIDKLKNKKLTVVATIAPAVRVAIGEEFGMEPGSLITEKMYGAMK
QAGFKVLDTVFTADQTIMEEGMELIAKIRHYALGEPTEHHLGPLPQFTSCCPAWVRYAE
LYYPEVLPNMSSAKSPMMMAGALGKTYGAKEVWKVNPEDIFMVGVMPCTAKKFEASR
PEFKSASEYWKTQGRSGSYPDIDVVLTTRDLARLLKKLNIDIRTVAEFTDKDNPLAQYS
GAGTIFANTGGVMEAALRTAYFVITGKELDVLEFKPVRGLKGVKEASVTMVDAKTGKEV
TLKVAVAHGTKENVKPLLEEVKAGKSPYHFIEIMNCPAGCVNGGGQPINPMGTS**WLD**K
A**K**A**VL**P**W**S

SO-SSU

MAHYDYVE**K**A**V**KV**SRR**E**FIG**IVGVAGAILWTGAYV**AT**D**LV**QD**R**TKYIKLRAQGIYNDDV
KAKVRQSHNNQAVTDVYKKFAQNPLSNLAEELFHTKYVDRTKLV

4.) *Dehalobacter sp* TBBPA1 (Bacillota; Clostridia)

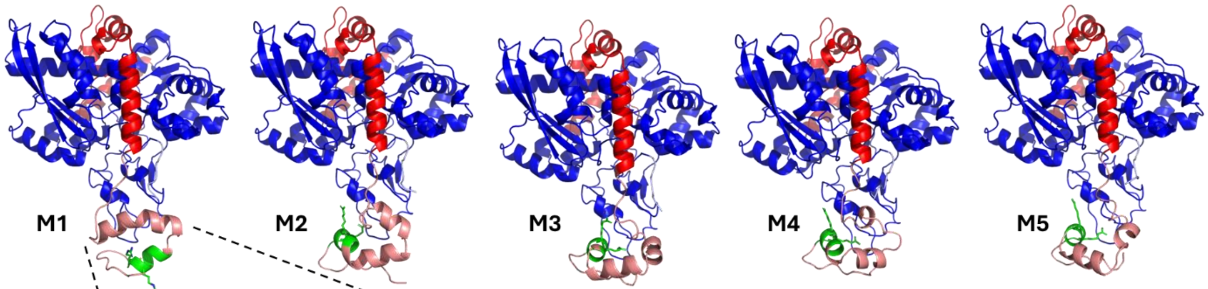

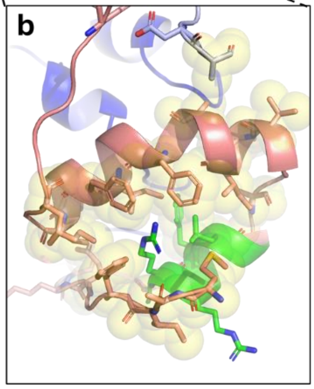

LSU

MSSLYAEKIIQISDRCRSCDHCTSVCPSGAIAGKLGEQHYIDHNKCLNCGQCLINCPAEIIKD
LSMAEEVKAALANPKKFVIVQTAPSVRVALGEEFGKAPGYNAKGKMFASLRKLGFDKVYD
TEFTADLTIMEEGSELINRVFKAVGQPGYETSGPLPQFTSCCPAWVKYAEDNYPKILPHVS
SAKSPQQMFGAVAKTYLPQKLNIEPADIFCVSVMPCTAKKYECSRPEMISSGYQDVNAVLT
TRELAQLIKEAGIDFDSLPEEAADSFVGQSTGAATIFGATGGVMEAALRTAYELLSGQSLEQ
IDFKAVRGLSPVREATVSIPIKALGGAVLPVKVAIVTGTKHVASLIEDVLAGRSNYHFIEVMN
CPGGCINGGGQ**PIRRDA**

SSU

MAIFYEKDGIN**RR**QFL**K**GAGAVIIAAAFPGIFTKIGLG↓RTHKSTEYIAQRAAGLYSLDEKMTI
RKSHENSEVLQLYKDFLSPGEVKPLSSTSHHLLHTKYGKEVTAYIEELKDESAGKEKVAS

Fig.SI2-3| Five plausible structure models of the assembled *Dd*HydAB^{WT} protein (1) and other heterodimeric M2-Type [FeFe]-hydrogenases (2-4) in their cytoplasmic pre-state with paired full-length LSU (blue) and SSU (red) subunits that include interacting N-terminal (brown) or C-terminal (blue) extensions prior to their translocation into the periplasm. Analogous to the function of the inserted peptide linkers in DdH-variants L2-L4, the structural configuration of the N-terminal segment preceding the lock-element in the native enzymatic pre-state may provide the balanced structural support needed for a rapid reconfiguration and closure of the H-cluster binding site after $2Fe_H$ insertion during *in vivo* maturation. Modelling was done applying the diffusion-based generative model Boltz-2 [8] (for details about the modelling data see **Supplementary Data File A**). (b) Zoomed view on the hydrophobic

interactions at inter- or intra-molecular crossing points along the extensions at corresponding large and small subunits. Polypeptide positions involved in these interactions are indicated as yellow letters in the corresponding sequences of SSU and LSU. The N-terminal part that cover the Tat-leader segment of the respective SSU sequence are marked by a brown background color, while the background of the likely C-terminal extension in the original LSU polypeptides is shown in blue. Amino acid positions involved in the Tat-transporter interaction are indicated by a green background color and a thin arrow marks the location of the cleavage site after the translocation step as indicated by SignalP6.

References:

1. **Y. Nicolet, C. Piras, P. Legrand, C. E. Hatchikian and J. C. Fontecilla-Camps**, Desulfovibrio desulfuricans iron hydrogenase: the structure shows unusual coordination to an active site Fe binuclear center. *Structure*, **1999**, *7*, 13–23. DOI:10.1016/S0969-2126(99)80005-7
2. **C. F. R. O. Matos, A. Di Cola and C. Robinson**, TatD is a central component of a Tat translocon initiated quality control system for exported FeS proteins in Escherichia coli. *EMBO Rep.*, **2009**, *10*, 474–479, DOI:10.1038/embor.2009.34.
3. **N. Blaudeck, P. Kreutzenbeck, R. Freudl and G. A. Sprenger**, Genetic Analysis of Pathway Specificity during Posttranslational Protein Translocation across the *Escherichia coli* Plasma Membrane. *J. Bacteriol.*, **2003**, *185*, 2811–2819, DOI:10.1128/JB.185.9.2811-2819.2003.
4. **A. C. Reis and H. M. Salis**, An automated model test system for systematic development and improvement of gene expression models. *ACS Synth. Biol.*, **2020**, *9*, 3145–3156, DOI:10.1021/acssynbio.0c00394.
5. **F. Teufel, J. J. Almagro Armenteros, A. R. Johansen, M. H. Gíslason, S. I. Pihl, K. D. Tsirigos, O. Winther, S. Brunak, G. von Heijne and H. Nielsen**, SignalP 6.0 predicts all five types of signal peptides using protein language models. *Nat. Biotechnol.*, **2022**, *40*, 1023–1025, DOI:10.1038/s41587-021-01156-3.
6. **E. C. Hatchikian, V. Magro, N. Forget, Y. Nicolet and J. C. Fontecilla-Camps**, Carboxy-terminal processing of the large subunit of [Fe] hydrogenase from *Desulfovibrio desulfuricans* ATCC 7757. *J. Bacteriol.*, **1999**, *181*, 2947–2952, DOI: 10.1128/JB.181.9.2947-2952.1999
7. **P. A. Lee, D. Tullman-Ercek and G. Georgiou**, The bacterial twin-arginine translocation pathway. *Annu. Rev. Microbiol.*, **2006**, *60*, 373–395, DOI:10.1146/annurev.micro.60.080805.142212.
8. **S. Passaro, G. Corso, J. Wohlwend, M. Reveiz, S. Thaler, V. Ram Somnath, N. Getz, T. Portnoi, J. Roy, H. Stark, D. Kwabi-Addo, D. Beaini, T. Jaakkola and R. Barzilay**, Boltz-2: Towards accurate and efficient binding affinity prediction. *bioRxiv*, **2025**, Preprint, DOI:10.1101/2025.06.14.659707.
9. **J. Felsenstein**, Confidence limits on phylogenies: An approach using the bootstrap. *Evolution Evolution*, **1985**, *39*, 783–791. DOI: 10.1111/j.1558-5646.1985.tb00420.x
10. **M. Nei and S. Kumar**, *Molecular Evolution and Phylogenetics*, Oxford University Press, New York, **2000**.Book DOI (online edition): 10.1093/oso/9780195135848.001.0001
11. **S. Kumar, G. Stecher, M. Li, C. Knyaz and K. Tamura**, MEGA X: Molecular Evolutionary Genetics Analysis across computing platforms. *Mol. Biol. Evol.*, **2018**, *35*, 1547–1549. DOI:10.1093/molbev/msy096.
